# The interaction of GRP78 and Zika virus E and NS1 proteins occurs in a chaperone-client manner

**DOI:** 10.1038/s41598-024-61195-z

**Published:** 2024-05-06

**Authors:** Wannapa Sornjai, Ploenphit Promma, Suphansa Priewkhiew, Suwipa Ramphan, Janejira Jaratsittisin, Pailin Jinagool, Nitwara Wikan, Michael Greenwood, David Murphy, Duncan R. Smith

**Affiliations:** 1 Molecular Pathology Laboratory, Institute of Molecular Biosciences, Mahidol University, 25/25 Phutthamonthon Sai 4 Road, Salaya, Nakhon Pathom, 73170 Thailand; 2 Department of Pharmacology, Faculty of Medicine, Chiang Mai University, Chiang Mai, 50200 Thailand; 3 Molecular Neuroendocrinology Research Group, Bristol Medical School, Translational Health Sciences, University of Bristol, Bristol, UK

**Keywords:** Zika virus, Chaperone, GRP78, Critical residues, ZIKV E, ZIKV NS1, GRP78 inhibitor, Pathogens, Mechanisms of disease, Virology

## Abstract

Glucose regulated protein 78 (GRP78) is a chaperone protein that is a central mediator of the unfolded protein response, a key cellular stress response pathway. GRP78 has been shown to be critically required for infection and replication of a number of flaviviruses, and to interact with both non-structural (NS) and structural flavivirus proteins. However, the nature of the specific interaction between GRP78 and viral proteins remains largely unknown. This study aimed to characterize the binding domain and critical amino acid residues that mediate the interaction of GRP78 to ZIKV E and NS1 proteins. Recombinant EGFP fused GRP78 and individual subdomains (the nucleotide binding domain (NBD) and the substrate binding domain (SBD)) were used as a bait protein and co-expressed with full length or truncated ZIKV E and NS1 proteins in HEK293T/17 cells. Protein–protein interactions were determined by a co-immunoprecipitation assay. From the results, both the NBD and the SBD of GRP78 were crucial for an effective interaction. Single amino acid substitutions in the SBD showed that R492E and T518A mutants significantly reduced the binding affinity of GRP78 to ZIKV E and NS1 proteins. Notably, the interaction of GRP78 with ZIKV E was stably maintained against various single amino acid substitutions on ZIKV E domain III and with all truncated ZIKV E and NS1 proteins. Collectively, the results suggest that the principal binding between GRP78 and viral proteins is mainly a classic canonical chaperone protein-client interaction. The blocking of GRP78 chaperone function effectively inhibited ZIKV infection and replication in neuronal progenitor cells. Our findings reveal that GRP78 is a potential host target for anti-ZIKV therapeutics.

## Introduction

Zika virus (ZIKV) is a positive sense single-stranded RNA virus, a member of the family *Flaviviridae*, genus *Flavivirus* together with other pathogenic viruses such as dengue virus (DENV), Japanese encephalitis virus (JEV), yellow fever virus and West Nile virus (WNV). ZIKV was first identified in a rhesus macaque in the Zika Forest of Uganda in 1947^1^ and the first case of human infection which occurred in the same area was reported in 1964^2,3^. Recently, ZIKV emerged globally and spread throughout Southeast Asia, the Pacific islands, the Americas, and the Caribbean^4^. This virus is transmitted to humans mainly by *Aedes aegypti* mosquitoes^5^, but also through sexual, transfusion-based and vertical transmission routes^6^. Most cases of infection are asymptomatic, or result in a mild self-limiting illness, however some adults may develop neurological complications such as Guillain-Barré syndrome^7^. ZIKV infection of a pregnant woman is a significant concern because the virus can pass to the developing foetus, sometimes resulting in a spectrum of severe neurological anomalies termed congenital Zika syndrome (CZS)^8^.

ZIKV is classified into two major lineages, an African and an Asian lineage^9^. The genome is approximately 11 Kb, encoding for 3 structural proteins; capsid, prM, envelope (E) and 7 non-structural (NS) proteins; NS1, NS2A, NS2B, NS3, NS4A, NS4B and NS5. The mature virion consists of a nucleocapsid surrounded by the envelope (E) and prM proteins embedded in a host derived membrane bilayer. The ZIKV E protein plays a key role in receptor binding and membrane fusion which mediate viral entry into host cells, and is a major target of host immune recognition. ZIKV E protein is composed of three major domains, a β-barrel-shaped domain I at the N-terminus, an elongated finger-like structure (domain II) and an immunoglobulin-like domain III at the C-terminus^10^. In addition to the structural proteins, the non-structural proteins are crucially required for viral replication. NS1 protein is a glycoprotein that is also composed of three distinct domains; a β-roll at the N-terminus, a wing domain and a β-ladder domain at the C-terminus^11,12^. NS1 proteins are formed as an embedded homodimer on the ER membrane that is essential for viral genome replication and viral assembly^13^. NS1 is also expressed on the cell surface and secreted from infected cells as a hexameric lipoparticle^14,15^. NS1 is strongly immunogenic, and is believed to participates in immune evasion via the complement system, and is potentially implicated in the overall pathogenesis of infection^16–20^.

Viruses are obligate parasites as their infection and replication are solely dependent upon the host cell machineries. The virus interacts with host molecules through a number of mechanisms including protein–protein interactions, to manipulate the host cell metabolism, and to create a favourable environment for effective replication^21^. GRP78, also known as binding immunoglobulin protein (Bip), is a member of the heat shock protein 70 (HSP70) superfamily^22–25^. GRP78 is highly conserved and is the most abundant chaperone protein. It performs a number of roles in maintaining protein homeostasis by facilitating protein folding, assembly, translocation of proteins to target organelles and cooperating with the cellular protein degradation pathway^26^. GRP78 is mainly located in the ER where it functions as a chaperone protein, but GRP78 is also found in mitochondria and the nucleus, as well as being expressed on the cell surface, and additionally is secreted into the circulation^27^. GRP78 consists of two major functional domains, an N-terminal nucleotide binding domain (NBD) and a C-terminal substrate binding domain (SBD) which are connected by a hydrophobic linker. The NBD contains an ATPase catalytic domain, while the SBD contains two subdomains, SBDβ forming a substrate binding pocket, and SBDα forming a coverlid. GRP78 and other HSP70s proteins generally bind to hydrophobic exposed polypeptides of the client protein, and allosteric coupling between the NBD and SBD upon ATP hydrolysis and substrate binding regulates the chaperone kinetic cycle^28^.

GRP78 is additionally the master regulator of the unfolded protein response (UPR). ER stress is activated during protein overload in the ER lumen, leading to activation of a cellular response to restore ER homeostasis by increasing protein folding, attenuating protein translation and accelerating misfolded protein degradation^29,30^. GRP78 responds to ER stress through three major transmembrane signalling proteins, activating transcription factor 6 (ATF6), inositol-requiring kinase 1 (IRE1) and protein kinase RNA-like endoplasmic reticulum kinase (PERK)^31^. GRP78 binds to the luminal domain of these transducers, and when unfolded proteins accumulate in the ER, it results in the dissociation of GRP78 from these sensors. The dissociated transducers activate pathways that promote processes to alleviate the stress, and numerous studies have shown that flavivirus infection activates the UPR in infected host cells^32–36^. When the stress is prolonged and unresolved, the UPR will promote cell death by activating the CCAAT/-enhancer-binding protein homologous protein (CHOP), resulting in subsequent apoptosis^37–41^.

GRP78 has been reported to play a significant role in the replication of a number of flaviviruses (Table 1) including DENV^42–44^, JEV^45–48^ and ZIKV^49–52^. Specific interactions have been reported between GRP78 and DENV E protein^43,53^, JEV E protein^46^, ZIKV E protein^49–51^ as well as DENV^54^ and ZIKV NS1^49^. This latter study also identified interactions between GRP78 and ZIKV NS2B, NS3, NS5 and C protein^49^. In some studies a role for GRP78 as a receptor protein mediating internalization to mammalian cells has been proposed for DENV^55^, JEV^46^, ZIKV^50^ and Tembusu virus (TMUV)^56^. However, it should be noted that contradictory results have been published for the role of GRP78 as a receptor protein for ZIKV, with some studies showing a receptor role for GRP78^50^, while others do not^49^.

**Table 1:** Studies showing involvement of GRP78 in flaviviral infections.

References	The role of GRP78 in Flavivirus infection		
	Viral replication	Receptor	Specifically interacts with flaviviral protein/s
DENV			
Diwaker et al. 2015^42^	GRP78 inhibitor decreased DENV E protein expression in infected K562 cells		
Jindadamrongwech et al. 2004^55^		Antibody blocking identified GRP78 as the receptor for DENV 2 on the HepG2 cells	
Limjindaporn et al. 2009^43^	GRP78 knockdown reduced DENV production		Yeast two hybrid and co-IP showed GRP78 interact with DENV E domain III
Wati et al. 2009^44^	GRP78 cleavage with toxin reduced viral proteins and viral production		
Jitobaom et al. 2016^53^			Co-IP, GRP78 interacts with DENV E and VDAC protein
Songprakhon et al. 2018^54^	GRP78 knockdown by siRNA reduced DENV NS1 production and secretion		DENV NS1 interacted with SBD of GRP78 protein
JEV			
Mukherjee et al. 2017^45^	GRP78 knockdown by siRNA reduced viral production		
Nain et al. 2017^46^	GRP78 cleavage by toxin reduced JEV protein synthesis and viral replication	Antibody blocking against GRP78 inhibited JEV entry into cells	GRP78 interacts with E protein domain III of JEV
Wang et al. 2016^47^	GRP78 mediate the trafficking of JEV E protein and virions		
Wu et al. 2011^48^	GRP78 knockdown decreased JEV production		
ZIKV			
Khongwichit et al. 2021^50^	GRP78 knockdown decreased ZIKV production	Antibody blocking reduced ZIKV entry	GRP78 Interacts with to ZIKV E protein domain III
Royle et al. 2020^51^	GRP78 knockdown decreased ZIKV protein and viral production		ZIKV E interacted with GRP78
Turpin et al. 2020^52^	ZIKV infection induced ER stress and manipulated UPR by down regulation of GRP78 expression		
Chen et al. 2020^49^	GRP78 promoted ZIKV infection and mediated the interaction between ALPP and ZIKV proteins		Co-IP, GRP78 directly interacts with ZIKV proteins including ZIKV E and NS1
TMUV			
Zhao et al. 2018^56^		Antibody blocking identified GRP78 as the receptor for TMUV entry into BHK-21 cells	

While GRP78 is critically required for flavivirus infection and replication^57^, few studies have probed the nature of the interaction between GRP78 and flaviviral proteins. Interestingly, GRP78 was first identified as an immunoglobulin binding protein (BiP)^22^ and studies have shown that GRP78/BiP can interact with both the light and heavy chains of immunoglobulins^22,58^. Importantly, studies have shown that the flavivirus E proteins contain an immunoglobulin-like β-barrel structure in domain III that is exposed on the viral surface and contains cellular-binding motifs^10,59^. One important question therefore is whether the interaction between GRP78/BiP and the ZIKV E protein is defined as a “lock and key” mechanism, or is a classic chaperone/client interaction. Given that a “lock and key” type interaction could induce the UPR earlier in infection, activating anti-apoptotic pathways early in infection, as opposed to a chaperone/client interaction that would necessitate the UPR being activated due to an influx of proteins, the matter is of some importance. In this study therefore we sought to define the molecular interaction between GRP78 and ZIKV E and NS1 proteins through in vivo protein–protein interaction and co-immunoprecipitation assays, and show that the GRP78 SBD amino acids R492 and T518 are crucial residues for the interaction with both viral proteins. Importantly, both full length proteins and truncated ZIKV E and NS1 proteins interacted with GRP78 protein. Our study demonstrates that GRP78 principally interacts with ZIKV proteins via a classic chaperone-client binding, and not through any “lock and key” mechanism. Blocking GRP78 chaperone function by HA15 significantly dampened ZIKV infection and replication in host cells, and suggests possible new therapeutic pathways.

## Materials and methods

### Cell line culture

The human embryonic kidney cell line HEK293T/17 (ATCC: CRL-11268) and human lung carcinoma cell line A549 (ATCC: CCL-185) were grown in Dulbecco's Modified Eagle's Medium (DMEM; Gibco, Waltham, MA) supplemented with 10% heat-inactivated fetal bovine serum (FBS; Gibco). The African green monkey kidney cell line Vero (ATCC: CCL-81) was grown in DMEM supplemented with 5% heat-inactivated FBS. All mammalian cell lines were cultured in an incubator maintained at 37 °C with 5% CO_2_ and a humidified atmosphere. The *Aedes albopictus* mosquito cell line C6/36 (ATCC: CRL-1660) was grown in Minimum Essential Medium (MEM; Gibco) supplemented with 10% heat-inactivated FBS and incubated at 28 °C in an incubator.

### Neural progenitor cell differentiation and culture

Human induced pluripotent stem cells (hiPSCs) were kindly provided by Associate Professor Methichit Wattanapanitch (Siriraj Center for Regenerative Medicine, Faculty of Medicine Siriraj Hospital, Mahidol University, Thailand)^60^. The hiPSCs were induced to differentiate into neuronal progenitor cells (NPCs) following STEMdiff Neural system instruction (STEMCELL Technologies, Vancouver, Canada). Briefly, hiPSCs were dissociated by ACCUTASE cell detachment solution (STEMCELL Technologies) and then plated into Aggrewell 800 plates (STEMCELL Technologies) in STEMdiff Neural induction Media containing 10 µM ROCK inhibitor (Y-27632, STEMCELL Technologies). Induced hiPSCs were incubated at 37 °C with 5% CO_2_ and the media was partially replaced daily with STEMdiff Neural induction Media. At day 5 of incubation, the formed embryoid bodies were plated onto the Matrigel coated 6-well plates and incubated at 37 °C with 5% CO_2_ with a daily media replacement until the neural rosettes were clearly formed. The neural rosettes were selected by STEMdiff Neural Rosette Selection Reagent (STEMCELL Technologies) and then plated onto Matrigel coated 6-well plates and subsequently incubated at 37 °C with 5% CO_2_ with a daily media replacement until 100% cell confluency was obtained. The confluent NPCs were dissociated by pipetting and grown onto Matrigel coated 6-well plates in DMEM/F12 (HyClone, Logan, UT) supplemented with 0.5X B27 (Invitrogen, Waltham, MA), 0.5Χ N2 (Invitrogen), 1X GlutaMAX (Gibco), 1X antibiotic–antimycotic (Gibco), and 20 ng/mL fibroblast growth factor 2 (FGF2) (Sigma, St. Louis, MO). NPCs were incubated at 37 °C with 5% CO_2_ and a humidified atmosphere. Complete media was replaced every other day and cells were passaged using ACCUTASE enzyme when they reached confluency.

### Virus production

ZIKV Thai strain SV0010/15 (Accession number KX051562) was kindly provided by the Armed Forces Research Institution of Medical Sciences (AFRIMS) and the Development of Disease Control, Ministry of Public Health, Thailand. The virus was inoculated and propagated in C6/36 cells and the virus supernatant was harvested from infected cells when cytopathic effects were clearly seen. The supernatant was centrifuged at 1000 *g* for 5 min to remove cell debris and kept at − 80 °C as the virus stock. The virus titer was quantitated by plaque assay.

### Plaque assay

Vero cells were seeded onto 6-well culture plates to reach 80% confluence after overnight incubation at 37 ºC with 5% CO_2_. ZIKV stock or infected supernatant was tenfold serially diluted in BA-1 medium (1X Medium 199, 1% BSA, 50 mM Tris–HCl pH 7.6, 100 unit/mL penicillin–streptomycin). Vero cells were inoculated with 200 µL of dilute virus sample and incubated at 37 °C for 2 h. After inoculation, infected cells were overlaid with 1.2% methyl cellulose (Sigma Aldrich, Rahway, NJ) containing 1X DMEM and 2% heat inactivated-FBS and then incubated at 37 °C and 5% CO_2_ for 7 days. Infected cells were fixed with 3.7% formaldehyde and subsequently stained with 0.5% crystal violet staining solution. Plaque number was counted and calculated as plaque forming units (PFU)/mL.

### RNA extraction and cDNA synthesis

Total mRNA was extracted from HEK293T/17 cells and ZIKV genome was extracted from virus stock using TRIzol reagent (Invitrogen) according to the manufacturer’s instructions. Complementary DNA was synthesized using the extracted RNA as a template by RevertAid RT reverse transcriptase kit (Thermo Fisher Scientific) and oligo dT (Bio Basic, Inc., Ontario, Canada) or random hexamer (Invitrogen) following the instruction’s protocol.

### Recombinant plasmid construction

A plasmid carrying the coding sequences of ZIKV capsid-prM-E and NS1 was commercially synthesized by GenScript (Piscataway, NJ) based on the sequence of ZIKV isolate FSS13025 (Accession number AFD30972.1) with codon optimization for efficient protein expression in mammalian cells. The commercialized coding sequence was used as the template for DNA fragment amplification. The mammalian expression vector pcDNA3.1 + _19CprME-Zika containing ZIKV prM-E sequences has been described in previous studies^61,62^. Both full length and subdomains of ZIKV E or ZIKV NS1 coding sequence were amplified using Phusion High-Fidelity DNA polymerase (Thermo Fisher Scientific) and specific primers as given in Supplemental Table S1. The DNA fragments were digested and ligated into pcDNA3.1 ( +) plasmid. Full length and isolated nucleotide binding domain (NBD) and substrate binding domain (SBD) of human GRP78 were amplified from HEK293T/17 cDNA using specific primers (Supplemental Table S1). The DNA fragments were digested and ligated into pEGFP-C2 (Clontech, Mountain View, CA) or pCDH-EF1-FHC (#64874, Addgene, Watertown, MA) plasmids. All ligated products were transformed into DH5α competent *E. coli* by the heat shock method. Transformed cells were grown on appropriate antibiotic selective LB agar. Bacterial colonies were screened by colony PCR and FastDigest Restriction Enzymes (Thermo Fisher Scientific) according to the manufacturer’s protocol. Nucleotide sequences of all constructs were confirmed by commercial sequencing analysis (Macrogen, Seoul, Republic of Korea or 1st base, Selangor DE, Malaysia).

### Bacteria culture and plasmid preparation

DH5α *E. coli* recombinants were grown in LB broth supplemented with 50 μg/mL of kanamycin or 50 μg/mL of ampicillin in an incubator maintained at 37 °C with a constant shaking at 220 rpm for 15 h. Bacteria culture was harvested and the recombinant plasmids were purified using FavorPrep™ plasmid purification kit (Favorgen, Ping-Tung, Taiwan) according to the manufacturer’s protocol.

### QuikChange site directed mutagenesis

Single mutations in the SBD of GRP78 or domain III of ZIKV E were introduced by QuikChange site directed mutagenesis. The wild type pEGFP-full length GRP78 and pcDNA ZIKV E-HA plasmid were used as the template. The mutated plasmid was synthesized using 1X HF buffer, 10 ng wild type plasmid, 250 µM dNTPs, 0.38 µM of each mutagenic primer (Supplemental Tables S2 and S3) and 0.025 unit/µL of Phusion High-Fidelity DNA polymerase (Thermo Fisher Scientific). The PCR reaction was carried out with an initial denaturation at 98 °C for 30 s and followed by 20 cycles of denaturation at 98 °C for 10 s, annealing at 60 °C for 30 s, extension at 72 °C for 2 min and a final elongation at 72 °C for 5 min. The methylated parental plasmid (wild type) was digested using *DpnI* restriction enzyme (Thermo Fisher Scientific) treatment following the manufacturer’s protocol. The treated product was subsequently transformed into DH5α competent *E. coli* by the heat shock method. Mutated nucleotide sequences were confirmed by commercial DNA sequencing analysis.

### Plasmid transfection by calcium phosphate method

HEK293T/17 cells were seed onto 100 mm^2^ cell culture dishes and cultured in antibiotic free-DMEM supplemented with 10% heat-inactivated FBS at a density that allowed approximately 60% confluence to be reached within 24 h. A total of 15 µg of each plasmid was diluted in sterile water and then mixed with an equal volume of 2X Hanks buffered saline solution and 36 µL of 2.5 M CaCl_2_ solution. The mixture was incubated at room temperature for 20 min for the formation of the DNA-calcium phosphate complex. The incubated reaction was gently dropped onto the surface of the culture media and the transfected cells were subsequently incubated at 37 °C with 5% CO_2_ until required.

### Lentiviral packaging and cell transduction

For lentivirus production, HEK293T/17 cells were transfected with either 1200 ng of wild type or R492E or T518A mutated pCDH-GRP78 or pCDH-empty plasmid along with 300 ng of pMD2.G plasmid (#12259, Addgene) and 900 ng of psPAX2 plasmid (#12260, Addgene) using Lipofectamine 3000 (Invitrogen) following the manufacturing protocol. The culture media was replaced at 14 h after transfection and transfected cells were incubated in 37 °C with 5% CO_2_ incubator. The lentivirus supernatant was harvested at 72 h post transfection by centrifugation at 1000 g for 5 min to remove cell debris and kept at − 80 °C until required.

A549 cells were transduced with 25 μL of lentivirus supernatant either carrying wild type or R492E or T518A mutated GRP78 or empty lentivirus as control vector. Transduced A549 cells were incubated at 37 °C with 5% CO_2_. After 48 h of transduction, transduced cells were selected with DMEM containing 10% heat inactivated- FBS and 3 μg/mL puromycin. Single clones of stable expressed wild type or mutated GRP78-A459 cells were isolated by limiting dilution and the selected A549 clones were maintained in DMEM containing 10% heat inactivated- FBS and 1.5 μg/mL puromycin during the experiment.

### Immunoprecipitation assay

Transfected HEK293T/17 cells were washed twice with ice cooled 1X PBS and lysed with 1 mL of IP lysis buffer (50 mM HEPES pH 7.4, 150 mM NaCl, 1 mM EGTA, 10% glycerol, 1% Triton X-100, 1X protease inhibitor cocktail). Cell lysate was immediately rotated at 4 °C for 45 min and subsequently centrifuged at 10,000 g with 4 °C for 10 min to remove DNA and cell debris. Protein lysate was incubated with 10 µL of equilibrated GFP-Trap beads (ProteinTek GmbH, Planegg-Martinsried, Germany) at 4 °C for 1 h with a constant rotation. After incubation, beads were centrifuged and washed with IP washing buffer (50 mM HEPES pH 7.4, 150 mM NaCl, 1 mM EGTA, 10% glycerol, 1% triton X-100). Immunoprecipitated proteins were eluted from the GFP beads by suspension in 35 μL of 2.5X protein loading buffer (0.15 M Tris–HCl pH 6.8, 2.5% SDS, 25% glycerol and 0.0075% bromophenol blue) containing 40 mM DTT and heating at 90 °C for 15 min. Sample was briefly incubated on ice and the eluted protein supernatant was subjected to western blot analysis.

### Western blot analysis

Total protein lysate and immunoprecipitated proteins were separated by electrophoresis through 12% SDS-PAGE gels. Separated proteins were subsequently transferred onto nitrocellulose membranes which were subsequently blocked with 5% skimmed milk in 1X TBS containing 0.05% Tween. Membranes were incubated with a rabbit polyclonal anti-ZIKV envelope antibody (GTX133326, GeneTex, Irvine, CA), or a rabbit polyclonal anti-ZIKV NS1 antibody (GTX133307, GeneTex), or a mouse monoclonal anti-HA tag antibody (26183, Invitrogen), or a rabbit polyclonal anti-GFP antibody (sc-8334, Santa Cruz, Dallas, TX), or a rabbit polyclonal anti-GRP78 antibody (ab21685, Abcam, Cambridge, UK), or a mouse monoclonal anti-ATF6 antibody (ab11909, Abcam) or a goat polyclonal anti-β actin antibody (sc-1616, Santa Cruz) in 5% skimmed milk overnight. The excess primary antibodies were removed and membranes were incubated with either a HRP labelled goat anti-rabbit IgG (31460, Thermo Fisher Scientific), or a HRP labelled rabbit anti-goat IgG (31402, Thermo Fisher Scientific) or a HRP labelled rabbit anti-mouse IgG (A9044, Sigma-Aldrich, Darmstadt, Germany) for 1 h. Some membranes were directly probed with a HRP labelled mouse monoclonal anti-β actin antibody (sc-8432 HRP, Sata Cruz) or a HRP labelled mouse monoclonal anti-GFP antibody (sc-9996 HRP, Sata Cruz). Immunochemiluminescent signal was developed using Luminata Forte Western HRP substrate (Merck, Darmstadt, Germany) and signal was recorded using an Azure Biosystem C400 (Azure Biosystems, Inc., Dublin, CA) or X-ray film.

### MTT assay

A549 cells or NPCs were seeded onto 96-well plate and incubated at 37 ºC with 5% CO_2_ in an incubator overnight. Monolayer cells were treated with the completed media containing a various concentration of HA15 (MedChemExpress, Monmouth Junction, NJ) or DMSO and incubated at 37 °C with 5% CO_2_ in an incubator for 24 h. After that, treated cells were incubated with 12.5 μL of 4 mg/mL MTT dye for 1 or 2 h. After incubation, the formazan crystal was dissolved with 100 μL of DMSO and the absorbance was measured at 590 nm. Cell viability was calculated and compared to complete media as a control.

### HA15 treatment and ZIKV infection

HA15 (MedChemExpress) was dissolved in DMSO (Sigma Aldrich) and kept at − 30 °C until required. A549 cells or NPCs were incubated with the complete media containing various concentrations of HA15 or the corresponding concentration of DMSO as a control for 3 h in 37 °C with 5% CO_2_ incubator. The compound was removed and treated cells were inoculated with ZIKV Thai strain SV0010/15 at the required MOI for 2 h. After virus inoculation, the unbound virus was removed and replaced with complete media containing HA15 or DMSO. The infected cells were maintained at 37 °C with 5% CO_2_.

### Indirect immunofluorescence assay (IFA)

NPCs were seeded onto Matrigel coated glass coverslip and 80% confluent cells or mock or transfected HEK293T/17 cells were fixed with 4% paraformaldehyde. Fixed cells were permeabilized with 0.3% triton X-100 in 1X PBS for IFA (154 mM Sodium chloride, 50 mM disodium hydrogen phosphate, 50 mM sodium dihydrogen phosphate, pH 7.4) for 10 min and subsequently blocked with 2% fetal bovine serum (Gibco) in 1X PBS for 1 h. Blocked cells were incubated with primary antibodies; a rabbit anti-human PAX-6 antibody (60094, STEMCELL Technologies), a mouse anti-human nestin antibody (60091, STEMCELL Technologies), a goat anti-SOX1 antibody (AF3369, R&D systems, Inc., Minneapolis, MN), a rabbit anti-musachi1 antibody (AB52865, Abcam, Cambridge, UK), a mouse anti-GFAP antibody (MA512023, Invitrogen), a mouse anti-Oct-3/4 antibody (SC-5279, Santa Cruz Biotechnology), a rabbit anti-calnexin antibody (ab92516, Abcam), or a goat anti-GRP78 antibody (sc-1050, Santa Cruz) for overnight. Unbound primary antibodies were removed by washing with 1X PBS containing 0.03% Triton X-100. Cells were subsequently incubated with DAPI (Invitrogen) for nuclear staining and the corresponding fluorescence conjugated secondary antibodies, an Alexa Fluor 647 labelled goat anti-human IgG (A21445, Invitrogen), an Alexa Fluor 488 labelled donkey anti-mouse IgG (A21202, Invitrogen), an Alexa Fluor 568 labelled donkey anti-goat IgG (A11057, Invitrogen) or an Alexa Fluor 647 labelled donkey anti-rabbit IgG (A31573, Invitrogen) for 1 h. Stained cells were mounted with ProLong (Invitrogen) and the fluorescence signal was observed under Zeiss LSM 800 confocal laser scanning microscope (Zeiss Group, Oberkochen, Germany).

### Statistical analysis

Western blot protein band intensities were quantitated using Quantity One software (Bio-Rad Laboratories, Hercules, CA). The co-immunoprecipitation ratio, protein expression, cell viability and virus titer were tested for significance using independent sample T-test by PASW statistics 18 (PASW Statistics for Windows, Version 18.0. SPSS Inc., Chicago, IL). P-values of less than 0.05 were considered as significant.

## Results

### ZIKV E and ZIKV NS1 protein effectively interact with full length GRP78 protein

**Figure 1:**
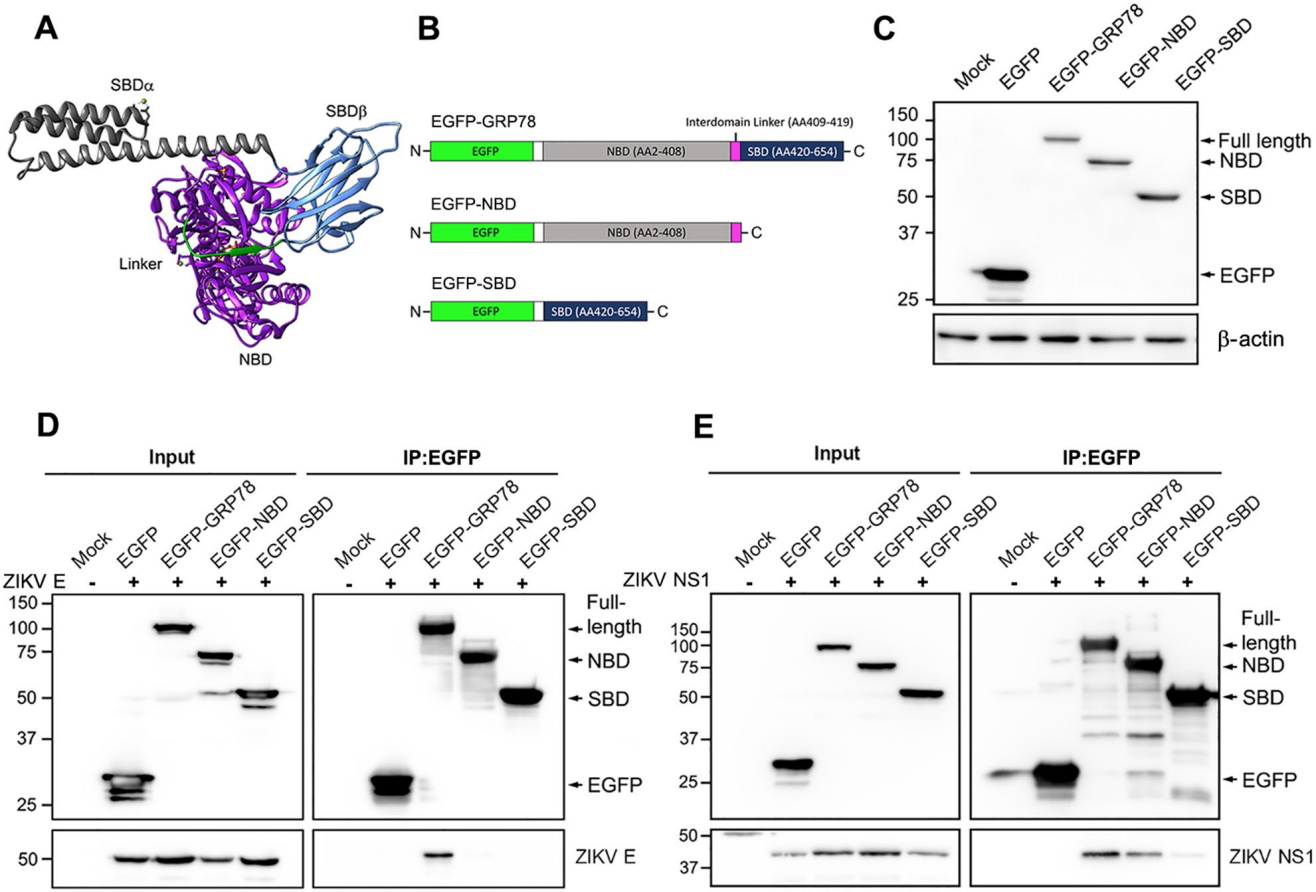
Interaction of human GRP78 with ZIKV E and NS1 proteins. (**A**) The structure of the ATP bound state of human GRP78 (PDB ID: 5E84) which contains two major functional domains the nucleotide binding domain (NBD; purple) and the substrate binding domain (SBD; grey and cornflower blue). These two domains are connected via the interdomain linker (green). The substrate binding domain consists of the SBDα (grey) and SBDβ (cornflower blue). (**B**) A schematic diagram illustrating the constructs for a full length EGFP fused GRP78 (EGFP-GRP78), and an isolated nucleotide binding domain of GRP78 (EGFP-NBD) and an isolated substrate binding domain of GRP78 (EGFP-SBD). (**C**) HEK293T/17 cells were either mock transfected or transfected with a pEGFP-C2 empty plasmid (EGFP), pEGFP-full length GRP78 (EGFP-GRP78), pEGFP-NBD GRP78 (EGFP-NBD) or pEGFP-SBD GRP78 (EGFP-SBD) plasmid. At 48 h post transfection, the expression of recombinant proteins was detected by western blot analysis. (**D**) HEK293T/17 cells were co-transfected with each individual recombinant plasmid pEGFP-C2 (EGFP), pEGFP-full length GRP78 (EGFP-GRP78), pEGFP-NBD GRP78 (EGFP-NBD) and pEGFP-SBD GRP78 (EGFP-SBD)) and with pcDNA3.1 + _19CprME Zika plasmid or (**E**) pcDNA3.1 + _ZIKV NS1 plasmid. Mock transfected cells (Mock) were used as a negative transfection control. At 48 h post transfection, mock and transfected cells were harvested and the interaction of EGFP-GRP78 fusion proteins (full length GRP78, NBD and SBD) and their interacting proteins was determined by an immunoprecipitation assay using GFP trap beads. Total protein lysate (Input) and the immunoprecipitated proteins (IP: EGFP) were examined for the presence of ZIKV E, ZIKV NS1 and GRP78 (full length, NBD and SBD) by western blot analysis. Panels C, D and E consist of composite images. Different western blots are separated by white vertical lines, and subsequent probings are separated by white horizontal lines. Full uncropped western blot images can be found in the supplemental materials.

GRP78 is composed of two major functional domains, namely the NBD and the SBD, which are connected by a hydrophobic peptide linker (Fig. 1A). The endogenous GRP78 protein in HEK293T/17 cells was colocalized with calnexin in the ER (Pearson’s coefficient = 0.3705) (Supplemental Fig. S1). Several studies have shown that both ZIKV E^49–51^ and NS1^49^ proteins interact with GRP78, the nature of the interaction remains largely undefined. To investigate this, a mammalian protein expression system was used for a protein–protein interaction assay. Mammalian expression plasmids were constructed to express a full length GRP78 (amino acids 2–654), the GRP78 NBD with the hydrophobic linker (amino acid 2–419) and the GRP78 SBD (amino acid 420–654). All recombinant proteins were fused with EGFP at the N-terminus and were used as bait proteins (Fig. 1B). HEK293T/17 cells were transfected with the recombinant plasmids for 24 h and all proteins were successfully expressed (Fig. 1C). The recombinant EGFP-full length GRP78, EGFP-NDB and EGFP-SBD proteins were expressed throughout the cells both cytoplasm and ER (Supplemental Fig. S2), covering all possible interactions with ZIKV E and NS1 protein both inside and outside the ER. However, Pearson’s coefficients with calnexin were not vastly different from endogenous GRP78 (Pearson’s coefficients 0.3705), (Pearson's Coefficient of full length GRP78-calnexin = 0.2314, SBD-calnexin = 0.2004, NBD-calnexin = 0.2097).

After that, individual proteins (EGFP-full length GRP78, EGFP-NBD or EGFP-SBD) were co-expressed with ZIKV E or ZIKV NS1 protein in HEK293T/17 cells. Protein lysate was collected at 48 h post-transfection, following which protein–protein interactions were investigated by an immunoprecipitation assay using GFP Trap beads. Pulled down proteins were eluted and subsequently analyzed by western blot analysis. From the results, ZIKV E protein was pulled down by EGFP-full length GRP78, but not with the two isolated subdomains (Fig. 1D). Interestingly, ZIKV NS1 protein was pulled down with the full length GRP78, and also by the two isolated subdomains (Fig. 1E). However, while a robust signal of ZIKV NS1 was seen after co-immunoprecipitation with the EGFP-full length GRP78 construct, the signal appeared to be markedly reduced in the co-precipitation with the EGFP-NBD construct and the EGFP-SBD construct (Fig. 1E). The results confirmed that both ZIKV E and NS1 proteins interact with GRP78, and while the significance of the reduced interaction between the isolated domains of GRP78 with ZIKV NS1 remains unclear, the results show that for a robust interaction, both domains are required.

### The SBD contains critical amino acid residues for the ZIKV E and ZIKV NS1 interaction

**Figure 2:**
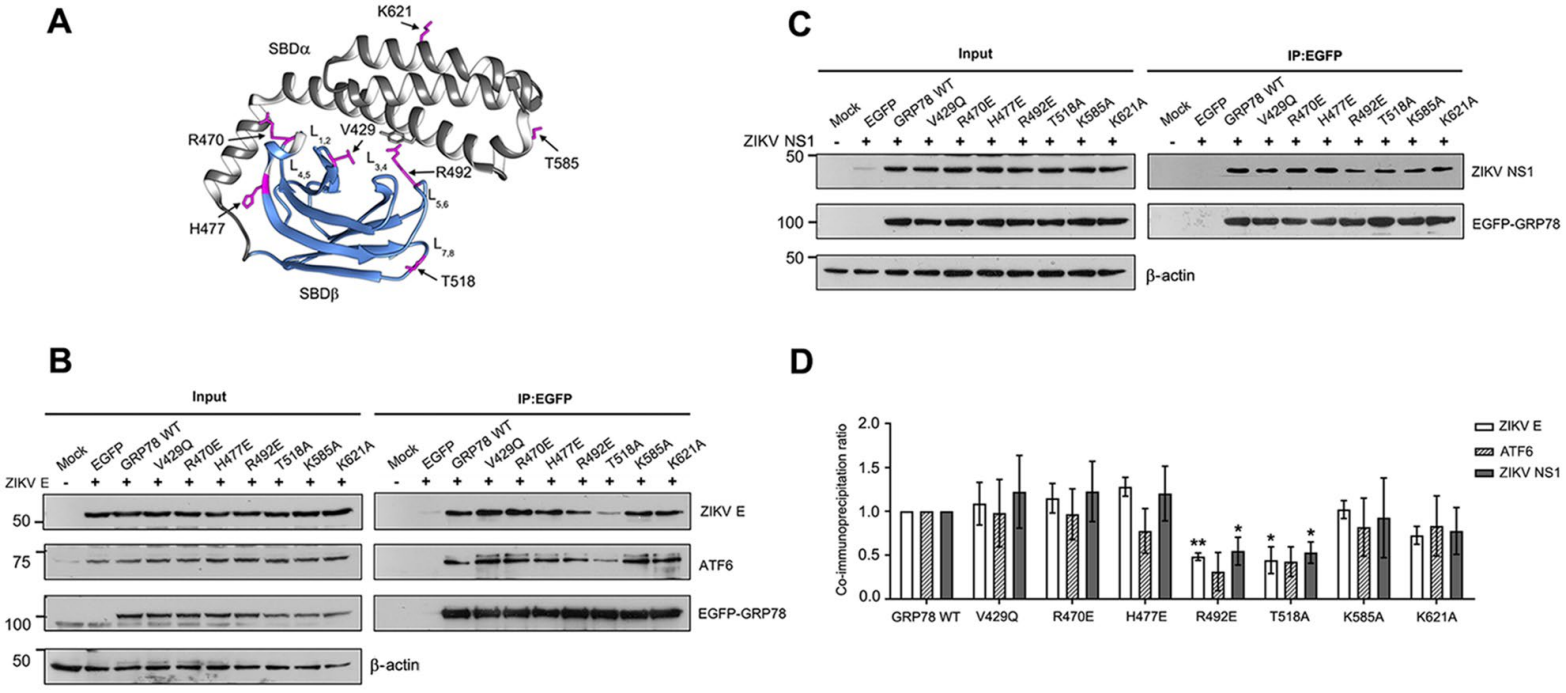
Analysis of critical GRP78 amino acid mediating the ZIKV E and NS1 protein interactions. (**A**) The structure of the ADP bound state SBD of human GRP78 (PDB ID: 5E85) and seven candidate amino acid residues in the SBD selected for characterization of their effect on ZIKV E and NS1 protein interactions are shown in magenta. (**B**) HEK293T/17 cells were co-transfected with either pEGFP-C2 (EGFP), pEGFP-full length wild type GRP78 (GRP78 WT) or mutated pEGFP-GRP78 plasmids and pcDNA3.1 + _19CprME Zika plasmid or (**C**) pcDNA3.1 + _ZIKV NS1. Mock transfected cells (Mock) were used as a negative transfection control. At 48 h post transfection, mock and transfected cells were harvested and the interaction of EGFP-GRP78 (wild type or mutant) and ZIKV proteins was determined by immunoprecipitation assay using GFP trap beads. Total protein lysate (Input) and the immunoprecipitated proteins (IP: EGFP) were examined by western blot analysis. (**D**) The band intensity of co-immunoprecipitated ZIKV E, NS1 and endogenous ATF6 was quantitated using Quantity One and normalized against the corresponding immunoprecipitated GRP78 and the GRP78 wild type ratio. Protein level is shown as a co-immunoprecipitation ratio in bar graphs. Error bars represents SEM and p-value less than 0.05 was considered as a significant difference (*p-value < 0.05, **p-value < 0.01). Panels B, and C consist of composite images. Different western blots are separated by white vertical lines, and subsequent probings are separated by white horizontal lines. Full uncropped western blot images can be found in the supplemental materials.

As both ZIKV E and NS1 proteins interact with GRP78, we next aimed to characterize the amino acid residues on GRP78 that mediate the interactions. To determine this, EGFP-full length GRP78 was used as a bait protein while ZIKV E and ZIKV NS1 proteins were used as preys. Seven single amino acid substitutions (V429Q, R470E, H477E, R492E, T518A, K585A and K621A) in both the SBDβ and SBDα domains were introduced into the SBD of the EGFP-full length GRP78 protein. These residues have been reported to directly interact with substrates or regulate the chaperone activity of GRP78. V429 and R492 are located in the substrate binding cleft, R470, H477 and T518 are located in the SBDβ while K585 and K621 are located on the SBDα helix lid (Fig. 2A). The amino acid sequence of SBD of human GRP78 was aligned with human and eukaryotic HSP70s and the result showed that R470, R492 and K585 are highly conserved in all those organisms (Supplemental Table S4 and Supplemental Fig. S3). To disrupt the protein–protein interaction, hydrophobic amino acid at V429 was substituted with a polar amino acid glutamine (Q). The positive charge amino acid at R470, H477 and R492 were substituted with the negative charge amino acid of glutamic acid. To eliminate the contribution of the side chain to protein–protein interaction, threonine and lysine amino acid at T518, K585 and K621 were replaced with alanine.

Wild type EGFP-full length GRP78 and individual mutants were co-expressed with ZIKV E or ZIKV NS1 proteins in HEK293T/17 cells. At 48 h post transfection, cells were collected and used to prepare total proteins. A total of 40 µL of total protein lysate was reserved as the input protein sample. Remaining sample was used to evaluate the protein–protein interaction by GFP bead co-immunoprecipitation assay. Both input protein samples and the immunoprecipitated protein samples (IP proteins) were analyzed by western blot and the presence of wild type and mutated EGFP-GRP78, ZIKV E, ZIKV NS1 and ATF6 protein were individually detected using specific antibodies. ATF6 is a well-characterized interacting partner of GRP78 in the UPR pathway^63^, and it was used as a positive cellular interacting control. From the results, most recombinant proteins (input proteins), wild type and mutated EGFP-GRP78, ZIKV E, ZIKV NS1 and ATF6 were expressed at a similar level when compared to the wild type EGFP-GRP78 control. However, the expression of GRP78 mutant R470E, H477E and ZIKV E protein (when co-transfected with GRP78 mutant H477E) showed a significantly reduced expression level when compared to the wild type EGFP-GRP78 control (Fig. 2B,C and Supplemental Fig. S4). To determine the level of co-immunoprecipitated protein, the protein band intensity of ZIKV E, ZIKV NS1 and ATF6 was quantitated and normalized against immunoprecipitated EGFP-full length GRP78. The level of immunoprecipitation was expressed as a co-immunoprecipitation ratio. From the results, ZIKV E, ZIKV NS1 and ATF6 were co-immunoprecipitated by V429Q, R470E, H477E, K585A and K621A at a similar level as compared to wild type EGFP-full length GRP78 (Fig. 2D). Interestingly, co-immunoprecipitated ZIKV E and ZIKV NS1 were significantly reduced in pull downs with GRP78 mutations R492E and T518A as compared to the co-immunoprecipitation with wild type EGFP-full length GRP78. Similarly, immunoprecipitation of ATF6 was also slightly reduced with these two mutations, albeit that the result did not reach statistical significance (Fig. 2D). These results demonstrated that R492 and T518 amino acids on GRP78 protein are critical for ZIKV E, ZIKV NS1 and ATF6 binding.

### GRP78 mutation increased infectious ZIKV production but not protein expression

**Figure 3:**
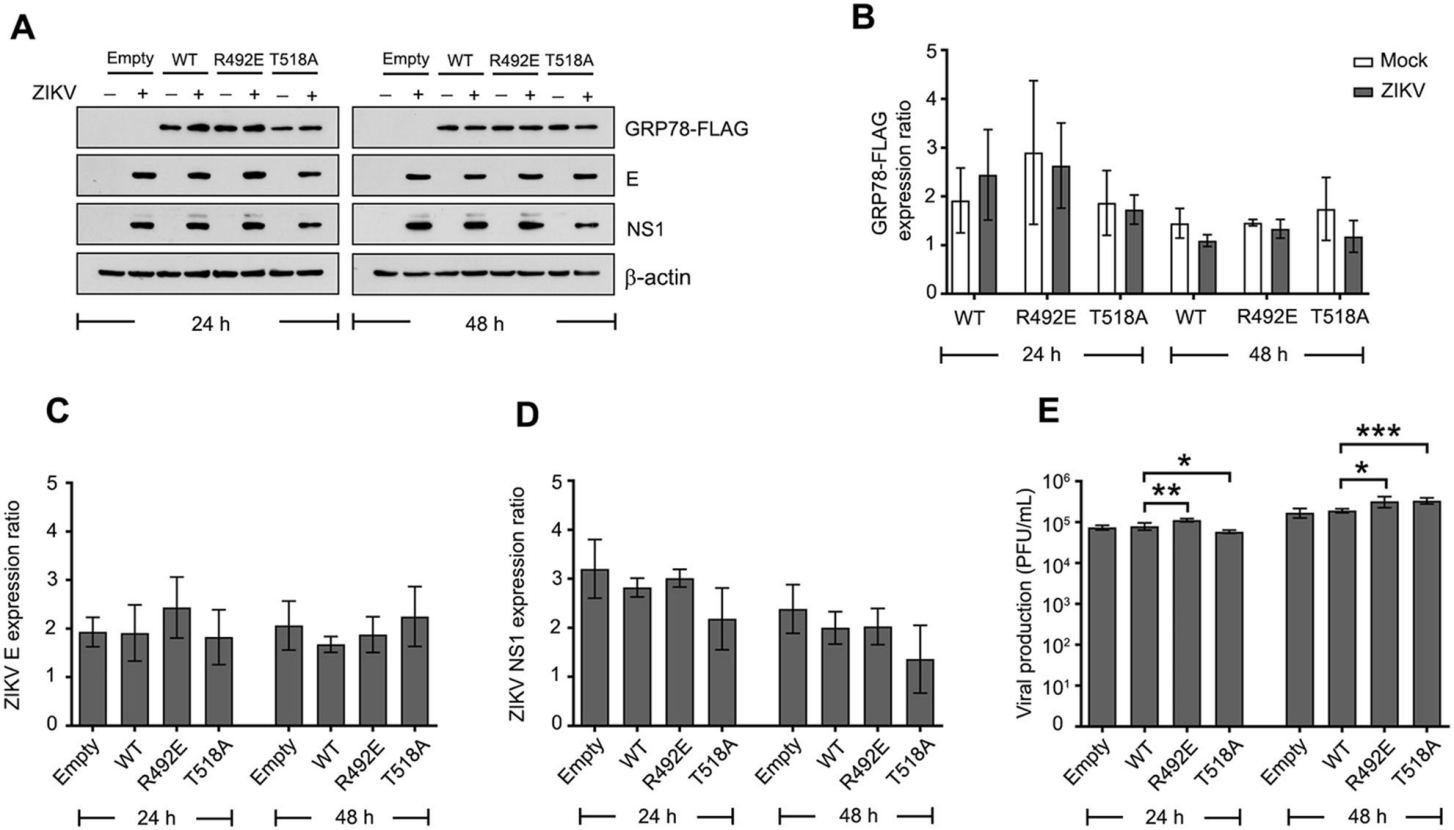
The effect of GRP78 mutation on ZIKV infection and replication. (**A**) Wild type or mutated GRP78 was stably expressed in A549 cells a using lentivirus system. An empty transduction was used as a negative control of expression. Transduced A549 cells were infected with ZIKV at MOI 1 and infected cells were harvested at 24 and 48 h. Protein expression was examined by western blot analysis. The band intensity of (**B**) FLAG tag GRP78 protein (**C**) ZIKV E and (**D**) ZIKV NS1 protein was quantitated using Quantity One and normalized against β-actin. Protein level is shown as an expression ratio in bar graphs. (**E**) The supernatant was harvested and the ZIKV production was measured by standard plaque assay. Error bars represents SD and p-value less than 0.05 was considered as a significant difference (*p-value < 0.05, **p-value < 0.01, ***p-value < 0.001). Panel A consist of a composite image. Different western blots are separated by white vertical lines, and subsequent probings are separated by white horizontal lines. Full uncropped western blot images can be found in the supplemental materials.

Glutamic acid substitution at R492 and alanine substitution at T518 on GRP78 protein significantly reduced the binding of ZIKV E and NS1 protein. The biological role of these GRP78 residues was subsequently investigated upon ZIKV infection and replication in mammalian cells. Stable cell lines derived from A549 were generated using a lentiviral vector carrying either wild type or R492E or T518A mutated GRP78 gene to stably overexpress the recombinant GRP78 protein. These recombinant proteins were tagged with FLAG and HA at the C-terminus to differentiate from the endogenous protein. Either wild type or mutated GRP78 overexpressing A549 cells were infected with ZIKV at a multiplicity of infection (MOI) of 1. After 24 and 48 h of infection, infected cells were collected and the viral protein expression was examined by western blot analysis. From the results, both wild type and mutated GRP78 protein were expressed in A549 at a similar level. When these cells were infected with ZIKV, there was no significant difference of ZIKV E and NS1 protein expression level in R492E and T518A expressed cells when compared to the wild type GRP78 expressed cells (Fig. 3A–D). The ZIKV production in the supernatant was quantitated by the standard plaque assay. At 24 h after infection, the infectious virus titer was significantly increased in R492E derived supernatant but decreased in T518A derived supernatant when compared to the wild type derived supernatant. Interestingly, the infectious ZIKV was significantly higher in both R492E and T518A derived supernatant when compared to the wild type at 48 h post infection (Fig. 3E). This result suggested that R492E and T518A mutations of GRP78 does not alter viral protein translation but may be involved in the later replication steps such as protein folding.

### GRP78 and ZIKV E interaction is maintained upon single amino acid substitutions of ZIKV E domain III

**Figure 4:**
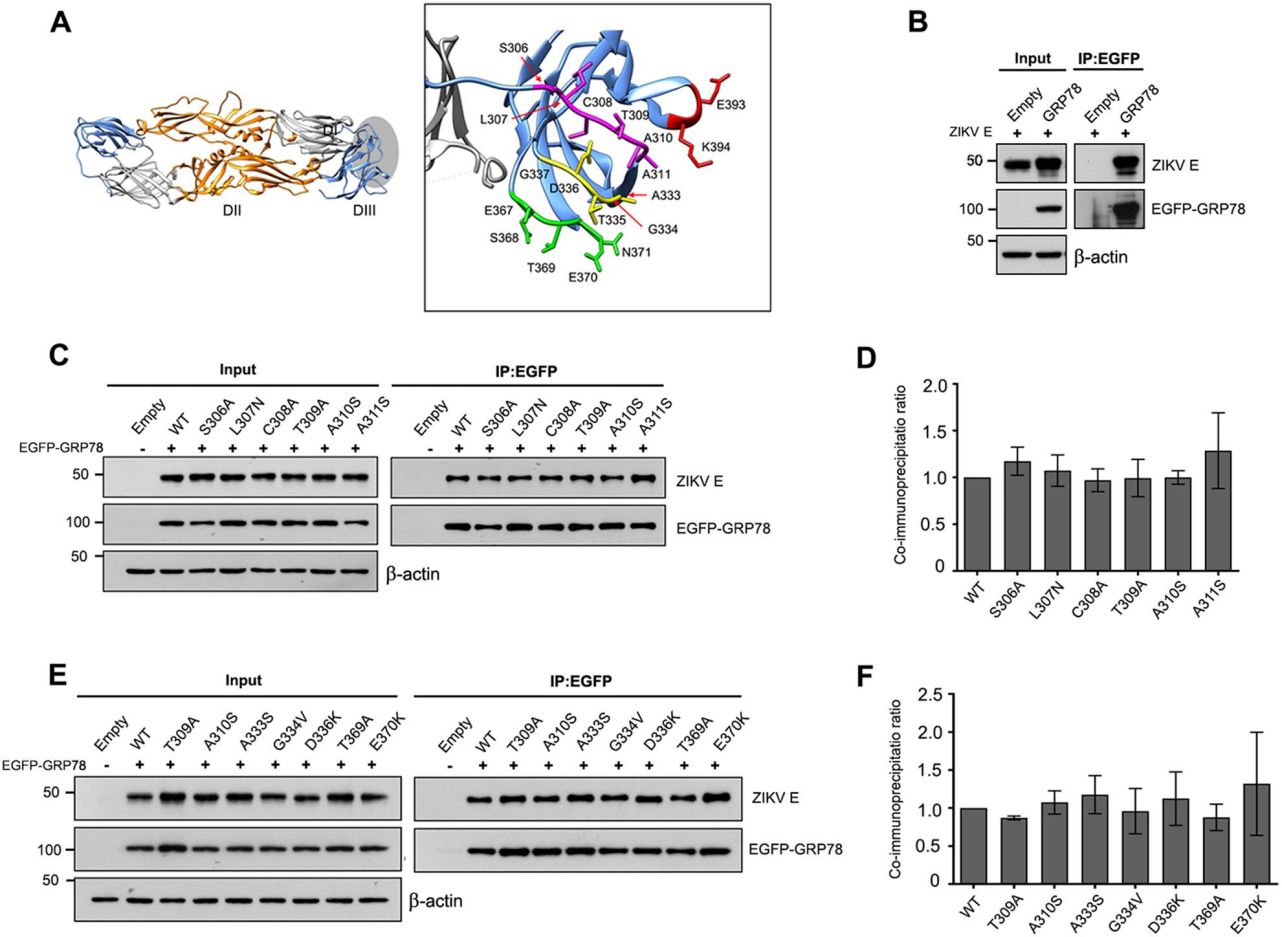
Analysis of critical ZIKV E amino acid mediating GRP78 protein interactions. (**A**) The structure of the ZIKV E dimer (PDB ID: 5jhm) which contains three distinct domains: β-barrel shaped domain I (grey), finger-like domain II (orange), and immunoglobulin-like domain III (blue). The candidate amino acid residues on ZIKV E domain III for site directed mutagenesis are shown in green, yellow, magenta and red color. (**B**) HEK293T/17 cells were transfected with either pEGFP-C2 (Empty) or pEGFP-GRP78 plasmid and co-transfected with wild type pcDNA-ZIKV E-HA plasmid. At 48 h post transfection, transfected cells were harvested and the interaction of EGFP-GRP78 and ZIKV E was determined by immunoprecipitation assay using GFP trap beads. Total protein lysate (Input) and immunoprecipitated proteins (IP: EGFP) were examined by western blot analysis. (**C**) and (**E**)) HEK293T/17 cells were transfected with either pEGFP-C2 or pEGFP-GRP78 and co-transfected with wild type (WT) or mutated pcDNA-ZIKV E-HA plasmid. The interaction of EGFP-GRP78 and wild type or mutated ZIKV E proteins was determined by immunoprecipitation assay. (**D**) and (**F**)) The band intensity of co-immunoprecipitated ZIKV E was quantitated using Quantity One and normalized against the corresponding immunoprecipitated GRP78 and wild type ZIKV E ratio. Data is shown as a co-immunoprecipitation ratio in bar graphs. Error bars represent SD and p-value less than 0.05 was considered as a significant difference. Panels B, C and E consist of composite images. Different western blots are separated by white vertical lines, and subsequent probings are separated by white horizontal lines. Full uncropped western blot images can be found in the supplemental materials.

We aimed to investigate the molecular mechanism underlying of GRP78 and ZIKV E protein interaction, by determining which amino acids on the envelope protein are critical for the binding. Our previous work has identified GRP78 as a receptor for ZIKV binding and internalization into host cells and plays important roles during virus infection and replication^50^. ZIKV E domain III (93 amino acids) is an immunoglobulin-like domain which plays a key role in receptor binding upon virus entry into host cell. Furthermore, Elfik and colleague predicted the binding of ZIKV E with GRP78 protein using the HADDOCK web server. From this docking study, hydrophobic residues C308, T309 and A310 on ZIKV E domain III were identified as interacting residues to SBD (I426, T428, V429, V432, T434, F451, S452, V457, and I459) of GRP78 protein^64^. Based on all evidence, those three predicted residues and the 15 surrounding amino acids with surface accessible position (S306:A311, A333:G337, E367:N371, E393 and K394) on ZIKV E domain III were selected to characterize their effect on GRP78 binding (Fig. 4A). To examine this, the recombinant pcDNA-ZIKV E-HA plasmid was constructed to express ZIKV E-HA tag alone without prM. The interaction of wild type ZIKV E-HA tag to EGFP-GRP78 protein was confirmed by co-immunoprecipitation assay using the same GFP trap beads and western blot analysis. From the results, wild type ZIKV E-HA protein was specifically co-immunoprecipitated with EGFP-GRP78 protein (Fig. 4B). Then, a single amino acid substitution of 18 candidate residues were individually introduced into pcDNA-ZIKV E-HA plasmid using QuikChange site directed mutagenesis. HEK293T/17 cells were transfected with pEGFP-GRP78 plasmid or pEGFP-C2 empty plasmid and co-transfected with either wild type or mutated pcDNA-ZIKV E-HA plasmid. Transfected cells were collected at 48 h and protein–protein interaction was examined by immunoprecipitation assay. The total protein lysate (Input) and all 35 μL of immunoprecipitated proteins (IP: EGFP) were examined by western blot analysis. From the screening results, EGFP-GRP78, wild type and mutated ZIKV E proteins were expressed in transfected cells at a similar level. T309A, A310S, A333S, D336K, and T369A mutated ZIKV E protein were significantly co-immunoprecipitated with EGFP-GRP78 at a lower level when compared to wild type ZIKV E protein (Supplemental Figs. S5–S7). However, high variation of co-immunoprecipitated ratio was obtained from 4 independent experiments. In addition, excess protein concentration in western blot analysis could lead to a saturated signal and problems with the relative protein quantification. To exclude this issue, the predicted interacting residues to GRP78 protein (C308, T309 and A310) and significant residues that showed a reduction on GRP78 binding was examined in an independent co-immunoprecipitation experiments. The loading volume was reduced from 35 μL to 15 μL of eluted protein for western blot analysis. EGFP-GRP78 and mutated ZIKV E protein were expressed in transfected cells at a similar level when compared to the wild type ZIKV E protein (Supplemental Fig. S8). Surprisingly, all mutated ZIKV E protein were co-immunoprecipitated with EGFP-GRP78 at a similar level when compared to the wild type ZIKV E, even T309A, A310S, A333S, D336K, and T369A mutated ZIKV E which had all shown a reduction of GRP78 binding at the screening IP (Fig. 4C–F). Taken together, the interaction between GRP78 and ZIKV E was stably maintained against single mutation of 18 amino acids on ZIKV E domain III. These results suggested that other two domains of ZIKV E maybe involved in the binding with a high dynamic range for their binding specificity.

### All subdomains of ZIKV E and NS1 proteins interact with GRP78

**Figure 5:**
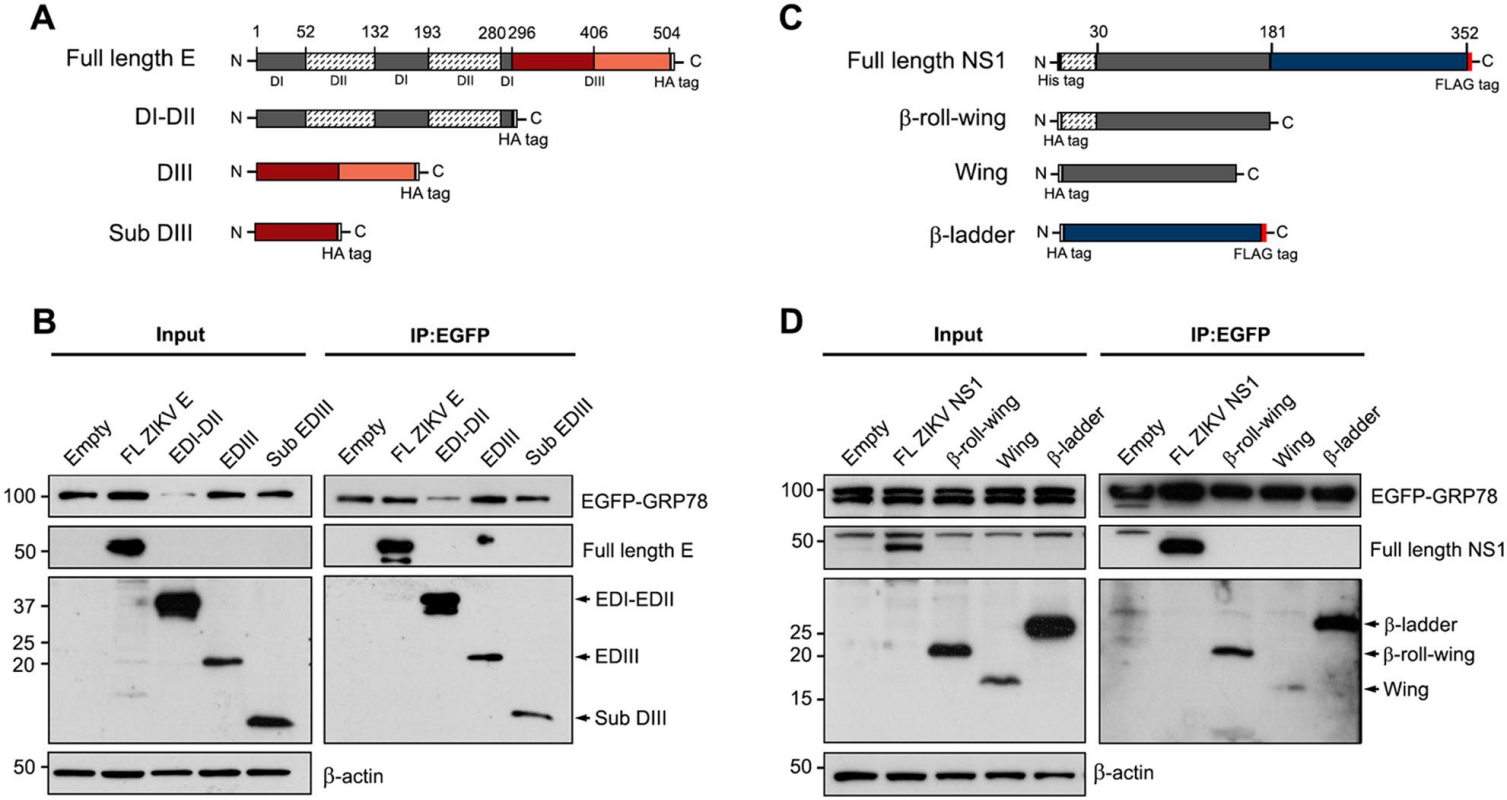
Characterization of interacting domain on ZIKV E and ZIKV NS1 mediating GRP78 protein interactions. (**A**) A schematic diagram illustrating the constructs for a full length ZIKV E, a domain I and II (DI-DII), a domain III (DIII) and a subdomain III (sub DIII) of ZIKV E. (**B**) HEK293T/17 cells were transfected with pEGFP-GRP78 (GRP78) and co-transfected with pcDNA3.1 + (Empty) or pcDNA3.1 + full length ZIKV E or a domain I and II (DI-DII), a domain III (DIII) and a sub domain III (sub DIII) of ZIKV E plasmid. (**C**) A schematic diagram illustrating the constructs for a full length ZIKV NS1 (His-ZIKV NS1-FLAG tag), a β-ladder domain, β-roll and wing domain and wing domain of ZIKV NS1 protein. (**D**) HEK293T/17 cells were transfected with pEGFP-GRP78 (GRP78) and co-transfected with pcDNA3.1 + (Empty) or pcDNA3.1 + full length ZIKV NS1 or a β-ladder domain, β-roll and wing domain or wing domain of ZIKV NS1 plasmid. At 48 h post transfection, transfected cells were harvested and the interaction of EGFP-GRP78 and viral proteins were determined by immunoprecipitation assay using GFP trap beads. Total protein lysate (Input) and the immunoprecipitated proteins (IP: EGFP) were examined by western blot analysis. Panels B and D consist of composite images. Different western blots are separated by white vertical lines, and subsequent probings are separated by white horizontal lines. Full uncropped western blot images can be found in the supplemental materials.

The amino acid substitutions on ZIKV E domain III was unable to abrogate the interaction between ZIKV E and GRP78. Importantly, the interacting domain on ZIKV E and NS1 protein to GRP78 has not been directly investigated. To understand the molecular binding mechanism, we stepped back to characterize the binding domain of ZIKV E and ZIKV NS1 protein for GRP78. Initially, recombinant plasmids were constructed to express the full length or truncated ZIKV E; with HA tag at the C-terminus (Fig. 5A) and full length or truncated ZIKV NS1 proteins; β roll-wing, wing and β-ladder domain with HA tag at the N-terminus (Fig. 5C). The interaction of full length and truncated ZIKV proteins to EGFP-GRP78 was examined by co-immunoprecipitation assay. From the results, full length ZIKV E, ZIKV NS1 and all subdomains were co-immunoprecipitated with EGFP-GRP78 protein (Fig. 5B,D). Several single amino acid changes on ZIKV E domain III and fragmented ZIKV E and NS1 proteins were unable to disrupt the binding by GRP78. These results suggested that GRP78 mainly recognizes and binds to ZIKV E and ZIKV NS1 protein through a chaperone-client protein interaction. As chaperone protein GRP78, recognizes hydrophobic domains of extended polypeptides and is able to bind at multiple sites of various target proteins.

### GRP78 inhibitor diminished ZIKV infection and replication in mammalian cells

**Figure 6:**
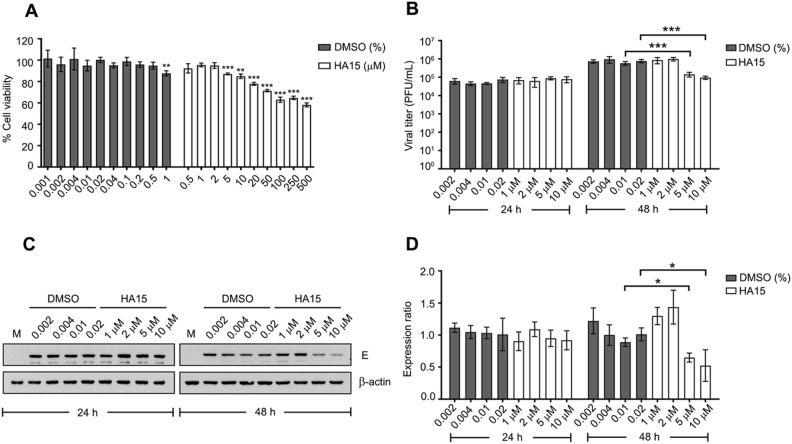
The effect of GRP78 inhibitor on ZIKV infection and replication in A549 cells. (**A**) A549 cells were treated with a various concentration of HA15 (a GRP78 inhibitor) or various concentration of the corresponding DMSO as control. At 24 h after treatment, the cell viability was measured by MTT assay. (**B**) A549 cells were treated with HA15 or corresponding DMSO for 3 h and then infected with ZIKV at MOI 0.5. The infected cells and supernatant were collected at 24 and 48 h. The viral titer was measured by plaque assay. (**C**) Viral protein expression was examined in the infected cells by western blot analysis. (**D**) The band intensity of protein was quantitated using Quantity One and normalized against β-actin. Protein level is shown as an expression ratio in bar graphs. Error bars represent SD and p-value less than 0.05 was considered as a significant difference (*p-value < 0.05 and ***p-value < 0.001). Panels C consist of a composite image. Different western blots are separated by white vertical lines, and subsequent probings are separated by white horizontal lines. Full uncropped western blot images can be found in the supplemental materials.

Our data clearly showed that the interaction between ZIKV E/NS1 and GRP78 protein was mainly through chaperone-client protein binding. ZIKV infection and replication was reduced upon GRP78 gene down regulation in A549 cells^50^. These results demonstrate that host chaperone GRP78 plays critical roles on ZIKV infection and replication in host cells. It may be a potential target for antiviral drug development. HA15 is a specific GRP78/Bip inhibitor by targeting and blocking the ATPase activity of the chaperone function with an anticancer activity^65^. However, the antiviral activity against ZIKV is unknown. For these reasons, we aimed to examine the effect of HA15 on ZIKV infection and replication in mammalian cells. The cytotoxicity of HA15 was initially investigated in A549 cells. These cells were treated with a various concentration of HA15 and the corresponding DMSO concentration as the control for 24 h. The cell viability was determined by MTT assay. From the result, up to 0.5% DMSO was not toxic to the treated A549 cells, only 1% DMSO significantly reduced cell viability when compared to control. Cell viability was significantly reduced at 5 μM of HA15 treatment when compared to the control (Fig. 6A). The 50% cytotoxic concentration (CC_50_) of HA15 treatment on A549 cells was 566.7 μM. To investigate the antiviral activity, A549 cells were pretreated with 1, 2, 5 or 10 μM of HA15 for 3 h and then subsequently infected with ZIKV at MOI 0.5. The infected cells were maintained in completed media containing HA15 or DMSO at a corresponding concentration. From the results, there was no effect of HA15 treatment to virus production at 24 h. Interestingly, infectious ZIKV production was significantly reduced in 5 and 10 μM HA15 treated cells when compared to the DMSO control at 48 h (Fig. 6B). In addition, ZIKV E protein expression was reduced in the 5 and 10 μM of HA15 treated cells when compared to DMSO control at 48 h but not at the early time point (Fig. 6C,D). However, HA15 was highly toxic to A549 cells as a result from anticancer activity and may lead to a reduction of ZIKV infection and replication in treated A549 cells. To verify this concern, primary cells such as neuronal progenitor cells (NPCs) are a suitable model for testing the antiviral activity of HA15.

**Figure 7:**
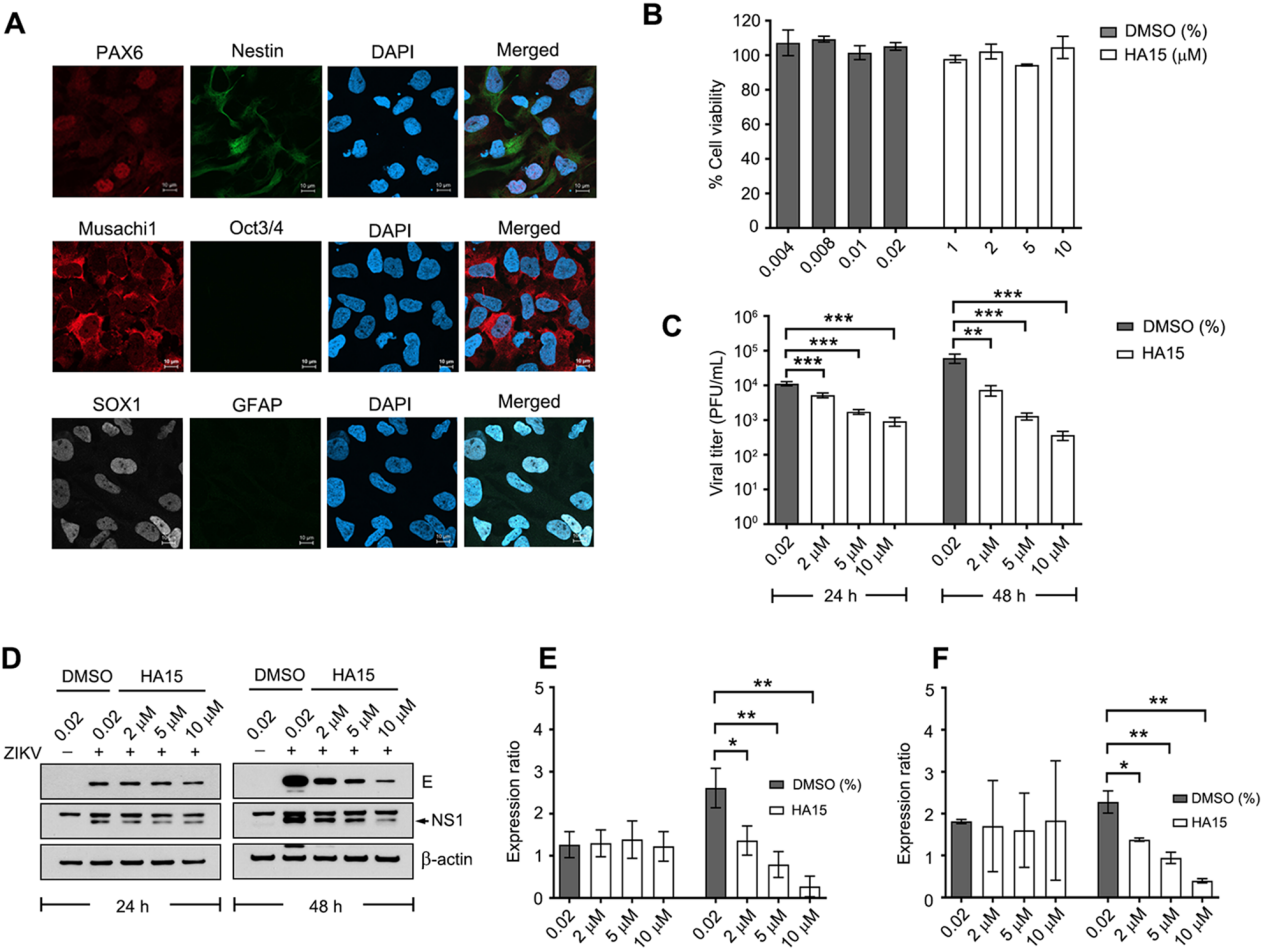
The effect of GRP78 inhibitor on ZIKV infection and replication in human neuronal progenitor cells (NPCs). (**A**) NPCs were stained against several protein markers using indirect immunofluorescence assay and observed under the confocal microscope. (**B**) NPCs were treated with a various concentration of HA15 (GRP78 inhibitor) or a various concentration of the corresponding DMSO as control. At 24 h after treatment, the cell viability was measured by MTT assay. (**C**) NPCs cells were treated with HA-15 or corresponding DMSO for 3 h and then infected with ZIKV at MOI 0.5. The infected cells and supernatant were collected at 24 and 48 h. The viral titer was measured by plaque assay. (**D**) Viral protein expression was examined in the infected cells by western blot analysis. ((**E**) and (**F**)) The band intensity of protein was quantitated using Quantity One and normalized against β-actin. Protein level is shown as an expression ratio in bar graphs. Error bars represent SD and p-value less than 0.05 was considered as a significant difference (*p-value < 0.05, **p-value < 0.01 and ***p-value < 0.001). Panels D consists of a composite image. Different western blots are separated by white vertical lines, and subsequent probings are separated by white horizontal lines. Full uncropped western blot images can be found in the supplemental materials.

Neuronal progenitor cells are the main target for ZIKV infection and contributing to the pathological damage of microcephaly. The antiviral activity of HA15 was determined against ZIKV infection of NPCs. Human induced pluripotent stem cells (hiPSCs) were induced to differentiate into NPCs and the protein markers were characterized by indirect immunofluorescence assay. From the results, PAX6, nestin, Musachi1 and SOX1 were expressed in the induced NPCs but Oct3/4 and GFAP was not present in the induced cells. These results confirmed that the induced cells were NPCs (Fig. 7A). The effect of HA15 to cell viability was investigated by MTT assay and the results showed that 2, 5 and 10 μM of HA15 treatment was not toxic to NPCs (Fig. 7B). To investigate the antiviral activity, these cells were treated with various concentrations of HA15 for 3 h and the treated cells were infected with ZIKV at MOI 0.5. After virus inoculation for 2 h, infected cells were maintained with complete media supplemented with the corresponding concentration of HA15 or DMSO. The supernatant was harvested at 24 and 48 h post infection. The virus production was determined by standard plaque assay. From results, the virus titer was significantly diminished at 24 h and 48 h post infection in a dose dependent manner when compared to the DMSO control (Fig. 7C). However, the viral protein expression level was not reduced by HA15 treatment at 24 h post infection (Fig. 7D–F). The HA15 treatment significantly reduced ZIKV E and ZIKV NS1 protein expression at 48 h post infection at all concentration when compared to the DMSO control (Fig. 7D–F). Our results revealed that GRP78 is a potential host target for antiviral drug development. The GRP78 inhibitor reduced infectious virus production from infected cells at 24 h without viral protein changes. This evidence suggests that GRP78 may play a role as a viral chaperone protein by facilitating viral protein folding.

## Discussion

GRP78 is a master regulatory protein of the UPR pathway^30^, mainly through functioning as an ER resident chaperone protein. However, GRP78 is a multifunctional protein, and expression has been shown to occur on the cell surface^66^ where it can act as a receptor for a number of ligands (reviewed in^67^). A number of flaviviruses including DENV^55^, JEV^46^, ZIKV^50^ and TMUV^56^ have been shown to be able to utilize GRP78 to enter mammalian cells, and in addition GRP78 has been shown to be required for replication of a number of flaviviruses^42–52,54^. Perhaps most importantly, specific interactions between GRP78 and flaviviral proteins have been reported for E protein for DENV^43,53^, JEV^46^ and ZIKV^49–51^, and for DENV and ZIKV NS1 protein^49,54^. In this study we confirmed the observation of Chen and colleagues^49^ that GRP78 interacts with ZIKV NS1 protein. Given that GRP78 interacts with both DENV and ZIKV NS1, it is possible that this interaction commonly occurs in flaviviral infections as has been seen for the flaviviral E protein^43,46,49–51,53^.

An efficient interaction between GRP78 and both ZIKV E and NS1 proteins required both domains of GRP78, and the interaction was largely abolished when the interaction was investigated with the isolated NBD and SBD domains. However, some interaction between ZIKV NS1 and the isolated GRP78 domains (particularly the NBD) was observed suggesting a possible novel interaction of uncertain significance. The SBD has previously been proposed computationally to be the interacting domain for ZIKV E protein^64^, and a yeast-2-hybrid analysis suggested that the DENV E protein binding domain was located in the SBD at position 467–530^43^. It was further proposed by the same group that DENV NS1 bound to both full length GRP78, and the isolated SBD^54^, an observation not supported by this study. It is unclear whether the contradictory results arise from the different viruses (DENV versus ZIKV), or methodological differences. For example, in this study both NS1 and GRP78 were transfected into cells, while in the previous study^54^ GRP78 was transfected while NS1 was expressed through infection of the transfected cells, meaning that there was a full complement of viral structural and non-structural proteins present. Conversely, this study used EGFP-tagged GRP78 proteins which could possibly alter binding or protein localization. For protein localization it was shown that the EGFP-tagged constructs (EGFP-GRP78, EGFP-NBD and EGFP-SBD) showed similar localization to endogenous GRP78, albeit that ER localization was somewhat reduced. However, GRP78 is widely expressed in the cell, and is not solely found in the ER^68^. In addition, given that co-immunoprecipitation occurs after a lysate stage, it is unclear as to how much the original cell location impacts the effect of the immunoprecipitation. In addition, under physiological conditions the ER lumen Ca^2+^ concentration can range from 100 to 800 μM, while cytosolic Ca^2+^ concentrations are several orders of magnitude lower, at approximately 100 nM^69^. GRP78/BiP itself acts as a major, high capacity Ca^2+^ binding protein to store Ca^2^ +, and GRP78/BiP is believed to be responsible for storing around 25% of the ER Ca^2+^^70^. It is also known that depletion of ER Ca^2+^ stores can abrogate the interactions between GRP78 and client proteins^71^. However, given that at least a proportion of the constructs used in this study do indeed locate to the ER lumen, it is uncertain if Ca2 + concentrations have a major effect on the results seen here.

GRP78 functions as a chaperone protein by binding to an extended polypeptide substrate through a cooperative domain motion between the NBD and SBD. The NBD contains the ATPase catalytic site while the SBD contains a substrate binding cleft and an α-helix lid covering the substrate binding pocket. As noted, the SBD has been identified as the binding site for DENV E and DENV NS1 proteins^43,54^ and is therefore likely to be the interacting domain for ZIKV E and NS1. In this study, single amino acid mutations were introduced throughout substrate binding pocket and the α-helix lid of the SBD. The candidate residues (V429Q, R470E, H477E, R492E, T518A, K585A and K621A) chosen to be investigated in this study were selected as they have previously been reported to directly interact with substrates, or to contribute to the chaperone function of GRP78. Human GRP78 V429 was proposed as a possible interacting amino acid for ZIKV E protein domain III^64^. This amino acid corresponds to V430 of hamster GRP78 on loop L_1,2_ of SBDβ which was identified as a key residue required to maintain the stability of the chaperone-substrate complex^72^. H477 and K585 residues may be involved in interdomain allosteric communication^73–76^, while K621 was proposed to be a key amino acid for dengue NS1 binding^54^. However, we found that V429Q, H477E, K585A and K621A had no effect on the ZIKV E and ZIKV NS1 interactions, casting a question as to whether these amino acids have functional roles in GRP78 binding to viral proteins.

Structural analysis of the SBD of DnaK and human GRP78 showed that R470 and R492 in SBDβ contact with SBDα, and it was proposed that these interactions maintain the stability of the SBDα lid to cover the polypeptide binding pocket^28^, and glutamic substitution at R470 and R492 has been shown to increase the dissociation of GRP78 from a peptide substrate^77^. A negative charge substitution at R470 (DnaK-R445) moderately destabilized substrate binding by disruption of the ionic contact to D552 (Dnak-D526) on the lid domain^77–80^. However, mutation of R470 had no effect on the ZIKV E and ZIKV NS1 interactions. In contrast, R492E showed an obvious defect in the interaction between GRP78 and both ZIKV E and NS1 proteins. The discrepancy between R470 and R492 is supported by the previous study of Yang and colleagues^28^ who observed a much greater peptide-binding defect with R492 as compared to R470. It is possible that the glutamic substitution at 492 may disrupt the hydrogen bond formed between R492 (DnaK-R467) and A454 (DnaK-A429), resulting in destabilizing loop 5,6 that acts as a supportive barrier of polypeptide binding pocket^77,81^. Furthermore, R492 has been proposed to be a site for a post-translation modification site termed ADP adenylation, resulting in the suppression of substrate binding activity. However, a recent study by Preissler and colleagues argues that ADP-ribosylation sites at R470 and R492 may be misidentified and are sites for AMPylation modification^77,82,83^. Glutamic substitution at R492 imitates a negative charge of post-translation modification (ADP ribosylation/AMPylation), and while change to alanine has not been directly examined in this context, this may still contribute to the viral protein binding defect of GRP78 through the destabilization of substrate binding activity.

We additionally identified T518 in the SBDβ of GRP78 as having a significant detrimental effect on both ZIKV E and NS1 protein binding. This is in contrast to a previous study that showed a GRP78 T518C mutant had no effect on the substrate binding kinetics of GRP78^84^. However, the two studies have technical differences including the nature of the assay (in vitro assay^84^ versus in vivo pull downs (this study)), as well as in the nature of the mutation introduced (T to C and T to A).

T518 is defined as an essential residue for post-translation modification in which protein adenylyltransferase FICD (also known as Huntington yeast partner E (HYPE)) mediates the attachment of an AMP molecule to GRP78 at T518 (AMPylation) which promotes the dissociation of substrate from hamster GRP78 and reduces J-protein stimulated ATP hydrolysis^82^. Glutamic substitution at T518 residue was examined and showed an effect of mimicking AMPylation. However, the effect of alanine substitution at this position has not been directly evaluated for an effect on chaperone-substrate binding activity. T518 is located on loop L_7,8_, and its side chain contacts with the side chain of D515/D535 and the backbone amine group of N520/K540, and thus alanine substitution may disrupt these interactions and affect allosteric coupling or the binding affinity for viral proteins.

R492E and T518A substitution on GRP78 significantly reduced ZIKV E and NS1 binding but the overexpression of these mutated GRP78 in A549 cells significantly increased virus production at the late time point. There was no inhibition effect on ZIKV replication in host cells. The mechanism underlying this contradictory result remains unknown. The mutations may change the substrate binding affinity and accelerate the binding kinetic. Alternatively, ZIKV may take advantage from other chaperones or a high abundant endogenous GRP78 presence in infected cells to cope with this binding defect. Moreover, the chaperone activity relies on other co-chaperone factors such as proteins that contain J-domains or HPD motifs, ERdj proteins and nucleotide exchange factors (NEFs) to regulate the binding and release of substrates^85^. These cofactors add more layers to the complexity of chaperone action.

ATF6 is an ER bound transcription factor associated with GRP78 through the luminal domain during the resting state^86^. Shen and colleague showed that GRP78 stably binds to ATF6^87^, and that the dissociation of GRP78 upon ER stress induces intramembrane proteolysis of ATF6 into an active transcription factor that activates the expression of chaperones and UPR mediators^63,87^. The binding is mediated by the peptide-binding pocket in the same manner as unfolded protein substrate binding^88^. In our study, the R492E and T518A mutants had a partial defect in ATF6 binding, although this did not reach statistical significance. Importantly, chaperone proteins (heat shock protein; Hsp) such as GRP78 generally bind to hydrophobic domain of extended polypeptide to promote the folding and prevent aggregation^89^. They bind to the substrate at multiple distinct sites^90,91^. In the same way, GRP78 maintains the binding capacity to all truncated ZIKV E and NS1 proteins. Single amino acid substitutions were unable to disrupt this interaction. These evidences suggest that the principal interaction of GRP78 and ZIKV proteins is mediated in a canonical chaperone-client fashion.

We note that our study primarily uses co-immunoprecipitation to determine the interaction between GRP78 and ZIKV E and NS1 proteins. While this is a classic, robust technique to determine protein–protein interactions, it does have some limitations, such as an inability to detect weak or transient protein interactions. Future studies could possibly employ other techniques such as surface plasmon resonance^92^ or biolayer interferometry assays^93^ to probe the interaction between GRP78 and ZIKV E or NS1 proteins. Ideally, these techniques would provide a more quantitative analysis of the interaction. However, semi-quantitation of co-IP results as undertaken here is commonly undertaken (e.g. Refs.^94–96^).

ZIKV infection in women during pregnancy causes microcephaly and neurological abnormalities in foetus and babies termed as congenital zika syndrome^8^. The infection of virus in NPCs attenuated the cell growth through the induction of cell apoptosis and the dysregulation of the cell cycle^91,97^. Our study showed that HA15 effectively inhibited ZIKV infection and replication in neuronal progenitor cells. HA15 is a lead compound in thiazole benzensulfonamides, exhibits an anticancer activity against melanoma, lung carcinoma and myeloid leukaemia cells. This compound specifically inhibits GRP78/Bip ATPase activity resulting in the induction of ER stress and leading to cell death through autophagy and apoptosis pathway^65,98,99^. GRP78 plays important roles during infection and replication of many viruses in host cells. It was identified as an auxiliary factor for SAR-CoV2 entry and infection into host cells^100^. GRP78 protein was upregulated upon SAR-CoV2 infection and HA15 treatment clearly showed the antiviral activity^101^. In addition, GRP78 was identified as an interacting protein to E2 protein of Venezuelan equine encephalitis virus (VEEV). HA15 treatment significantly reduced the infectious virus titer of Venezuelan equine encephalitis virus (VEEV), eastern equine encephalitis virus (EEEV), chikungunya virus (CHIKV) and Sindbis virus (SINV)^102^. RNA viruses generally possess high mutation rate and trend for drug resistance against compounds that direct to the viral proteins. Thus, critical host proteins that are required for virus infection and replication can be an alternative target. These evidences demonstrated that HA15 an GRP78 inhibitor, is a broad-spectrum antiviral compound.

In summary, R492 and T518 in the SBD of GRP78 are characterized as critical residues for ZIKV E and NS1 protein binding. They may directly interact with ZIKV proteins or may be important contributing residues in the allosteric transition required for GRP78 function. The binding of GRP78 to ZIKV proteins is mainly via a chaperone-client protein pathway, and given the interaction between GRP78 and multiple domains of ZIKV E protein, the interaction is clearly not a “lock and key” interaction governed by GRP78s immunoglobulin binding properties and the immunoglobulin-like domain on ZIKV E protein. Given the broad involvement of GRP78 in flaviviral infection (Table 1), with interactions occurring at multiple stages during the replication cycle and with multiple proteins. This finding has put a spotlight on GRP78 protein as a potential therapeutic target to inhibit ZIKV infection.

### Supplementary Information

Supplementary Information.

## Data Availability

All data generated or analyzed during this study are included in this published article (and its Supplementary Information file).
