# Supplementary material for: The interaction of GRP78 and Zika virus E and NS1 proteins occurs in a chaperone-client manner
Source: Sci Rep. 2024 May 6;14:10407. doi: 10.1038/s41598-024-61195-z (PMC11074156; doi:10.1038/s41598-024-61195-z)
Supplement: Supplementary file 1 — Supplementary Information. [file 41598_2024_61195_MOESM1_ESM.pdf]

Supplemental materials

The interaction of GRP78 and Zika virus E and NS1 proteins occurs in a chaperone-client manner

Wannapa Sornjai[1], Ploenphit Promma[1], Suphansa Priewkhiew[1], Suwipa Ramphan[1], Janejira Jaratsittisin[1], Pailin Jinagool[1], Nitwara Wikan[1,2], David Murphy[3], Duncan R. Smith[1]*

[1]Molecular Pathology Laboratory, Institute of Molecular Biosciences, Mahidol University, 25/25 Phutthamonthon Sai 4 road, Salaya, Nakhon Pathom 73170, Thailand

[2]Department of Pharmacology, Faculty of Medicine, Chiang Mai University, Chiang Mai, 50200, Thailand;

[3]Molecular Neuroendocrinology Research Group, Bristol Medical School: Translational Health Sciences, University of Bristol, Bristol, United Kingdom

[*]Correspondence to: Duncan R. Smith, Molecular Pathology Laboratory, Institute of Molecular Biosciences, Mahidol University, 25/25 Phutthamonthon Sai 4 road, Salaya, Nakhon Pathom, Thailand 73170; Phone: 66(0) 2441-9003 to 7, Fax: 66(0) 2441-1013, Email: duncan_r_smith@hotmail.com, duncan.smi@mahidol.ac.th

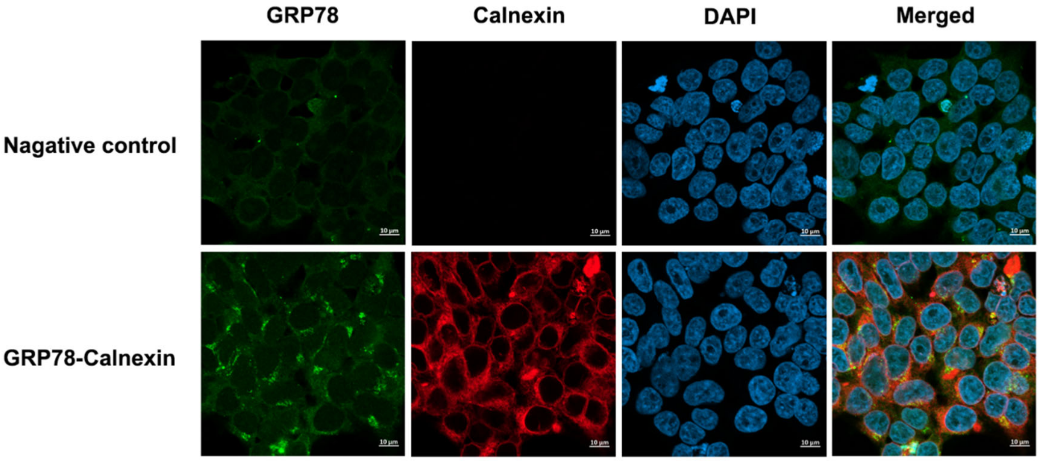

Supplemental Figure S1. The expression of endogenous GRP78 in HEK293T/17 cells

The expression of endogenous GRP78 protein and calnexin (ER marker) in HEK293T/17 cells were detected by indirect immunofluorescence assay. Stained cells were observed under a confocal microscope. Green color represents GRP78 protein and red color represents calnexin expression. The nucleus was stained with DAPI. The colocalization of GRP78 and calnexin is shown as yellow color.

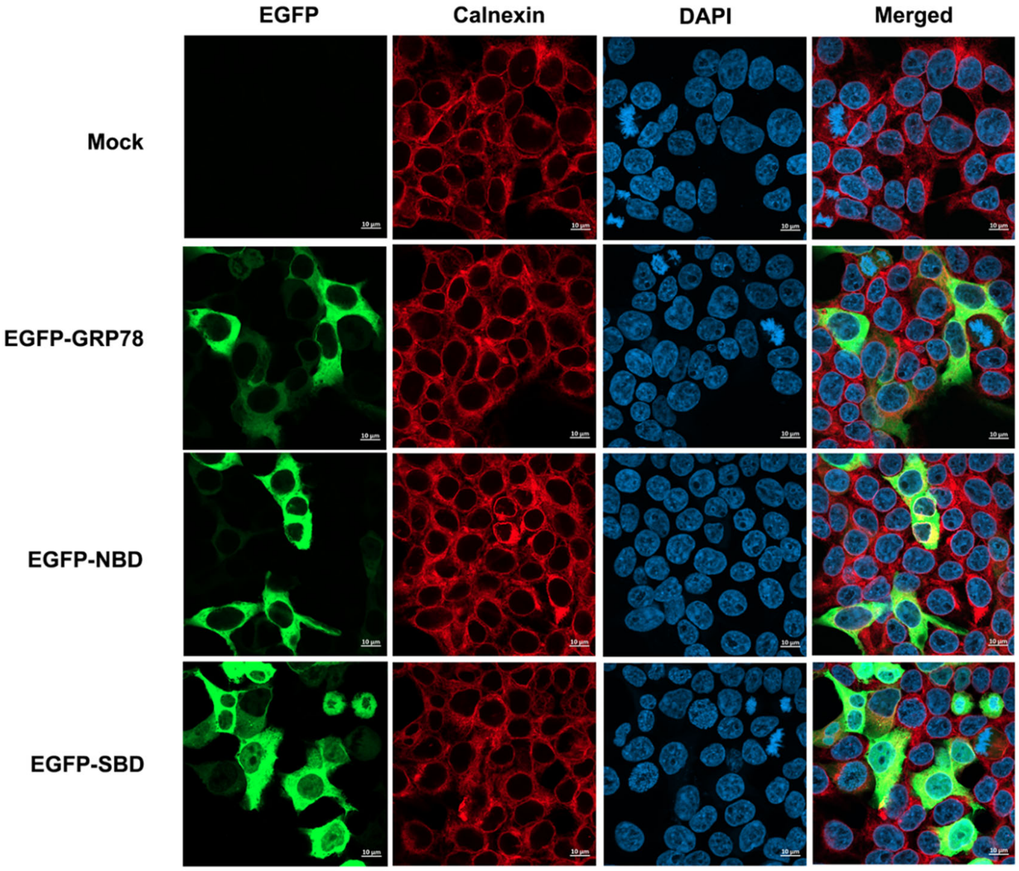

Supplemental Figure S2. The expression of EGFP-GRP78 and subdomains in HEK293T/17 cells

HEK293T/17 cells were either mock transfected or transfected with a pEGFP-full length GRP78 (EGFP-GRP78), pEGFP-NBD GRP78 or pEGFP-SBD GRP78 (EGFP-SBD) plasmid. At 24 h post transfection, the expression of recombinant proteins and calnexin an (ER protein marker) were detected by indirect immunofluorescence assay. Stained cells were observed under a confocal microscope. Green color represents EGFP fusion proteins and red color represents calnexin expression. Nucleus was stained with DAPI. The colocalization of EGFP fusion proteins and calnexin is shown as yellow.

```
                                       V429
                             |         |         |         |         |         |
Human GRP78    411    ----------CPLTLGIETVGGVMTKLIPRNTVVPTKKSQIFSTASDNQPTVTIKVYEGE    469
Hamster GRP78 411    -TGDLVLLDVCPLTLGIETVGGVMTKLIPRNTVVPTKKSQIFSTASDNQPTVTIKVYEGE    469
DnaK          386    -VKDVLLLDVTPLSLGIETMGGVMTTLIAKNTTIPTKHSQVFSTAEDNQSAVTIHVLQGE    444
Ssa1          411    KTQDLLLLDVAPLSLGIETAGGVMTKLIPRNSTIPTKKSEIFSTYADNQPGVLIQVFEGE    443
Human HSC70   384    NVQDLLLLDVTPLSLGIETAGGVMTVLIKRNTTIPTKQTQTFTTYSDNQPGVLIQVYEGE    446
Mouse HSC70   387    NVQDLLLLDVTPLSLGIETAGGVMTVLIKRNTTIPTKQTQTFTTYSDNQPGVLIQVYEGE    446
Human HSP70   387    NVQDLLLLDVAPLSLGLETAGGVMTALIKRNSTIPTKQTQIFTTYSDNQPGVLIQVYEGE    446
Mouse HSP70   387    NVQDLLLLDVAPLSLGLETAGGVMTALIKRNSTIPTKQTQTFTTYSDNQPGVLIQVYEGE    446
                      . *::**** **:**:** ***** ** :*:.:***::: *:*  ***  * *:* :**

                     R470   H477           R492                       T518
                             |         |         |         |         |         |
Human GRP78    470    RPLTKDNHLLGTFDLTGIPPAPRGVPQIEVTFEIDVNGILRVTAEDKGTGNKNKITITND    529
Hamster GRP78 470    RPLTKDNHLLGTFDLTGIPPAPRGVPQIEVTFEIDVNGILRVTAEDKGTGNKNKITITND    529
DnaK          445    RKRAADNKSLGQFNLDGINPAPRGMPQIEVTFDIDADGILHVSAKDKNSGKEQKITIKAS    504
Ssa1          444    RAKTKDNNLLGKFELSGIPPAPRGVPQIEVTFDVDSNGILNVSAVEKGTGKSNKITITND    503
Human HSC70   447    RAMTKDNNLLGKFELTGIPPAPRGVPQIEVTFDIDANGILNVSAVDKSTGKENKITITND    506
Mouse HSC70   447    RAMTKDNNLLGKFELTGIPPAPRGVPQIEVTFDIDANGILNVSAVDKSTGKENKITITND    506
Human HSP70   447    RAMTKDNNLLGRFELSGIPPAPRGVPQIEVTFDIDANGILNVTATDKSTGKANKITITND    506
Mouse HSP70   447    RAMTRDNNLLGRFELSGIPPAPRGVPQIEVTFDIDANGILNVTATDKSTGKANKITITND    506
                     *  : **: ** *:* ** *****:*******::* :***.*:* :*.:*: :****. .

                                                                             K585
                             |         |         |         |         |         |
Human GRP78    530    QNRLTPEEIERMVNDAEKFAEEDKKLKERIDTRNELESYAYSLKNQIGDKEKLGGKLSSE    589
Hamster GRP78 530    QNRLTPEEIERMVNDAEKFAEEDKKLKERIDTRNELESYAYSLKNQIGDKEKLGGKLSSE    589
DnaK          505    SG-LNEDEIQKMVRDAEANAEADRKFEELVQTRNQGDHLLHSTRKQVEE---AGDKLPAD    560
Ssa1          504    KGRLSKEDIEKMVAEAEKFKEEDEKESQRIASKNQLESIAYSLKNTISE-AG--DKLEQA    560
Human HSC70   507    KGRLSKEDIERMVQEAEKYKAEDEKQRDKVSSKNSLESYAFNMKATVED-EKLQGKINDE    565
Mouse HSC70   507    KGRLSKEDIERMVQEAEKYKAEDEKQRDKVSSKNSLESYAFNMKATVED-EKLQGKINDE    565
Human HSP70   507    KGRLSKEEIERMVQEAEKYKAEDEVQRERVSAKNALESYAFNMKSAVED-EGLKGKISEA    565
Mouse HSP70   507    KGRLSKEEIERMVQEAERYKAEDEVQRDRVAAKNALESYAFNMKSAVED-EGLKGKLSEA    565
                     .. *. ::*::** :**      *.    : : ::*  :    .. :  : :      .*:

                                                    K621
                             |         |         |         |         |         |
Human GRP78    590    DKETMEKAVEEKIEWLESHQDADIEDFKAKKKELEEIVQPIISKLYGSAGPPPTGEED--    647
Hamster GRP78 590    DKETMEKAVEEKIEWLESHQDADIEDFKAKKKELEEIVQPIISKLYGSAGPPPTGEED--    647
DnaK          561    DKTAIESALTALETALK---GEDKAAIEAKMQELAQVSQKLMEIAQQQHAQQQTAGAD--    615
Ssa1          561    DKDTVTKKAEETISWLDSNTTASKEEFDDKLKELQDIANPIMSKLYQAGGAPGGAAGGAP    620
Human HSC70   566    DKQKILDKCNEIINWLDKNQTAEKEEFEHQQKELEKVCNPIITKLYQSAGGM---PGGMP    622
Mouse HSC70   566    DKQKILDKCNEIISWLDKNQTAEKEEFEHQQKELEKVCNPIITKLYQSAGGM---PGGMP    622
Human HSP70   566    DKKKVLDKCQEVISWLDANTLAEKDEFEHKRKELEQVCNPIISGLYQGAGGP---G---P    619
Mouse HSP70   566    DKKKVLDKCQEVISWLDSNTLADKEEFVHKREELERVCSPIISGLYQGAGAP---G---A    619
                     **  : .         *.     .   :  : :**  : . ::        .

                             |         |
Human GRP78    648    -----TA-----------EKDEL----    654
Hamster GRP78 648    -----TS-----------EKDEL----    654
DnaK          616    ----ASANNAKDDDVVDAEFEEVKDKK    638
Ssa1          621    GGFPGGAPPA--PEAEGPTVEEVD---    642
Human HSC70   623    GGFPGGGAPPSGGASSGPTIEEVD---    646
Mouse HSC70   623    GGFPGGGAPPSGGASSGPTIEEVD---    646
Human HSP70   620    GGFG--AQGPKGGSGSGPTIEEVD---    641
Mouse HSP70   620    GGFG--AQAPKGASGSGPTIEEVD---    641
                          .               :*:
```

Supplemental Figure S3. The alignment was built using amino acid sequences of human GRP78, hamster GRP78, Dnak of *E. coli*, Ssa1 of *S. cerevisiae*, human HSC70, mouse HSC70, human HSP70 and mouse HSP70. The alignment was undertaken by Clustal Omega. The mutated amino acids on human GRP78 in this study and the corresponding amino acid in other proteins are labeled in gray.

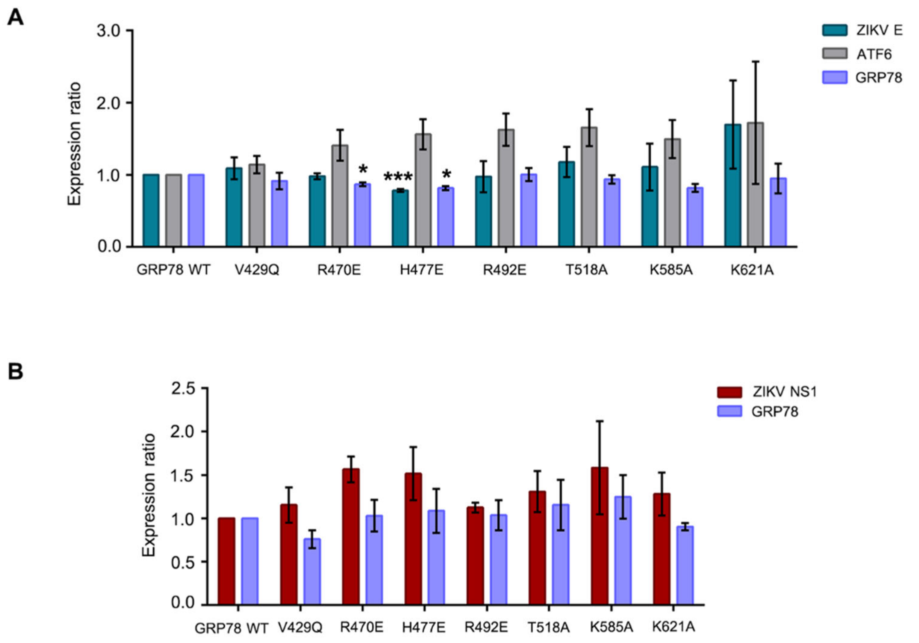

Supplemental Figure S4. The expression level of recombinant proteins from the protein-protein interaction assays

HEK293T/17 cells were transfected with either pEGFP-C2 (EGFP), pEGFP-full length wild type GRP78 (GRP78 WT) or mutated pEGFP-GRP78 plasmid and co-transfected with either (A) pcDNA3.1+_19CprME Zika plasmid or (B) pcDNA3.1+_ZIKV NS1. Mock transfected cells (Mock) were used as a negative transfection control. At 48 h post transfection, mock and transfected cells were harvested and protein lysate (Input) were examined by western blot analysis. The expression level of wild type and mutant GRP78, ZIKV E, ATF6 and ZIKV NS1 was quantitated using Quantity One and normalized against actin and GRP78 wild type control. Protein level is shown as an expression ratio in bar graphs. Error bars represents SEM and p-value less than 0.05 was considered as a significant difference (*; p-value < 0.05, ***; p-value < 0.001)

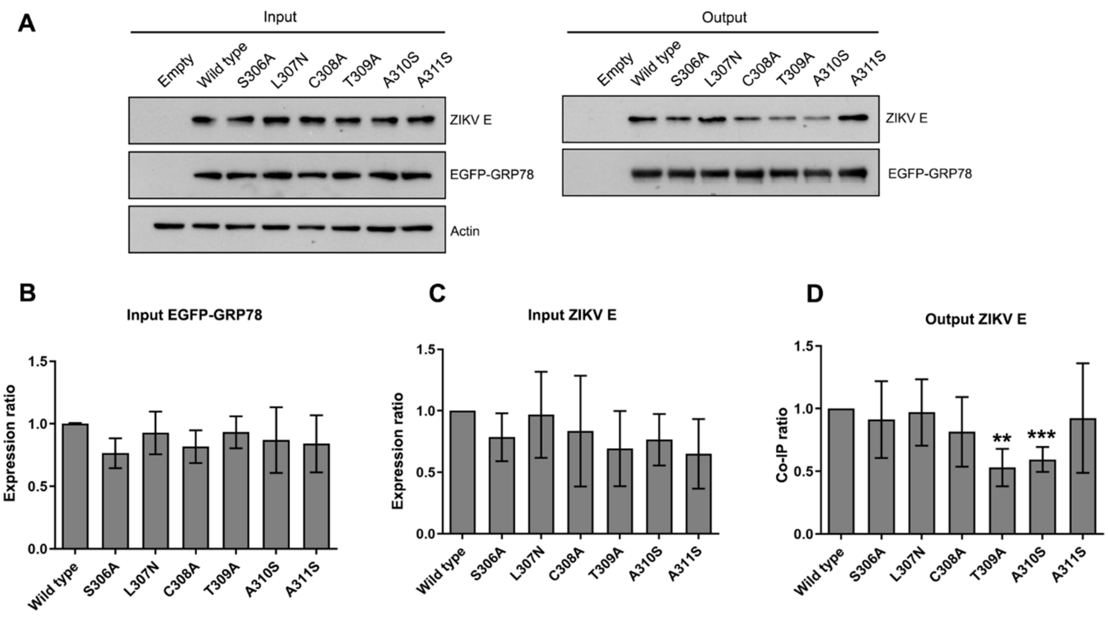

Supplemental Figure S5. The effect of single amino acid substitutions on ZIKV E domain III to GRP78 interaction

HEK293T/17 cells were transfected with either pEGFP-C2 empty or pEGFP-GRP78 plasmid and co-transfected with either pcDNA3.1 empty (Empty) or wild type pcDNA ZIKV E-HA or mutated pcDNA ZIKV E-HA (S306A, L307N, C308A, T309A, A310S and A311S) plasmid for 48 h. The interaction of EGFP-GRP78 and wild type ZIKV E or mutated ZIKV E proteins was determined by immunoprecipitation assay in 4 independent experiments. (A) Total protein lysate (Input) and the immunoprecipitated proteins (Output) were examined by western blot analysis. The protein band intensity of EGFP-GRP78, ZIKV E and actin was quantitated using Quantity One program. (B and C) Protein expression level of EGFP-GRP78 and ZIKV E was normalized against actin and the wild type ZIKV E control. (D) Co-immunoprecipitated ZIKV E was quantitated and normalized against the corresponding immunoprecipitated EGFP-GRP78 and the wild type ZIKV E ratio. Data is shown as a co-immunoprecipitation ratio in bar graphs and the error bars represent SD and p-value less than 0.05 was considered as a significant difference (**; p-value < 0.01 and ***; p-value < 0.001).

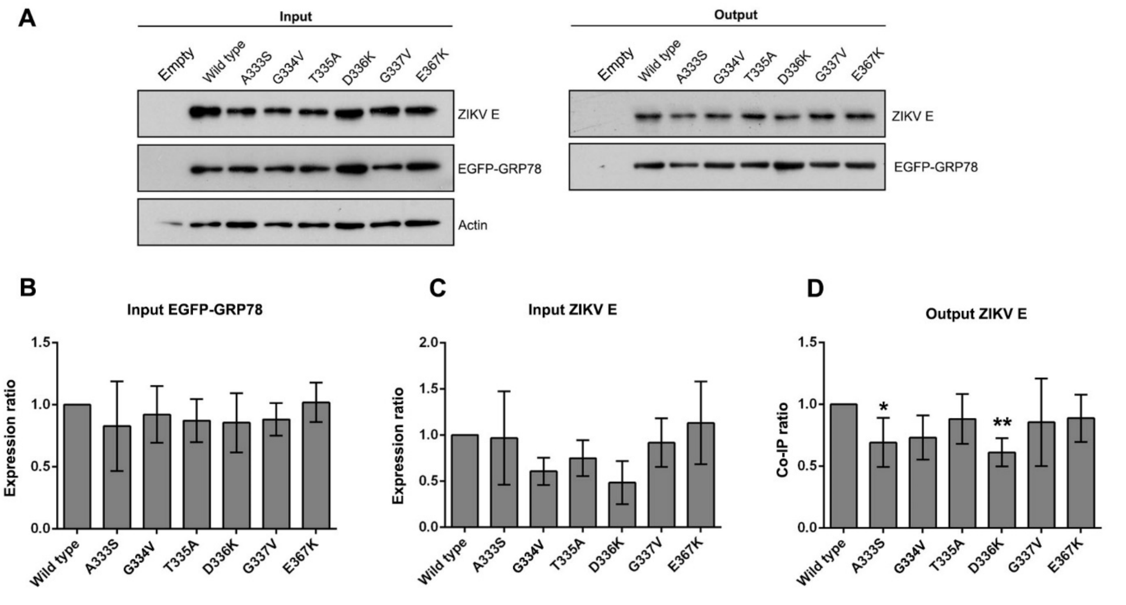

Supplemental Figure S6. The effect of single amino acid substitutions on ZIKV E domain III to GRP78 interaction

HEK293T/17 cells were transfected with either pEGFP-C2 empty or pEGFP-GRP78 plasmid and co-transfected with either pcDNA3.1 empty (Empty) or wild type pcDNA ZIKV E-HA or mutated pcDNA ZIKV E-HA (A333S, G334V, T335A, D336K, G337V and E367K) plasmid for 48 h. The interaction of EGFP-GRP78 and wild type ZIKV E or mutated ZIKV E proteins was determined by immunoprecipitation assay in 4 independent experiments. (A) Total protein lysate (Input) and the immunoprecipitated proteins (Output) were examined by western blot analysis. The protein band intensity of EGFP-GRP78, ZIKV E and actin was quantitated using Quantity One program. (B and C) Protein expression level of EGFP-GRP78 and ZIKV E was normalized against actin and the wild type ZIKV E control. (D) Co-immunoprecipitated ZIKV E was quantitated and normalized against the corresponding immunoprecipitated EGFP-GRP78 and the wild type ZIKV E ratio. Data is shown as a co-immunoprecipitation ratio in bar graphs and the error bars represent SD and p-value less than 0.05 was considered as a significant difference (*; p-value < 0.05 and **; p-value < 0.01).

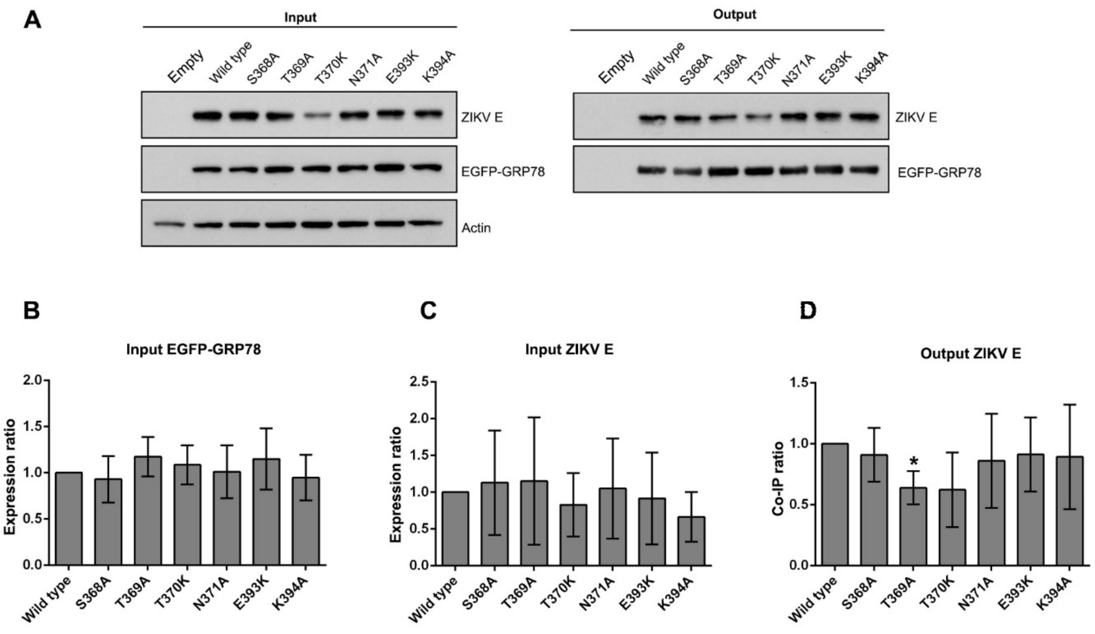

Supplemental Figure S7. The effect of single amino acid substitutions on ZIKV E domain III to GRP78 interaction

HEK293T/17 cells were transfected with either pEGFP-C2 empty or pEGFP-GRP78 plasmid and co-transfected with either pcDNA3.1 empty (Empty) or wild type pcDNA ZIKV E-HA or mutated pcDNA ZIKV E-HA (S368A, T369A, T370K, N371A, E393K and K394A) plasmid for 48 h. The interaction of EGFP-GRP78 and wild type ZIKV E or mutated ZIKV E proteins was determined by immunoprecipitation assay in 4 independent experiments. (A) Total protein lysate (Input) and the immunoprecipitated proteins (Output) were examined by western blot analysis. The protein band intensity of EGFP-GRP78, ZIKV E and actin was quantitated using Quantity One program. (B and C) Protein expression level of EGFP-GRP78 and ZIKV E was normalized against actin and the wild type ZIKV E control. (D) Co-immunoprecipitated ZIKV E was quantitated and normalized against the corresponding immunoprecipitated EGFP-GRP78 and the wild type ZIKV E ratio. Data is shown as a co-immunoprecipitation ratio in bar graphs and the error bars represent SD and p-value less than 0.05 was considered as a significant difference (*; p-value < 0.05).

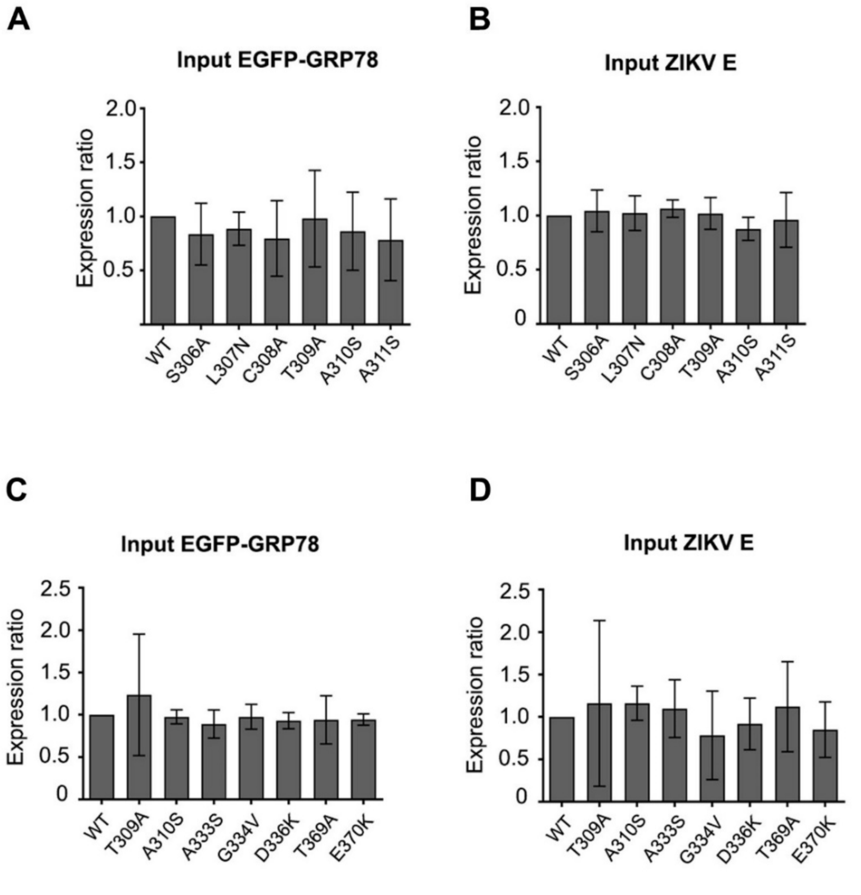

Supplemental Figure S8. The expression level of EGFP-GRP78 and ZIKV E protein in transfected HEK293T/17 cells

HEK293T/17 cells were transfected with either pEGFP-C2 empty or pEGFP-GRP78 and con-transfected with either wild type pcDNA ZIKV E-HA (WT) or mutated pcDNA ZIKV E-HA plasmid for 48 h. The interaction of EGFP-GRP78 and wild type ZIKV E or mutated ZIKV E proteins was determined by immunoprecipitation assay. Total protein lysate (Input) were examined by western blot analysis. The band intensity of EGFP-GRP78, ZIKV E and actin was quantitated using Quantity One. The expression of EGFP-GRP78 and ZIKV E was normalized against actin and wild type ZIKV E ratio and is shown as an expression ratio in bar graphs. Error bars represent SD and p-value less than 0.05 was considered as a significant difference.

Supplemental Table S1. Primers for PCR amplification

Inserted fragment	Primer name	Primer sequence (5′-3′)
Full length GRP78	HindIII_GRP78-NBD-F	ATAAGCTTAAAGCTCTCCCTGGTGGC
	BamHI_GRP78-SBD-R	ATGGATCCCTACAACTCATCTTTTTCTGC
Nucleotide binding domain-GRP78	HindIII_GRP78-NBD-F	ATAAGCTTAAAGCTCTCCCTGGTGGC
	BamHI_GRP78-ITD-R	ATGGATCCCTATACATCAAGCAGTACCAGG
Substrate binding domain-GRP78	HindIII_GRP78-SBD-F	ATAAGCTTATGTCCCCTTACACTTGG
	BamHI_GRP78-SBD-R	ATGGATCCCTACAACTCATCTTTTTCTGC
Full length ZIKV E	ZIKV_E (19prME)-NheI-F	CTAGCTAGCGCCACCATGATCCGCTGCAT TGGGGTGTCT
	ZIKV_E(19prME) -HA-BamHI-R	GCGGATCCTTAAGCGTAATCTGGAACATC GTATGGGTAACCACCTGCGGACACTGCG GTGGACAG
ZIKV E domain I and II-HA tag	ZV_EDI+II (19prME) -HA-BamHI-R	GCGGATCCTTAAGCGTAATCTGGAACATC GTATGGGTAACCACCATCCATCTTCAGCC GGCATT
ZIKV E domain III-HA tag	ZV_EDIII (19prME)-NheI-F	CTAGCTAGCGCCACCATGAAACTGAGACTG AAGGGAGTGAGC
	ZV_EDIII (19prME) -HA-BamHI-R	GCGGATCCTTAAGCGTAATCTGGAACATCG TATGGGTAACCACCTGCGGACACTGCGGTG GACA
ZIKV E sub domain III-HA tag	ZV_EDIII (297-406)-HA-BamHI-R	GCGGATCCTTAAGCGTAATCTGGAACATC GTATGGGTAACCACCGGTACTGCCGCTCC GGTG
Full length ZIKV NS1	ZNS1pCF	CGCGGATCCGCGATGCATCATCATCATCA-TCACGGTGGAGATGTGGGGTGCTCGGT
	ZNS1pCR	TCAATGGTGACTGCAGGTGGTGACTACAA-AGACGATGACGACAAGTAACCGCTCGAGCGG

ZIKV NS1 β-roll and wing domain	pc-NheI-HA-ZIKV_NS1-F	TTGCTAGCGCCACCATGTACCCATACGATGTTCCAGATTACGCTGGTGGTGATGTGGGGTGCTCGGTG
	pc-ZV_NS1(180)-EcoRI-R	ATGAATTCTCAATCACACTCTAATGAATAATCTTCTCTAACC
ZIKV NS1 wing domain	pc-NheI-HA-ZIKV_NS1(31)-F	TTGCTAGCGCCACCATGTACCCATACGATGTTCCAGATTACGCTGGTGGTAGGTACAAGTACCATCCTGACT
ZIKV NS1 β-ladder domain	pc-NheI-HA-ZIKV_NS1(181)-F	TTGCTAGCGCCACCATGTACCCATACGATGTTCCAGATTACGCTGGTGGTCCAGCCGTCATTGGAACAG
	pc-ZIKV_NS1-FLAG-EcoRI-R	ATGAATTCTCACTTGTCGTCATCGTCTTTGTAGTCACCACCTGCAGTCACCATTGACCTTAC
pCDH-GRP78	pCDH-NheI_GRP78_F3	CTAGCTAGCGCCACCATGAAGCTCTCCCTGGTG
	GRP78_BamHI_pCDH_R	CGGGATCCGCCGCCCAACTCATCTTTTTCTGCTG

Supplemental Table S2. Mutagenic primers for QuikChange site directed mutagenesis of pEGFP-full length GRP78 plasmid

Mutation	Primer name	Sequence (5'-3')
V429Q	GRP78_V429Q-F	GTATTGAAACT**CA**GGGAGGTGTCATGACCAAACTGATTCC
	GRP78_V429Q-R	GGTCATGACACCTCCC**TG**AGTTTCAATACCAAGTGTAAGG
R470E	GRP78_R470E-F	TGAAGGTGAA**GA**ACCCCTGACAAAAGACAATCATCTTCTGGG
	GRP78_R470E-R	TGTCAGGGGT**TC**TTCACCTTCATAGACCTTGATTGTAACAG
H477E	GRP78_H477E-F	GACAAAAGACAAT**G**A**A**CTTCTGGGTACATTTGATCTGACTGG
	GRP78_H477E-R	TACCCAGAAG**T**T**C**ATTGTCTTTTGTCAGGGGTCTTTCACC
R492E	GRP78_R492E-F2	CCTGCTCCT**GAA**GGGGTCCCACAGATTGAAGTCACCTTTG
	GRP78_R492E-R2	TGTGGGACCCC**TTC**AGGAGCAGGAGGAATTCCAGTCAGAT
T518A	GRP78_T518A-F	GACAAGGGT**G**C**C**GGGAACAAAAATAAGATCACAATCACCAATG
	GRP78_T518A-R	TTTGTTCCC**G**G**C**ACCCTTGTCTTCAGCTGTCACTCGAAGAA
K585A	GRP78_K585A-F	GCTGGGAGGT**GCC**CTTTCCTCTGAAGATAAGGAGACCATGGA
	GRP78_K585A-R	CAGAGGAAAG**GGC**ACCTCCCAGCTTTTCTTTATCTCCAATCTG
K621A	GRP78_K621A-F	GACTTCAAAGCTAAGAAG**GCC**GAACTGGAAGAAATTGTTCAACC
	GRP78_K621A-R	GGTTGAACAATTTCTTCCAGTTC**GGC**CTTCTTAGCTTTGAAGTC

Supplemental Table S3. Mutagenic primers for QuikChange site directed mutagenesis of pcDNA-ZIKV E-HA plasmid

Mutation	Primer name	Sequence (5'-3')
S306A	ZIKVE_S306A-F	TGAGCTAC**G**CCCTGTGCACCGCCGCCTTCACCTT
	ZIKVE_S306A-R	CACAGGG**C**GTAGCTCACTCCCTTCAGTCTCAGTT
L307N	ZIKVE_ L307N-F	GAGCTACTCC**AAC**TGCACCGCCGCCTTCACCTTC ACCAAAA
	ZIKVE_ L307N-R	GCGGTGCA**GTT**GGAGTAGCTCACTCCCTTCAGTC TCAGTTTA
C308A	ZIKVE_C308A-F	ACTCCCTG**GC**CACCGCCGCCTTCACCTTCACCAA AATC
	ZIKVE_C308A-R	CGGCGGTG**GC**CAGGGAGTAGCTCACTCCCTTCA GTCTC
T309A	ZIKVE_T309A-F	CCTGTGC**G**CCGCCGCCTTCACCTTCACCAAA
	ZIKVE_T309A-R	GGAGTGA**G**CTACTCCCTGTGCGCCGCCGC
A310S	ZIKVE_A310S-F	TGCACC**T**CCGCCTTCACCTTCACCAAAATCC
	ZIKVE_A310S-R	AAGGCGG**A**GGTGCACAGGGAGTAGCTCACTC
A311S	ZIKVE_A311S-F	ACCGCC**T**CCTTCACCTTCACCAAAATCCCAG
	ZIKVE_A311S-R	TGAAGG**A**GGCGGTGCACAGGGAGTAGCTC
A333S	ZIKVE_A333S-F	CAGTAT**T**C**C**GGCACTGACGGCCCTTGTAA
	ZIKVE_A333S-R	AGTGCC**G**G**A**ATACTGGACTTCCACTGTGA
G334V	ZIKVE_ G334V-F	ATGCTG**TG**ACTGACGGCCCTTGTAAGGT
	ZIKVE_ G334V-R	CGTCAGT**CA**CAGCATACTGGACTTCCACTG
T335A	ZIKVE_ T335A-F	CTGGC**G**C**C**GACGGCCCTTGTAAGGTGCC
	ZIKVE_ T335A-R	GCCGTC**G**G**C**GCCAGCATACTGGACTTCCAC
D336K	ZIKVE_ D336K-F	TGGCACT**A**A**G**GGCCCTTGTAAGGTGCCTGCAC
	ZIKVE_ D336K-R	AGGGCC**C**T**T**AGTGCCAGCATACTGGACTTC
G337V	ZIKVE_ G337V-F	ACTGACG**TG**CCTTGTAAGGTGCCTGCACAG
	ZIKVE_ G337V-R	ACAAGG**CA**CGTCAGTGCCAGCATACTGGA
E367K	ZIKVE_E367K-F	TTACA**A**AGAGCACTGAAAACTCCA
	ZIKVE_E367K-R	GCTCT**T**TGTAATGACAGGATTTGC
S368A	ZIKVE_S368A-F	ACAGAG**GC**CACTGAAAACTCCAAAATGAT
	ZIKVE_S368A-R	TCAGTG**GC**CTCTGTAATGACAGGATTTG
T369A	ZIKVE_T369A-F	AGAGAGC**G**C**C**GAAAACTCCAAAATGATGCTG
	ZIKVE_T369A-R	AGTTTTC**G**G**C**GCTCTCTGTAATGACAGGATT
E370K	ZIKVE_E370K-F	GAGCACT**A**A**G**AACTCCAAAATGATGCTGGAG
	ZIKVE_E370K-R	GGAGTT**C**T**T**AGTGCTCTCTGTAATGACAGG
N371A	ZIKVE_N371A-F	ACTGAA**GC**CTCCAAAATGATGCTGGAGC
	ZIKVE_N371A-R	TTTGGAG**GC**TTCAGTGCTCTCTGTAATGA
E393K	ZIKVE_E393K-F	TCGGG**A**A**G**AAGAAAATCACACACCATTGG
	ZIKVE_E393K-R	TTTCTT**C**T**T**CCCGACGCCAATCACGATATA
K394A	ZIKVE_K394A-F	CGGGGAA**GCC**AAAATCACACACCATTGGCACC
	ZIKVE_K394A-R	GTGATTTT**GGC**TTCCCCGACGCCAATCACGATA

Supplemental Table S4. NCBI accession number of HSP70 protein family

Protein	Organism	NCBI Accession	Gene
Human GRP78	Homo sapiens	NP_005338	HSPA5
Hamster GRP78	Cricetulus griseus	NP_001233668	HSPA5
DnaK	Escherichia coli	NP_414555	dnaK
SSA1	Saccharomyces cerevisiae	NP_009396	SSA1
Heat shock cognate 71 kDa protein isoform 1 (HSC70)	Homo sapiens	NP_006588	HSPA8
Heat shock cognate 71 kDa protein isoform 1 (HSC70)	Mus musculus	NP_112442	HSPA8
Heat shock 70 kDa protein 1A (HSP70)	Homo sapiens	NP_005336	HSPA1
Heat shock 70 kDa protein 1A (HSP70)	Mus musculus	NP_034609	HSPA1

Figure 1C: EGFP

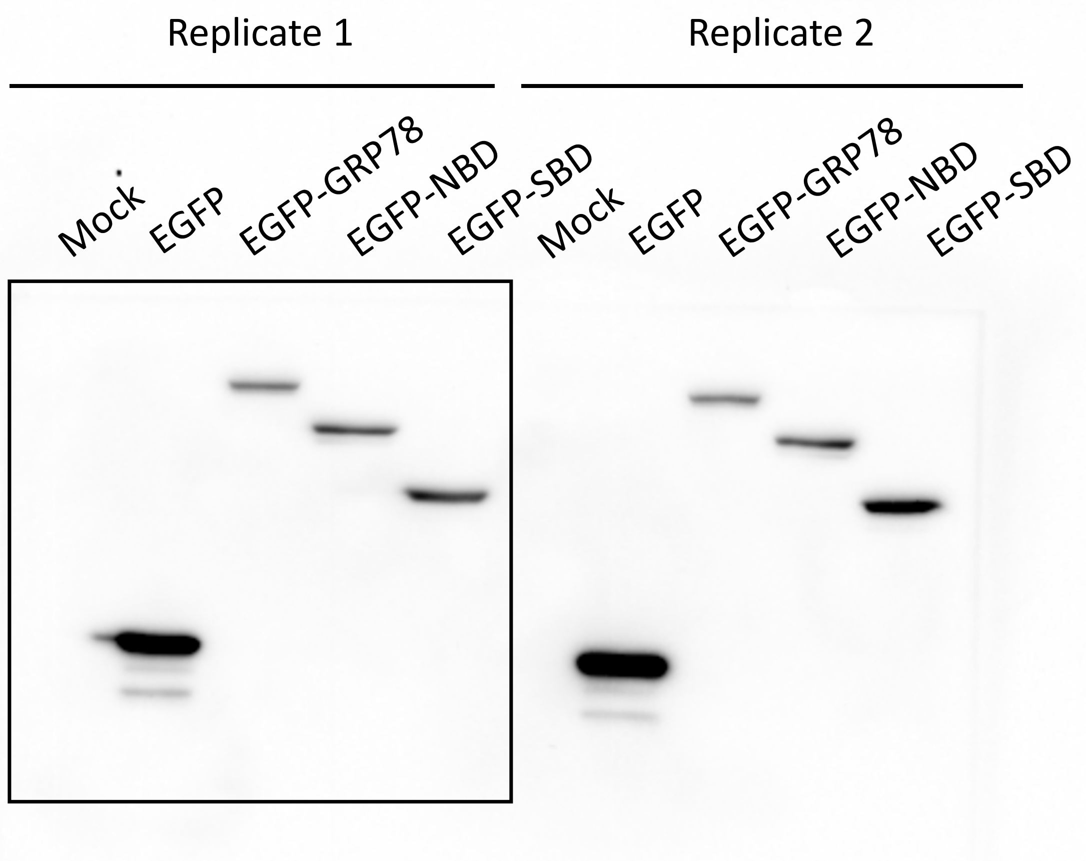

Figure 1C: Actin

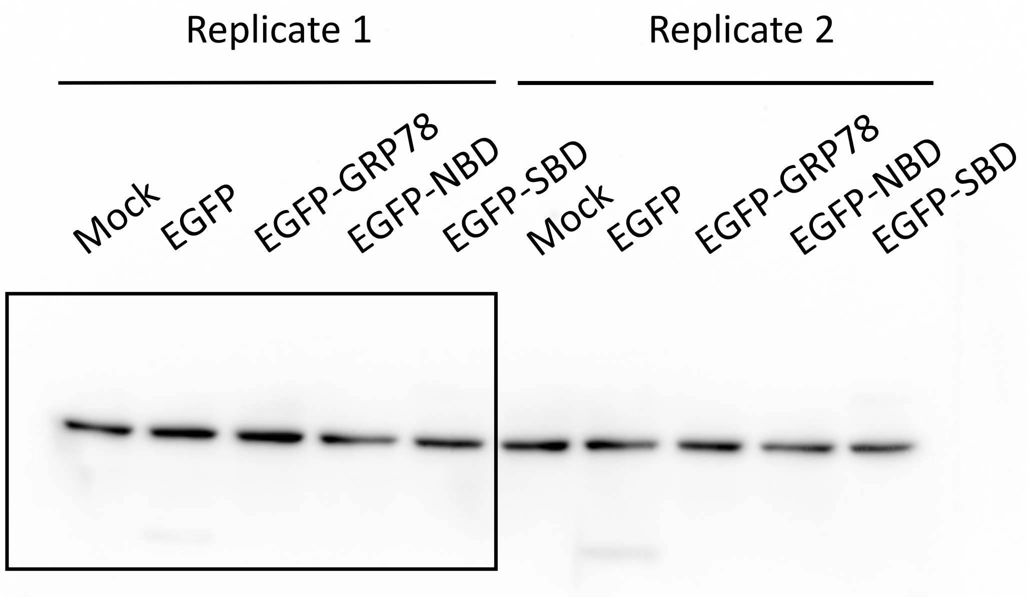

Figure 1D: Input EGFP detection

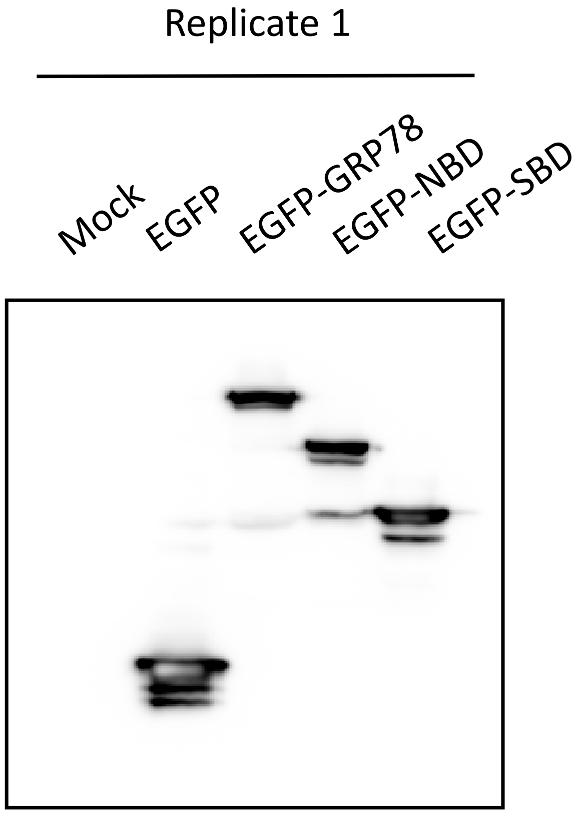

Figure 1D: Output EGFP

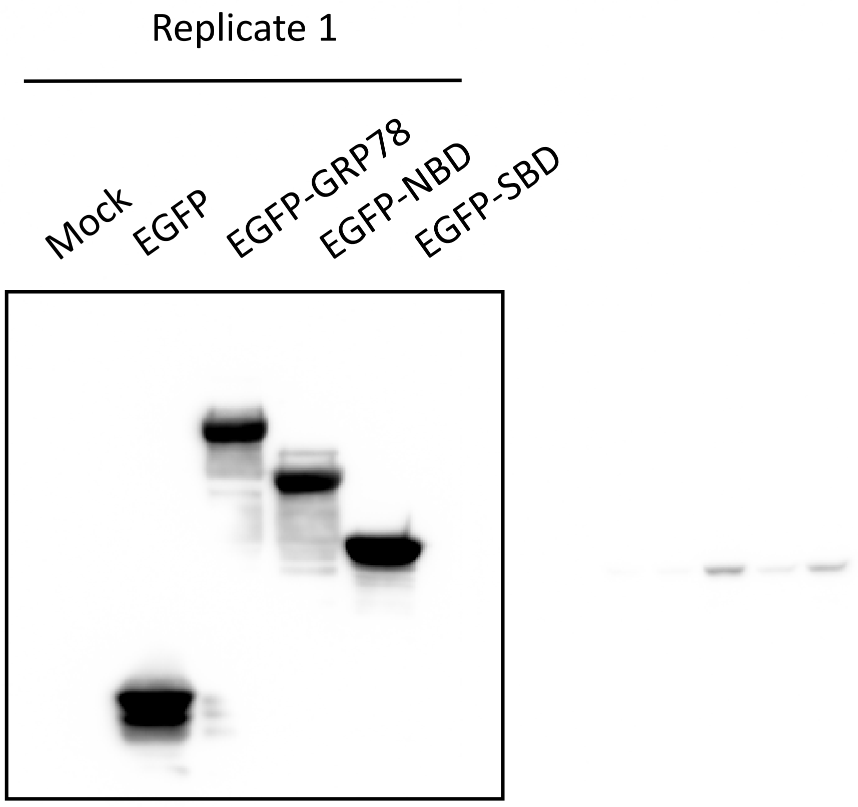

Output EGFP replicate 2 and 3 of Figure 1D

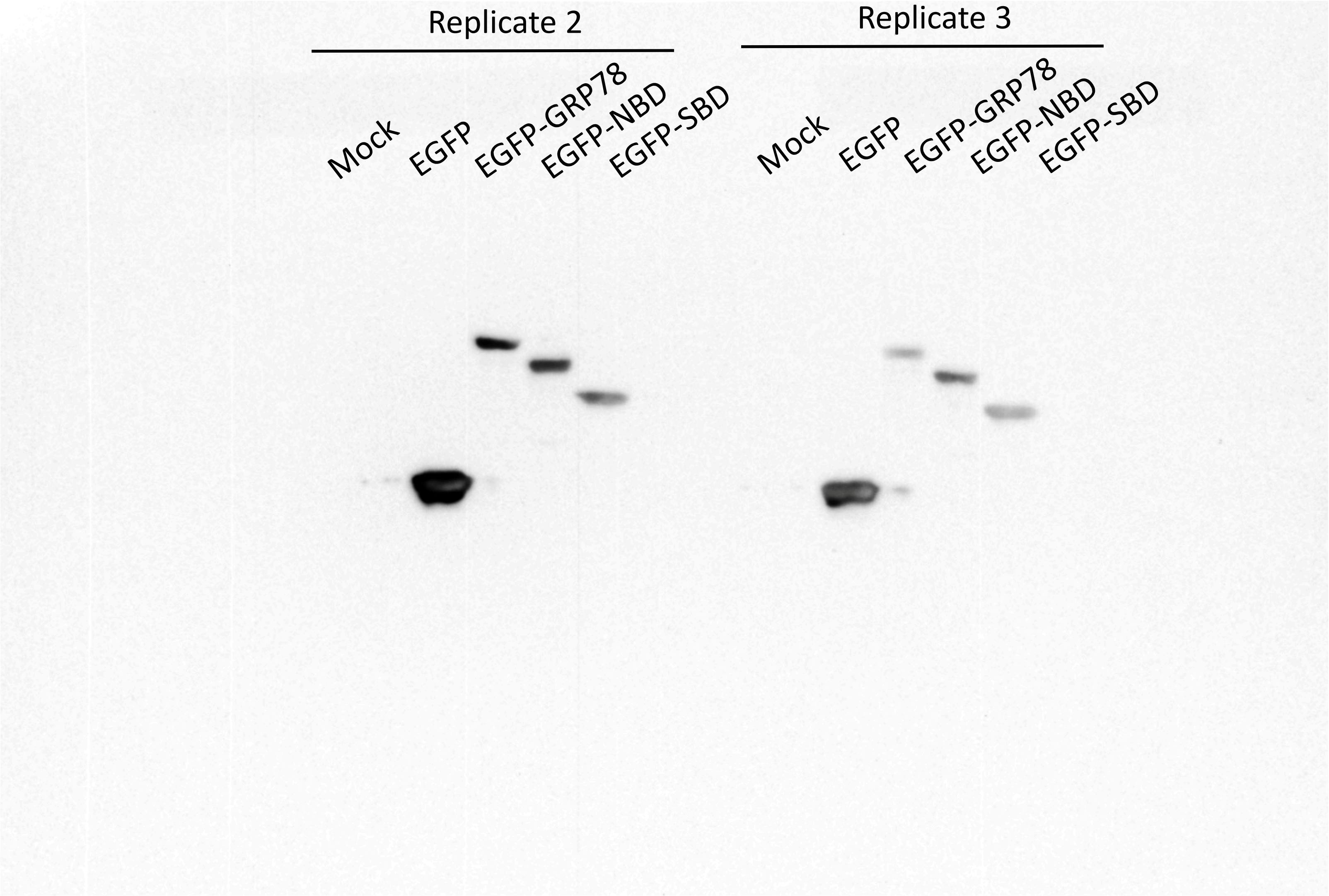

Figure 1D: Input ZIKV E

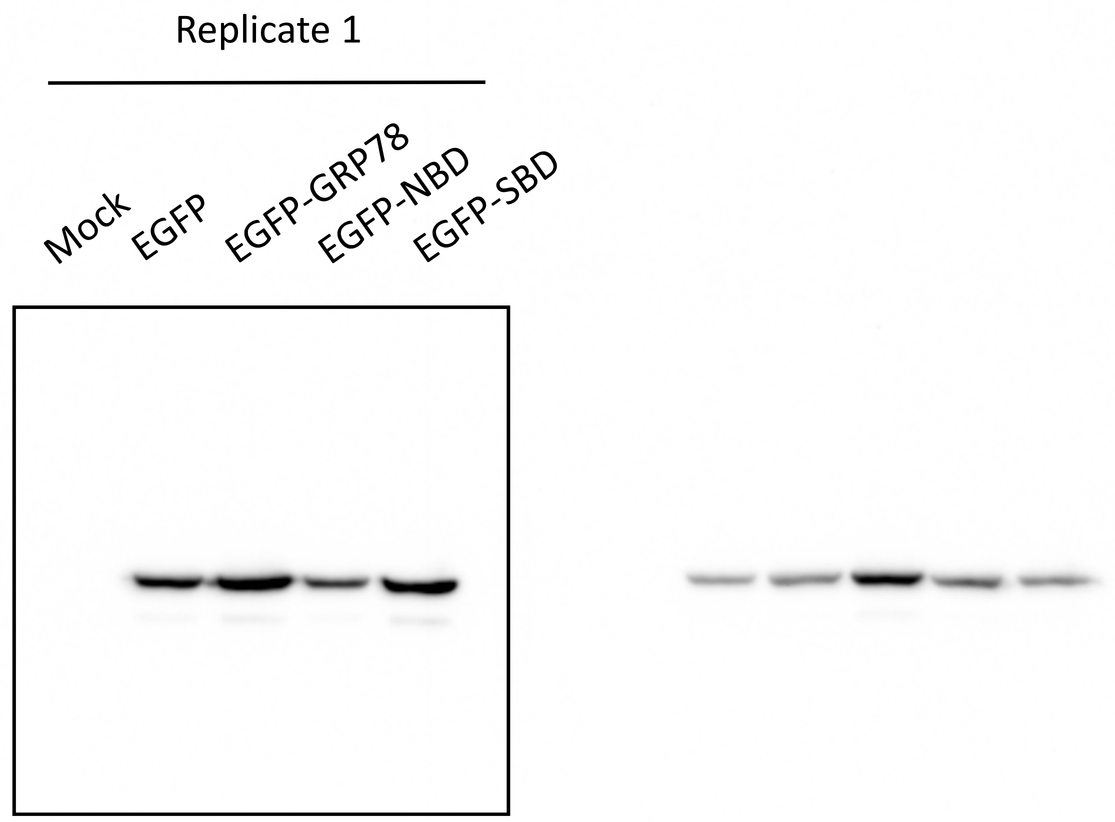

Input ZIKV E replicate 2 and 3 of Figure 1D

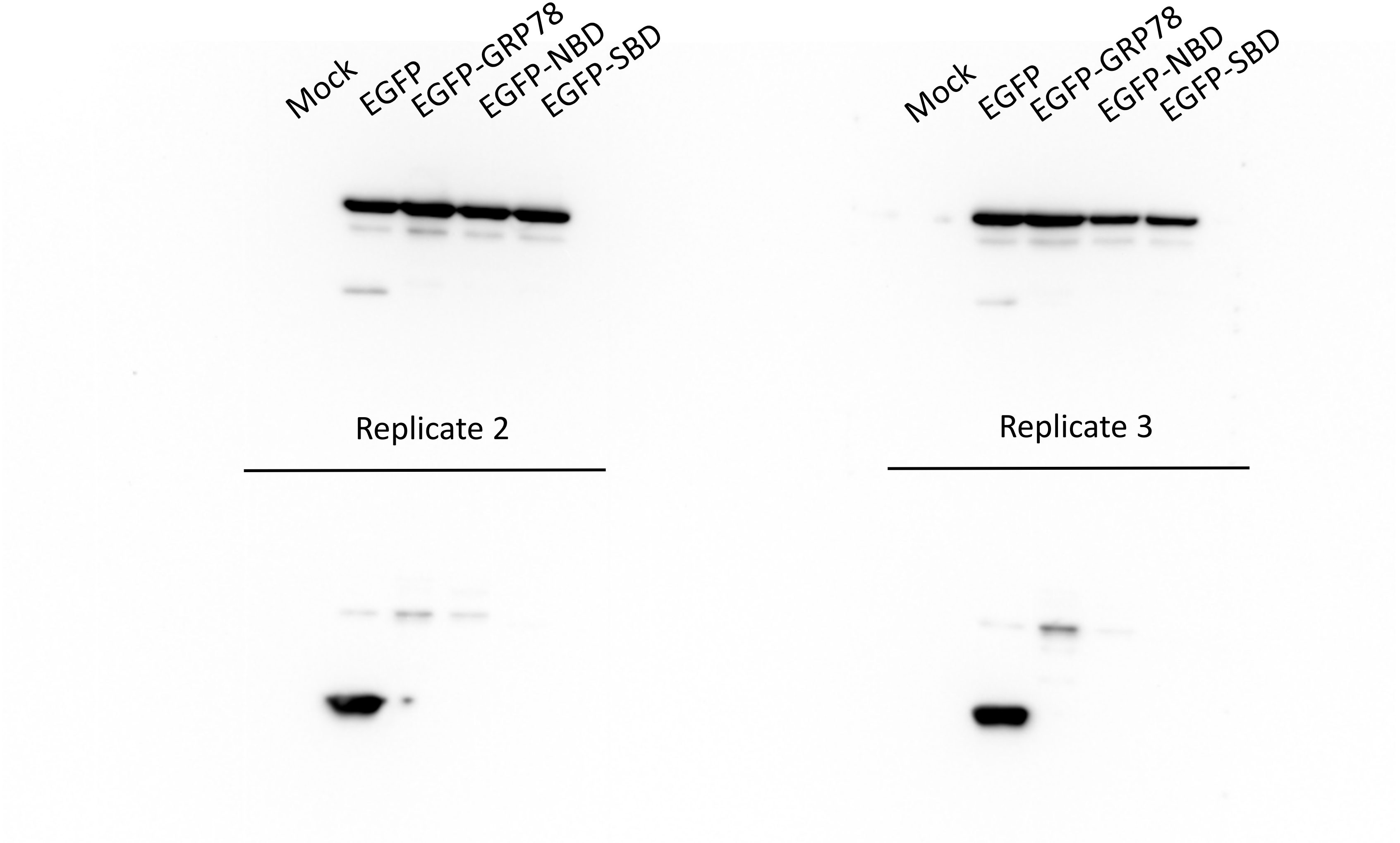

Figure 1D: Output ZIKV E

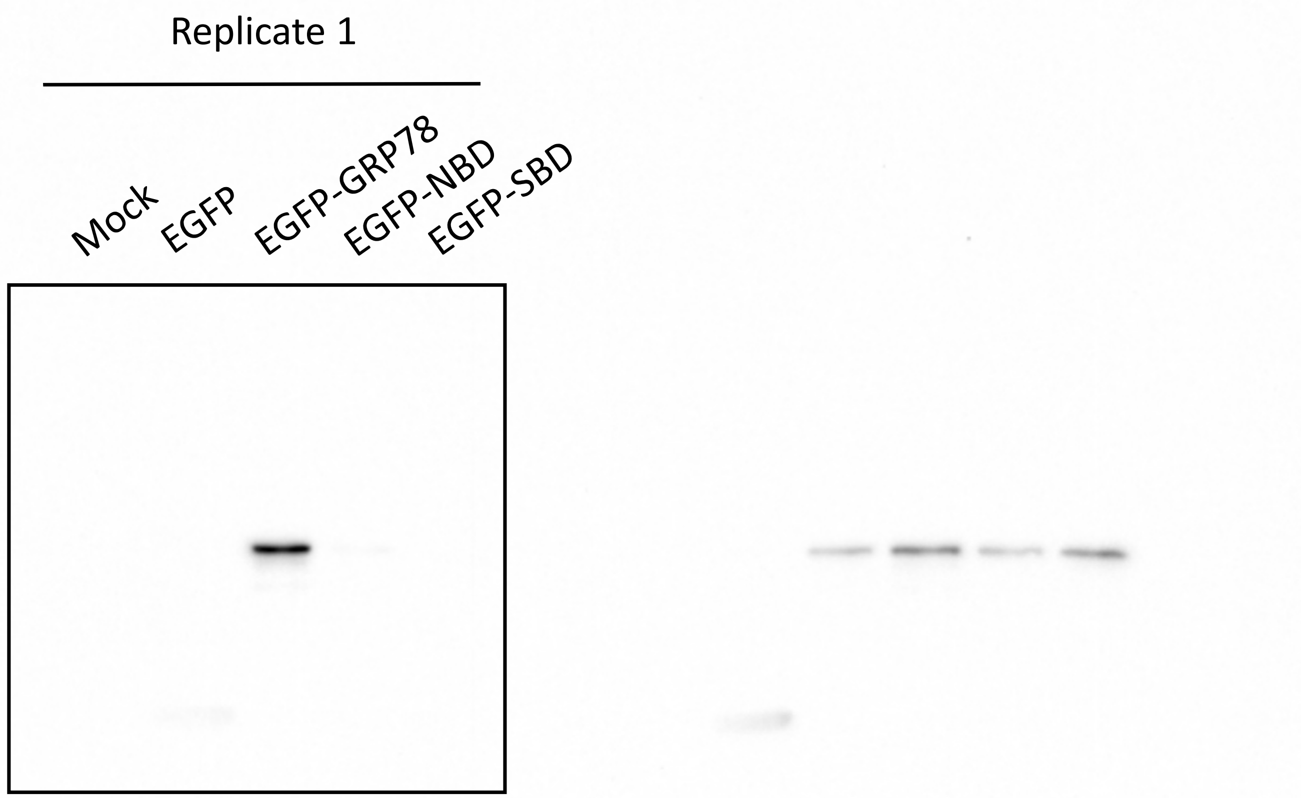

Output ZIKV E replicate 2 and 3 of Figure 1D

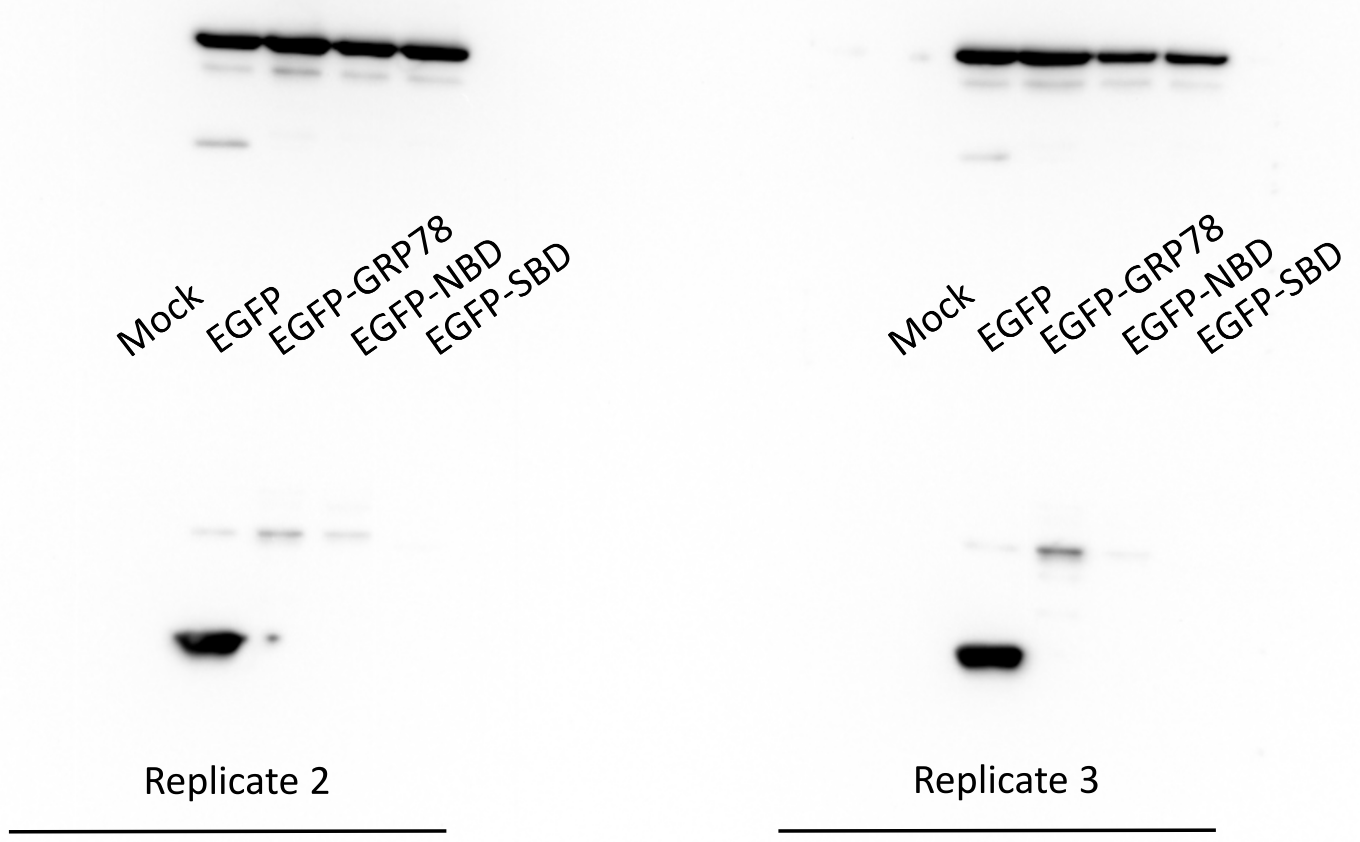

Figure 1E: Input EGFP-GRP78

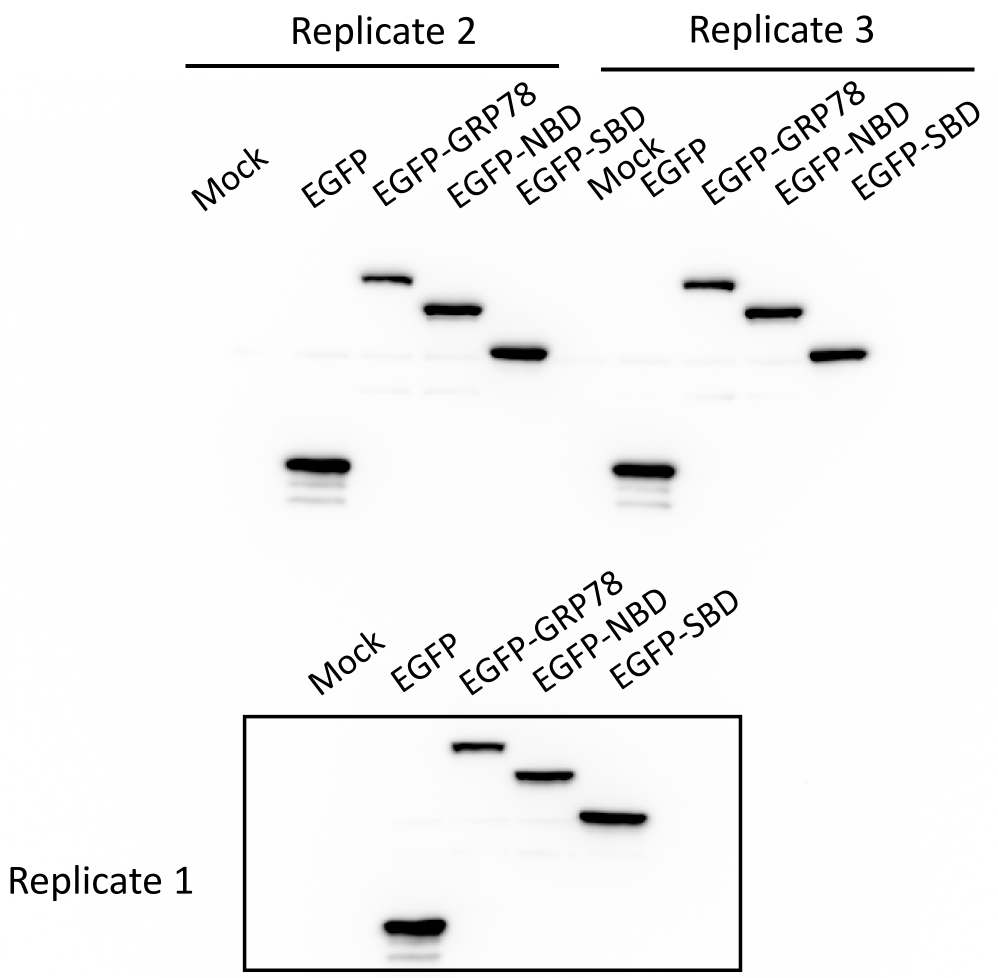

Figure 1E: Output EGFP-GRP78

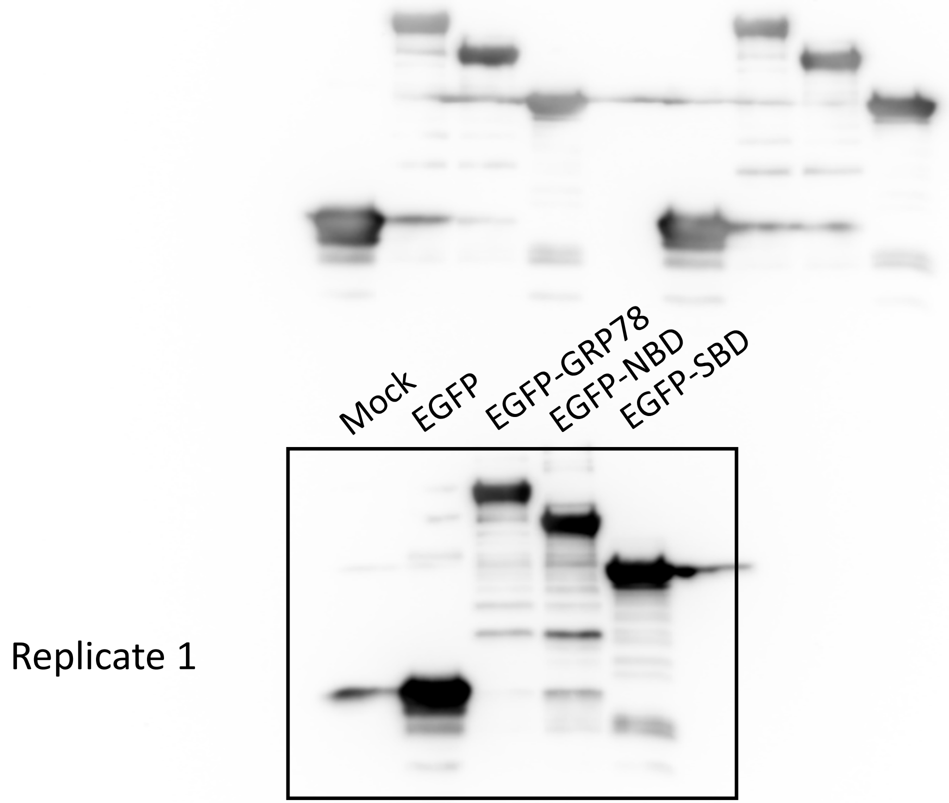

Output EGFP-GRP78 replicate 2 and 3 of Figure 1E

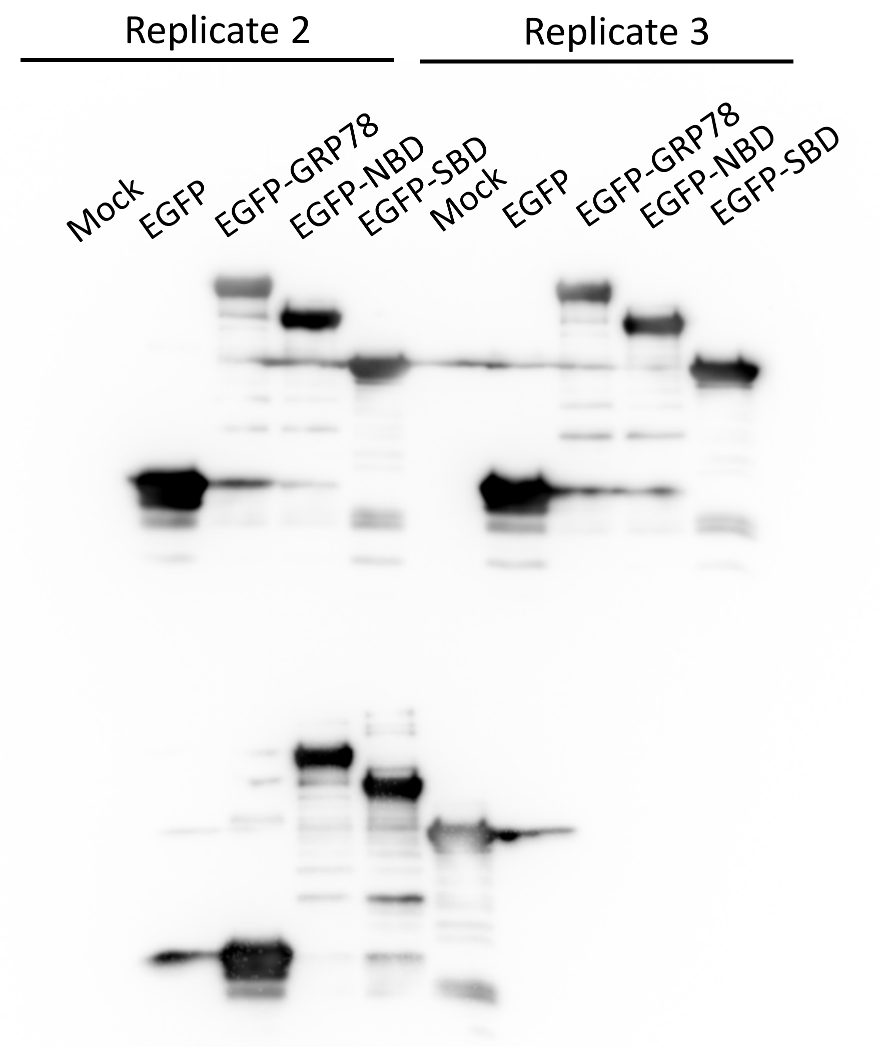

Figure 1D: Input ZIKV NS1

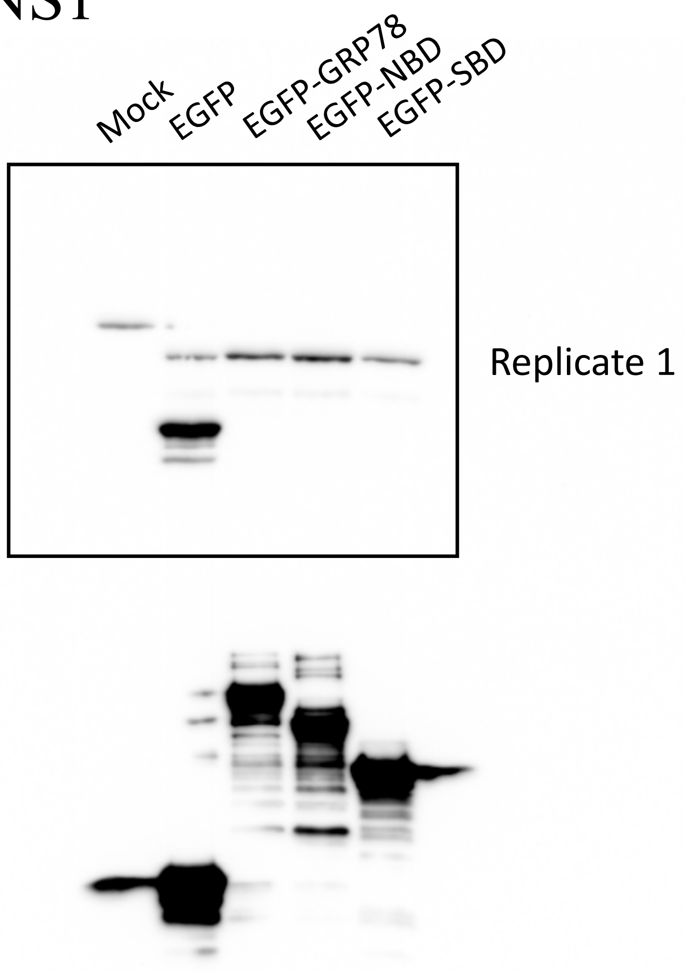

Input ZIKV NS1 replicate 2 and 3 of Figure 1E

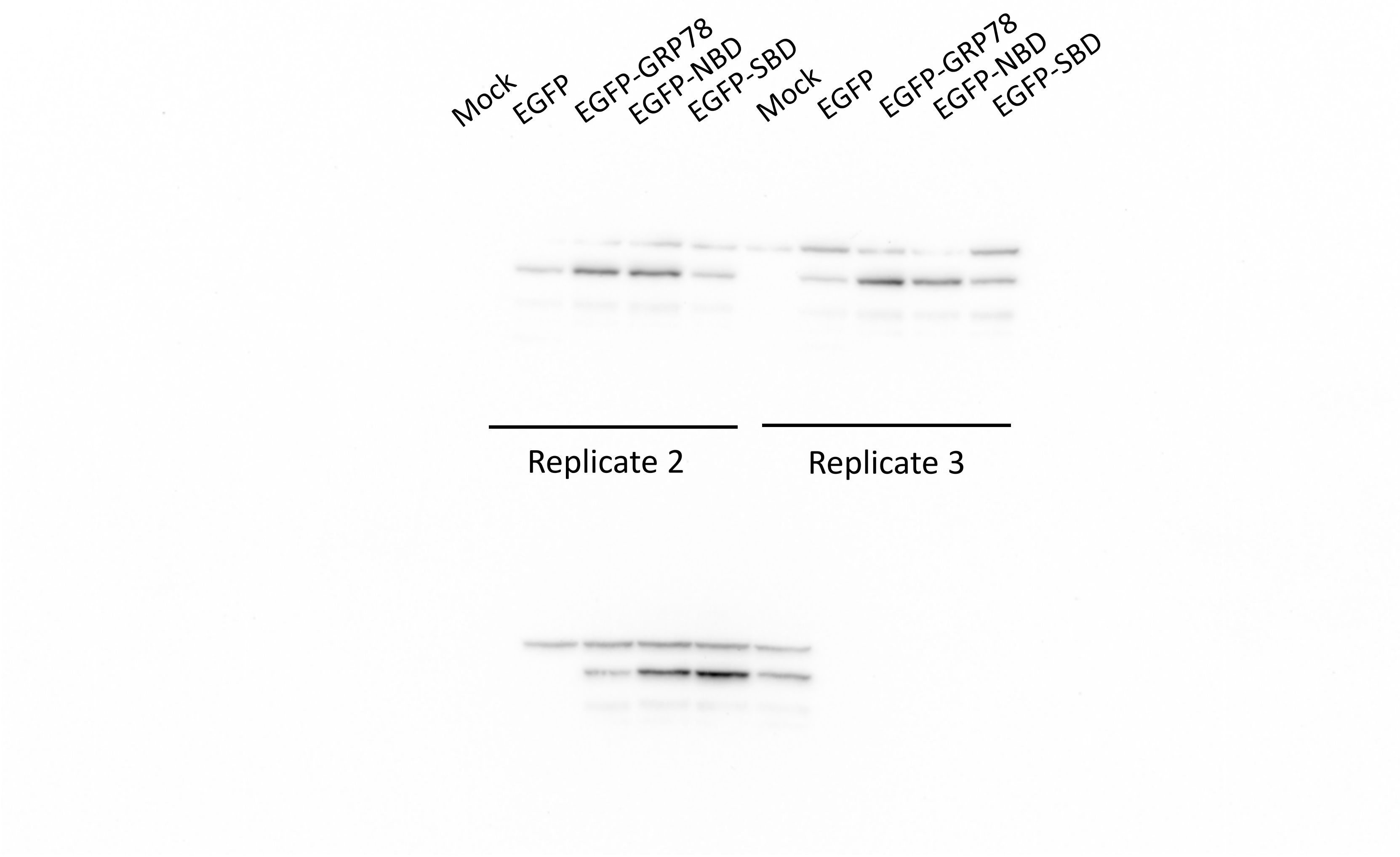

Figure 1E: Output ZIKV NS1

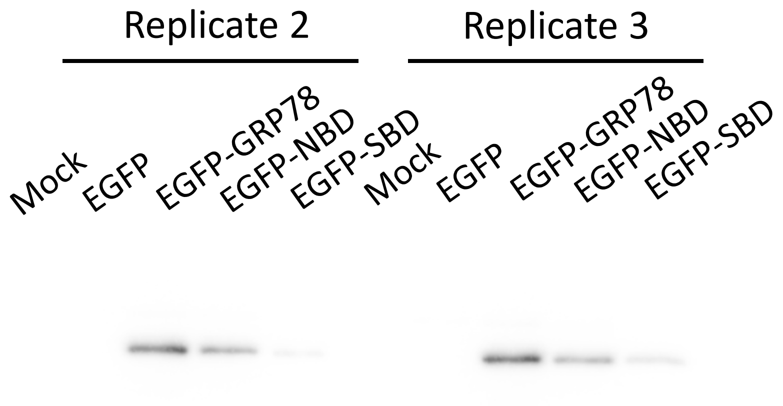

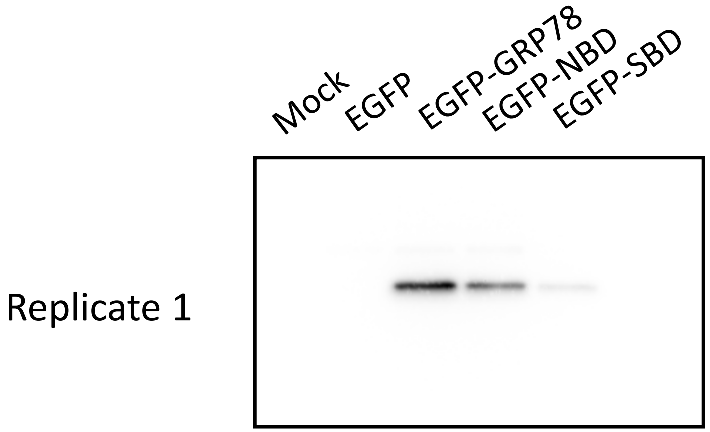

Figure 2B: Input ZIKV E

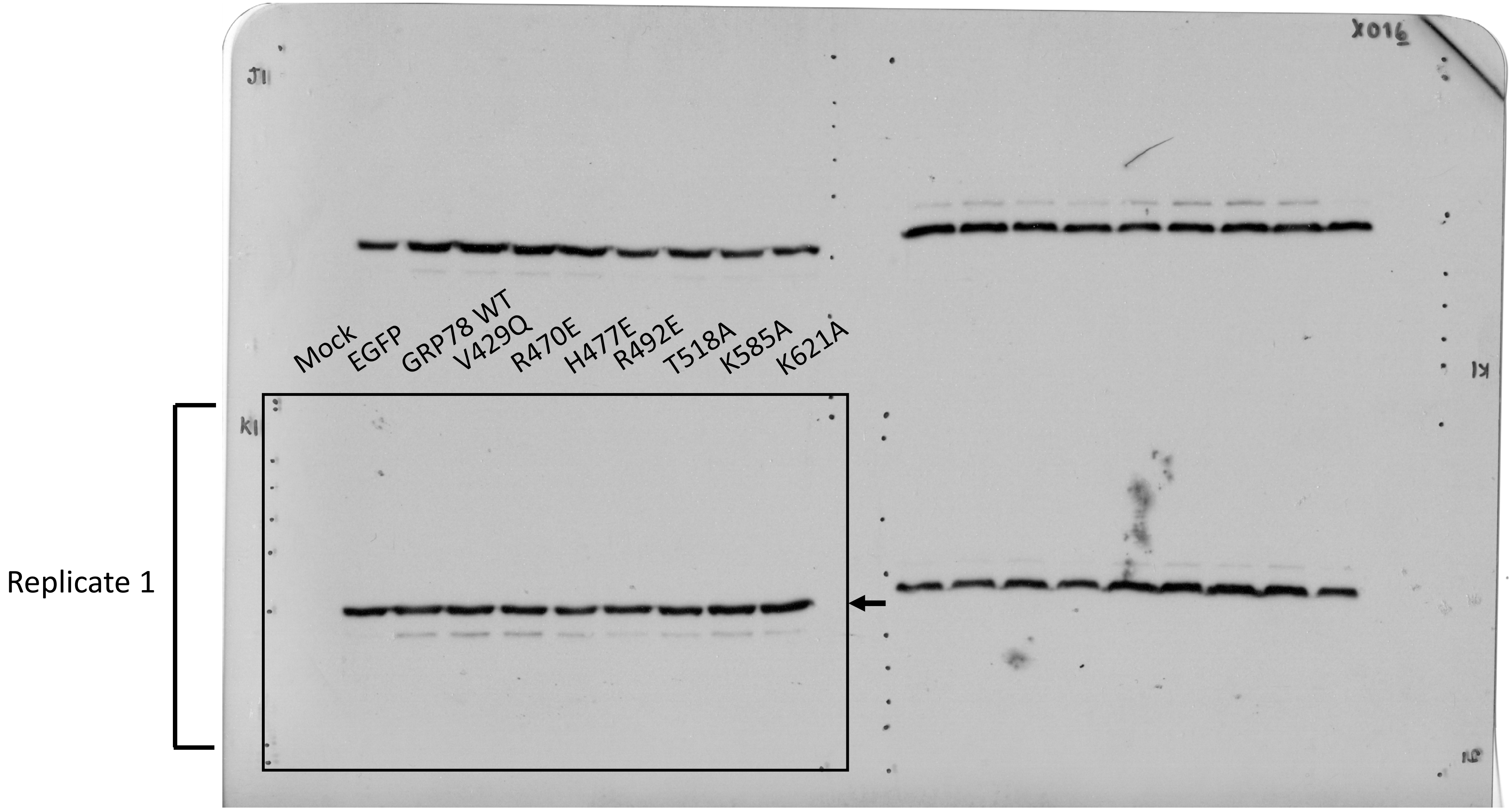

Input ZIKV E replicate 2 of Figure 2B

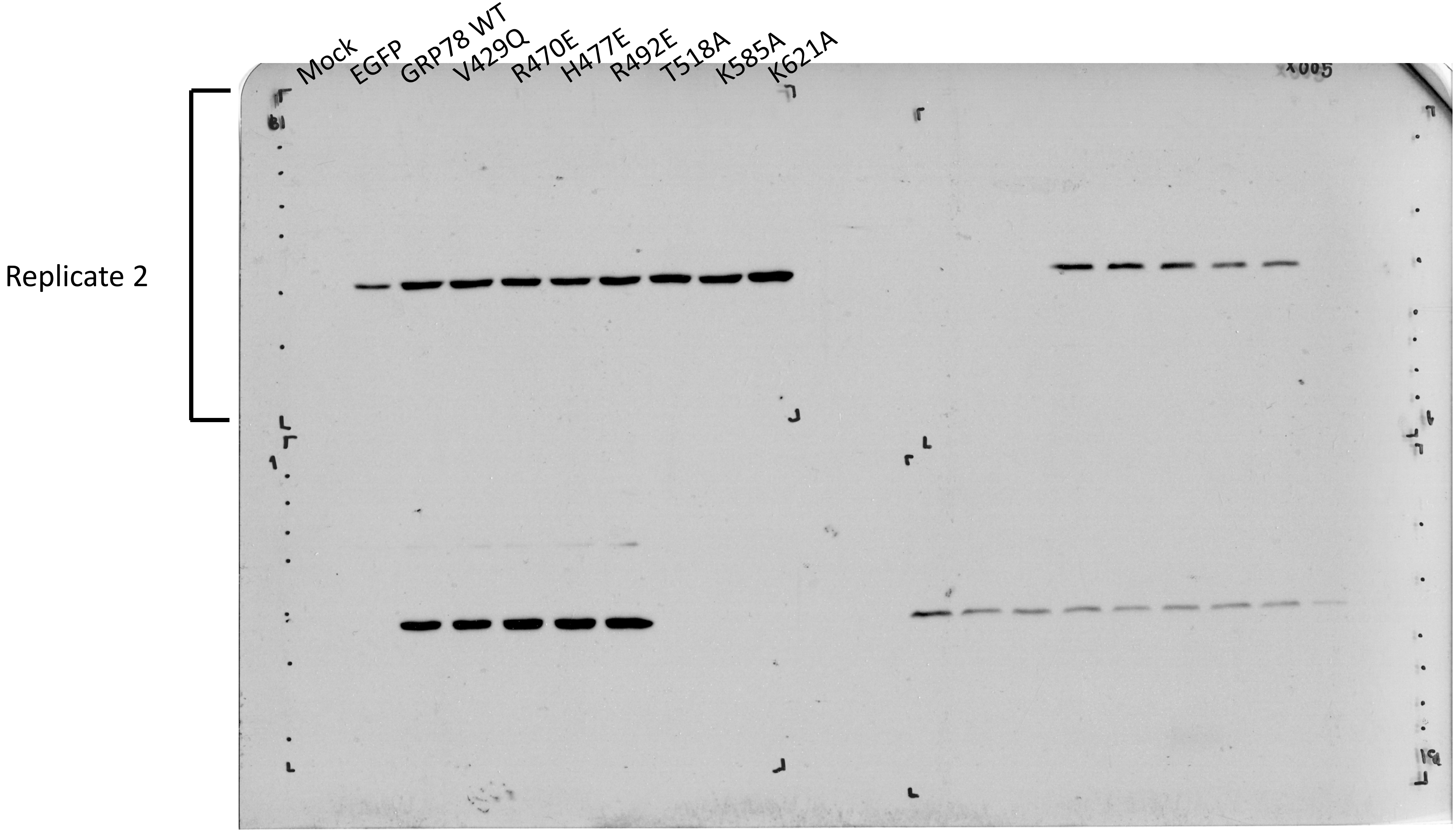

Input ZIKV E replicate 3 of Figure 2B

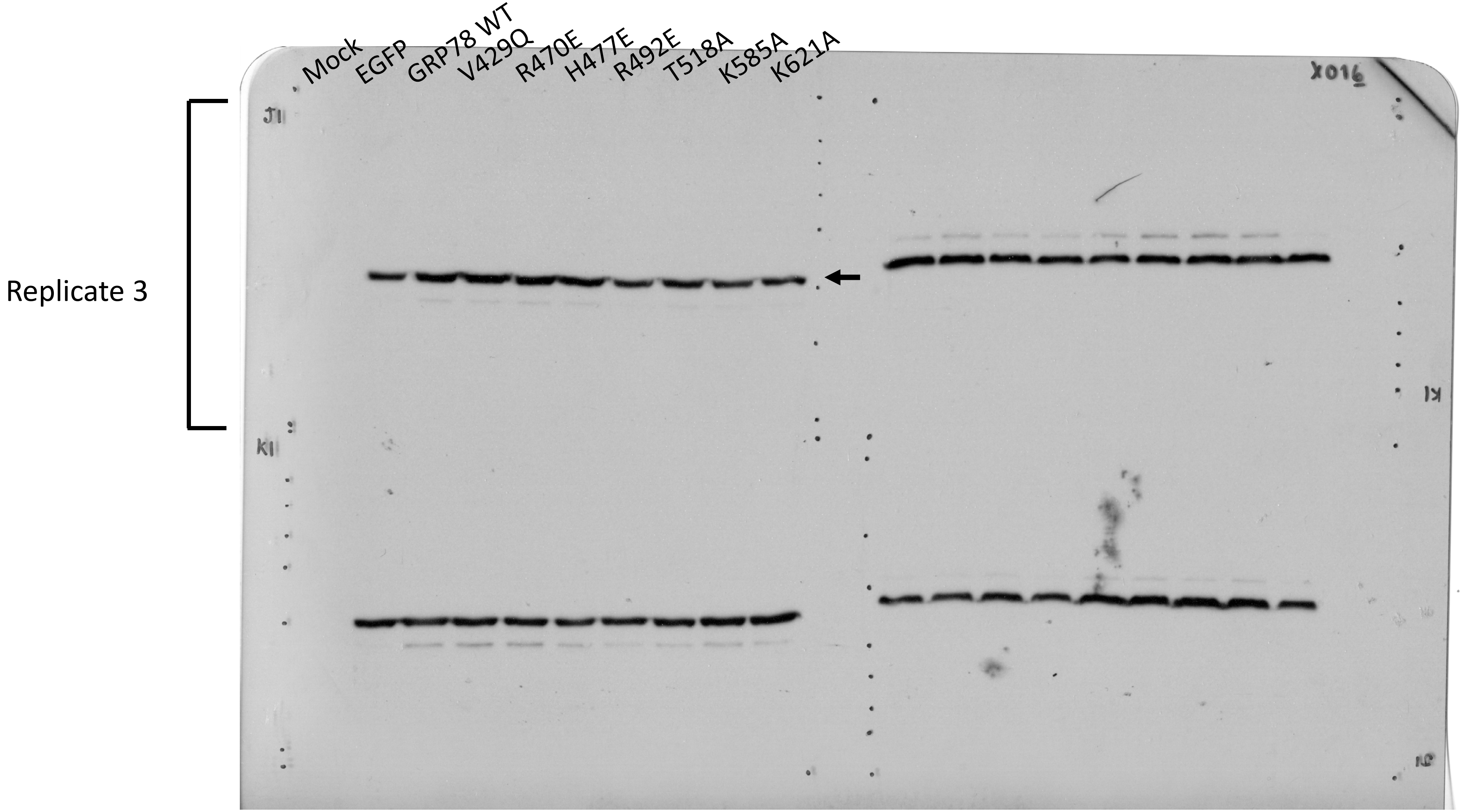

Figure 2B: Input ATF6

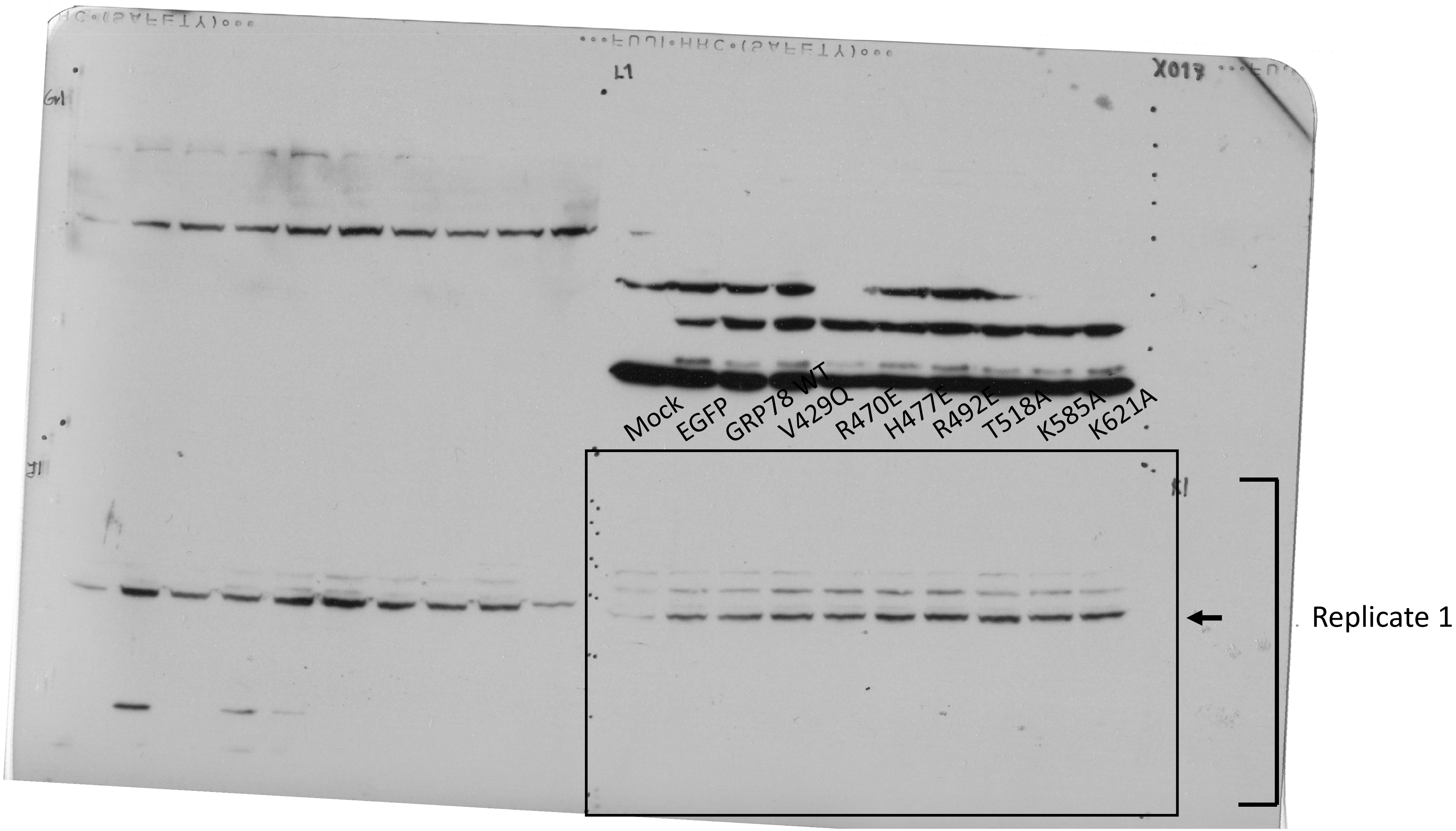

Input ATF6 replicate 2 of Figure 2B

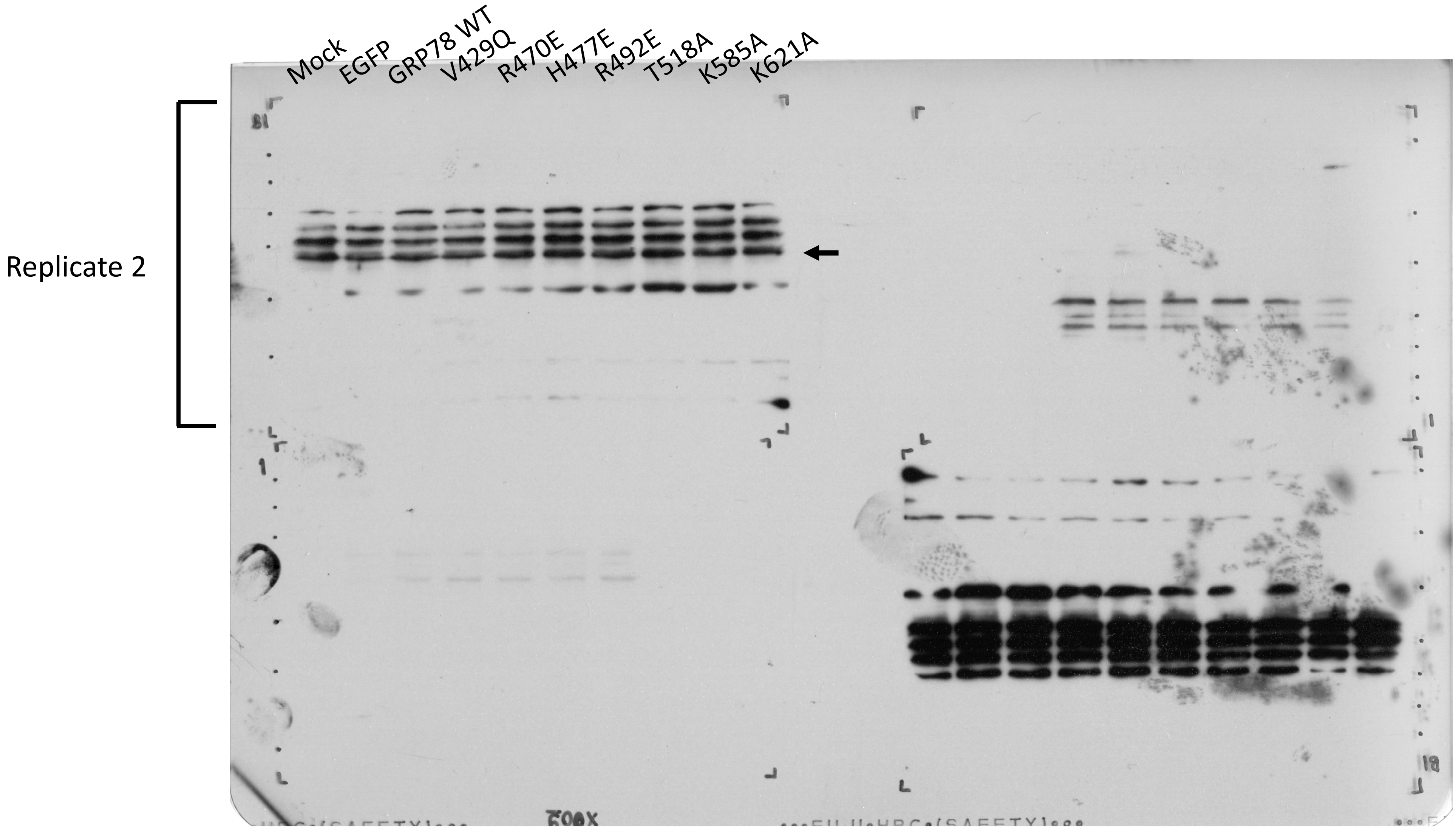

Input ATF6 replicate 3 of Figure 2B

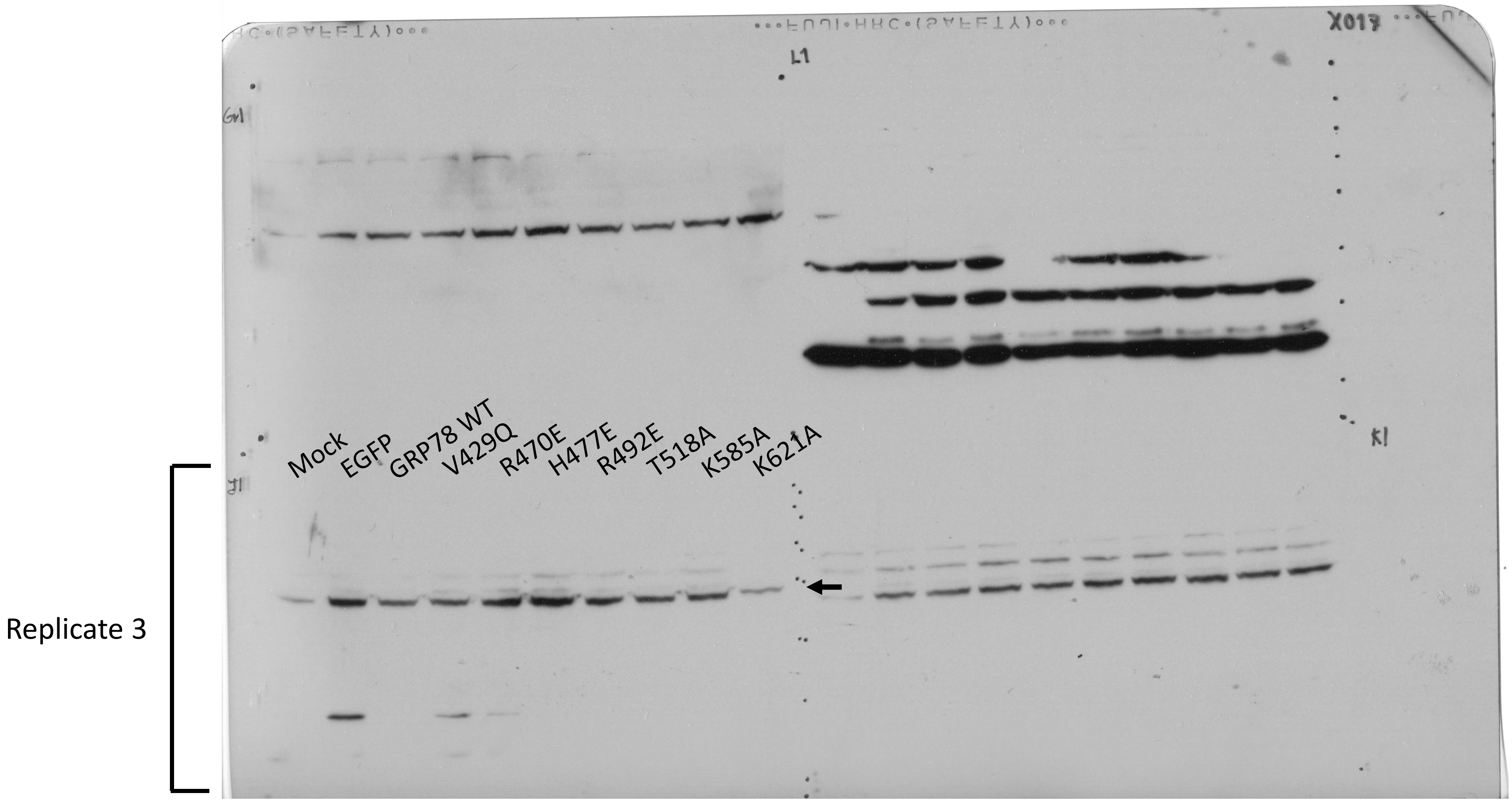

Figure 2B: Input EGFP-GRP78

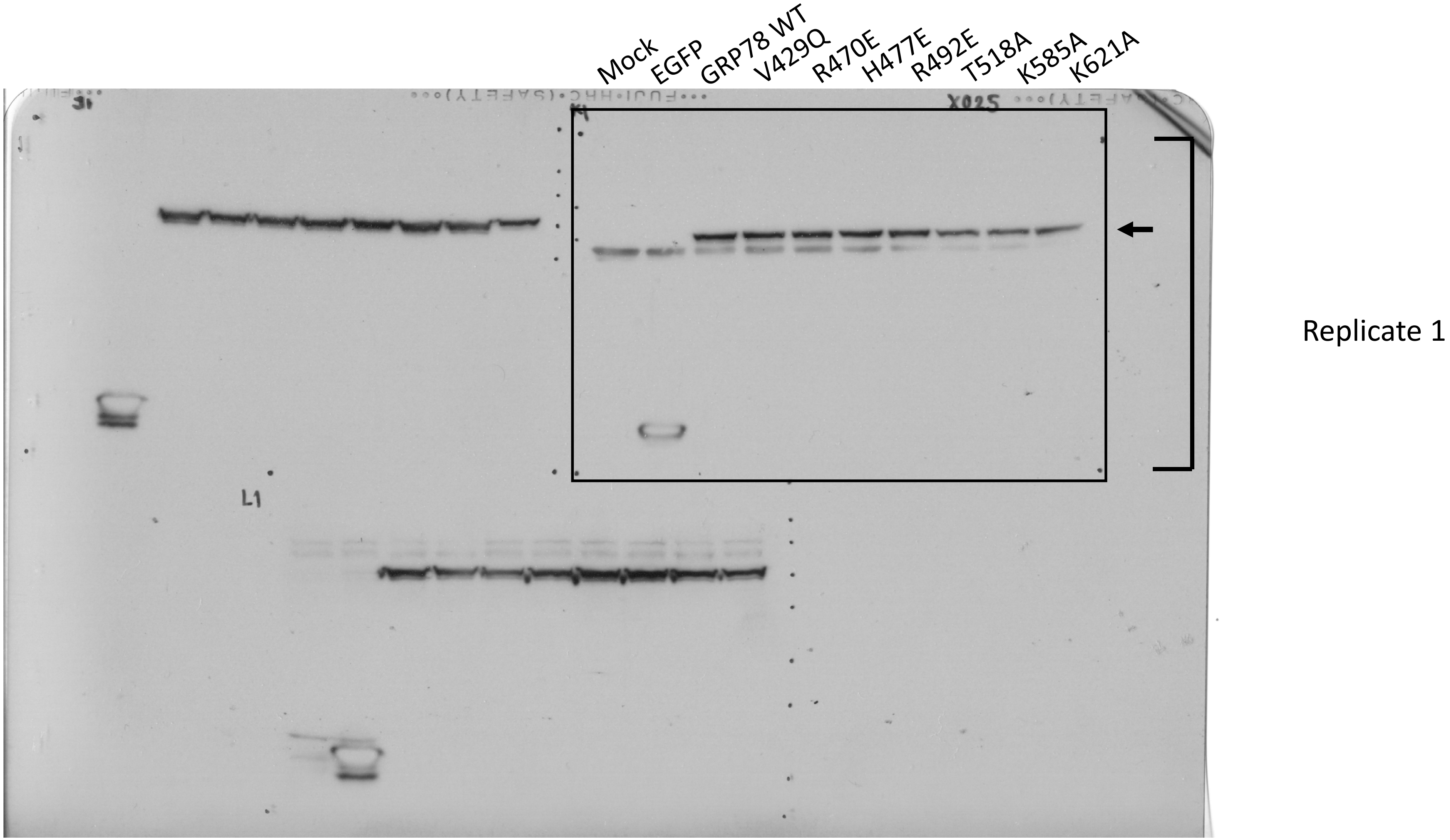

Input EGFP-GRP78 replicate 2 of Figure 2B

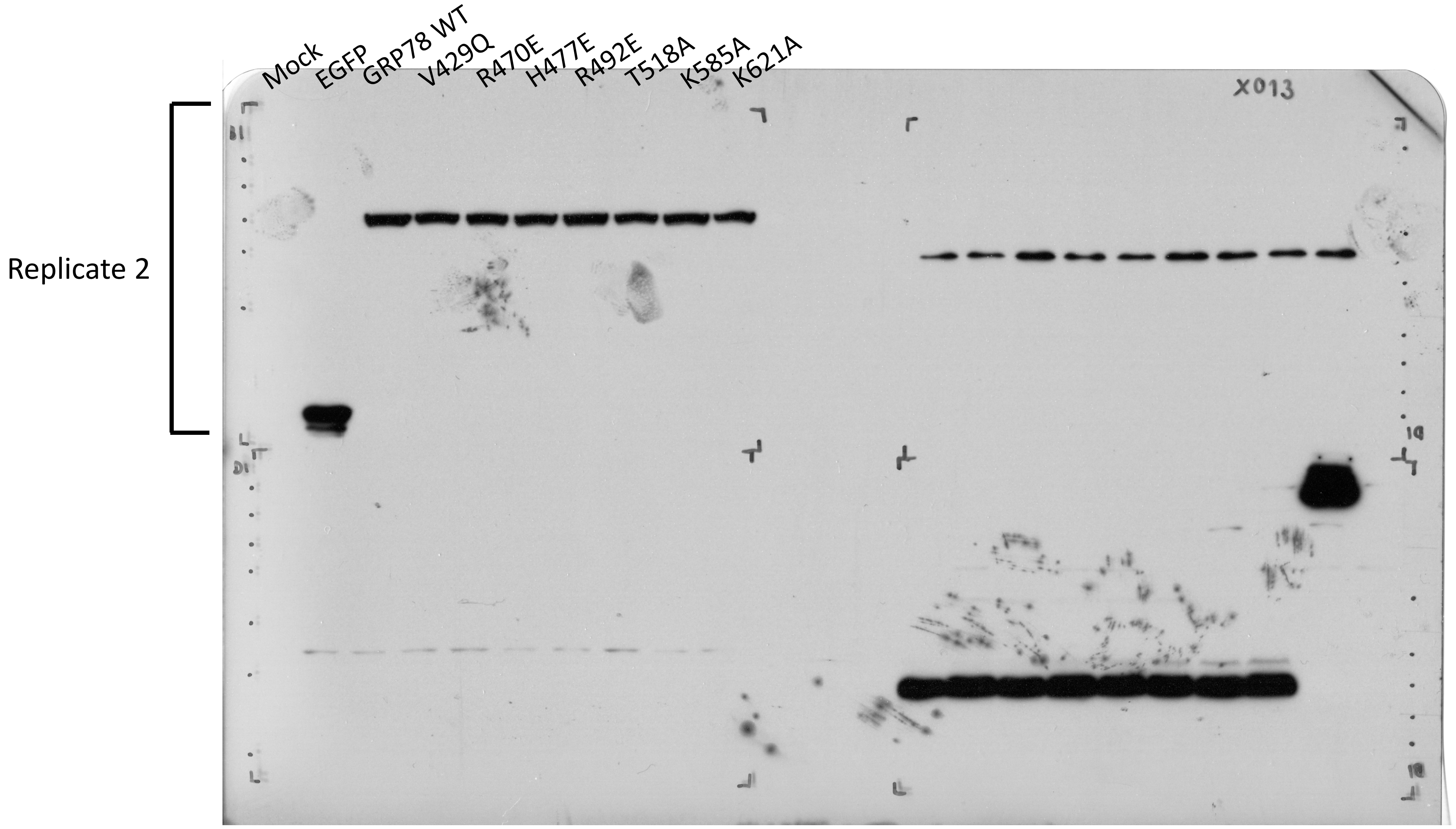

Input EGFP-GRP78 replicate 3 of Figure 2B

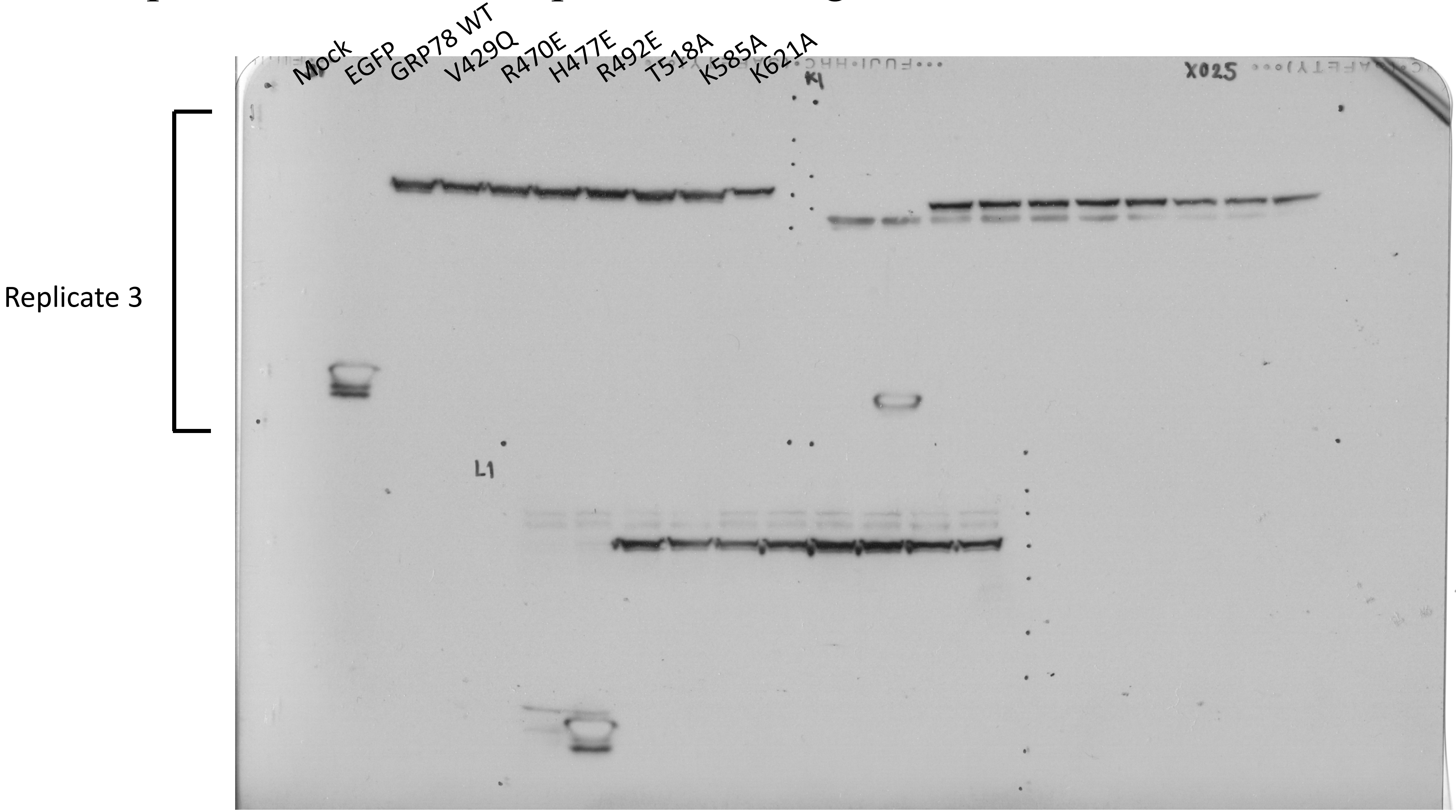

Figure 2B: Input Actin

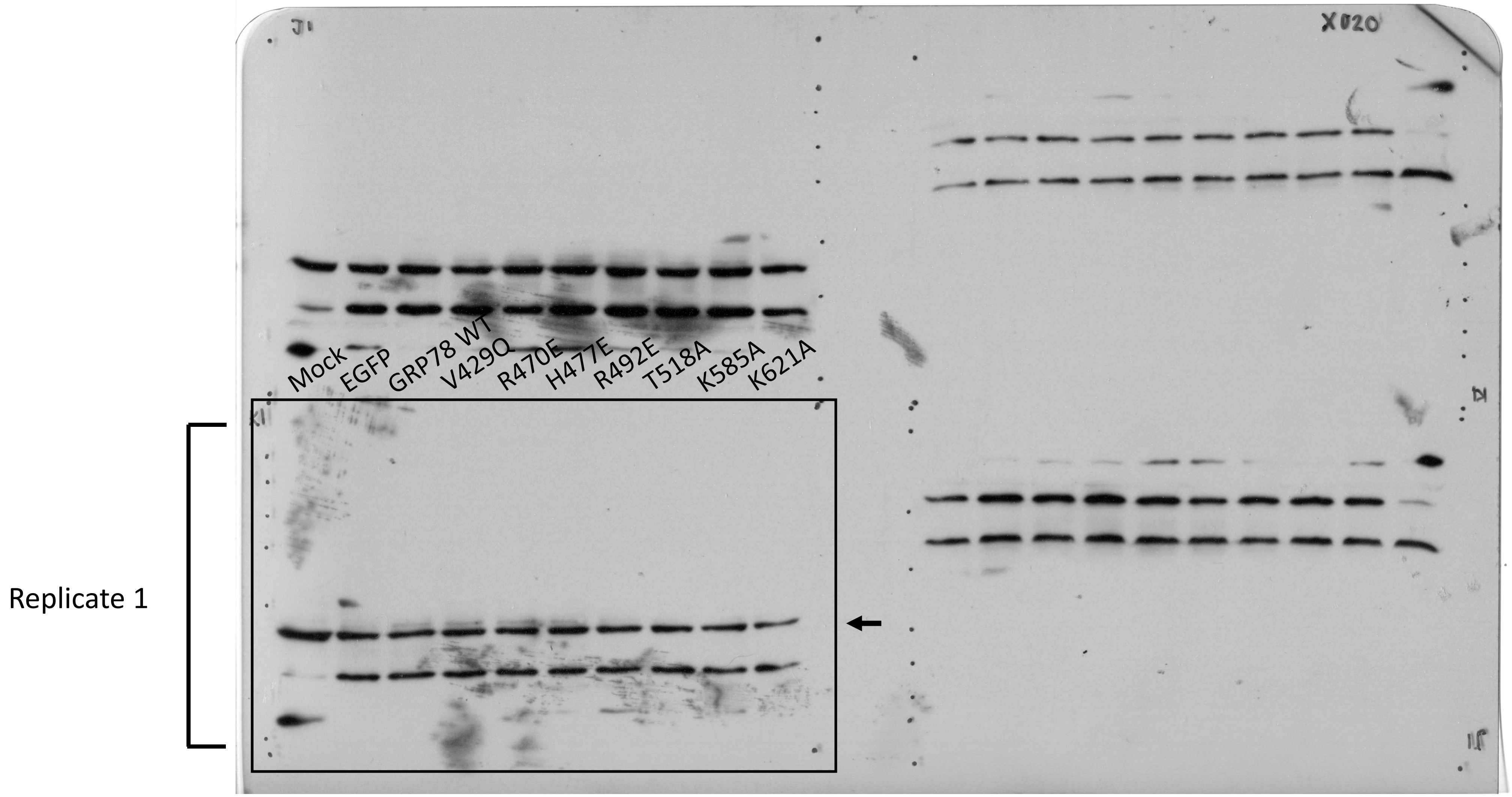

Input actin replicate 2 of Figure 2B

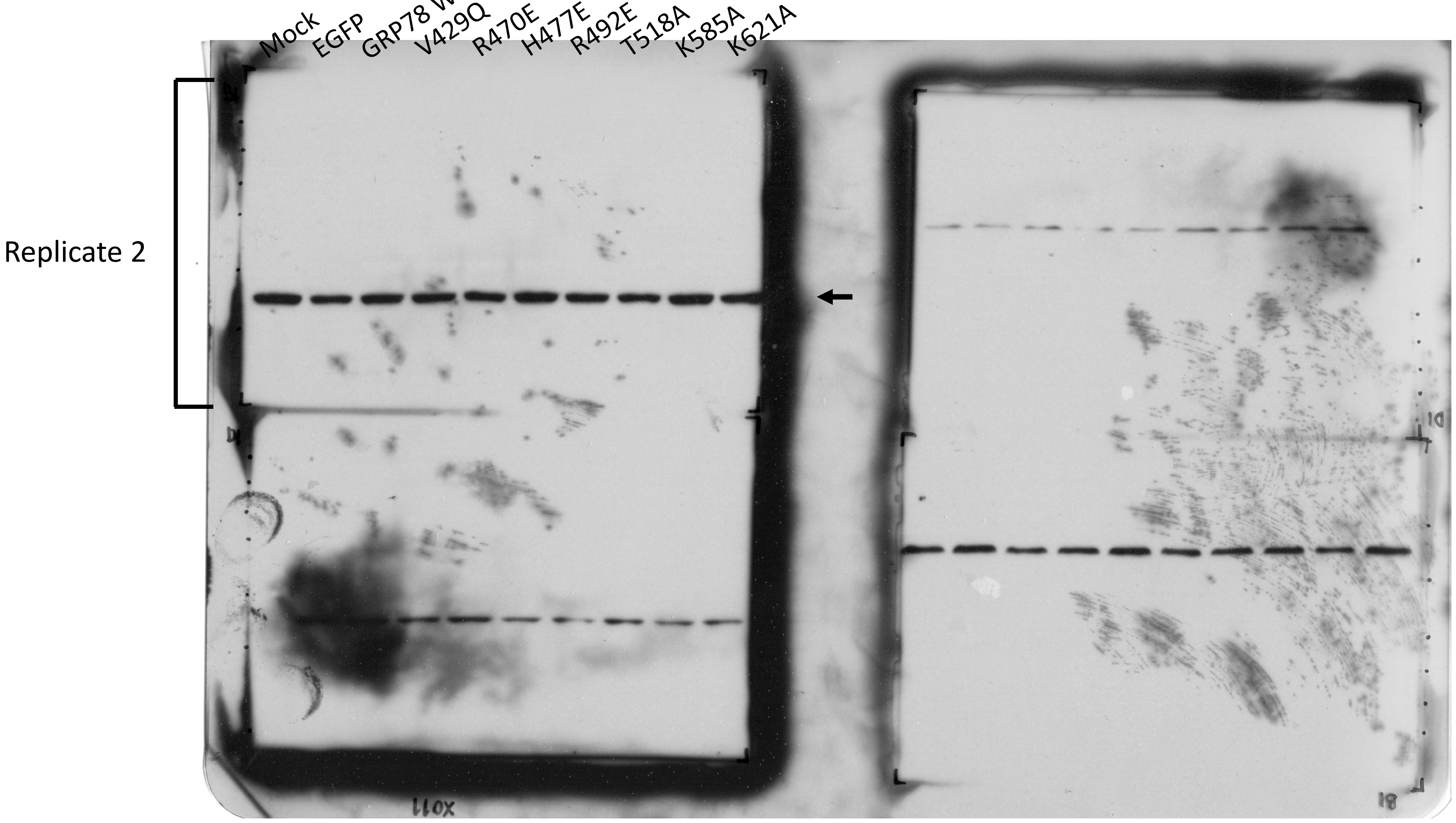

Input actin replicate 3 of Figure 2B

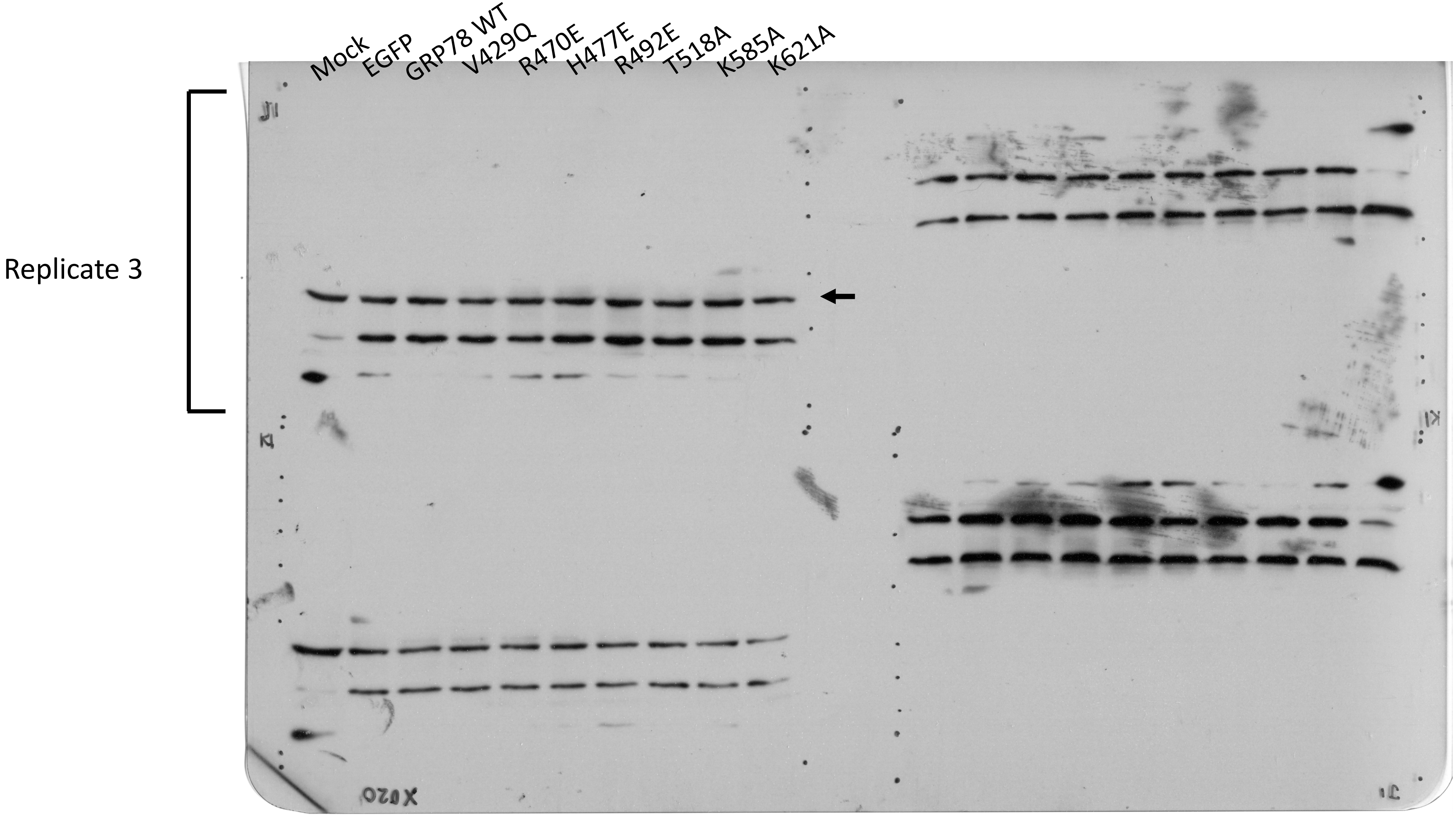

Figure 2B: Output ZIKV E

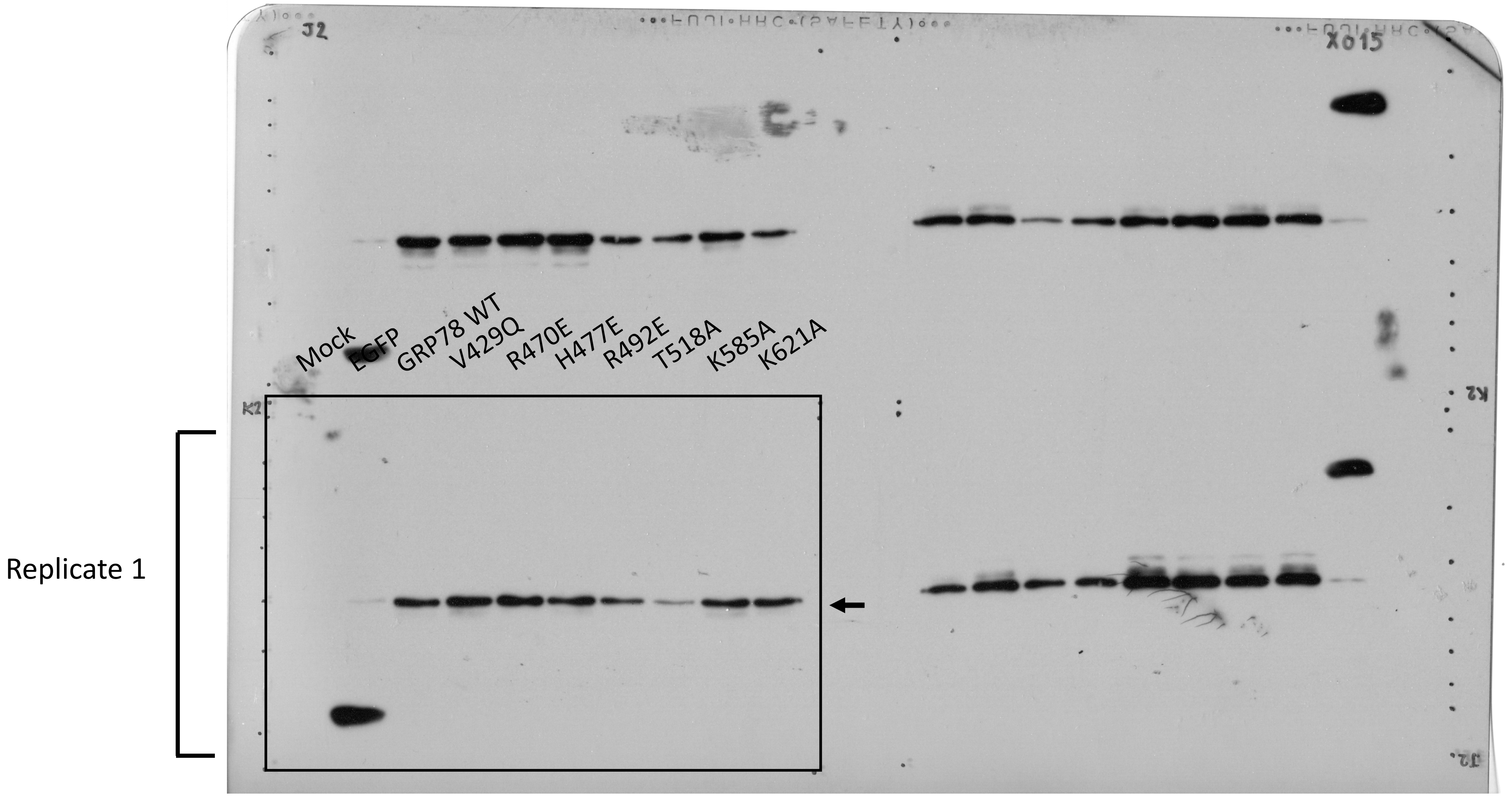

Output ZIKV E replicate 2 of Figure 2B

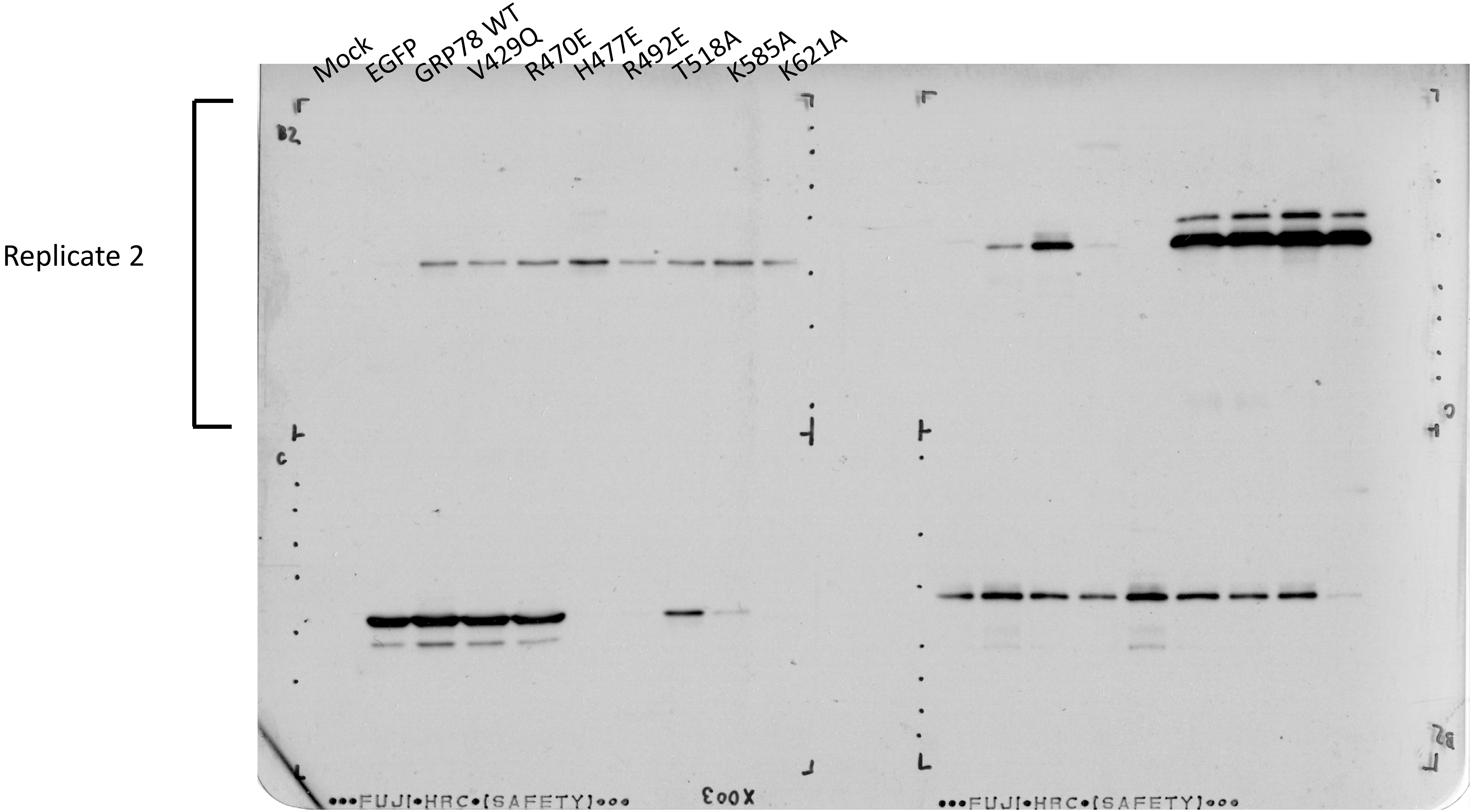

Output ZIKV E replicate 3 of Figure 2B

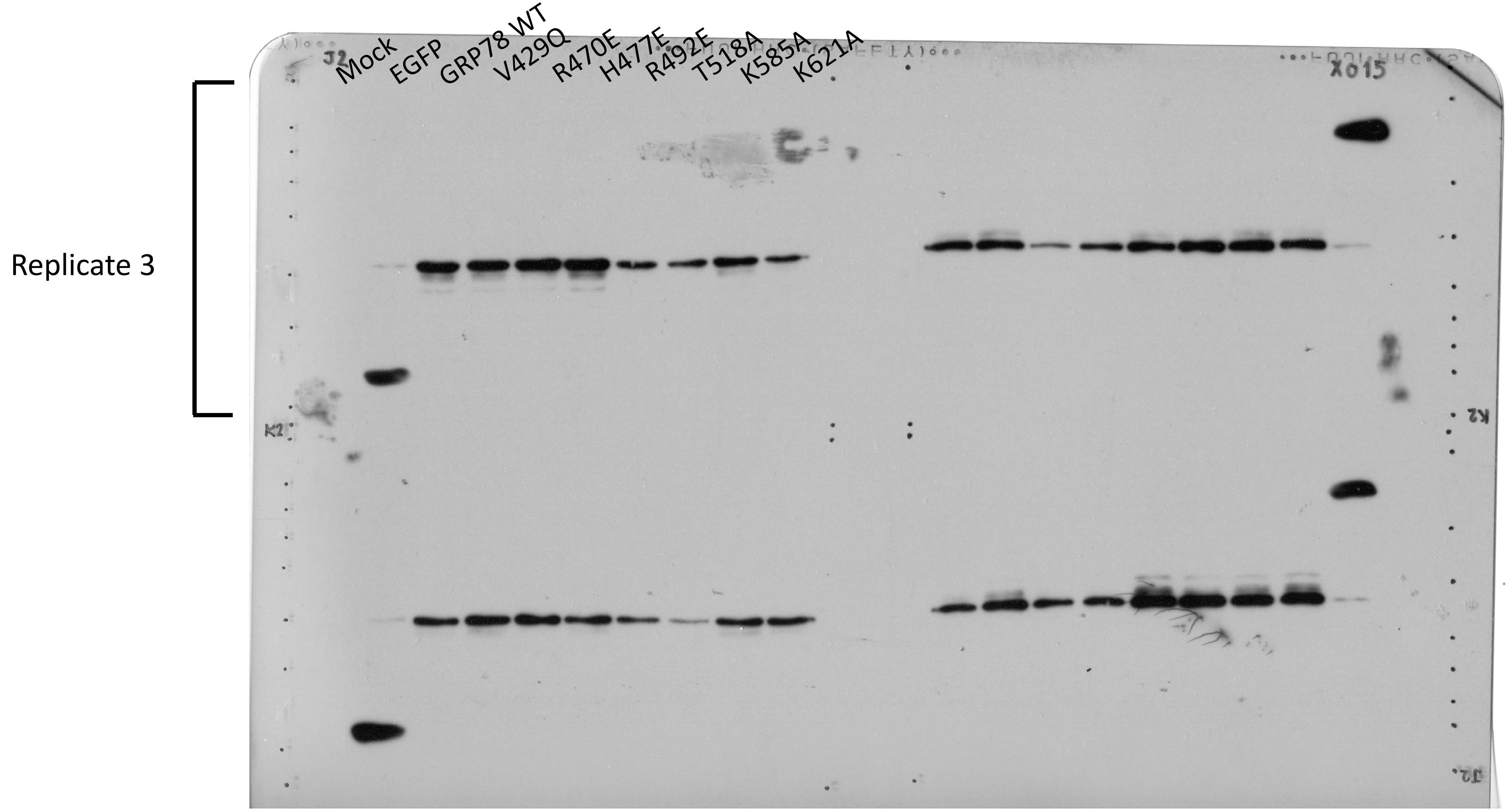

Figure 2B: Output ATF6

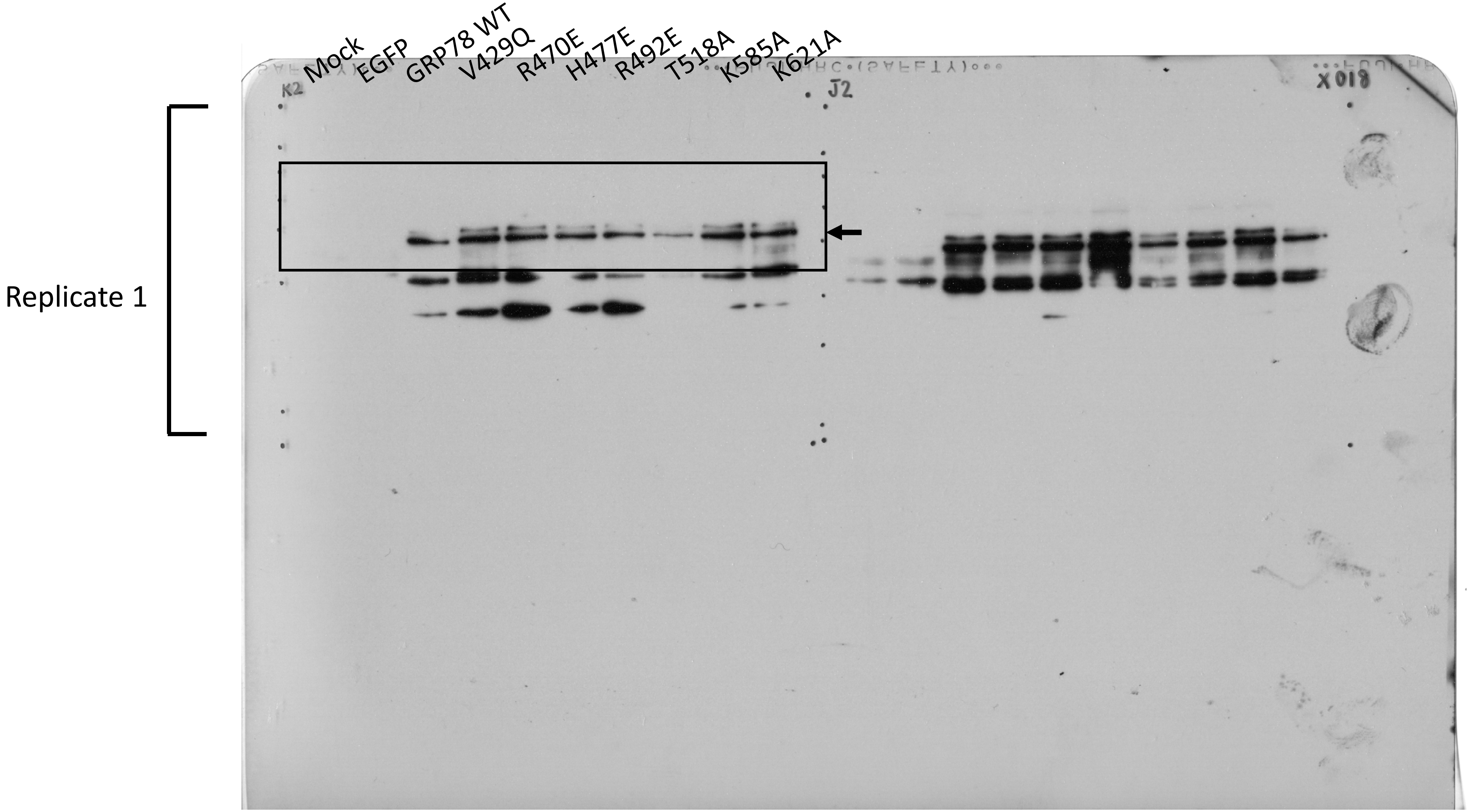

Output ATF6 replicate 2 of Figure 2B

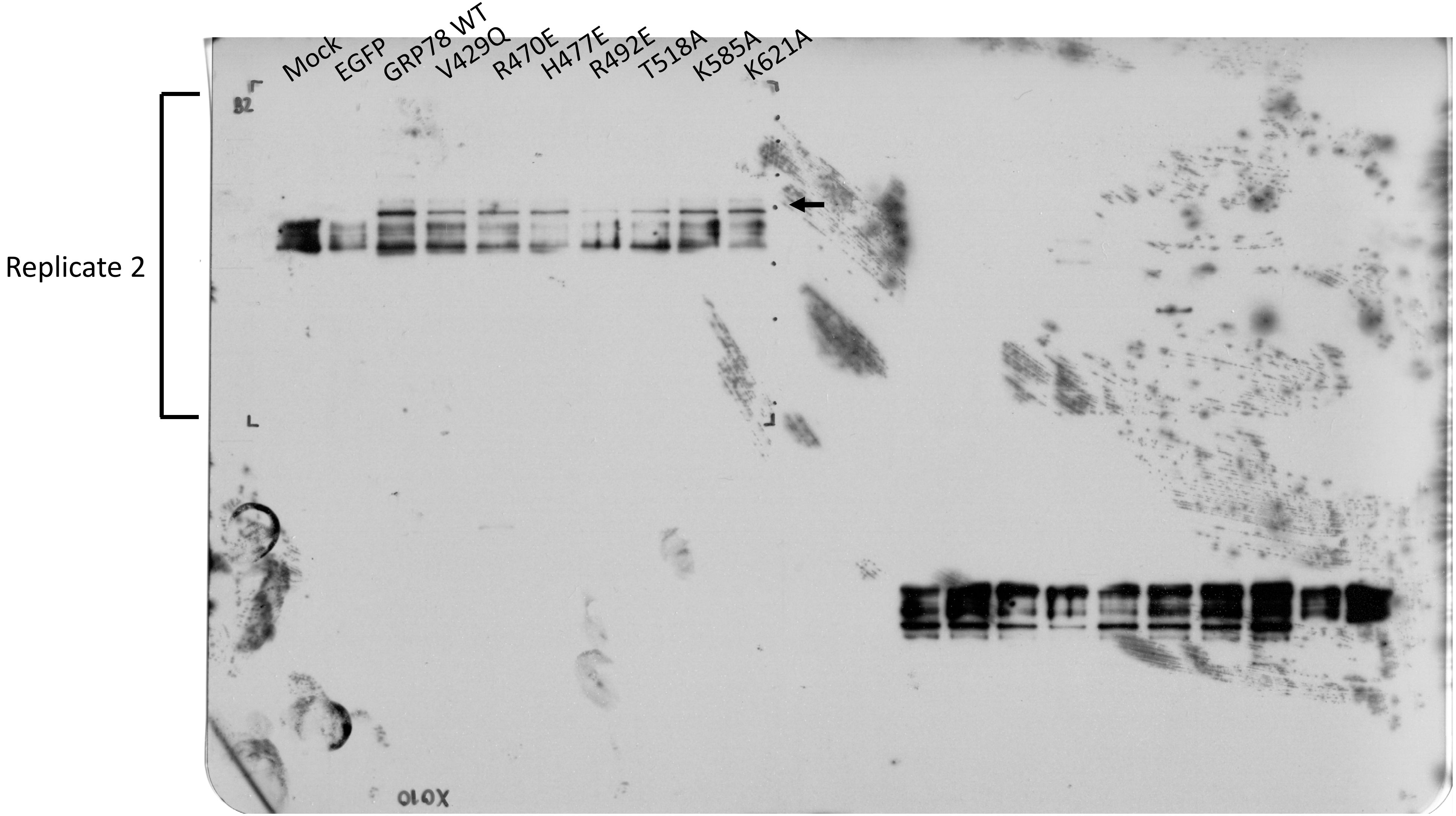

Output ATF6 replicate 3 of Figure 2B

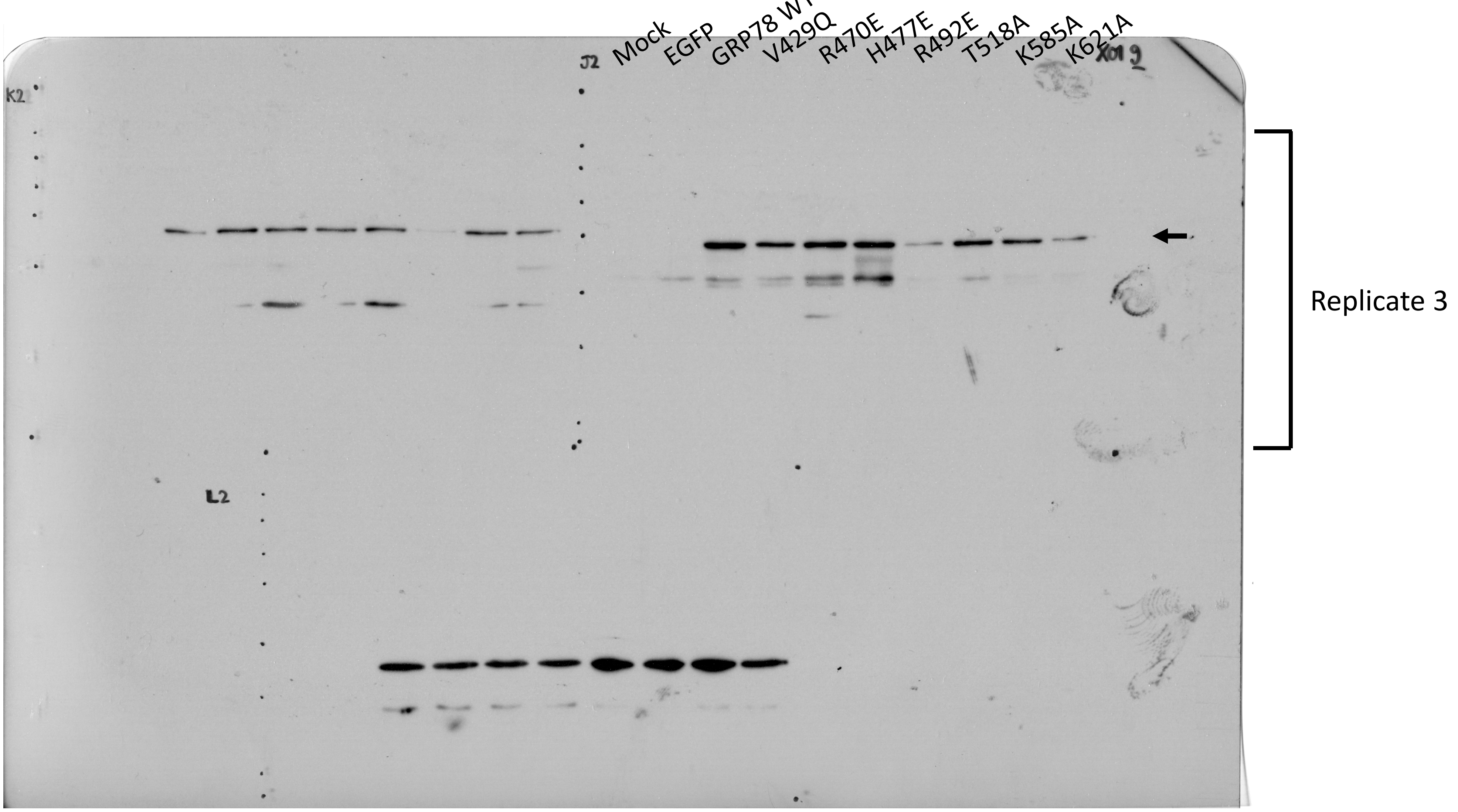

Figure 2B: Output EGFP-GRP78

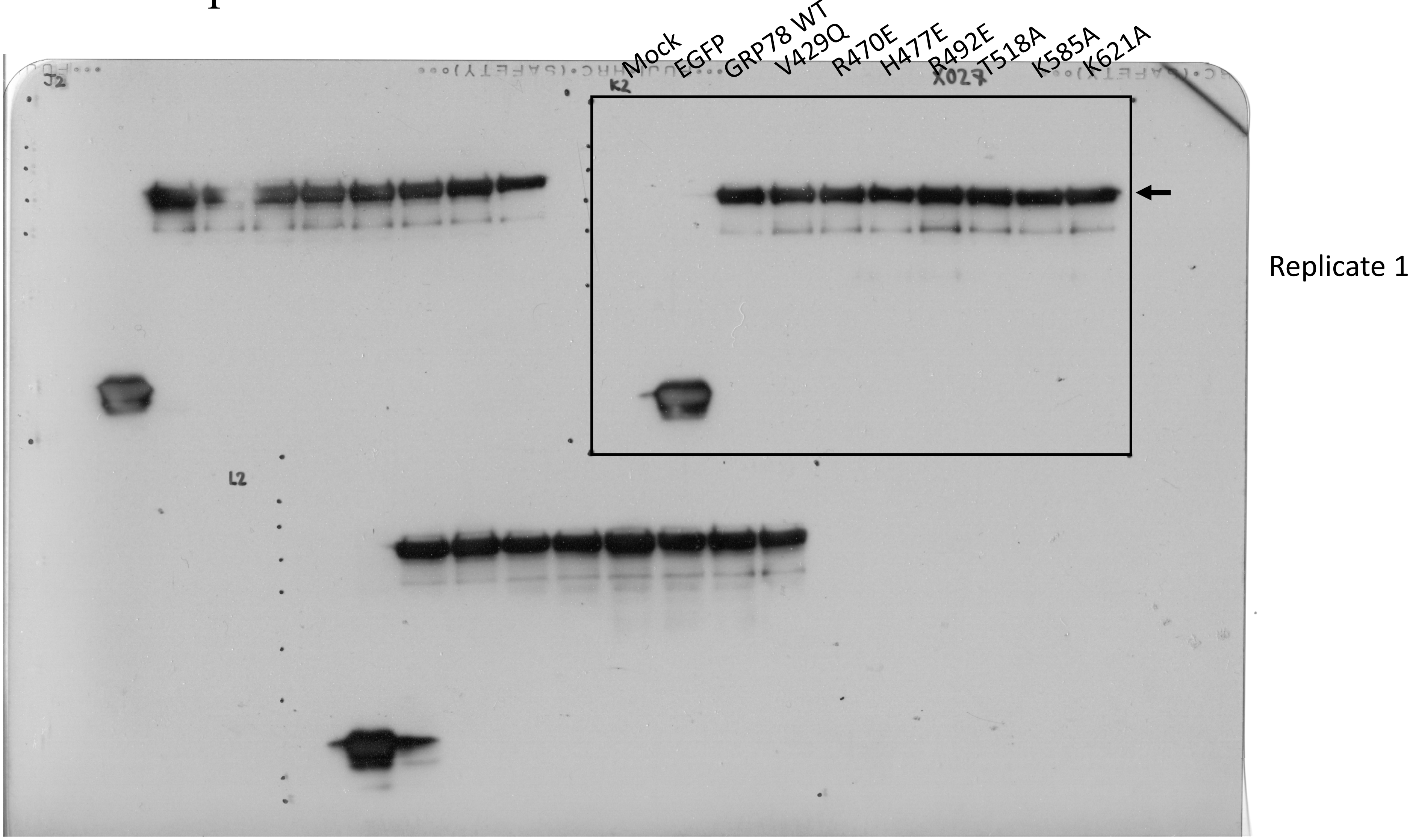

Output EGFP-GRP78 replicate 2 of Figure 2B

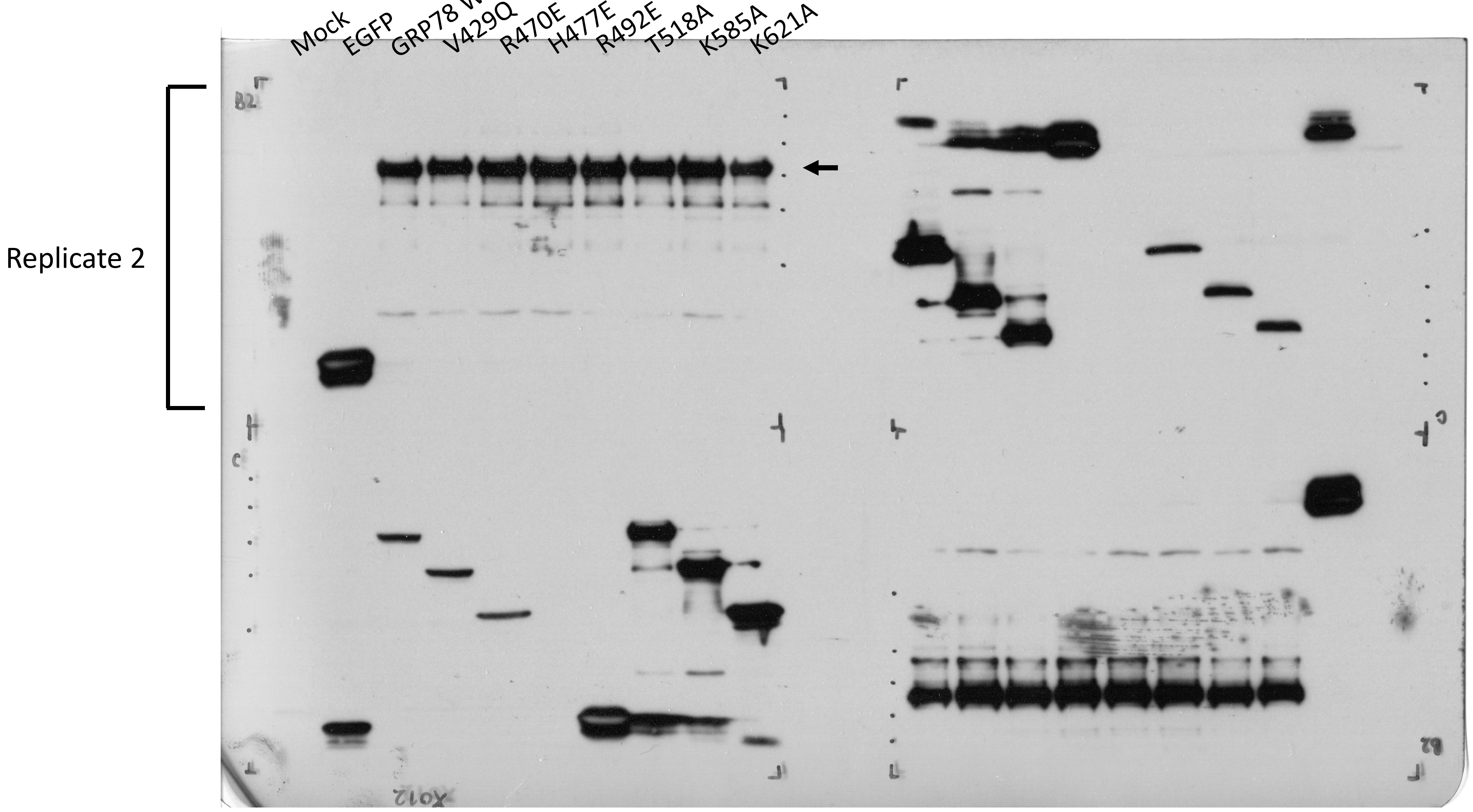

Output EGFP-GRP78 replicate 3 of Figure 2B

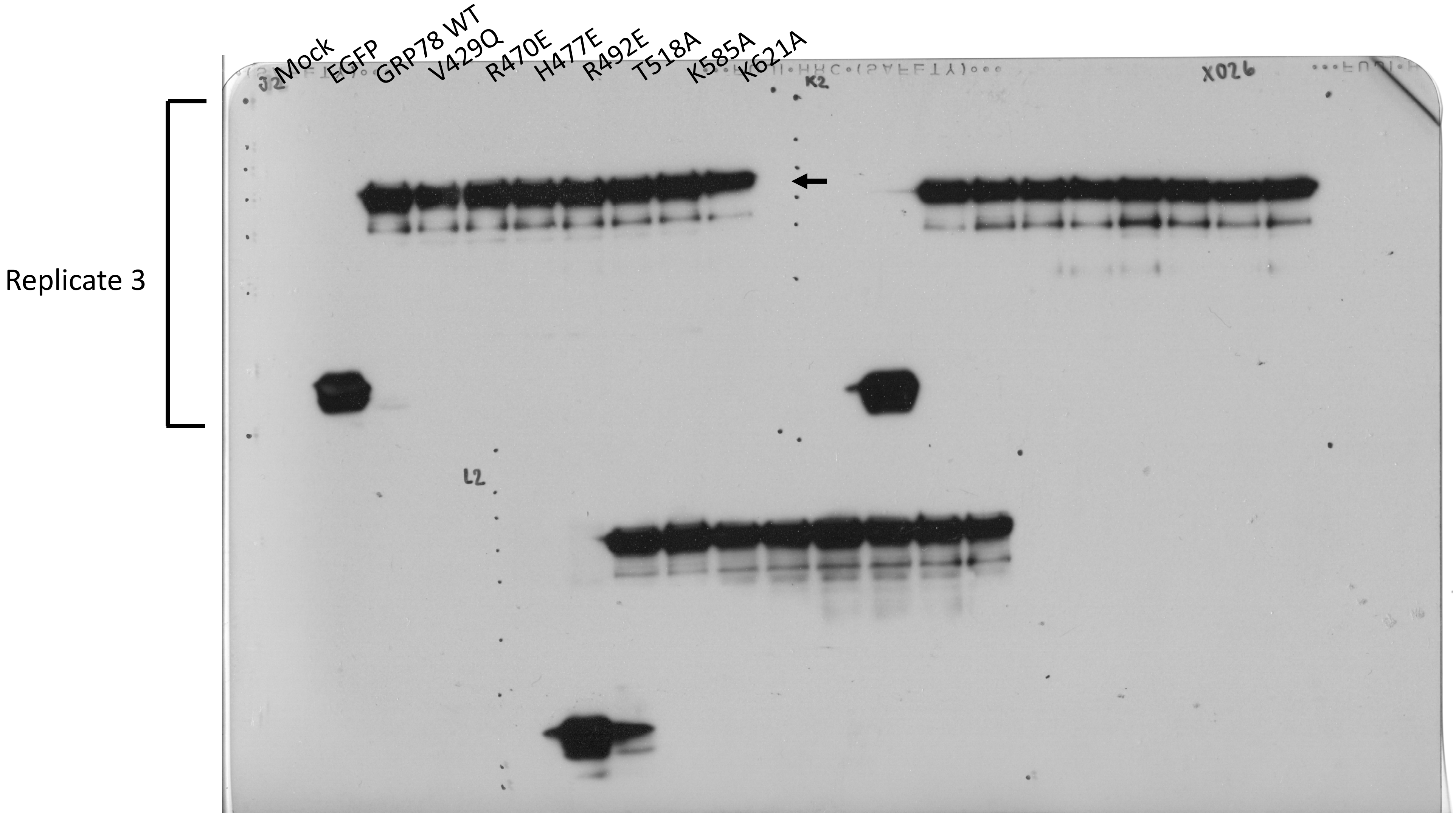

Figure 3B: Input ZIKV NS1

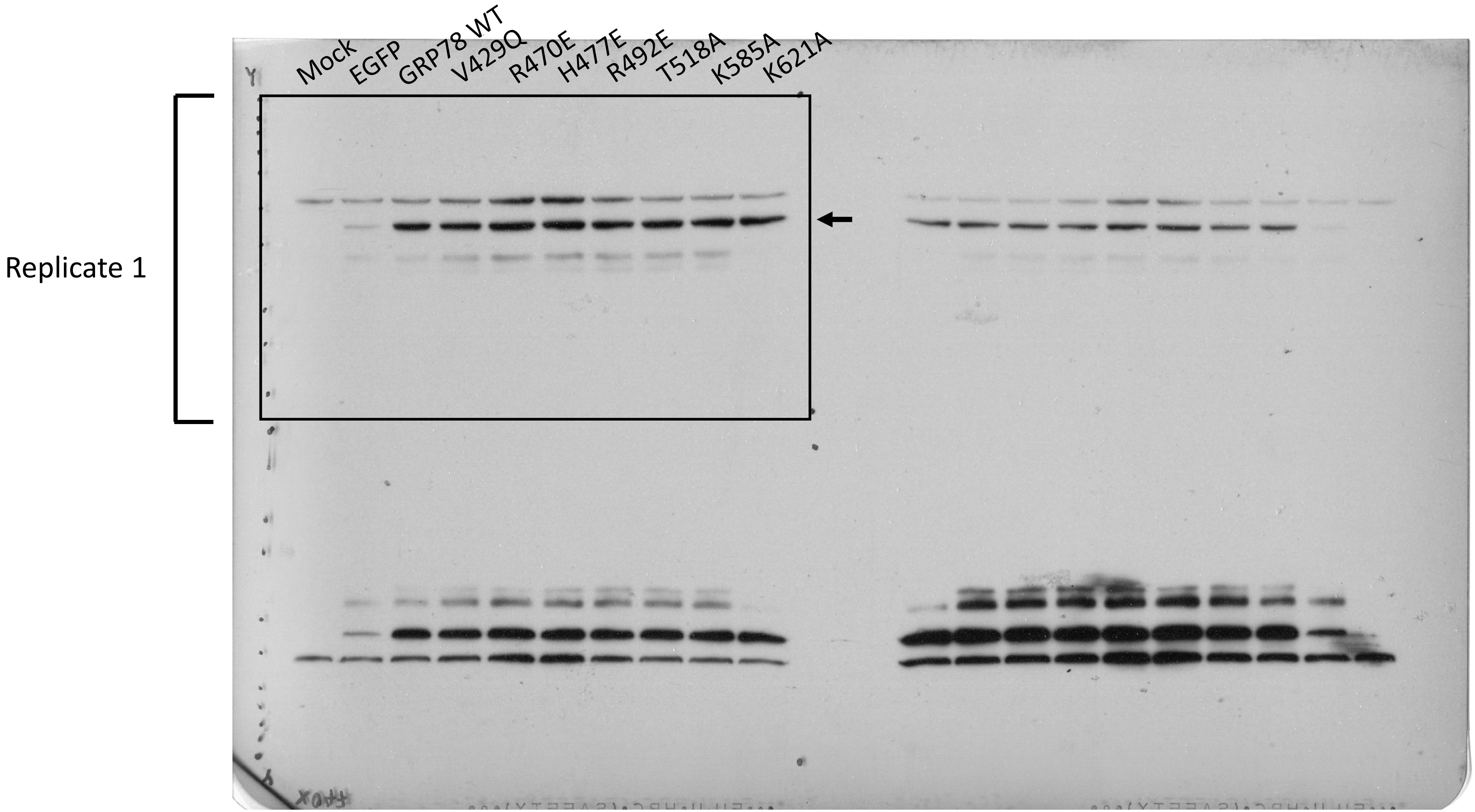

Input ZIKV NS1 replicate 2 of Figure 2C

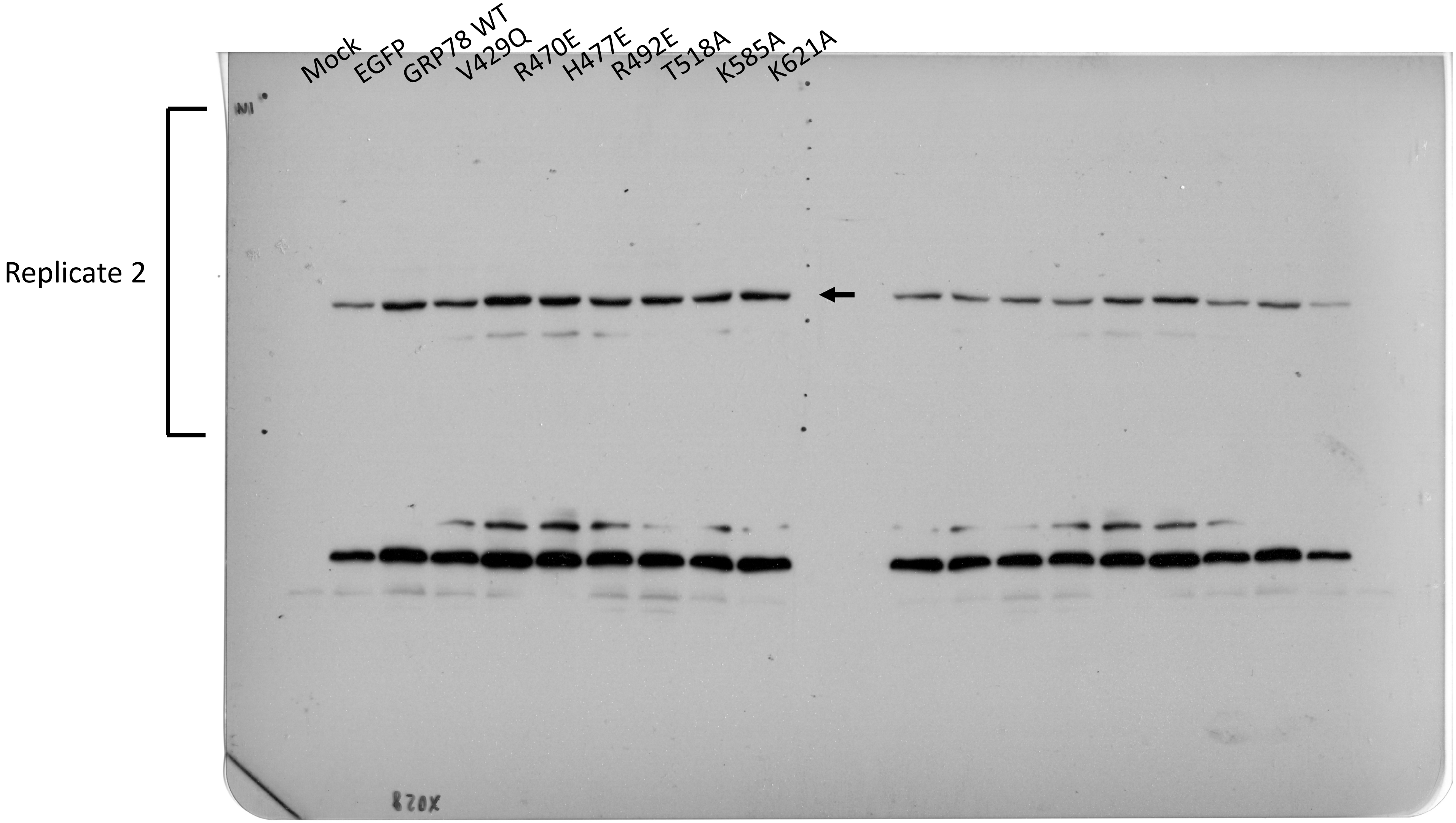

Input ZIKV NS1 replicate 3 of Figure 2C

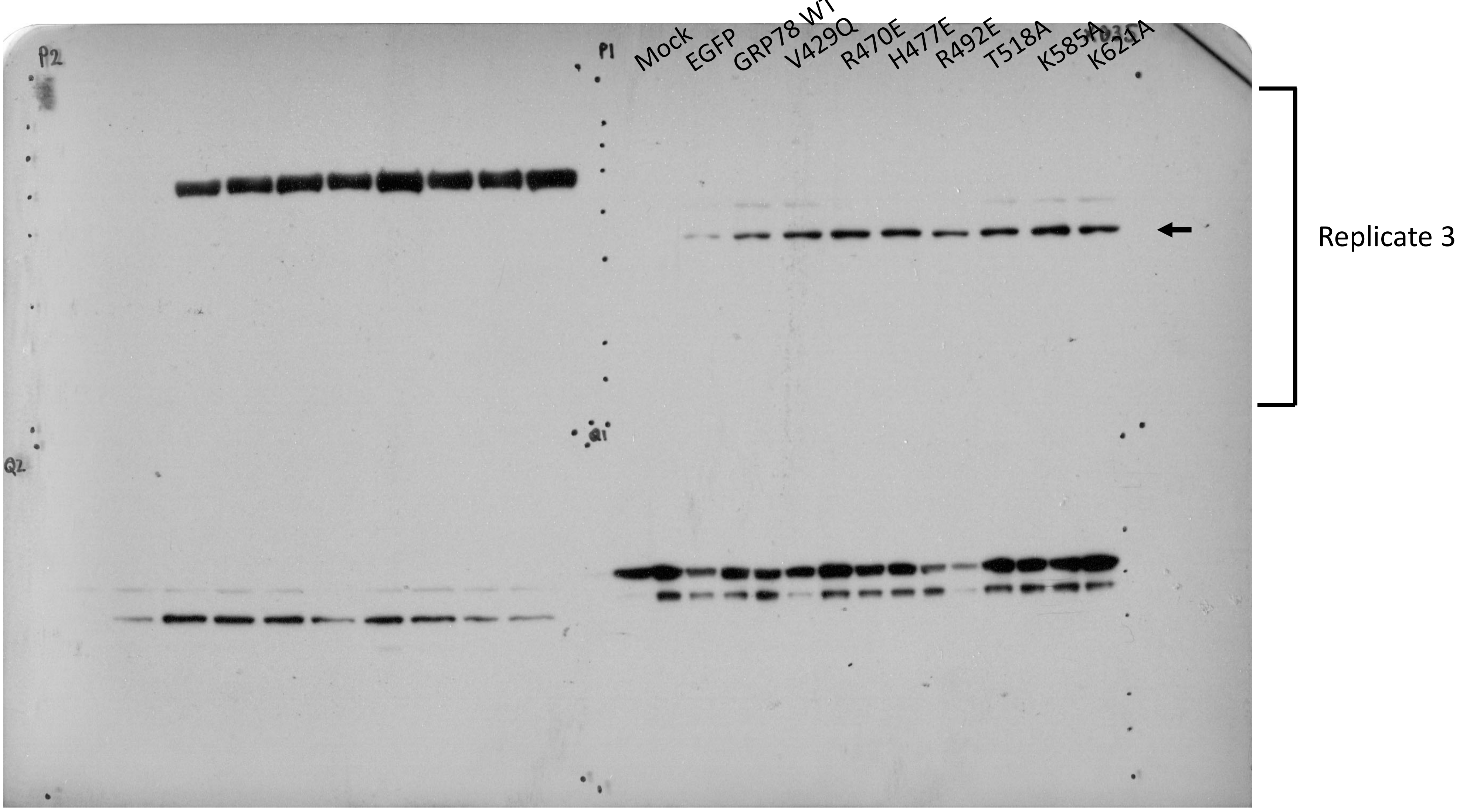

Figure 3B: Input EGFP-GRP78

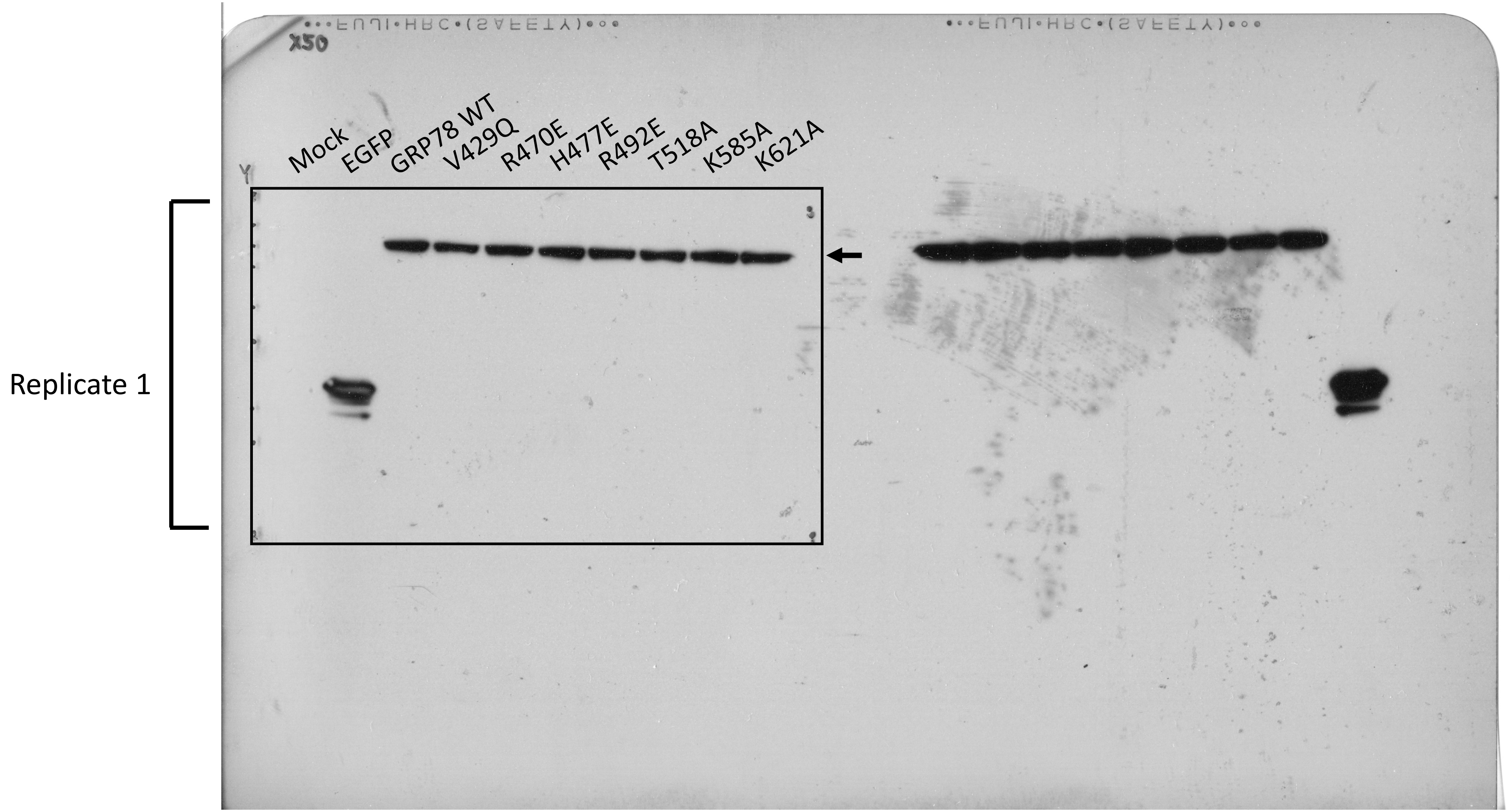

Input EGFP-GRP78 replicate 2 of Figure 2C

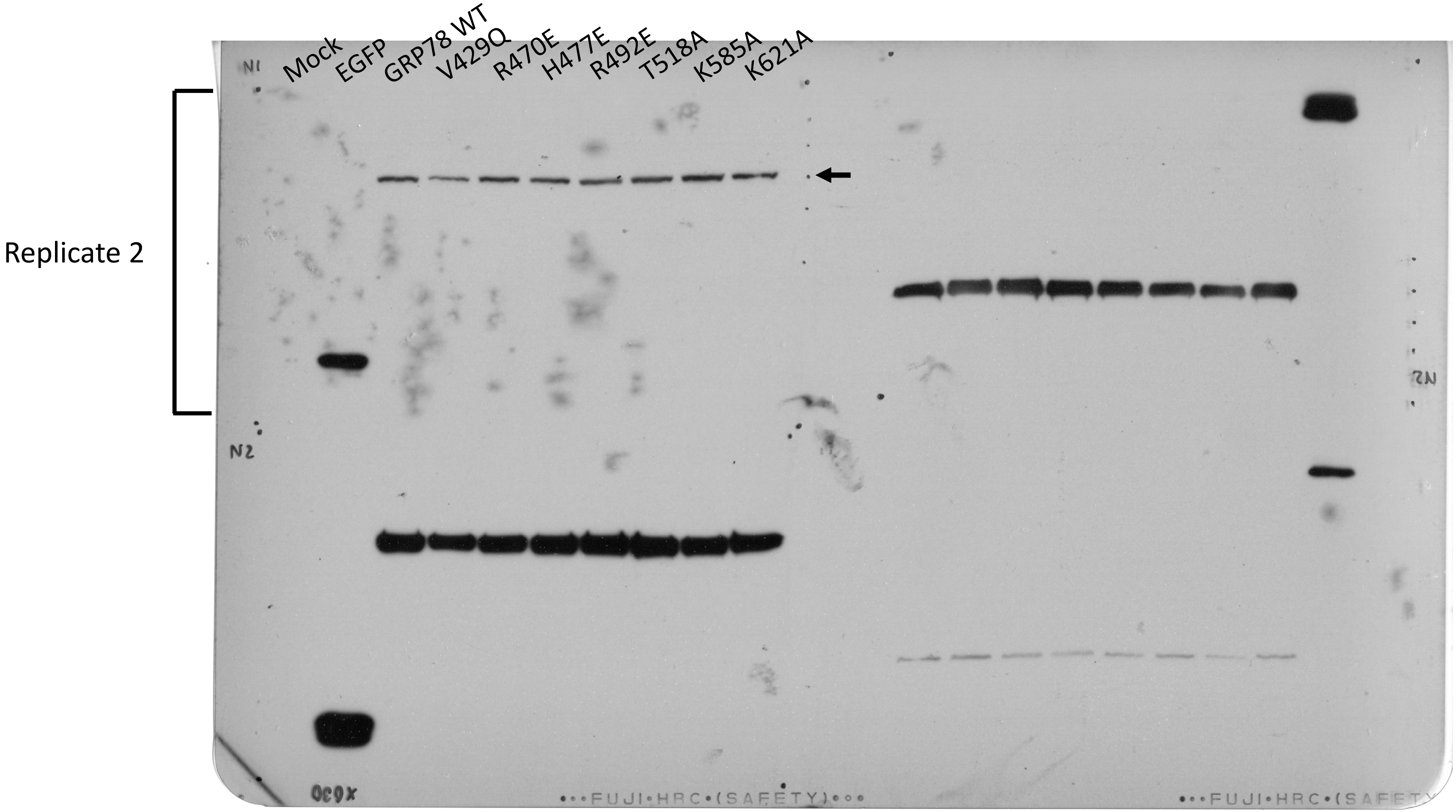

Input EGFP-GRP78 replicate 3 of Figure 2C

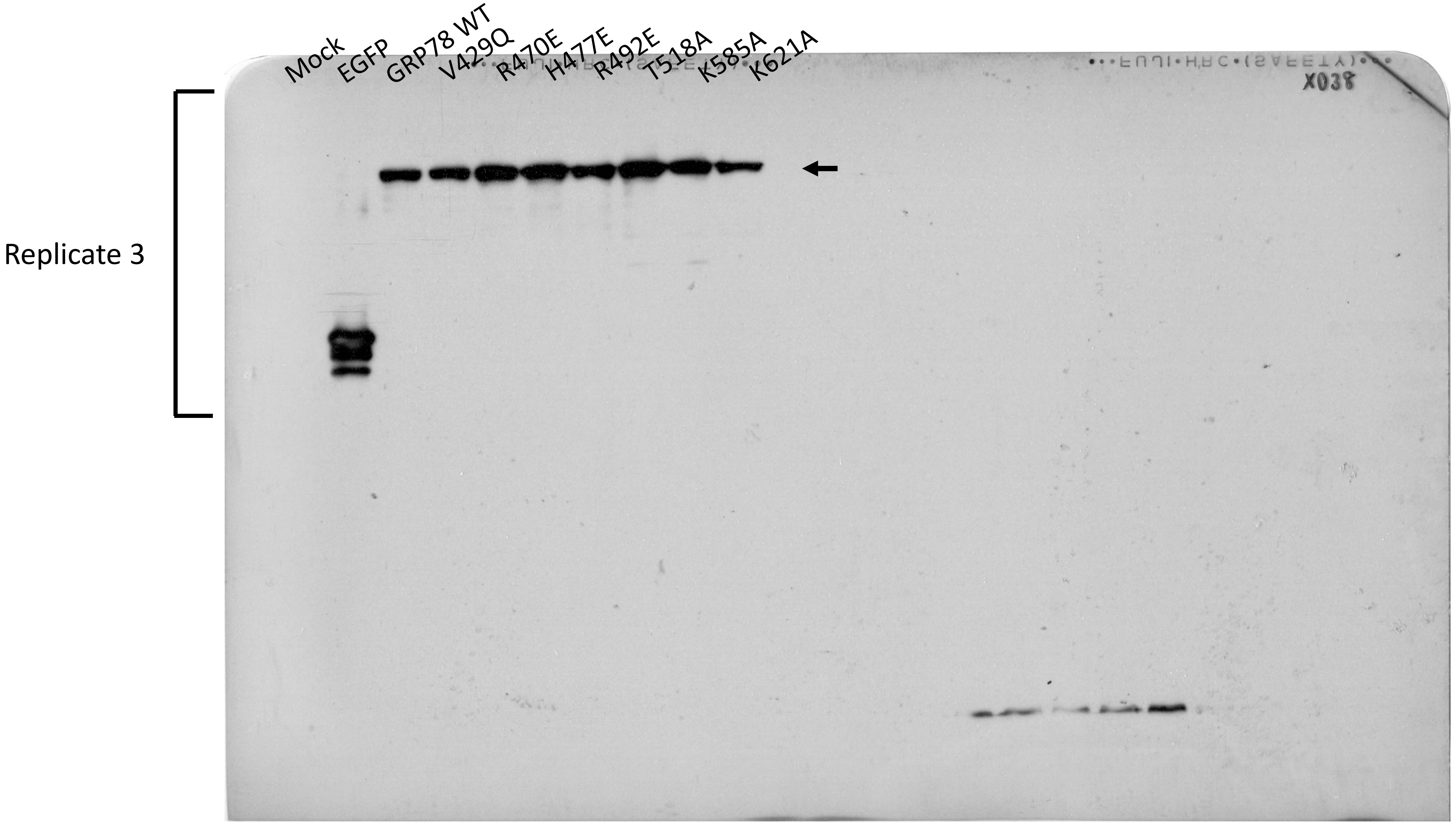

Figure 2C: Input Actin

Replicate 1

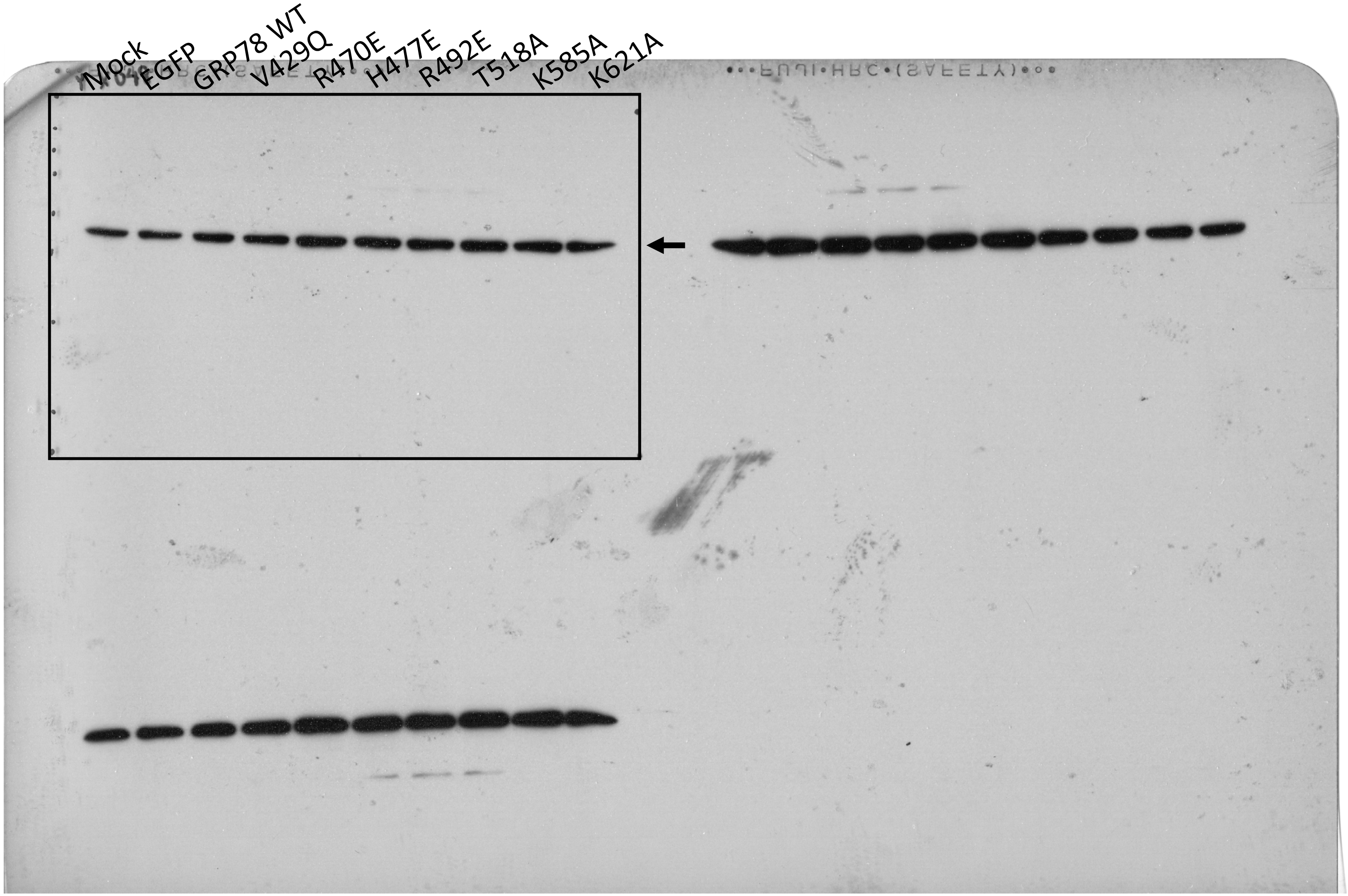

Input actin replicate 2 of Figure 2C

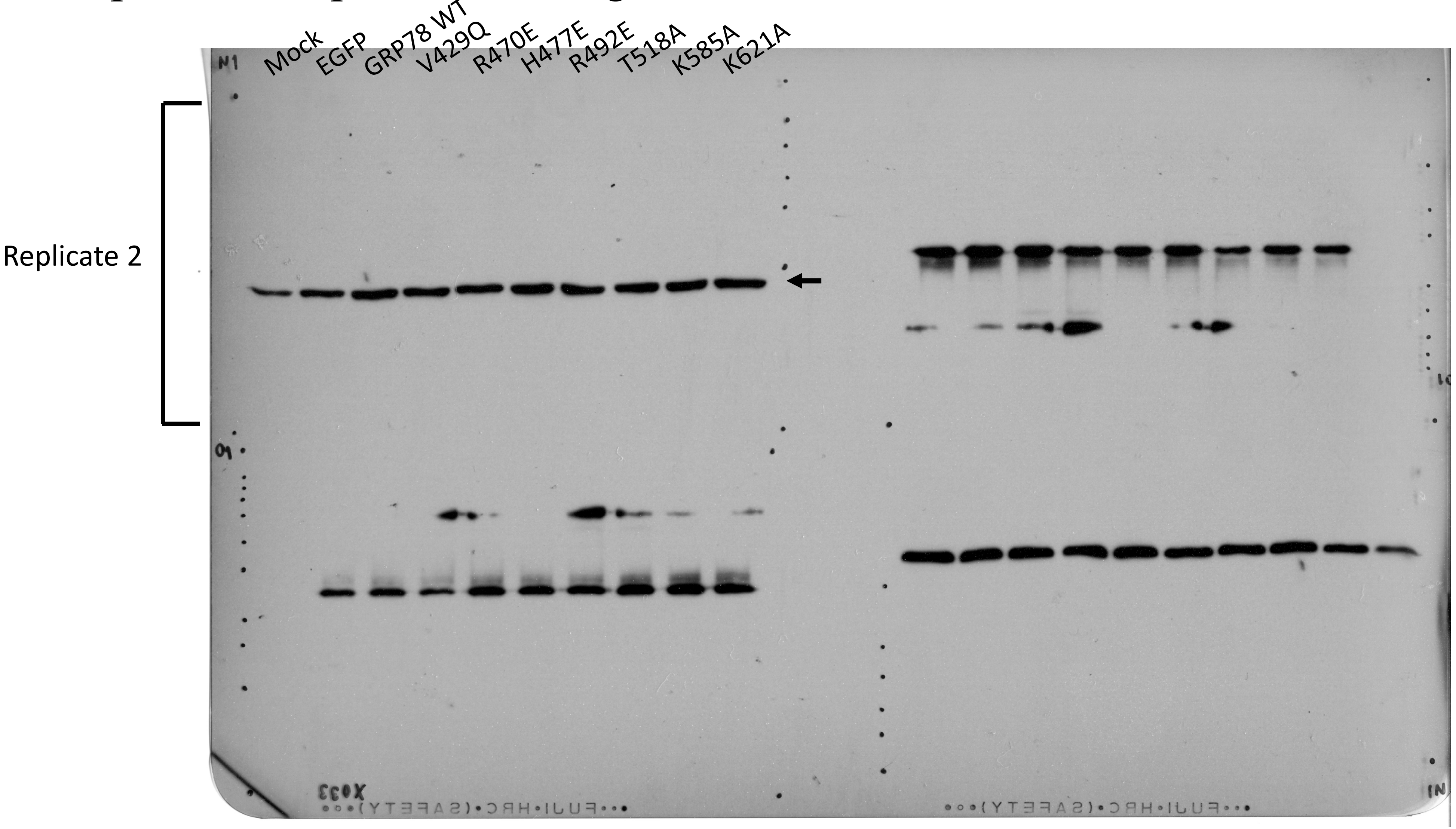

Input actin replicate 3 of Figure 2B

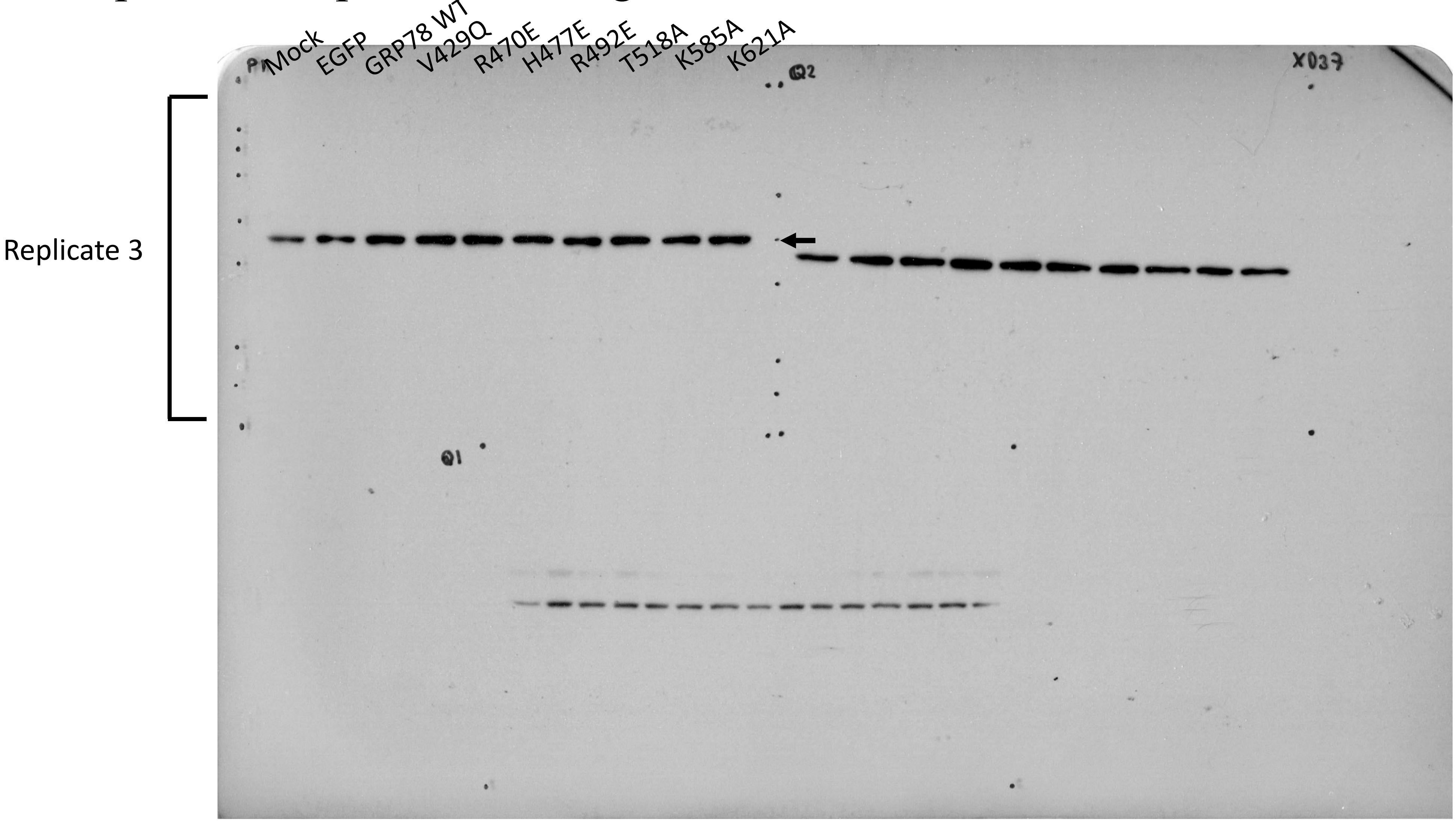

Figure 2C: Output ZIKV NS1

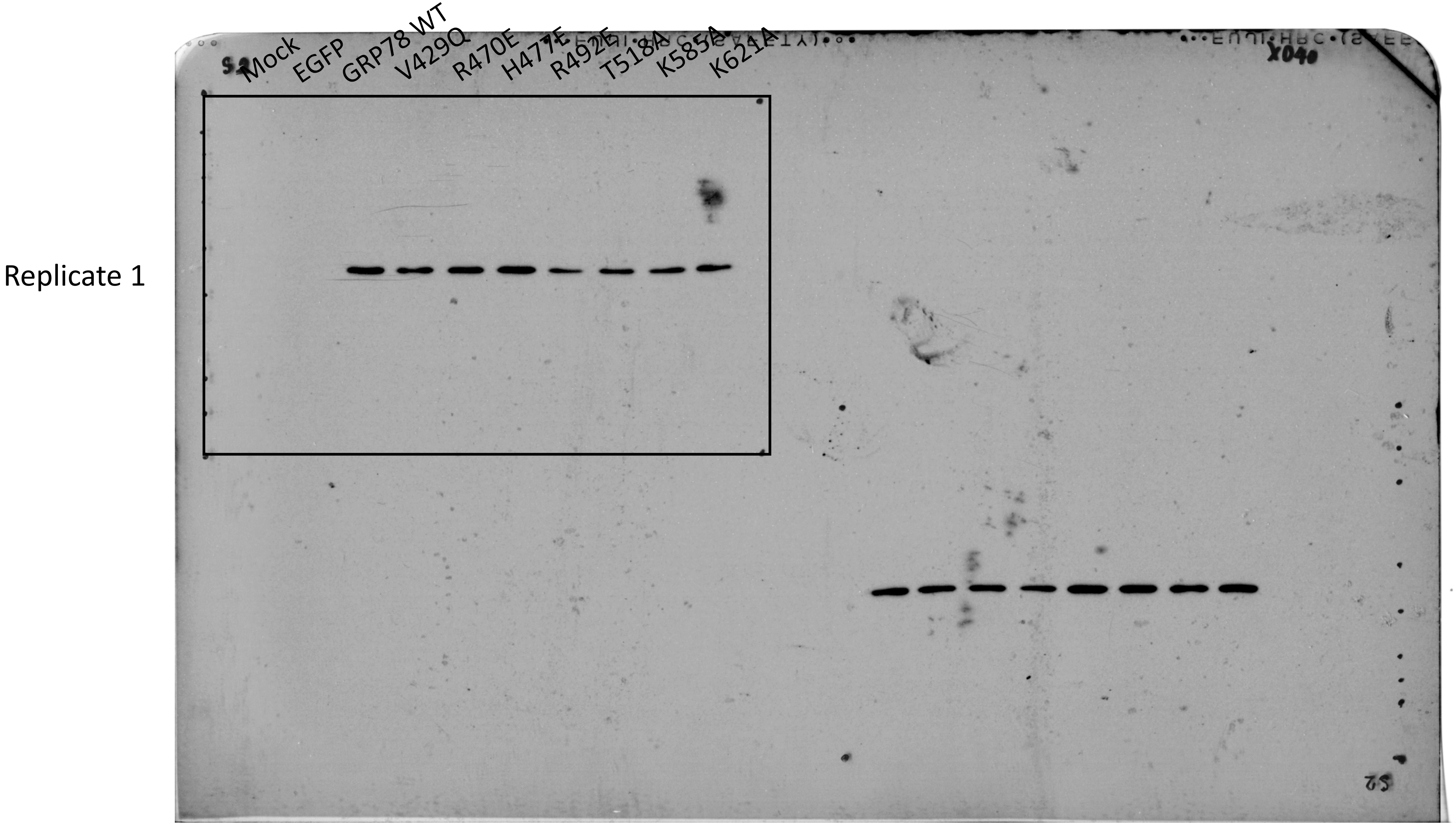

Output ZIKV NS1 replicate 2 of Figure 2C

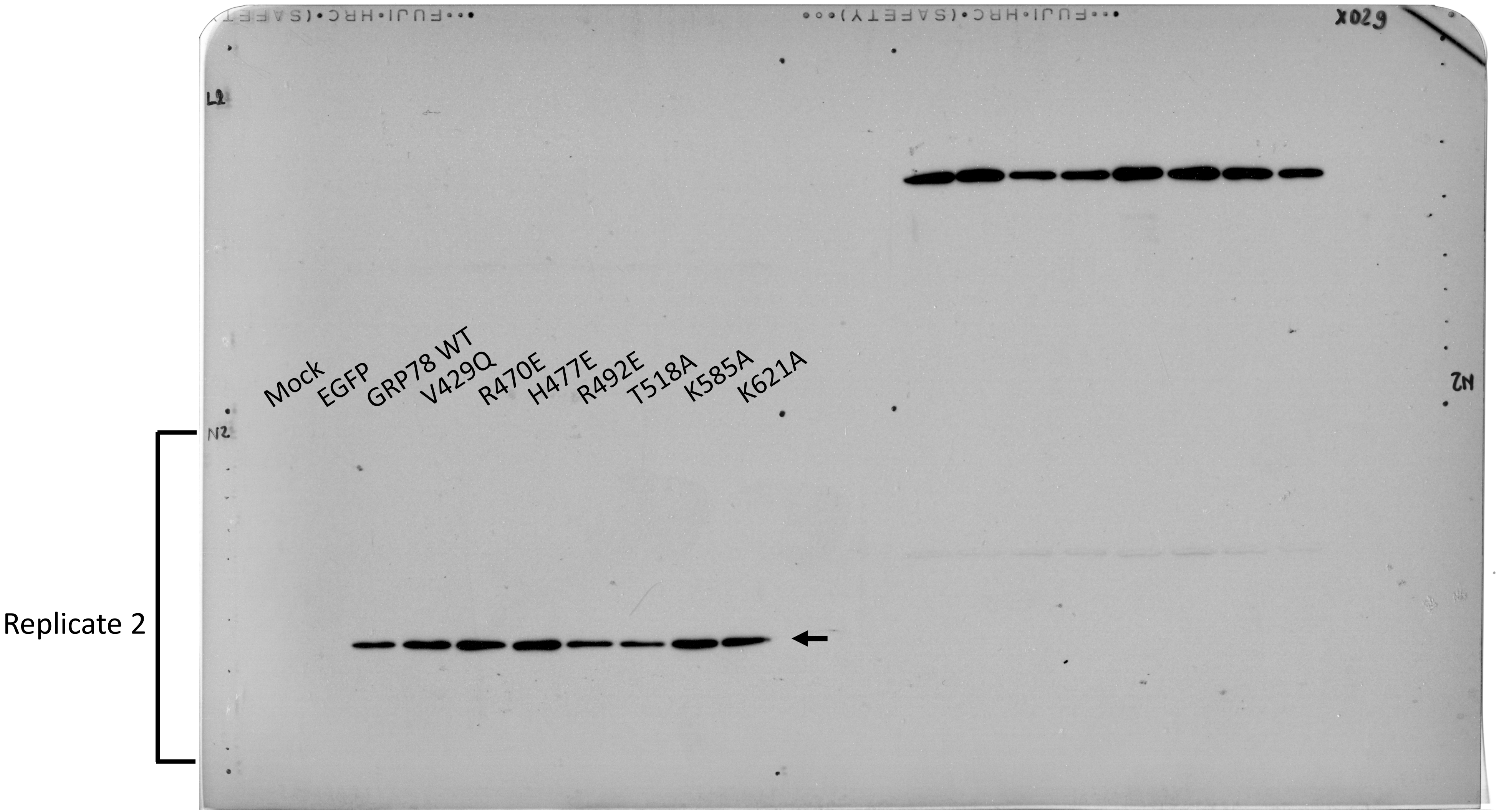

Output ZIKV NS1 replicate 3 of Figure 2C

Replicate 3

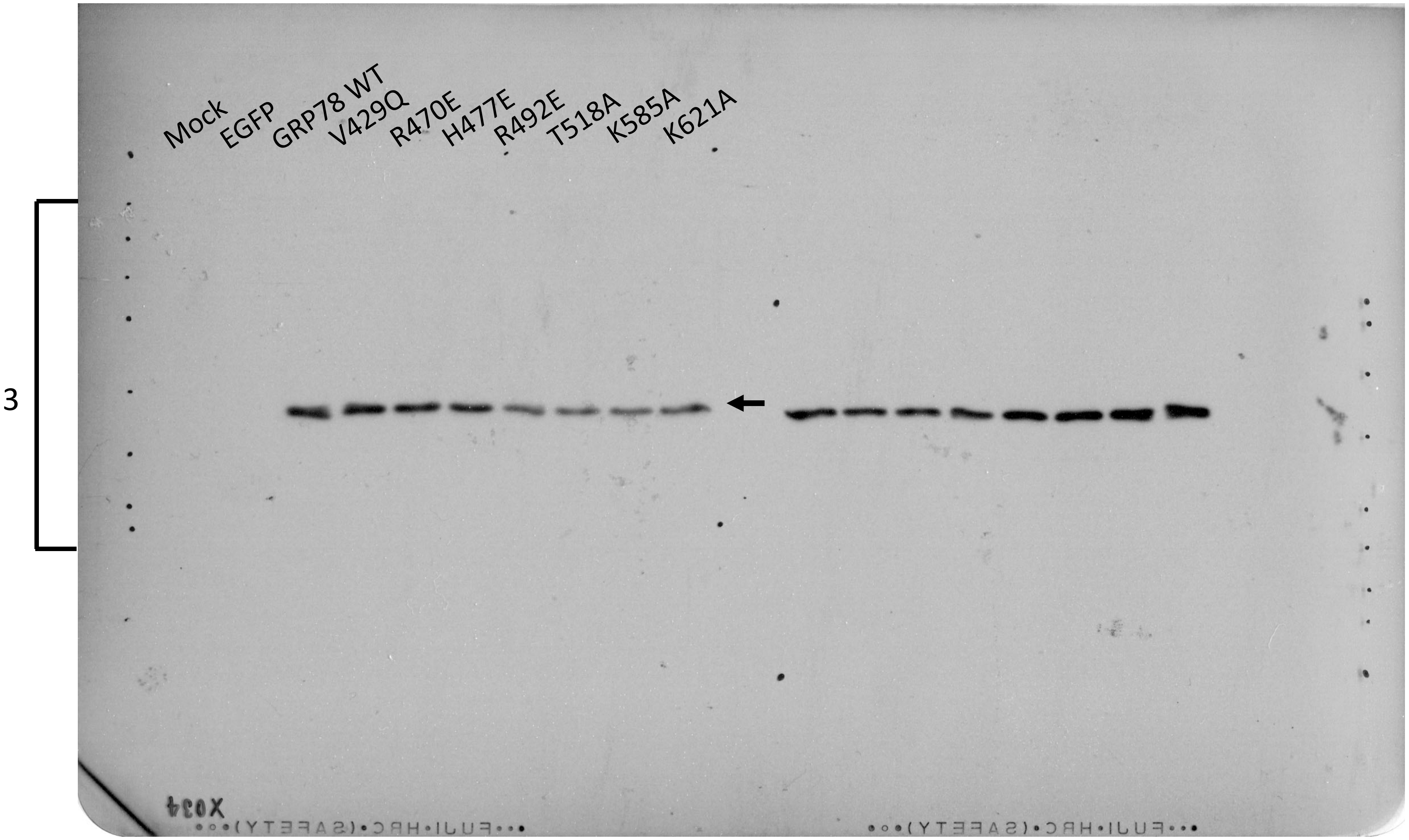

Figure 2C: Output EGFP-GRP78

Replicate 1

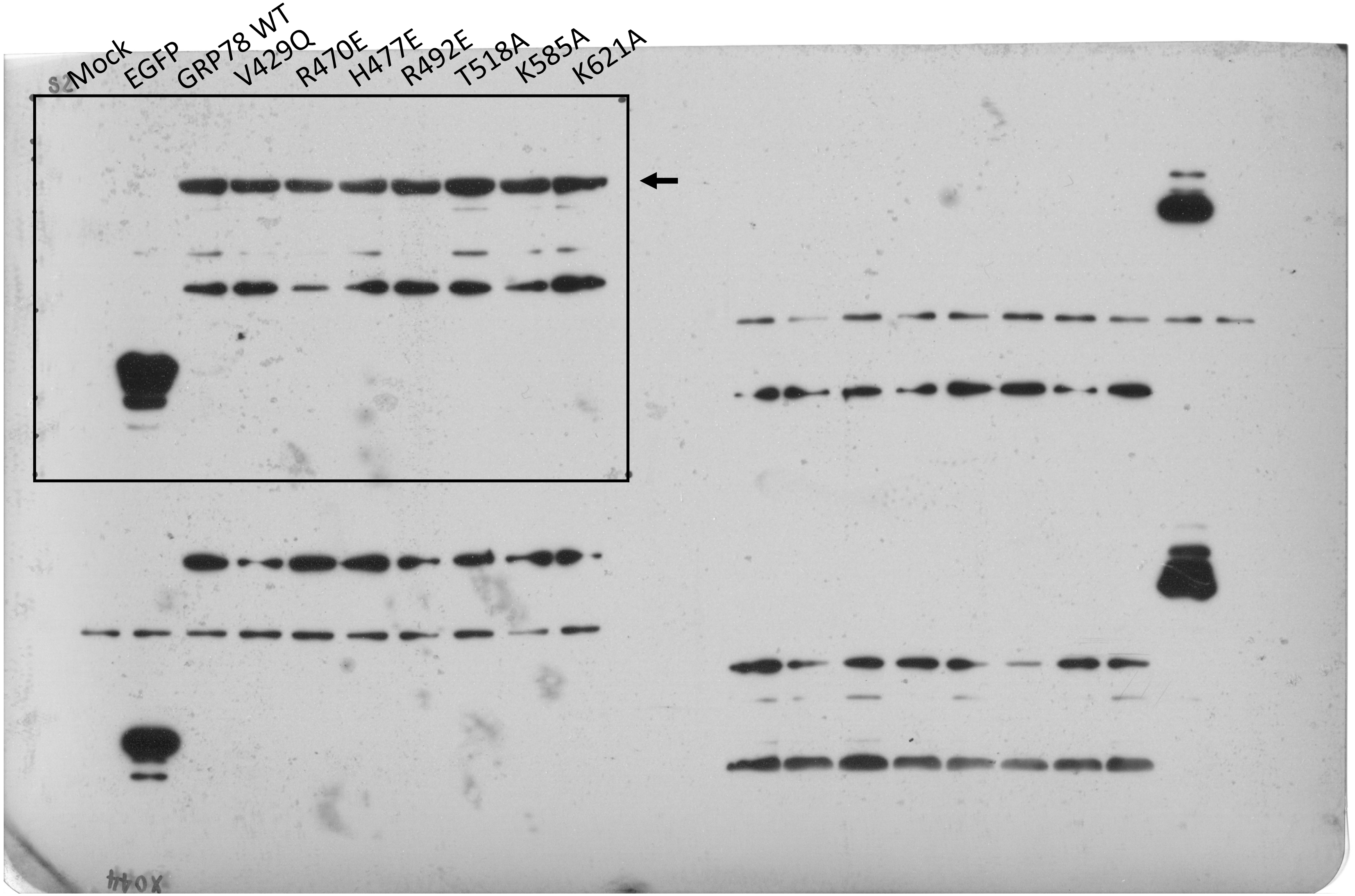

Output EGFP-GRP78 replicate 2 of Figure 2C

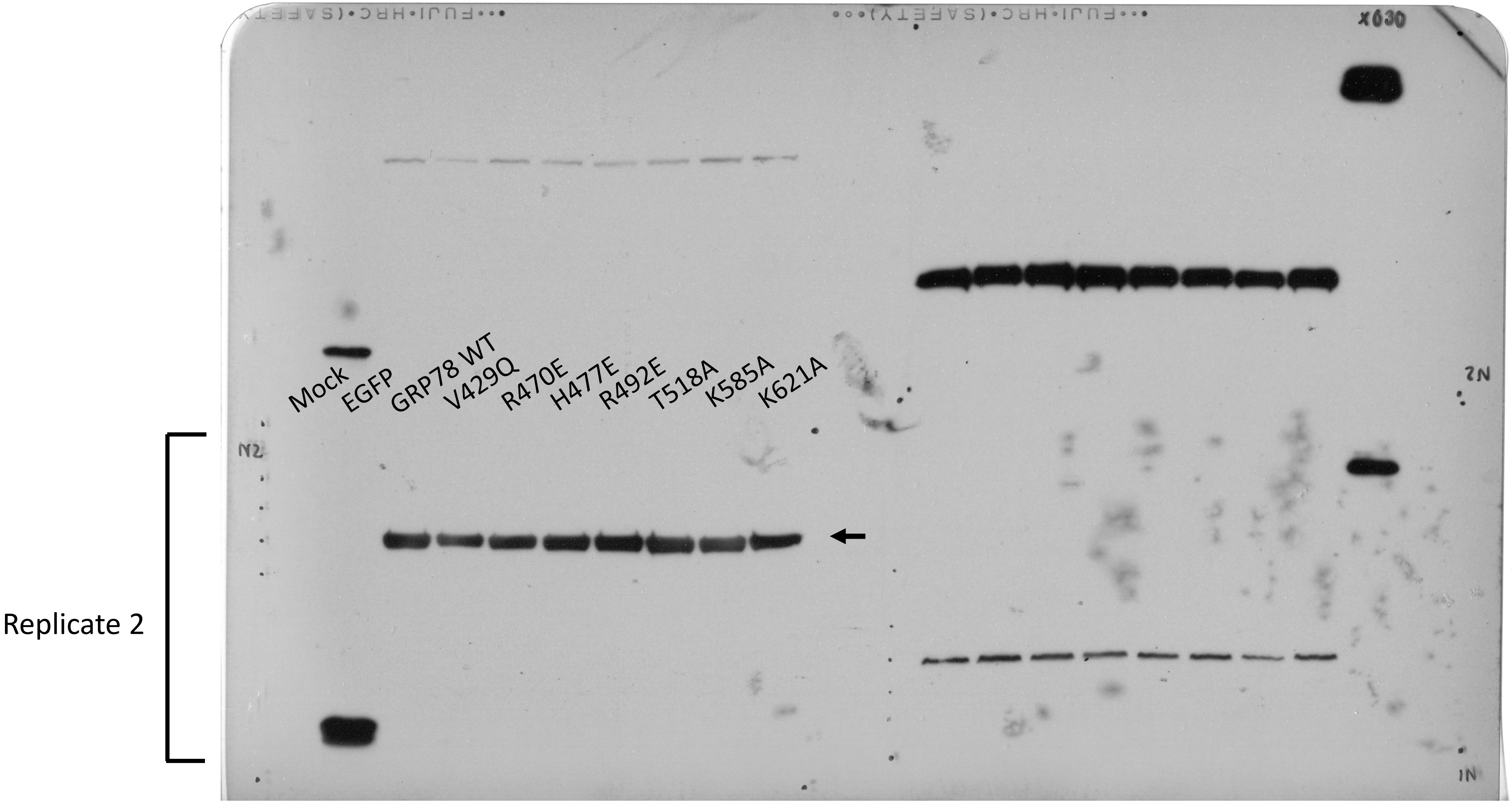

Output EGFP-GRP78 replicate 3 of Figure 2C

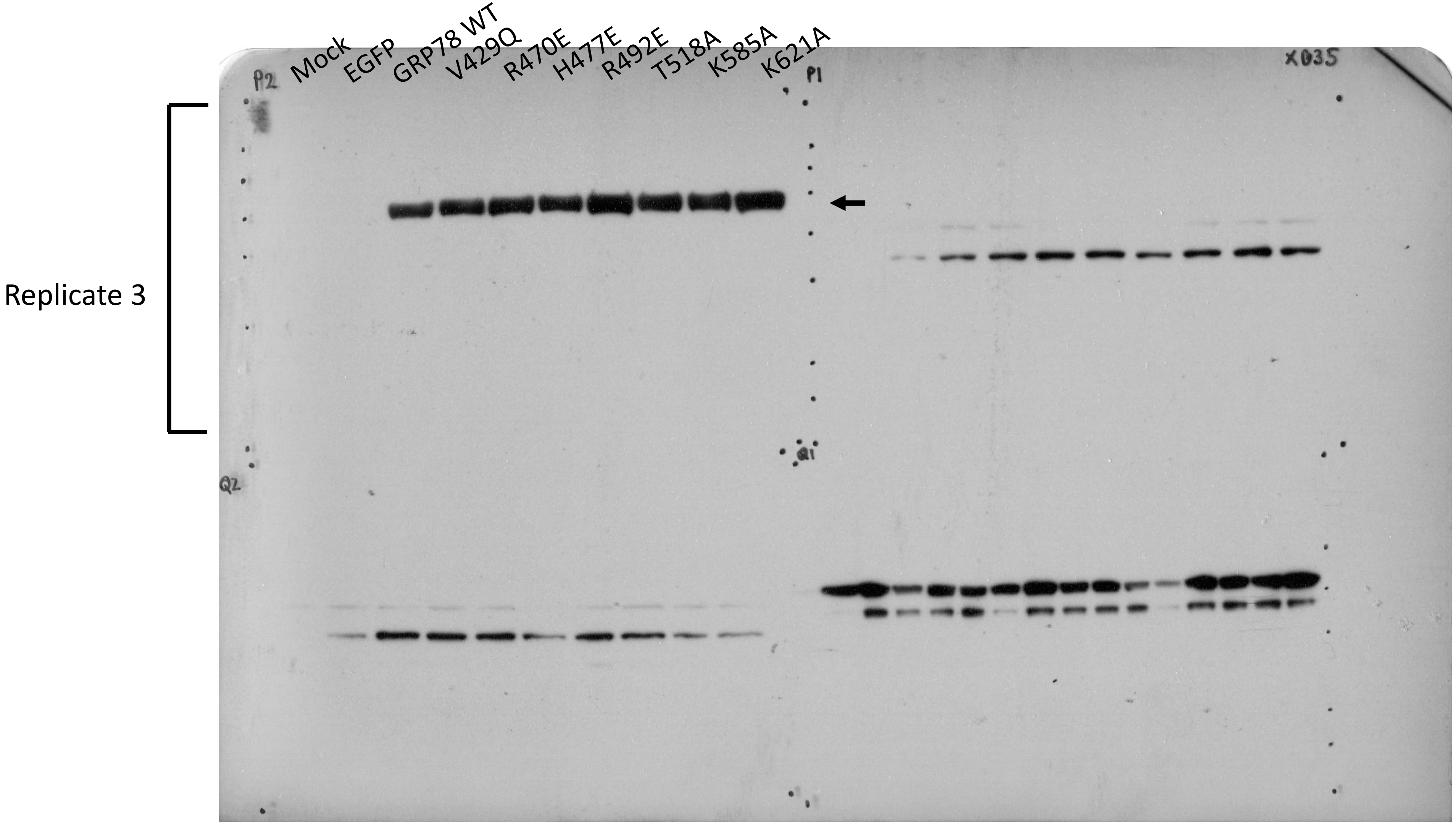

Figure 3A: GRP78-FLAG at 24 h

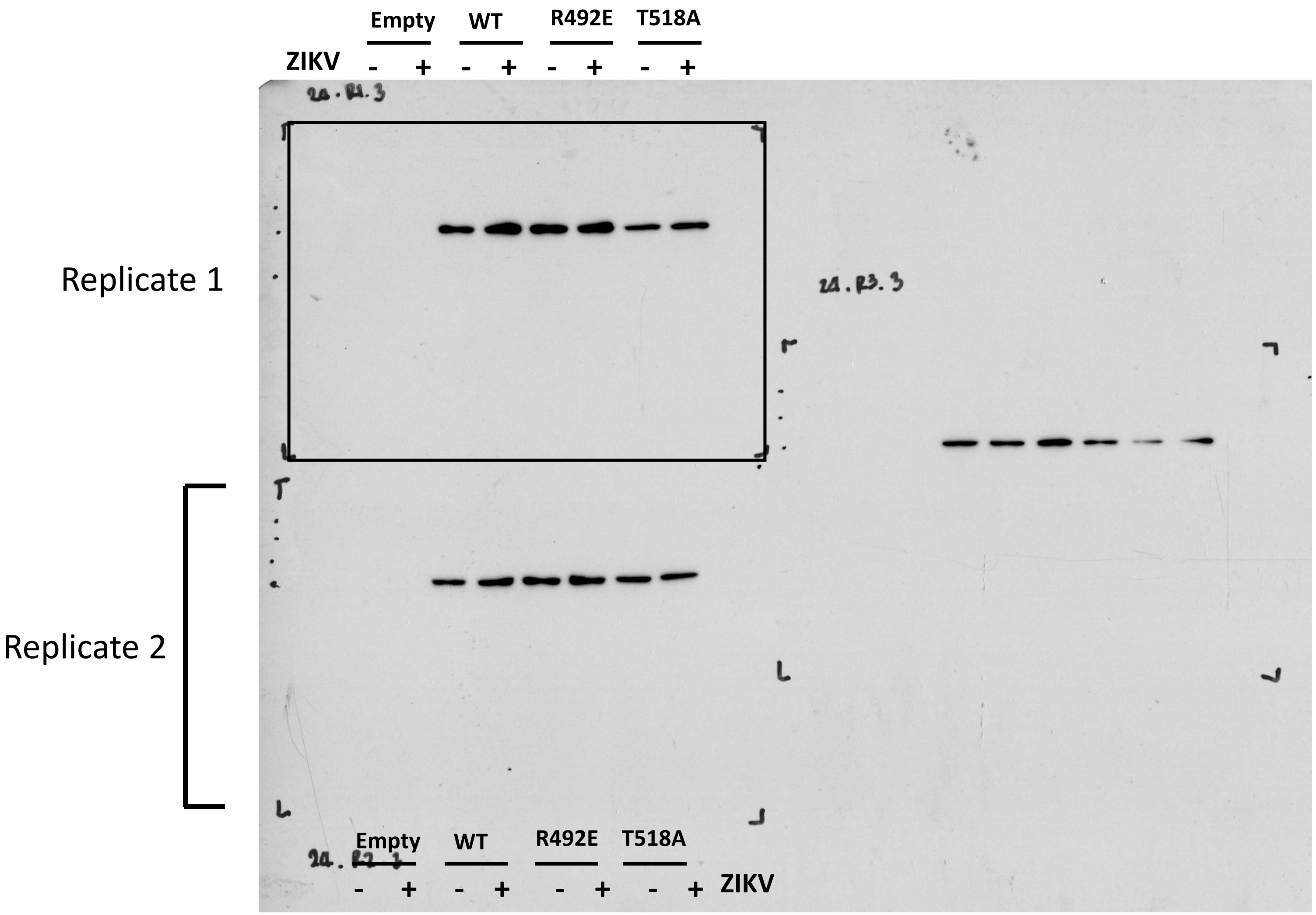

GRP78-FLAG at 24 h replicate 3 of Figure 3A

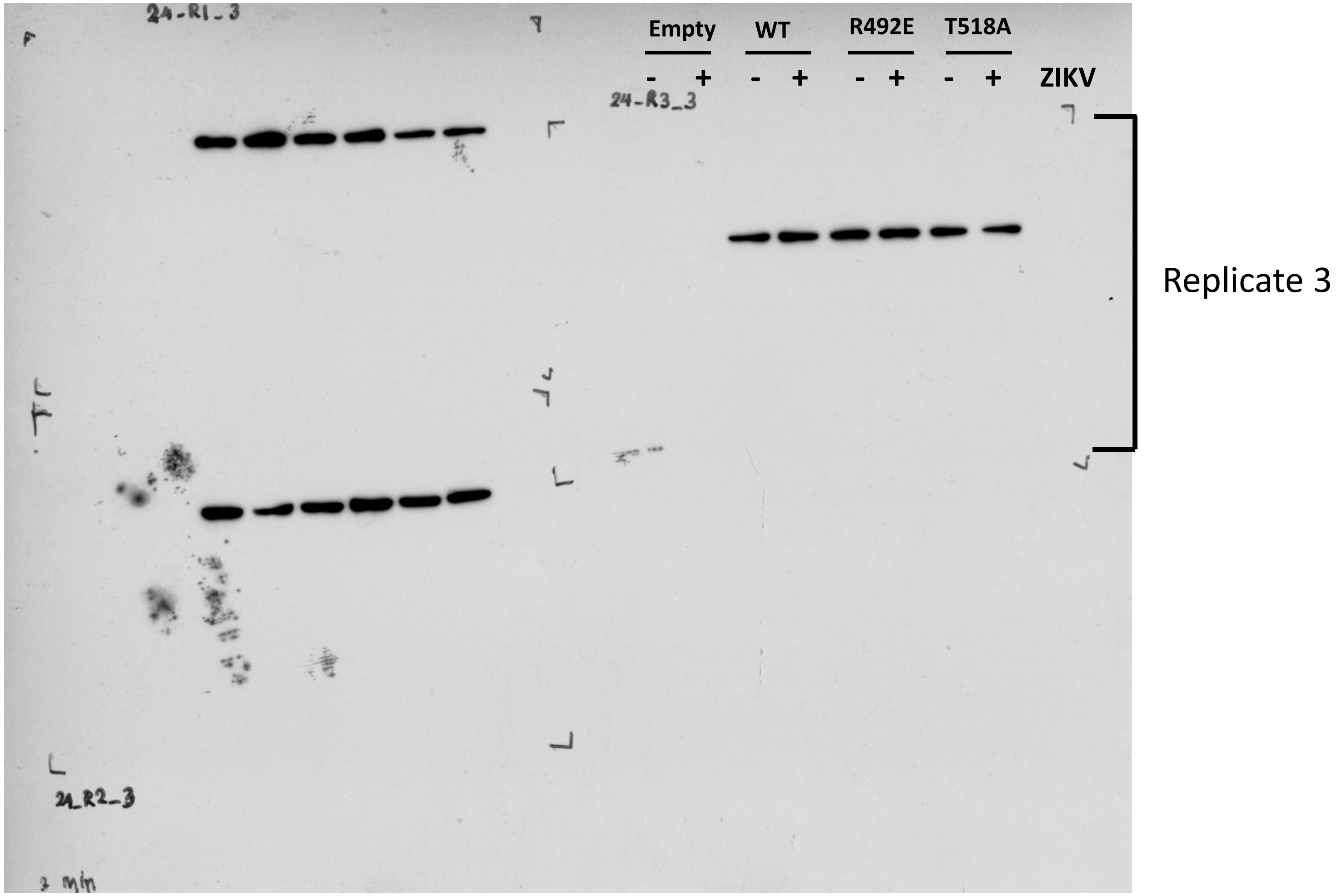

Figure 3A: GRP78-FLAG at 48 h

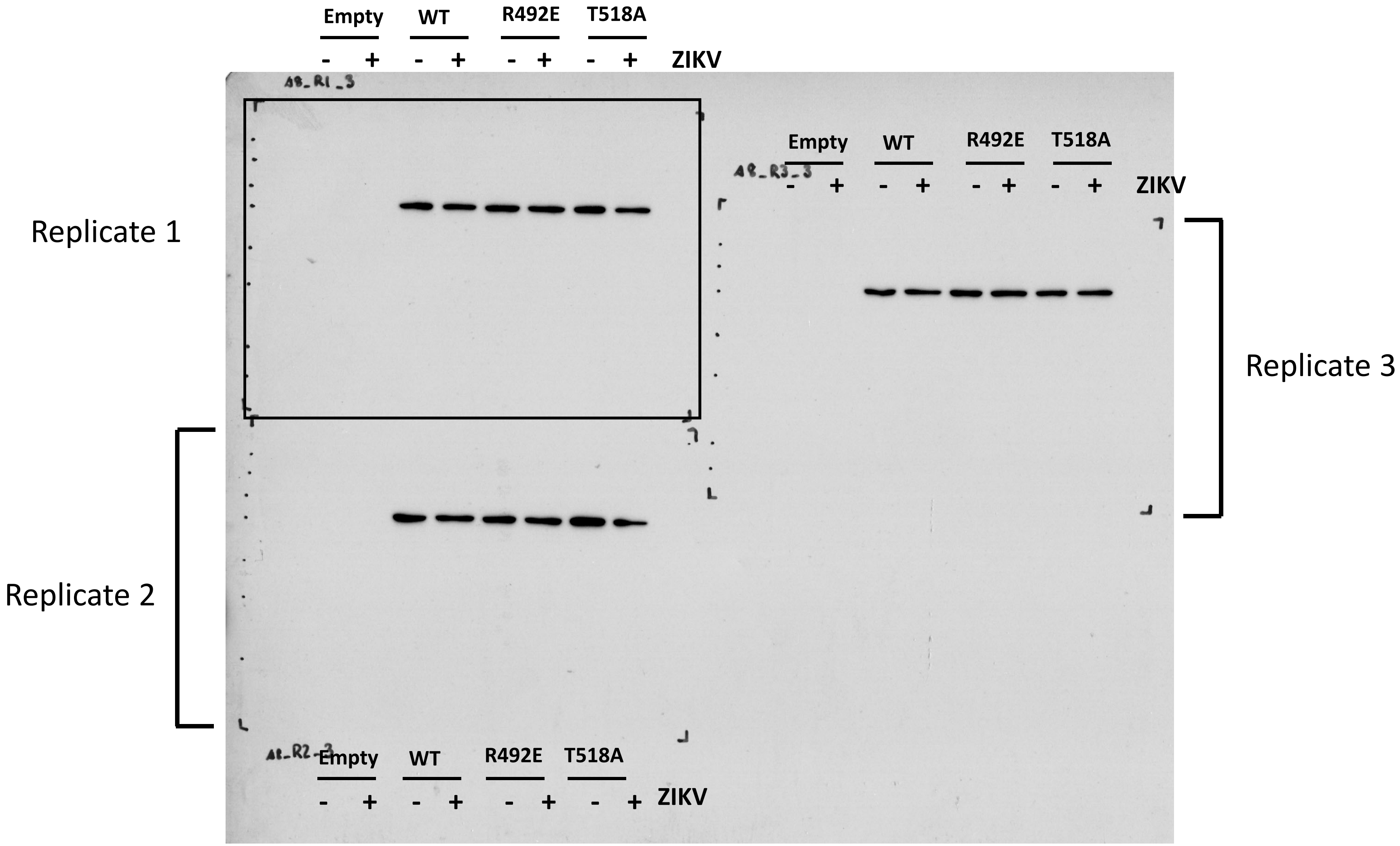

Figure 3A: ZIKV E at 24 h

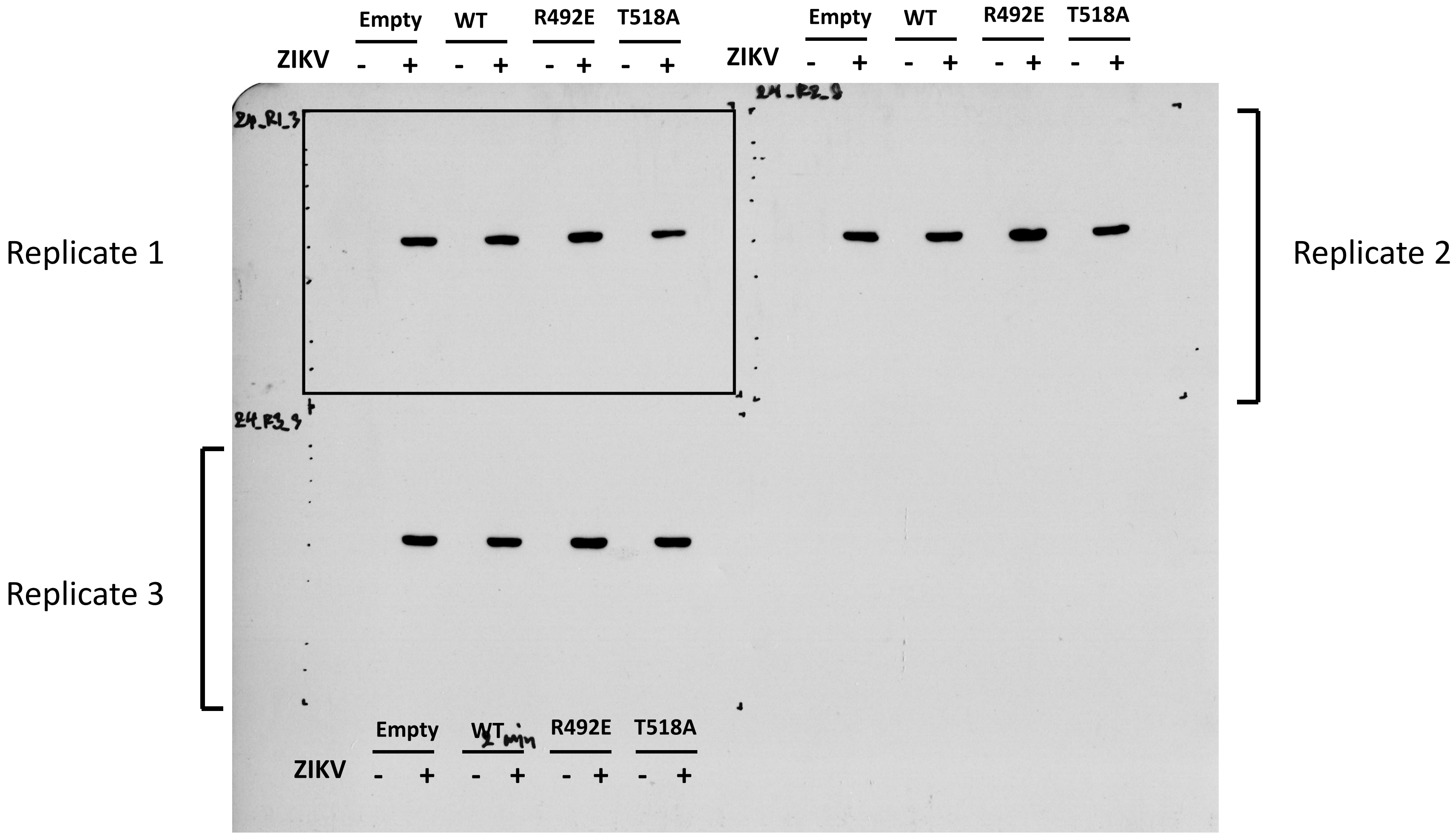

Figure 3A: ZIKV E at 48 h

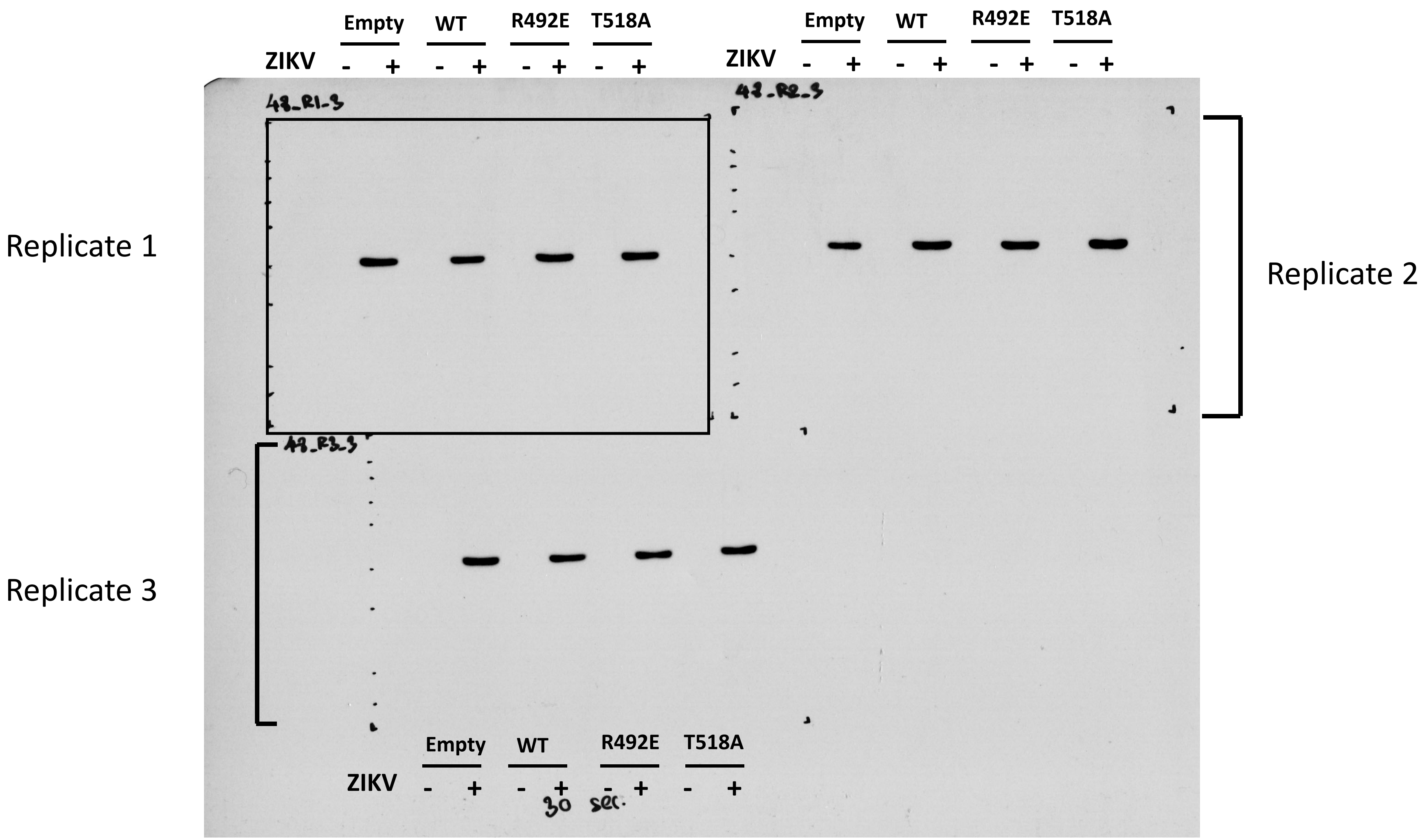

Figure 3A: ZIKV NS1 at 24 h

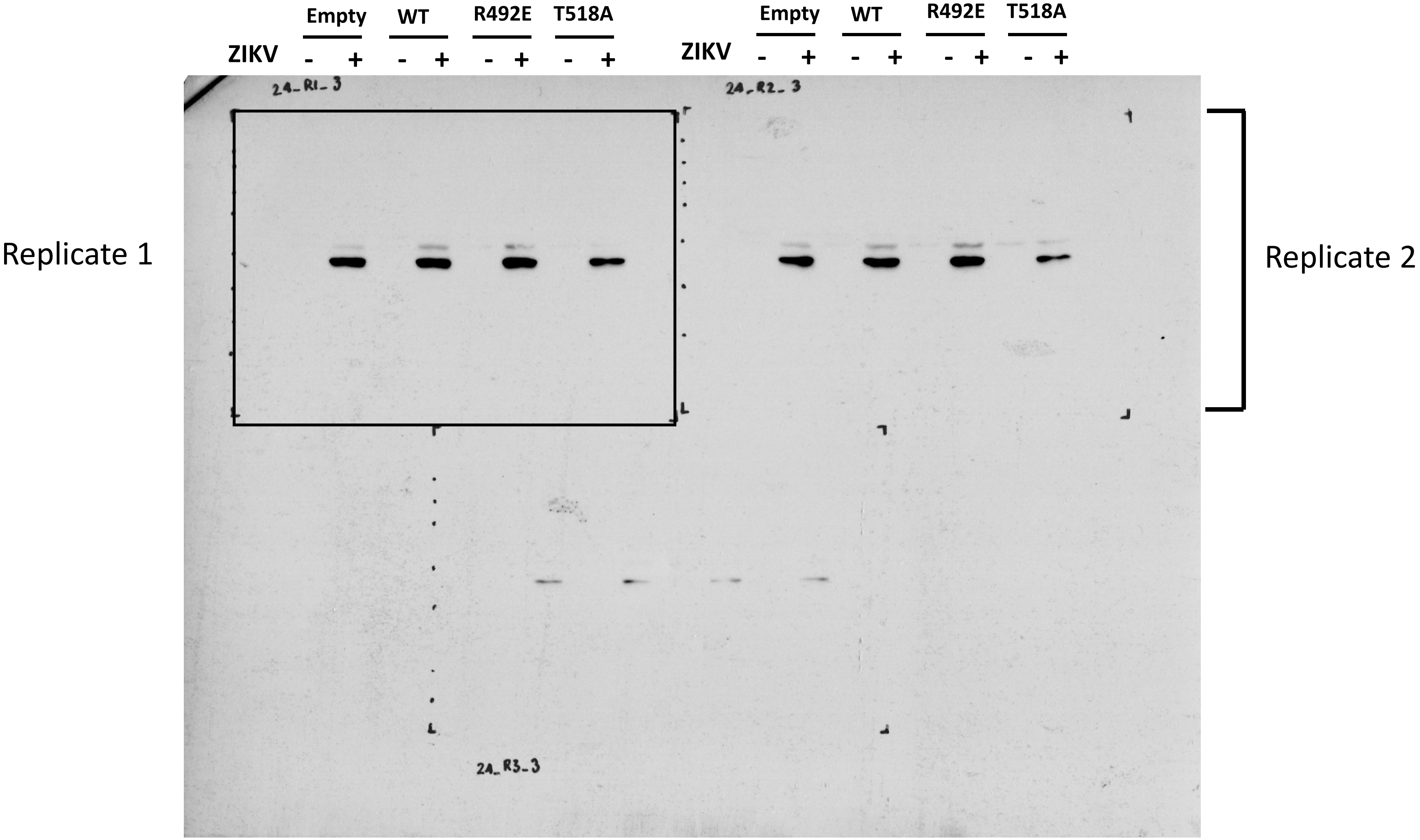

ZIKV NS1 at 24 h replicate 3 of Figure 3A

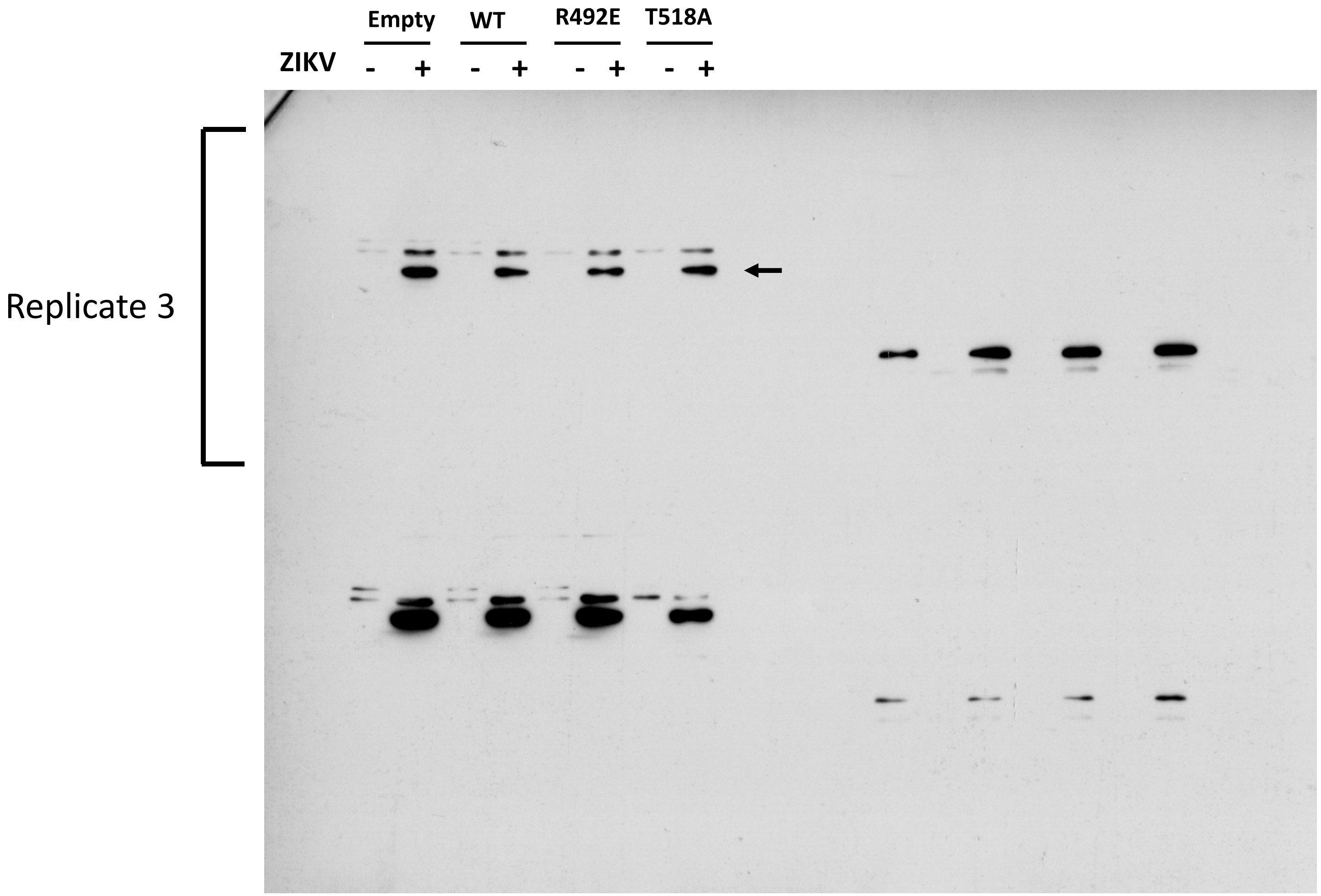

Figure 3A: ZIKV NS1 at 48 h

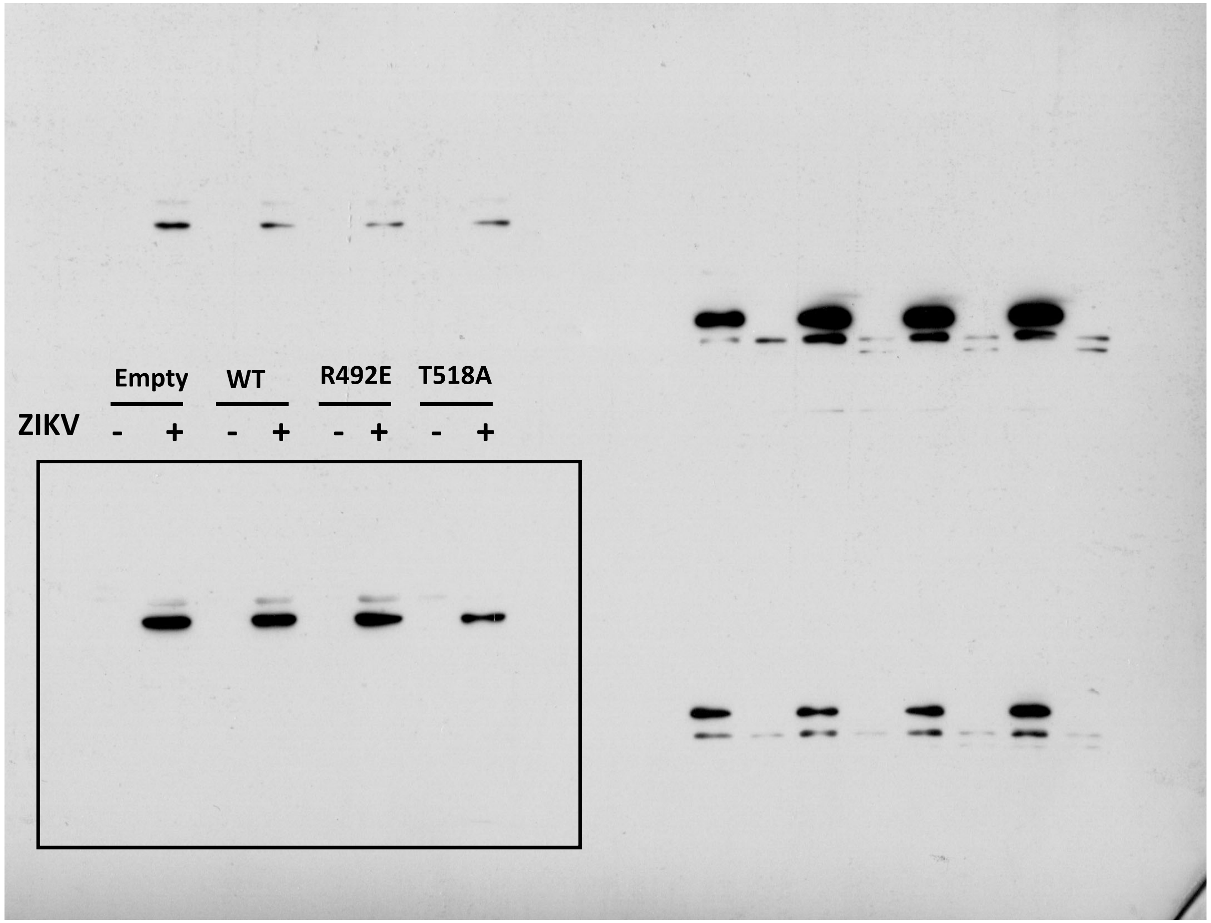

Replicate 1

ZIKV NS1 at 48 h replicate 2 and 3 of Figure 3A

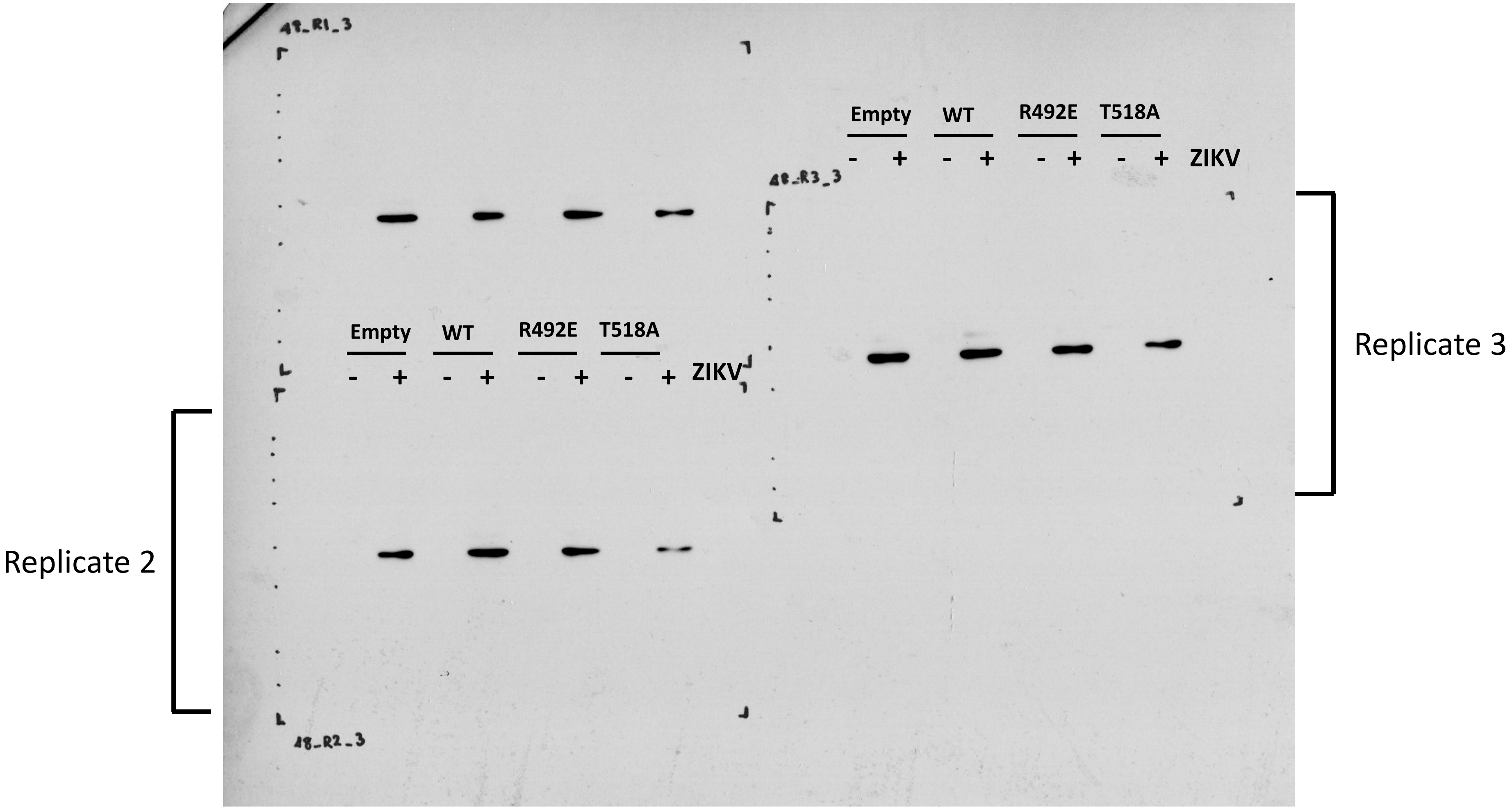

Figure 3A: Actin at 24 h

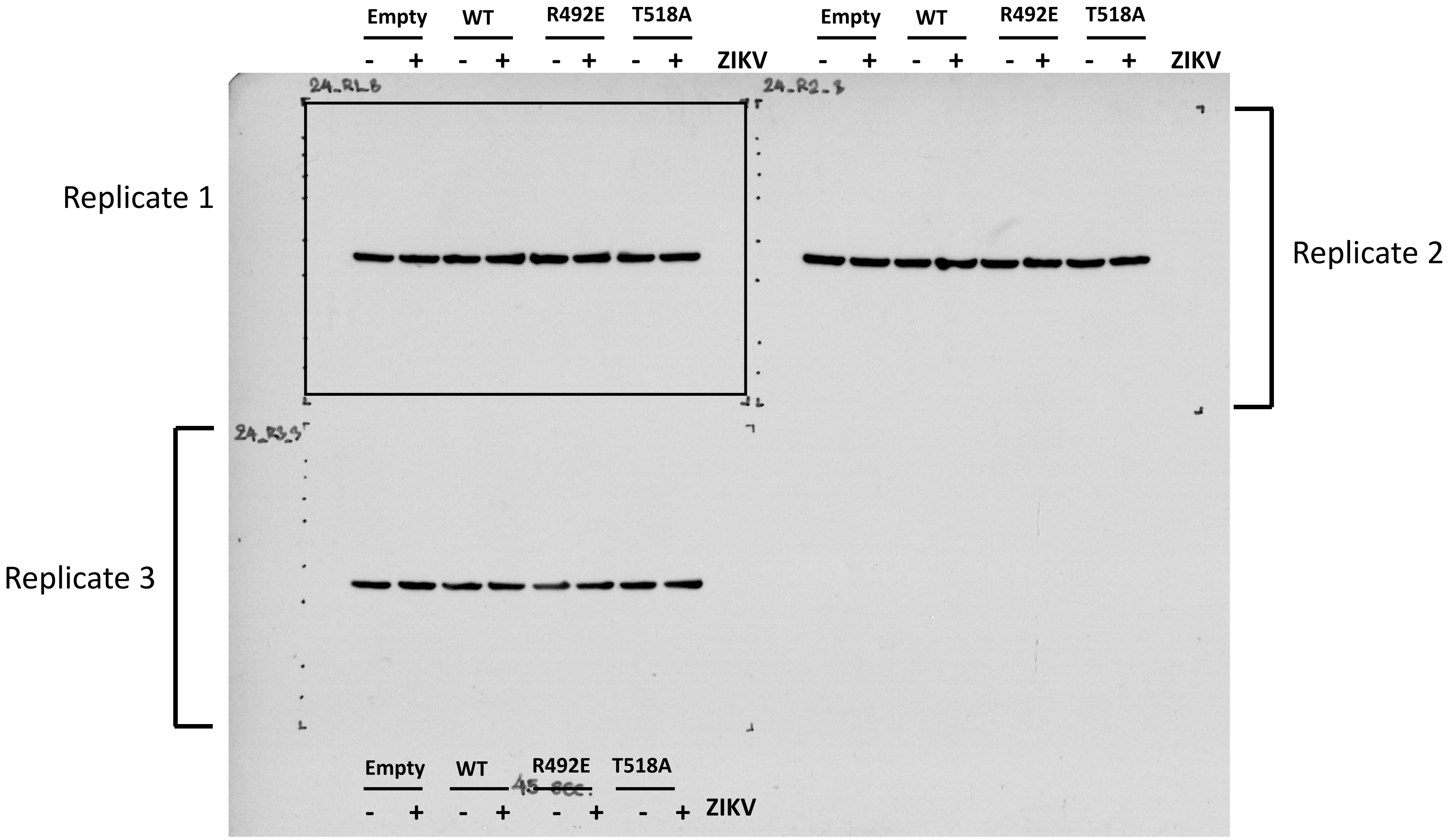

Figure 3A: Actin at 48 h

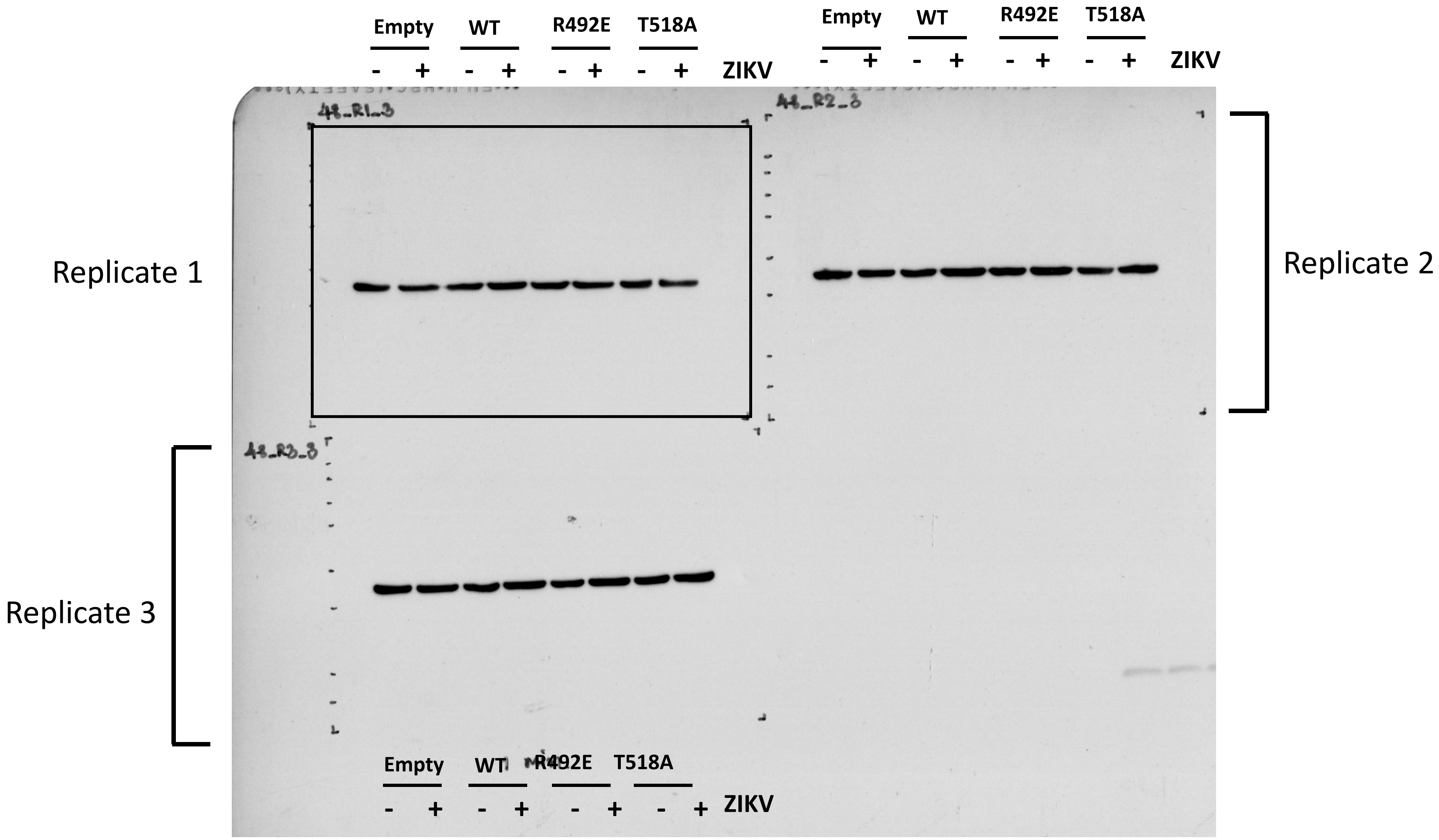

Figure 4B: Input ZIKV E-HA

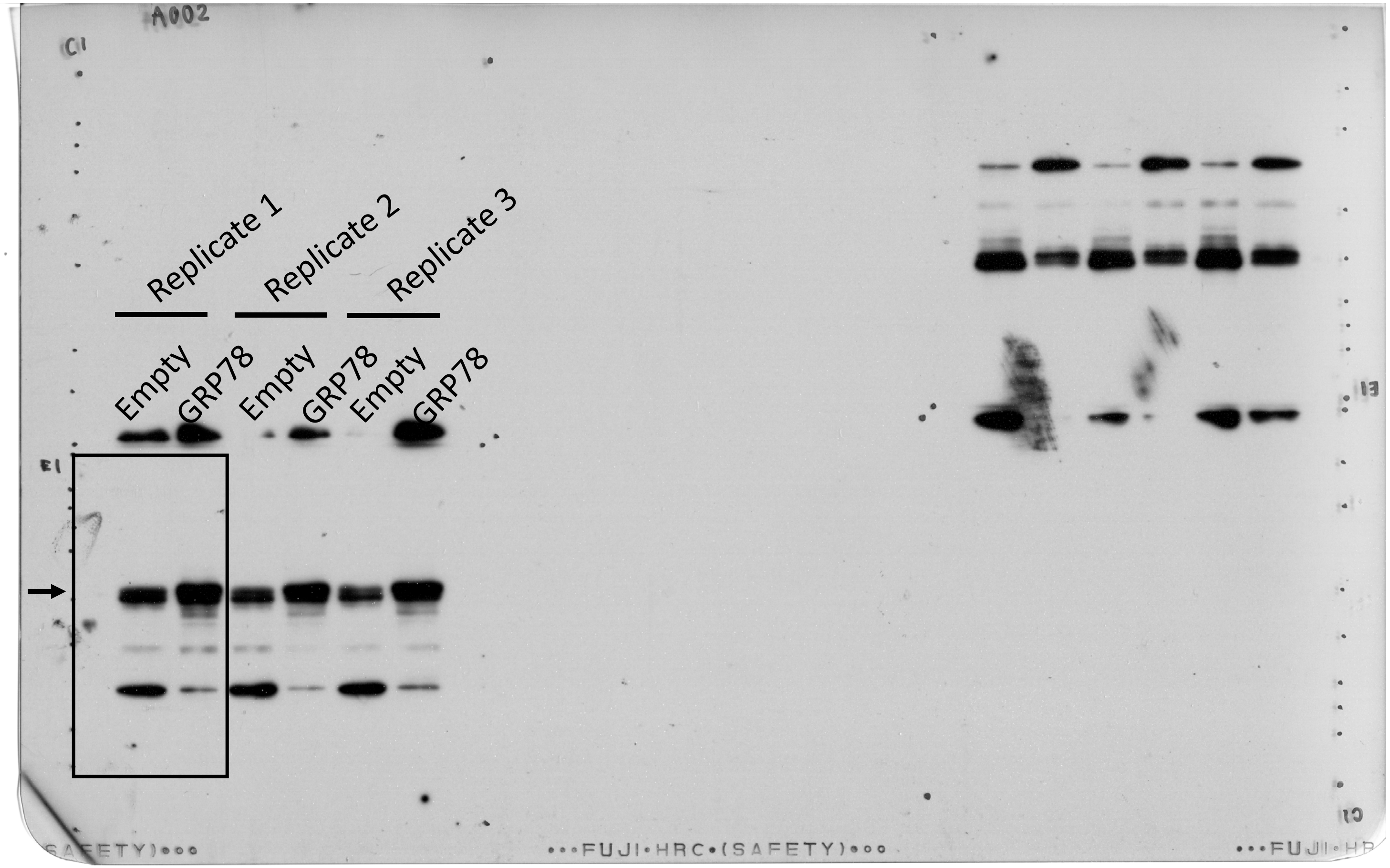

Figure 4B: Input EGFP-GRP78

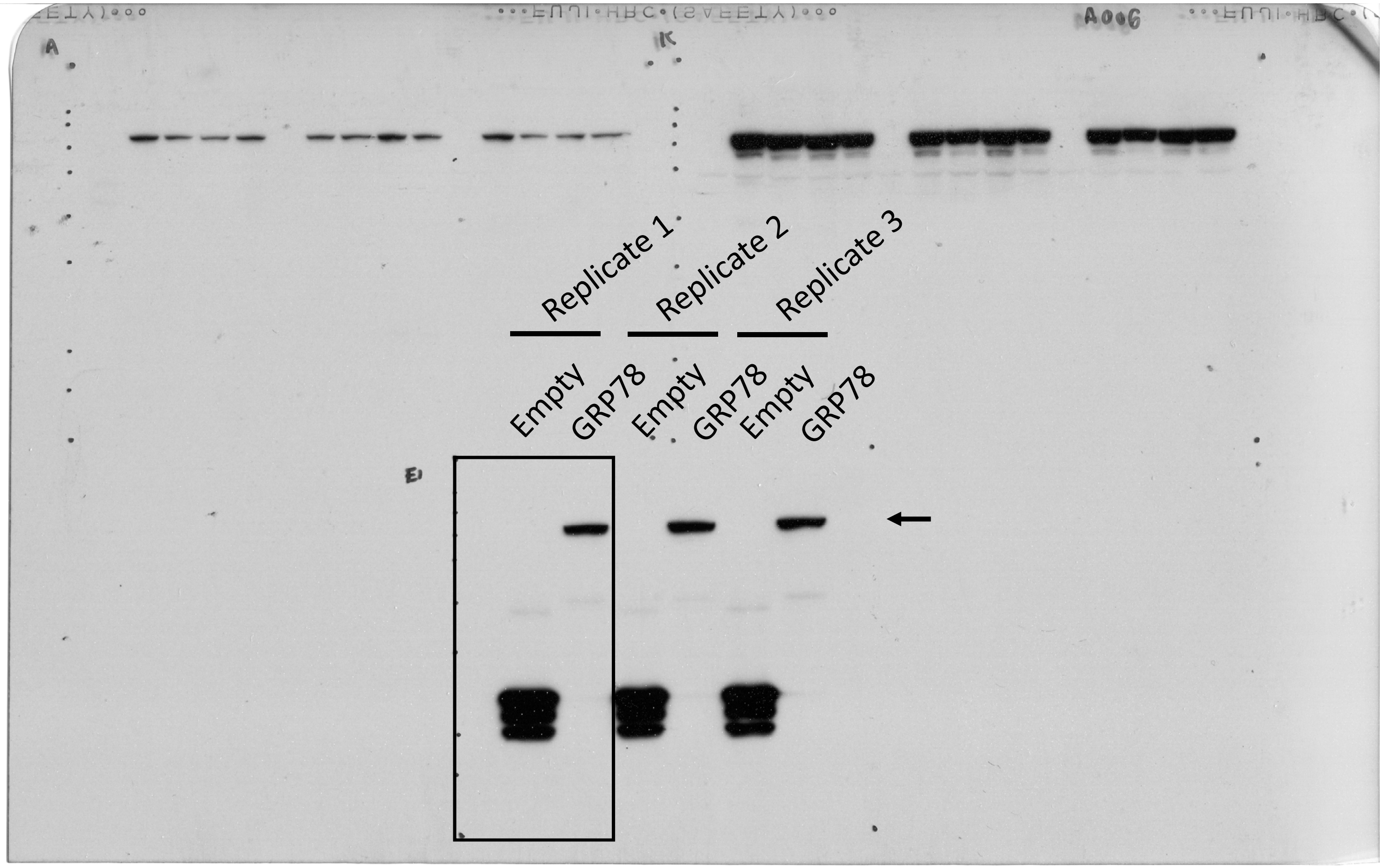

Figure 4B: Input Actin

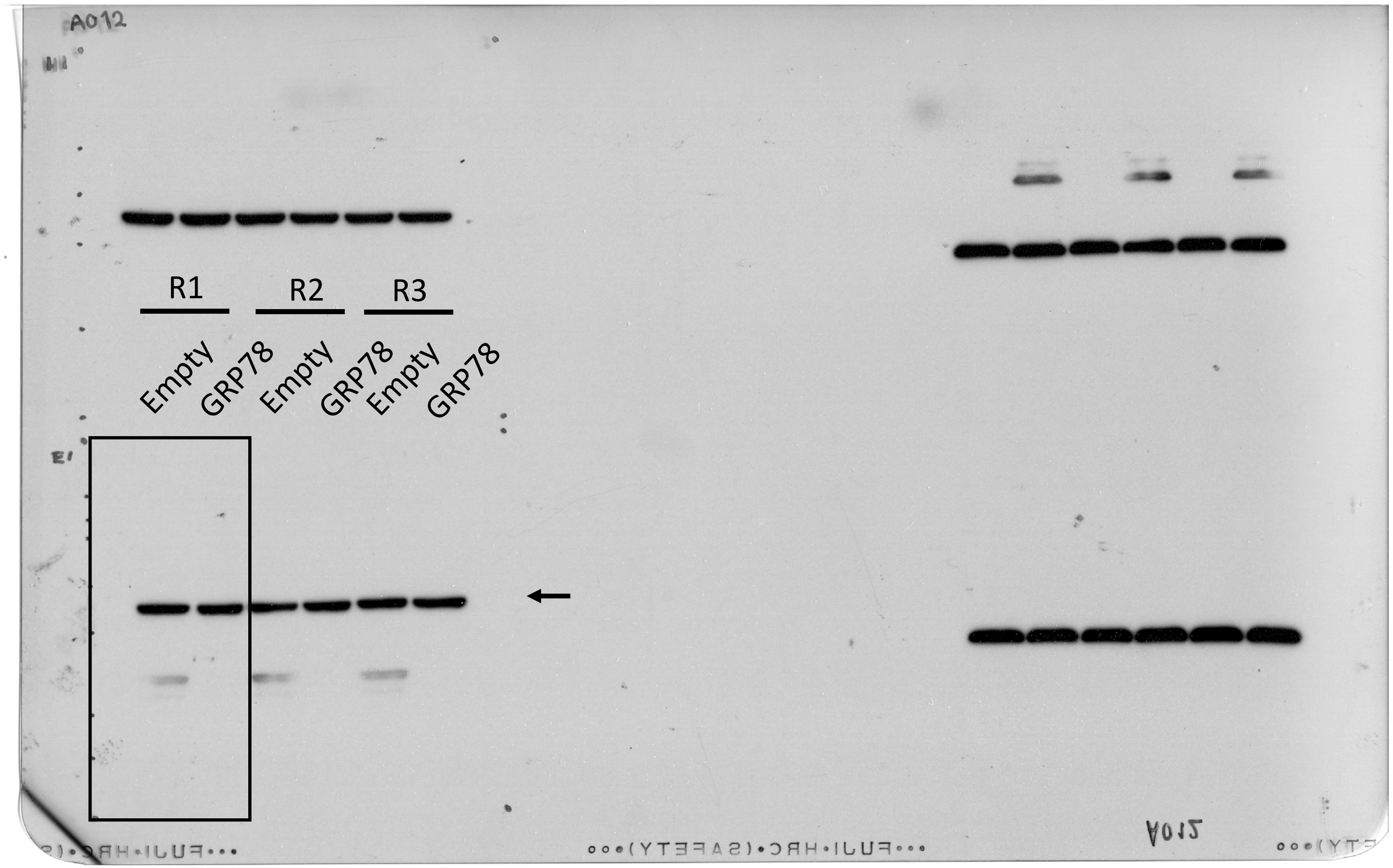

Figure 4B: Output ZIKV E-HA

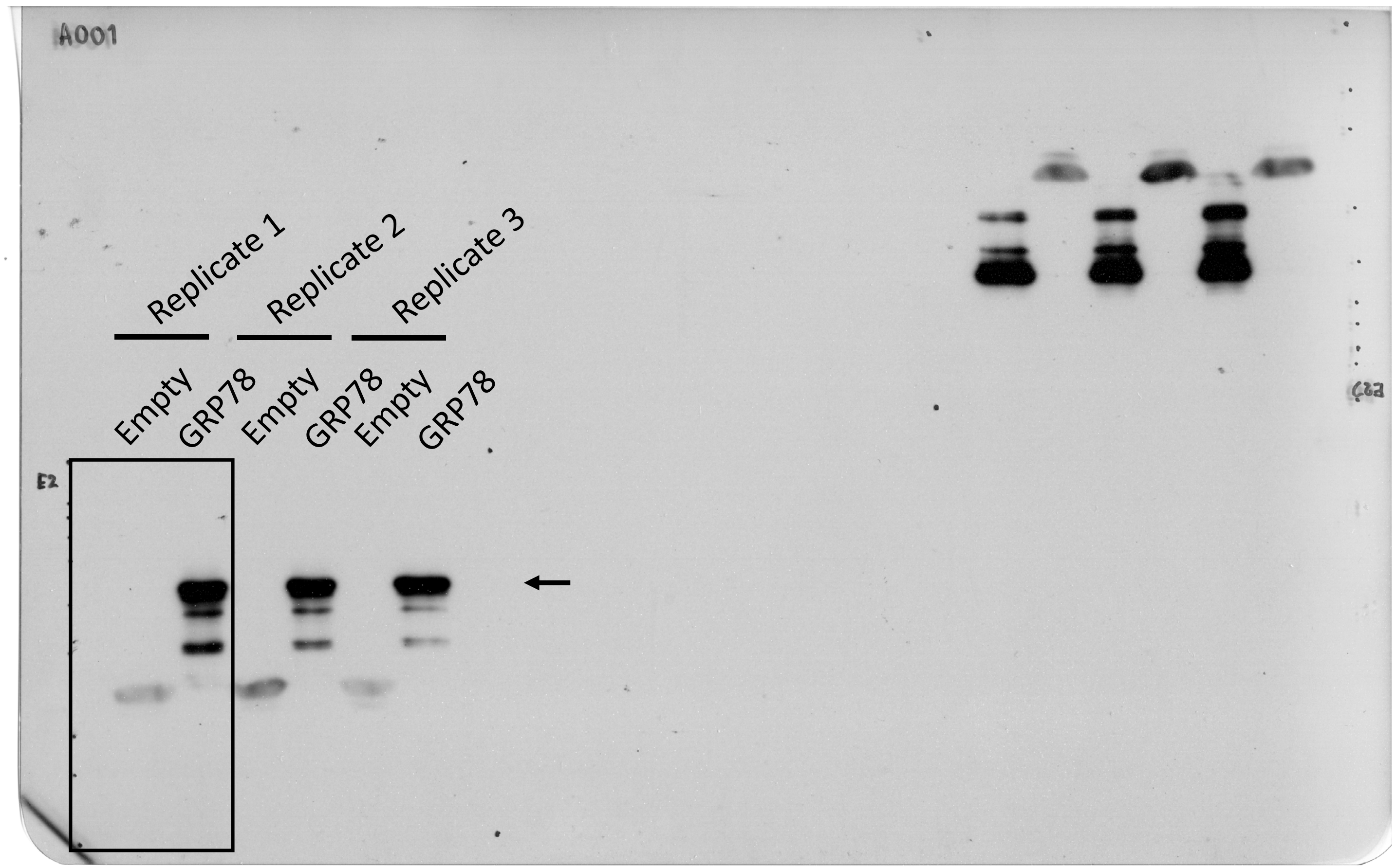

Figure 4B: Output EGFP-GRP78

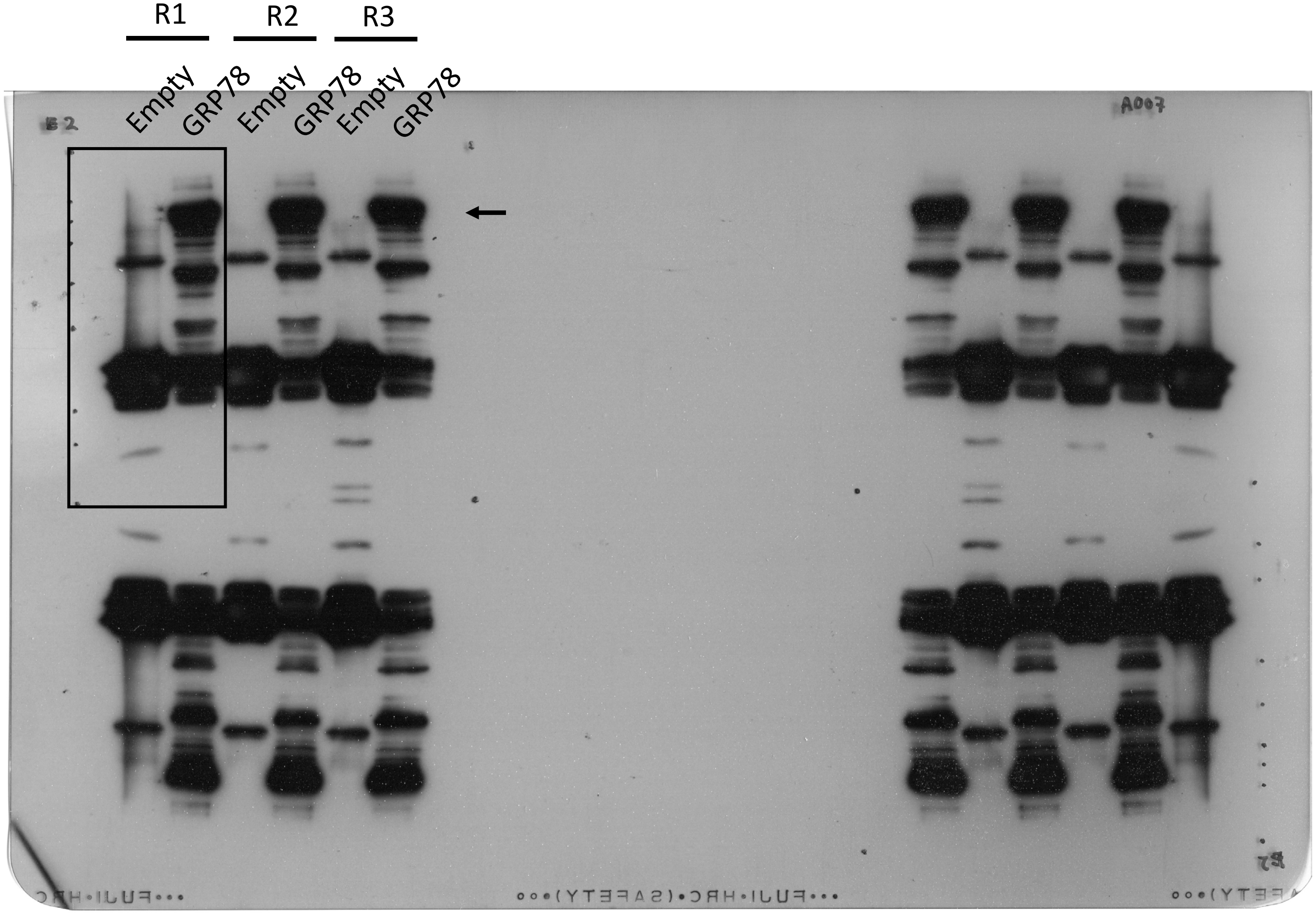

Figure 4C: Input ZIKV E-HA

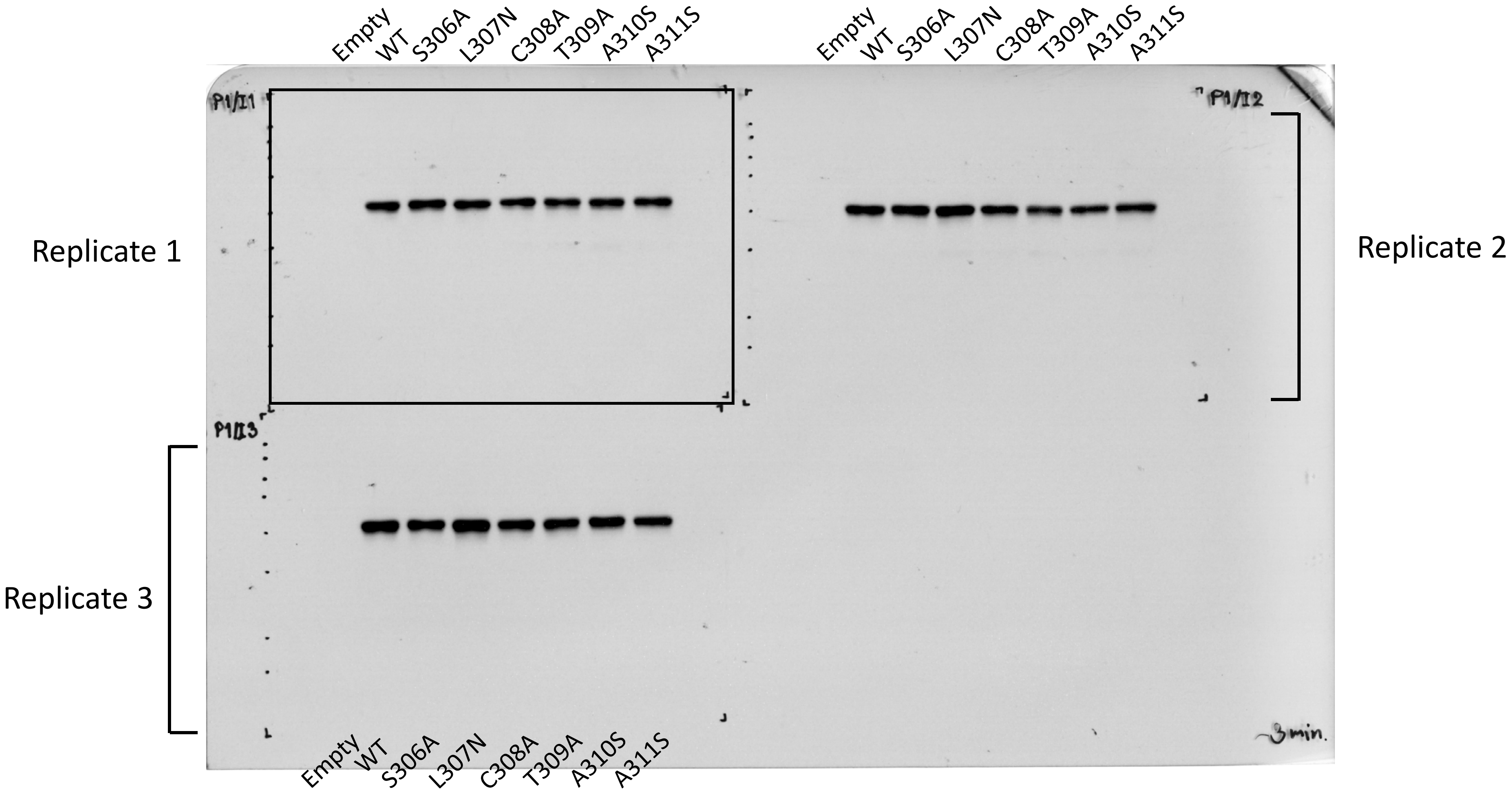

Figure 4C: Input EGFP-GRP78

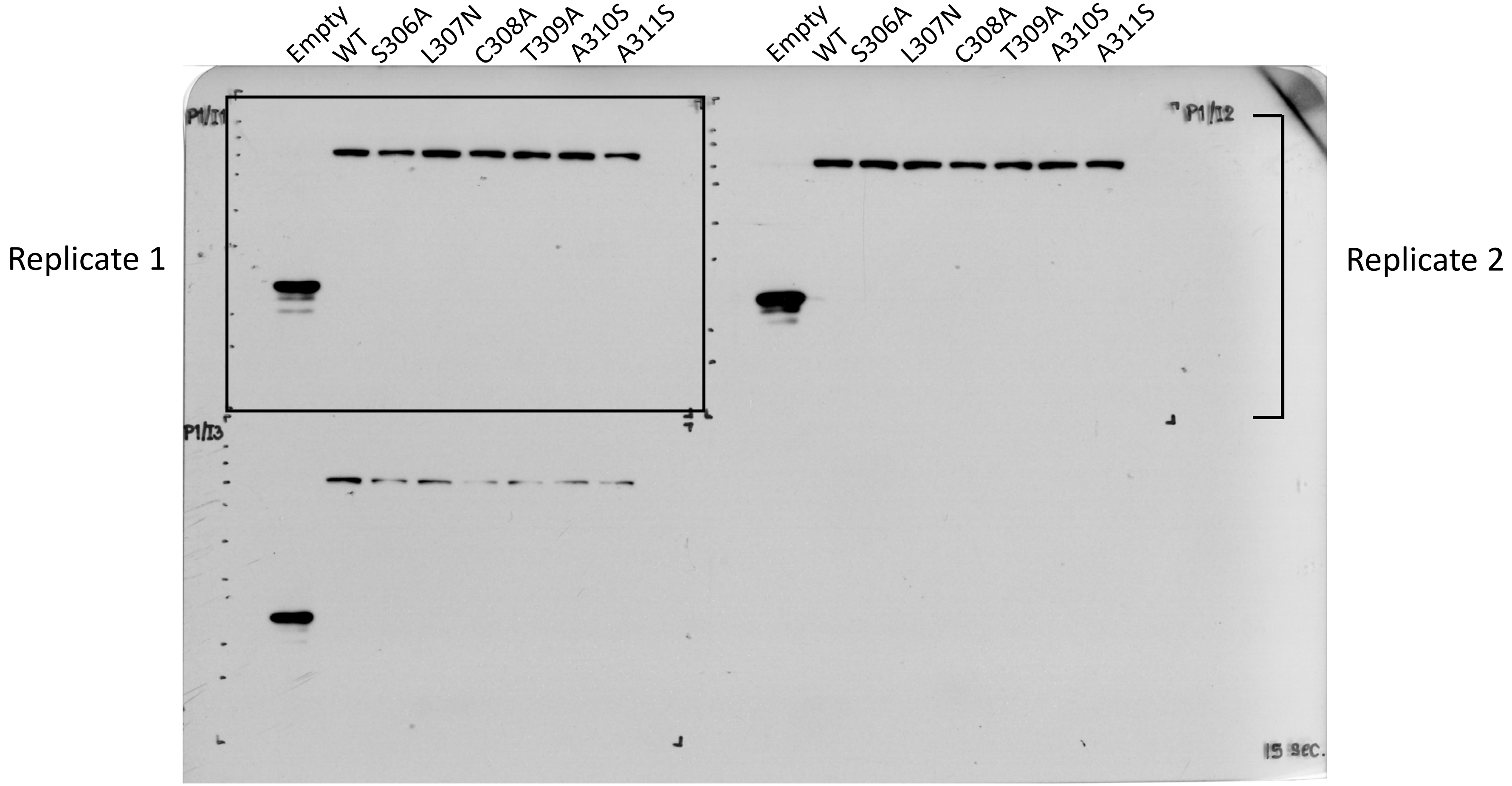

Input EGFP-GRP78 replicate 3 of Figure 4C

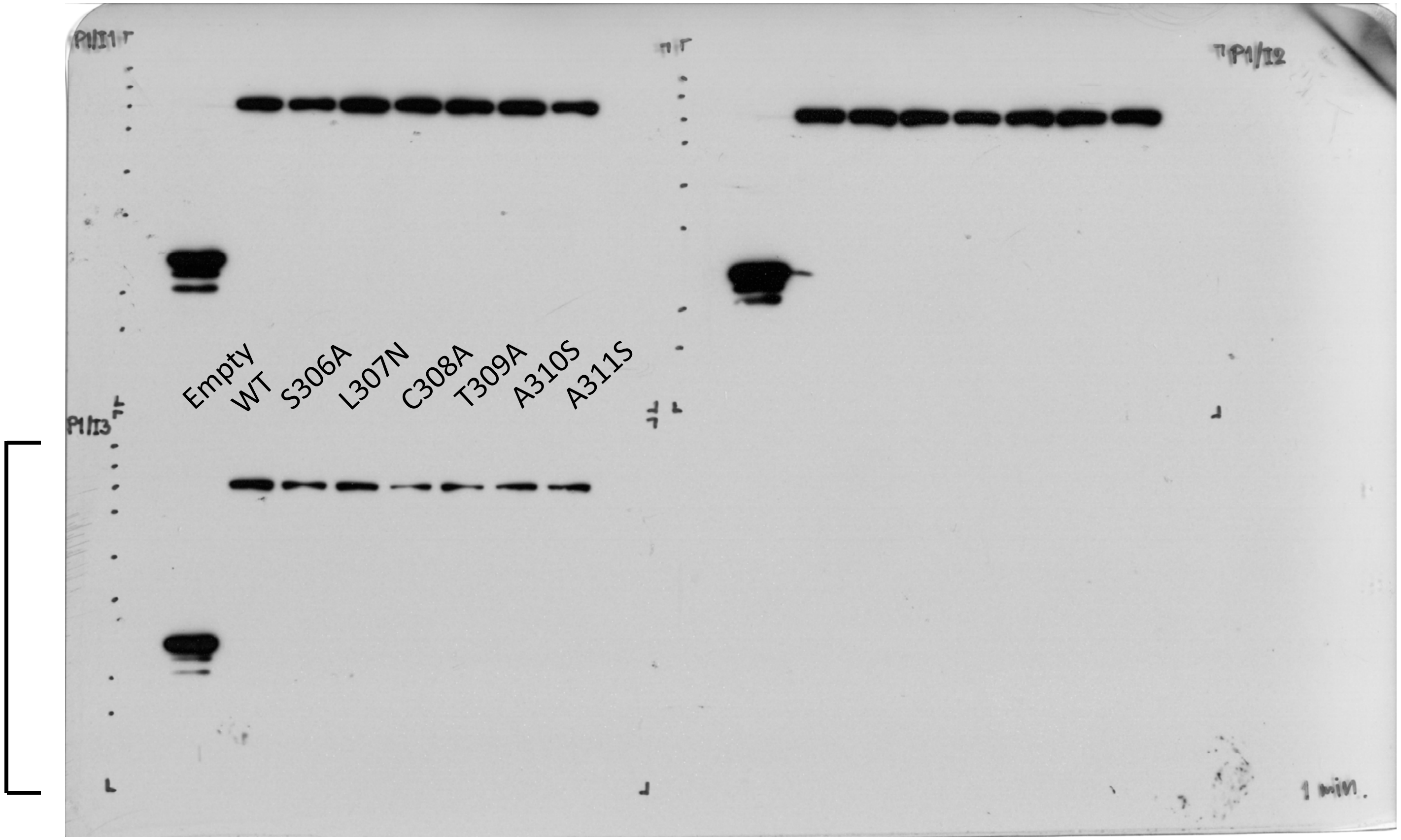

Replicate 3

Figure 4C: Input Actin

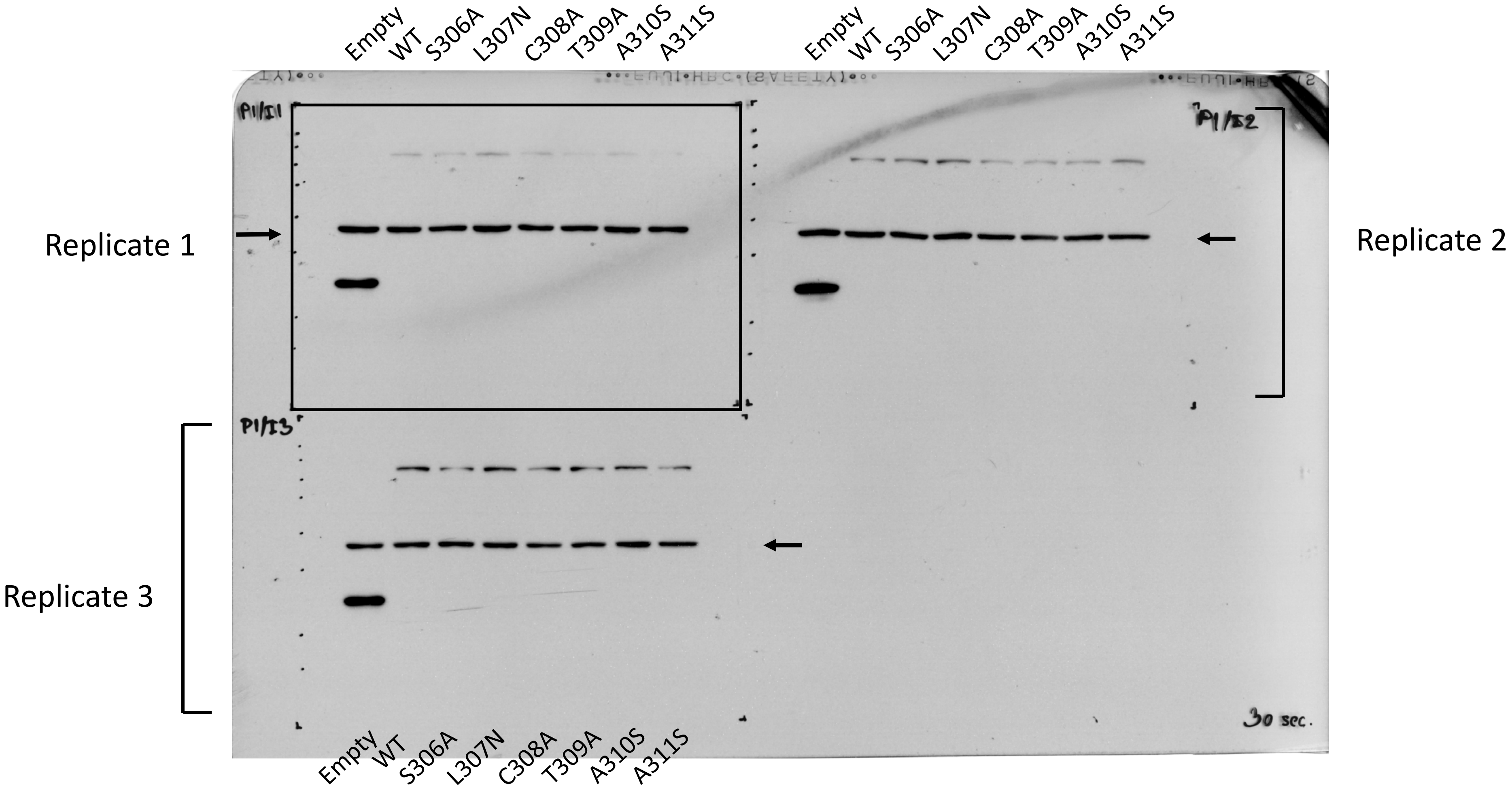

Figure 4C: Output ZIKV E-HA

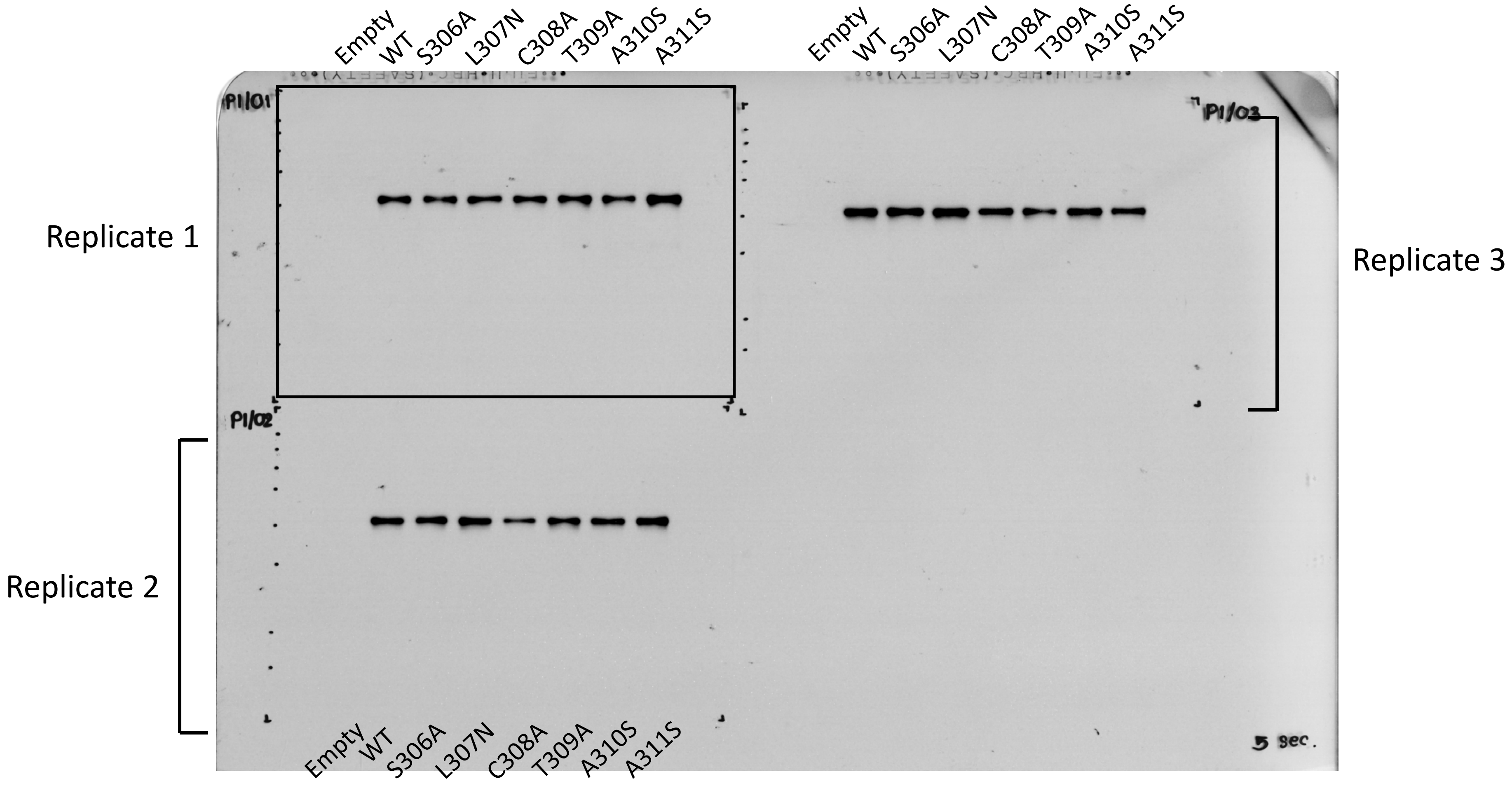

Figure 4C: Output EGFP-GRP78

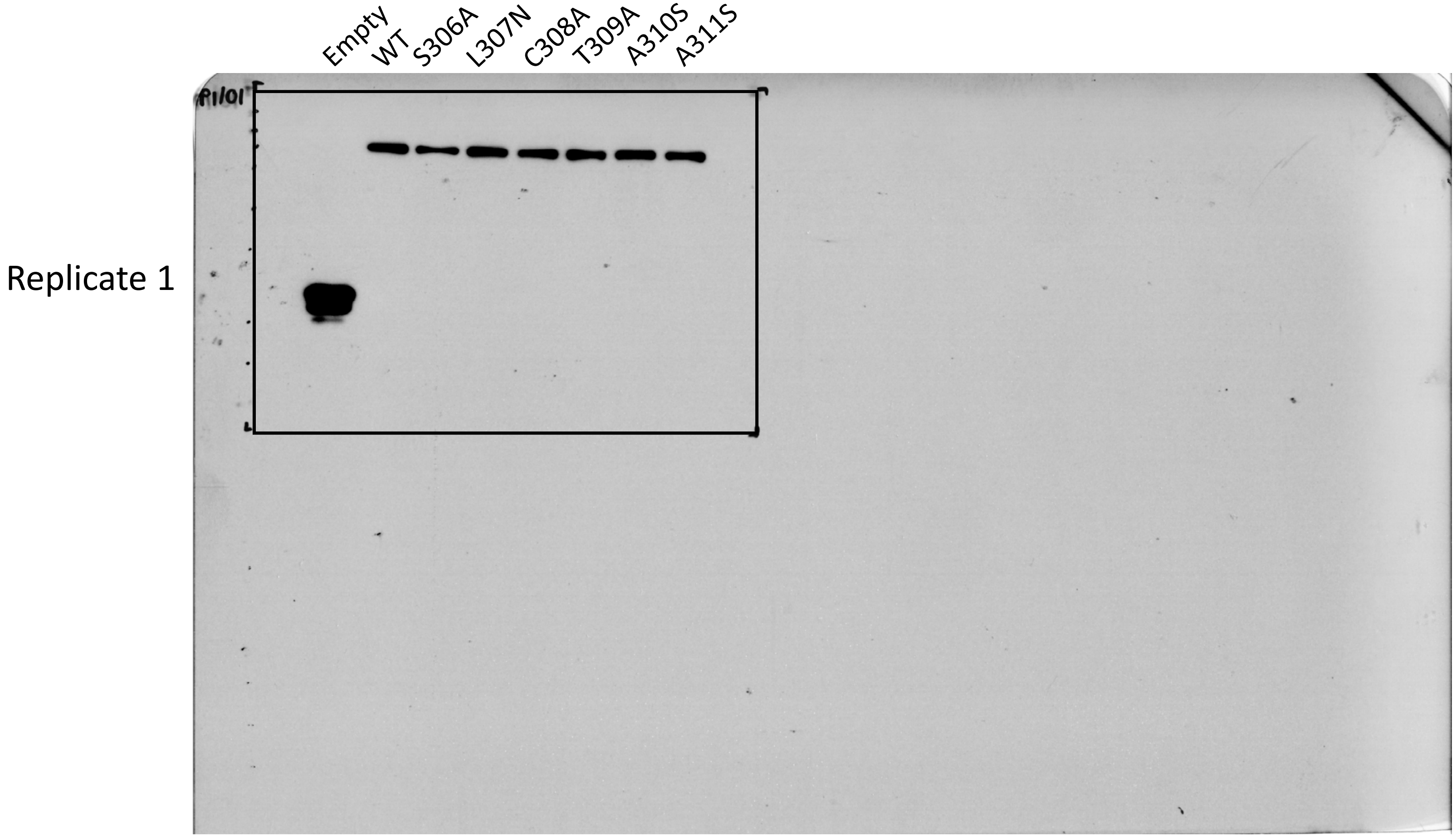

Output EGFP-GRP78 replicate 2 and 3 of Figure 4C

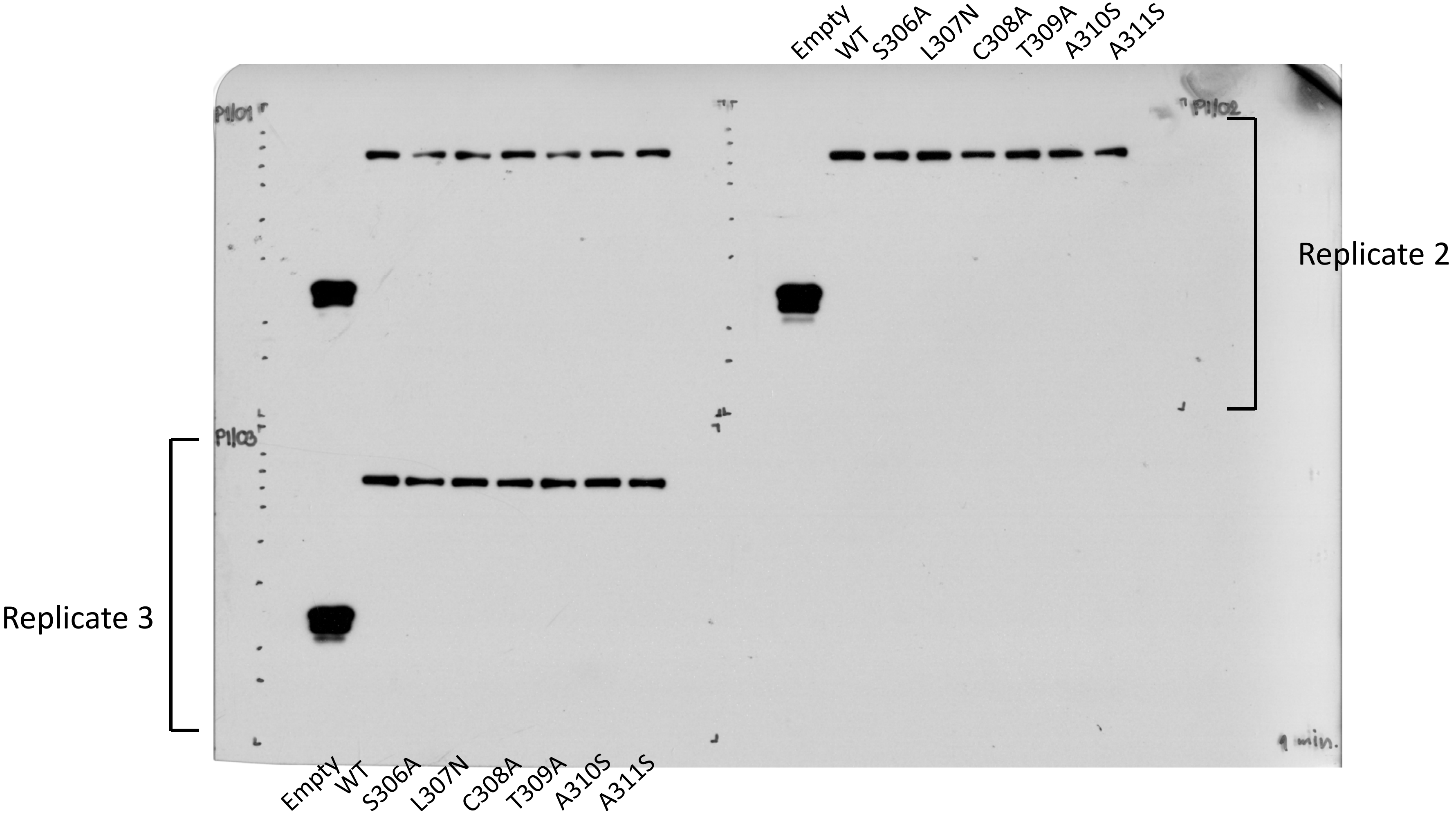

Figure 4E: Input ZIKV E-HA

Replicate 1

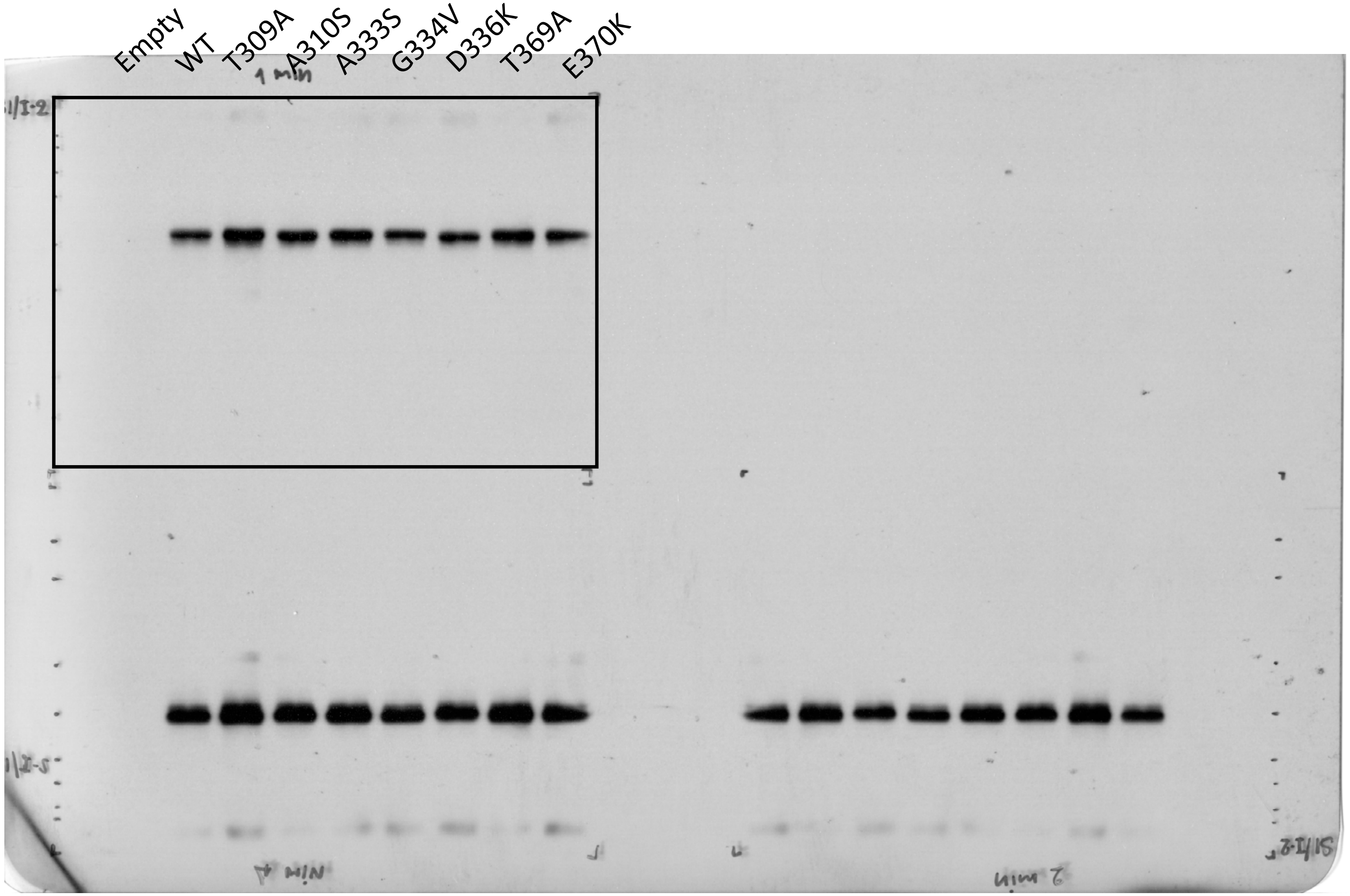

Input ZIKV E-HA replicate 2 of Figure 4E

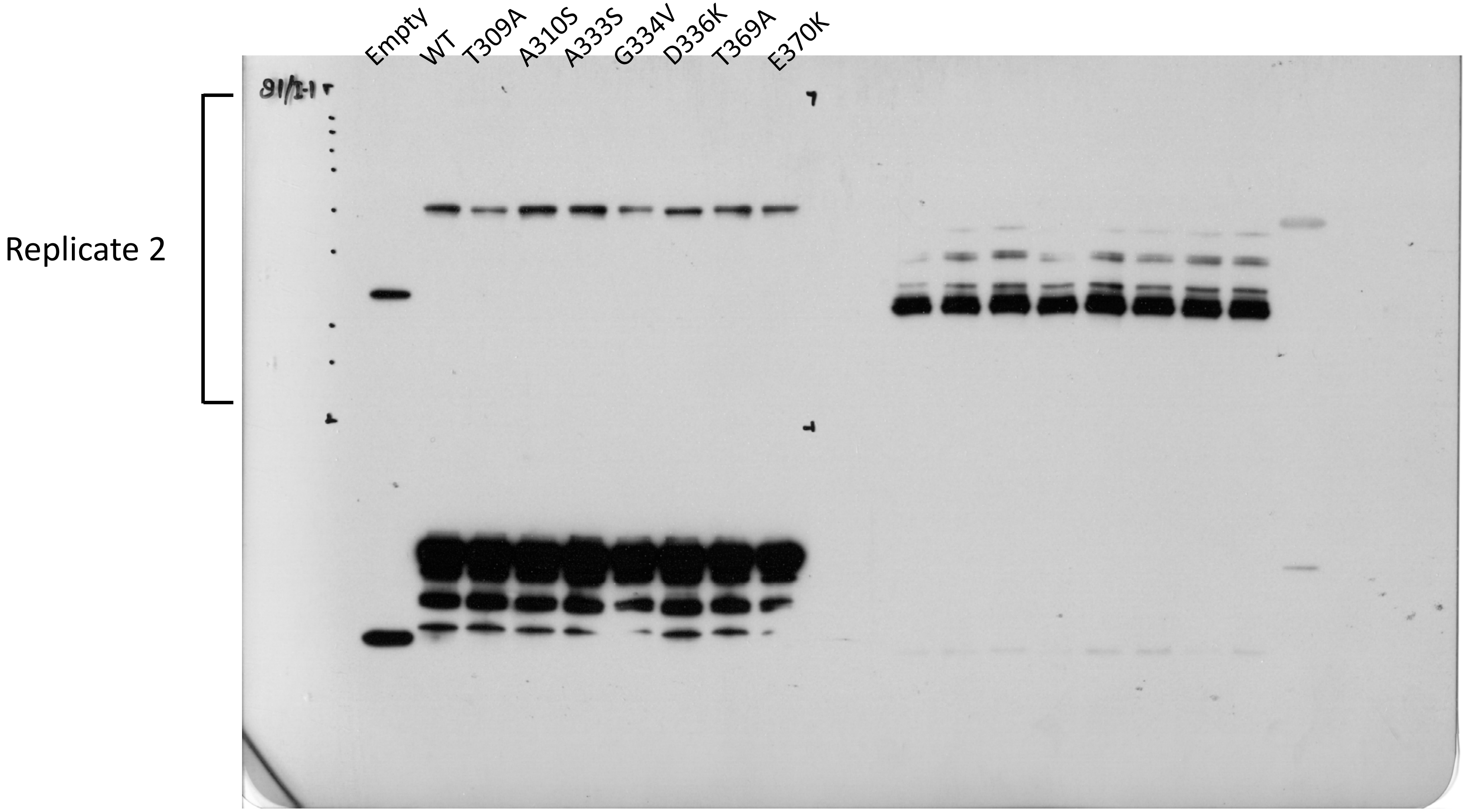

Figure 4E: Input EGFP-GRP78

Replicate 1

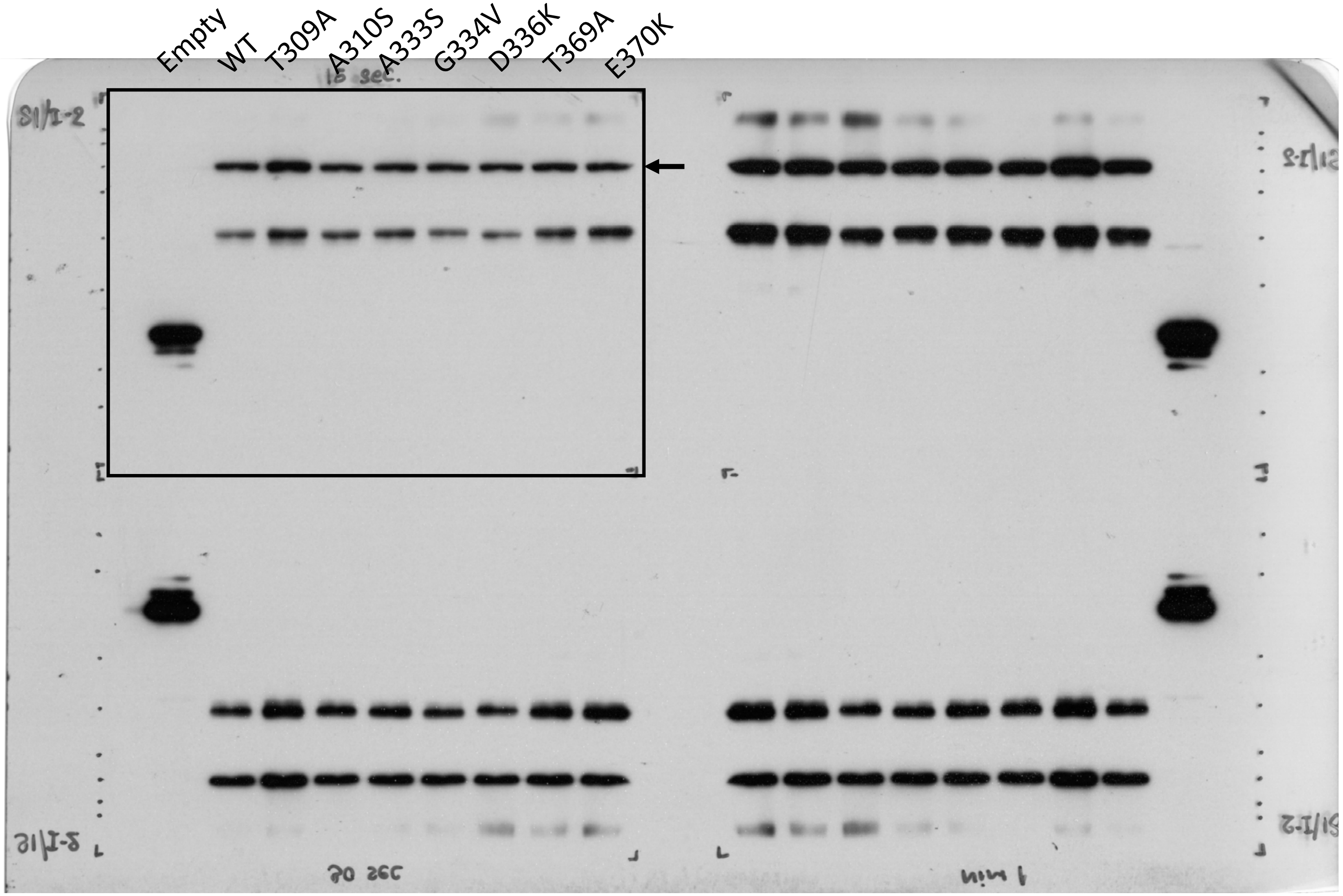

Input EGFP-GRP78 replicate 2 of Figure 4E

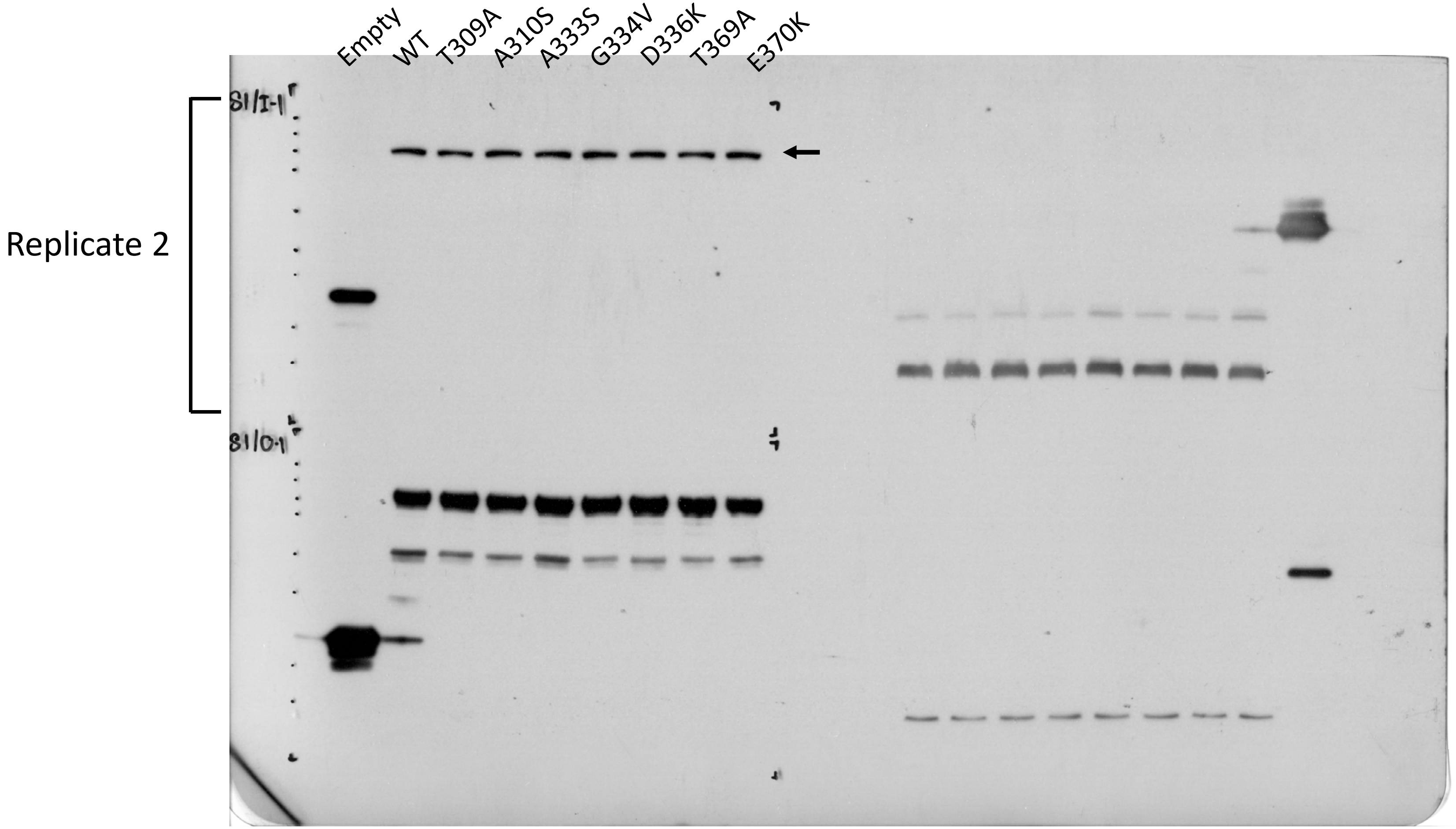

Figure 4E: Input Actin

Replicate 1

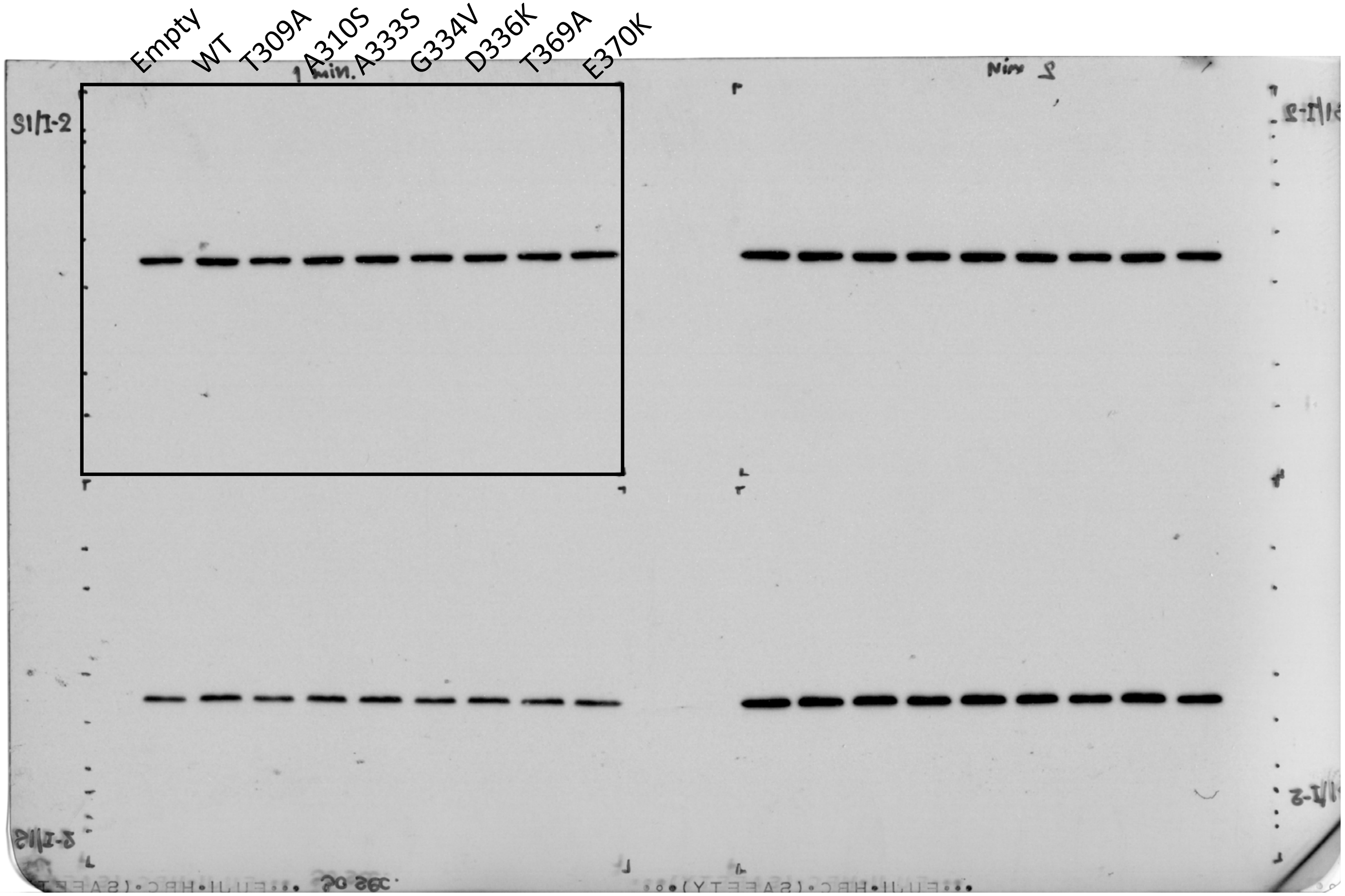

Input Actin replicate 2 of Figure 4E

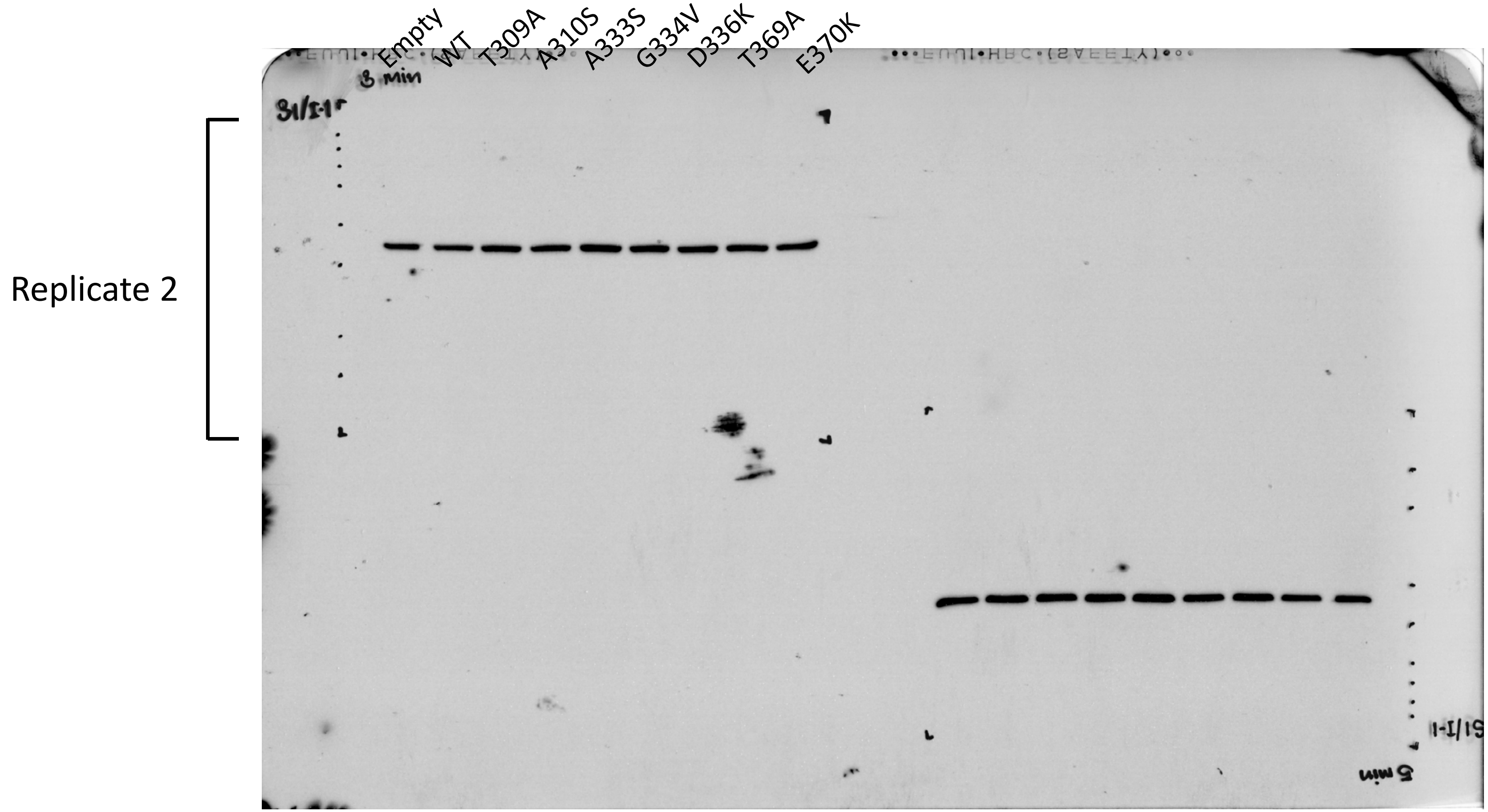

Figure 4E: Output ZIKV E-HA

Replicate 1

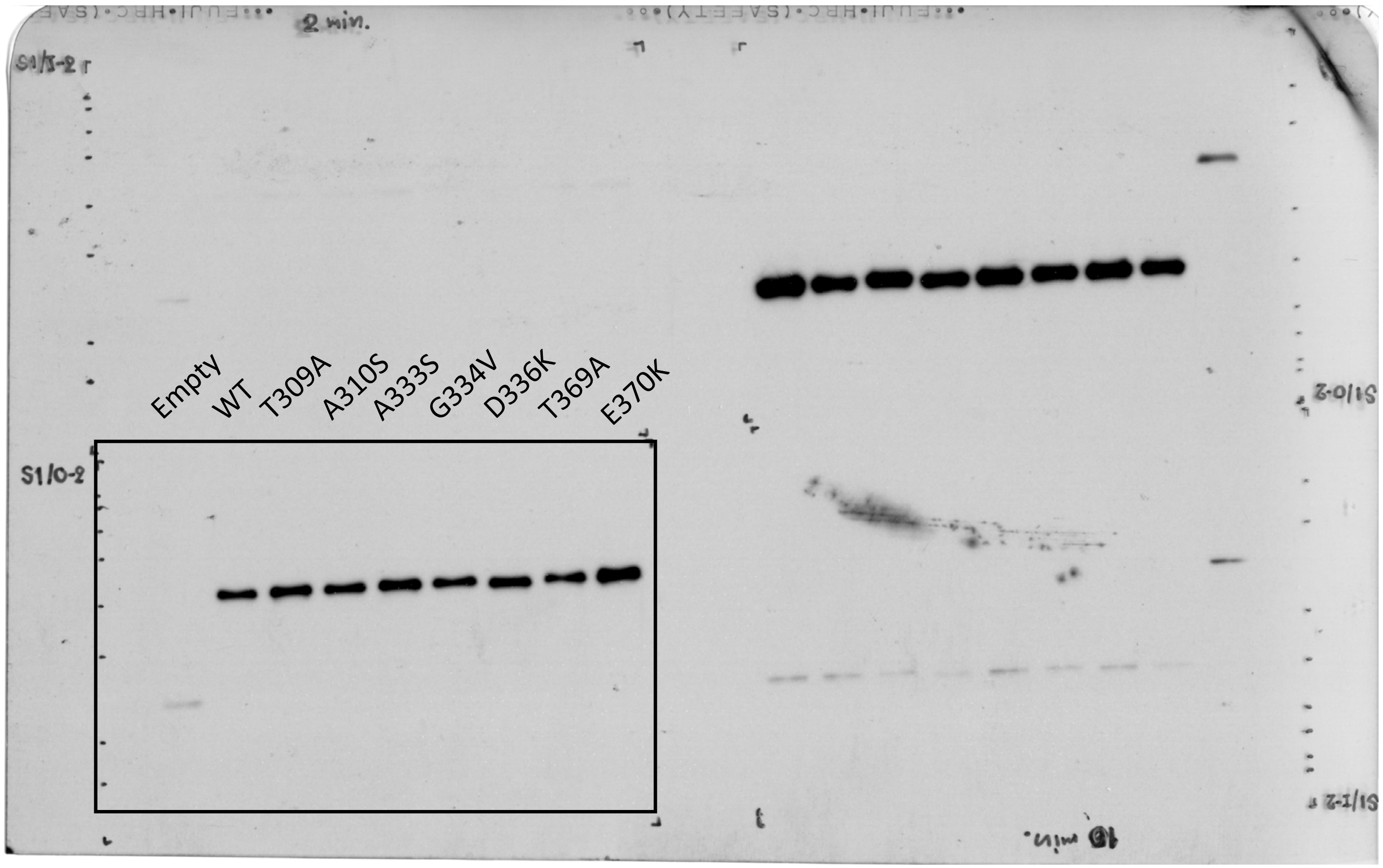

Output ZIKV E-HA replicate 2 of Figure 4E

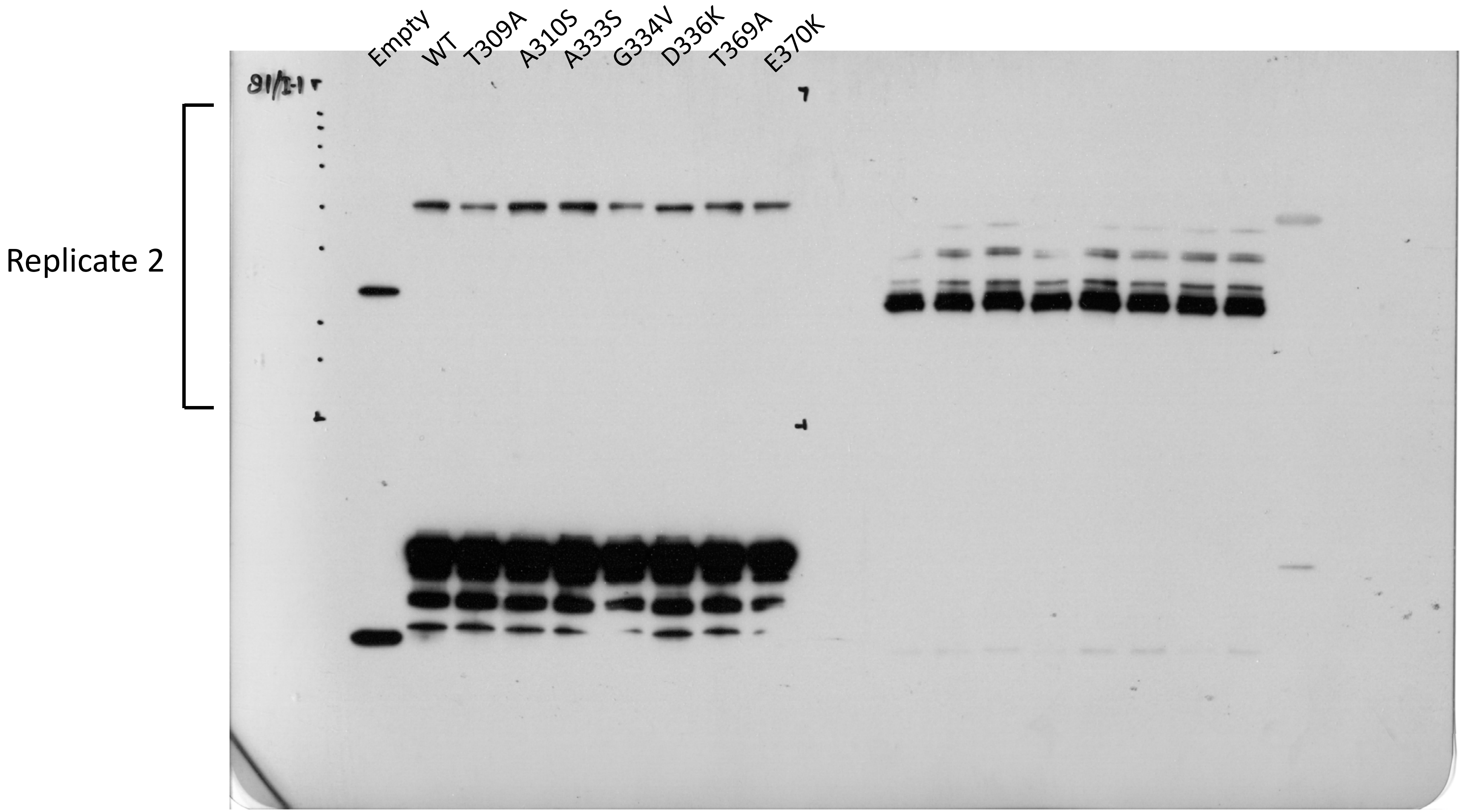

Figure 4E: Output EGFP-GRP78

Replicate 1

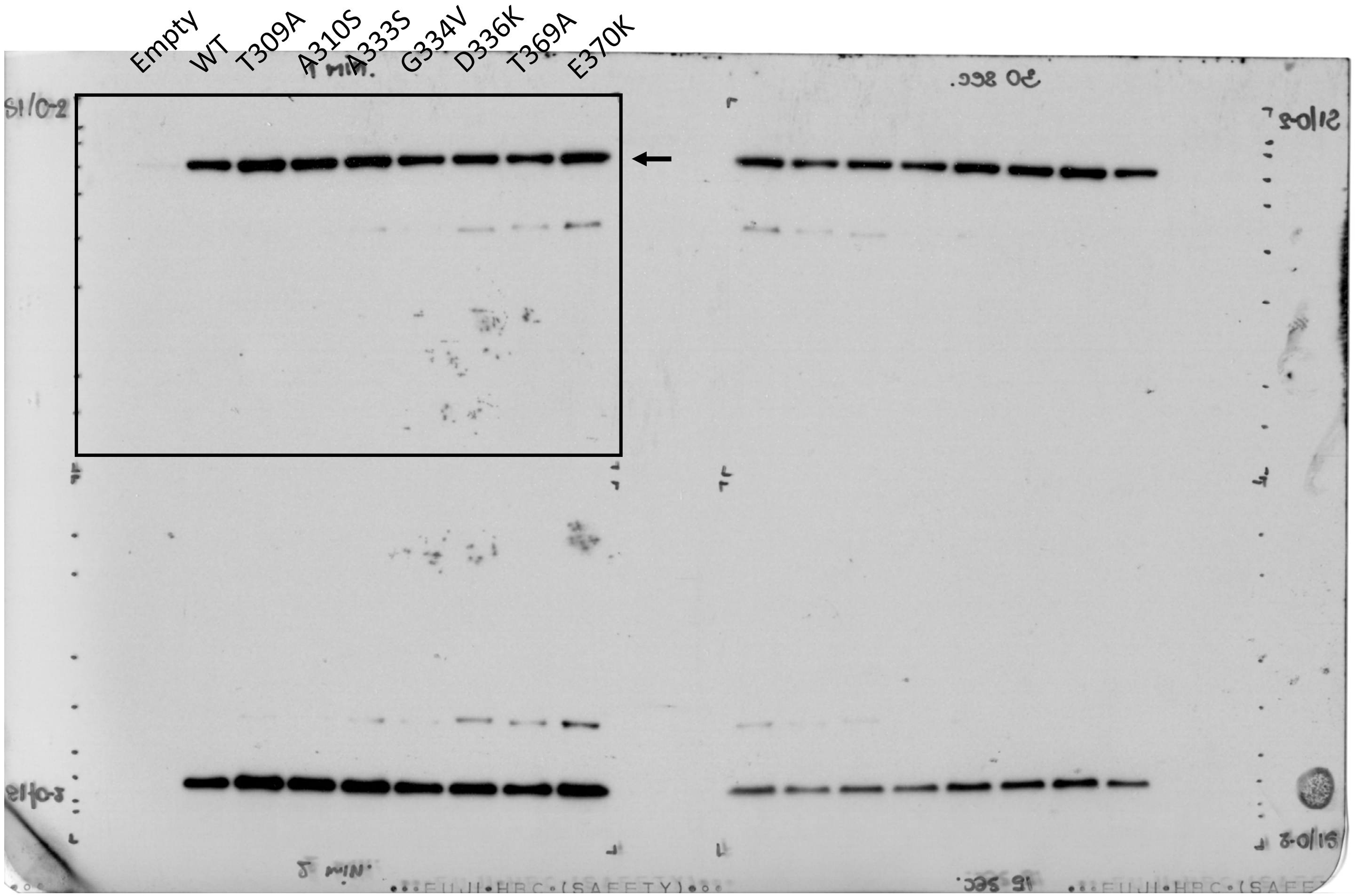

Output EGFP-GRP78 replicate 2 of Figure 4E

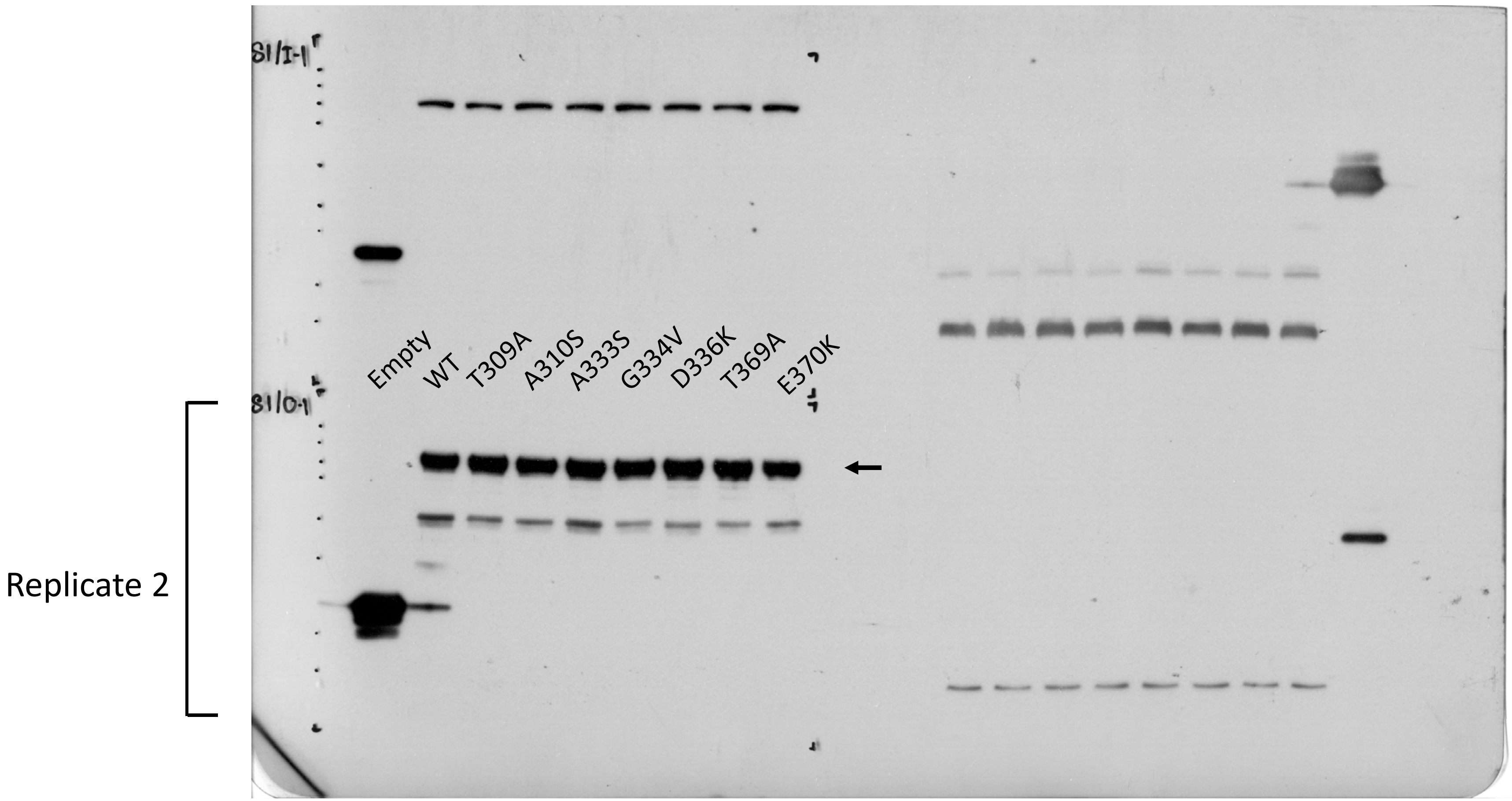

Figure 5B: Input EGFP-GRP78

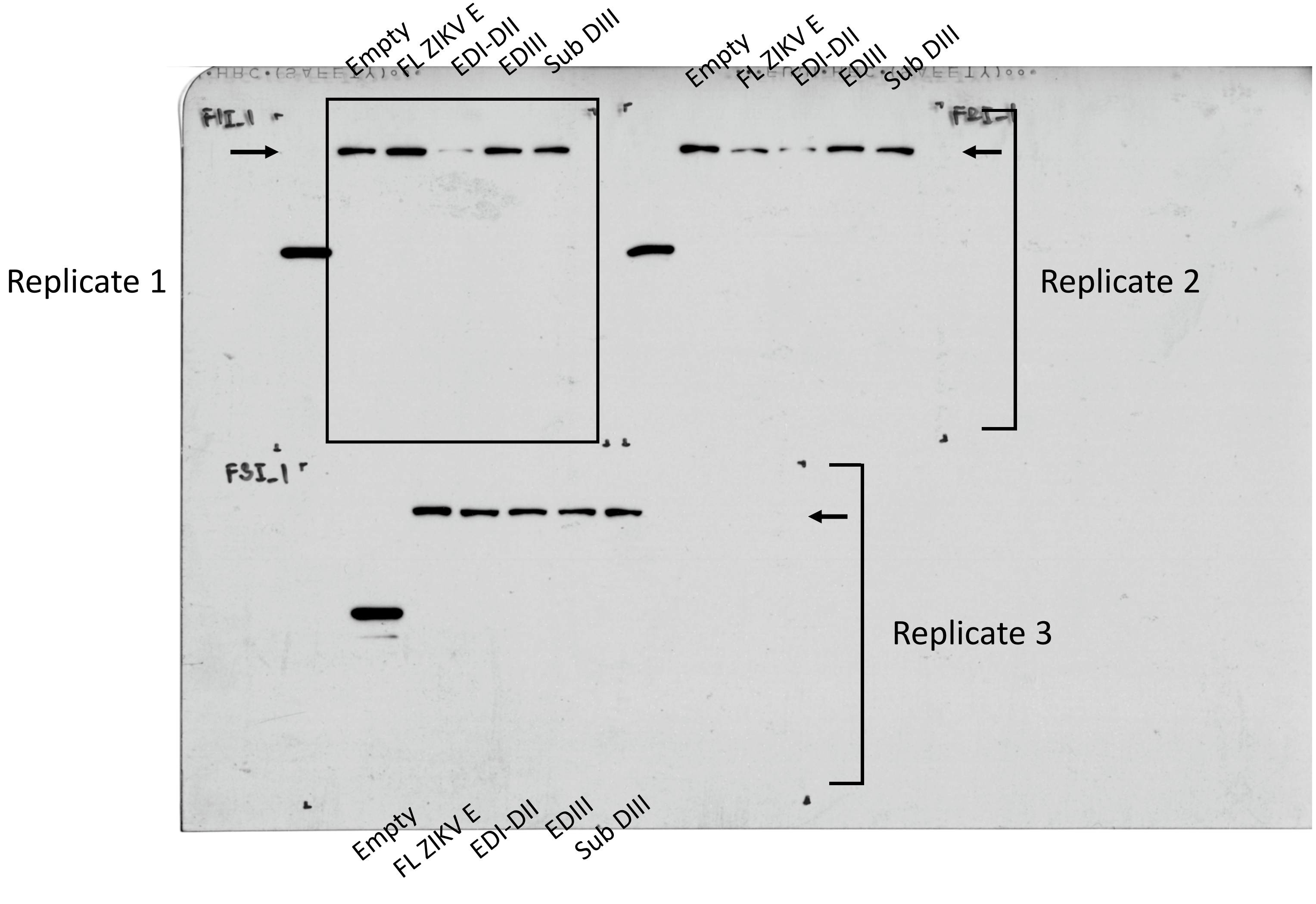

Figure 5B: Input full length ZIKV E

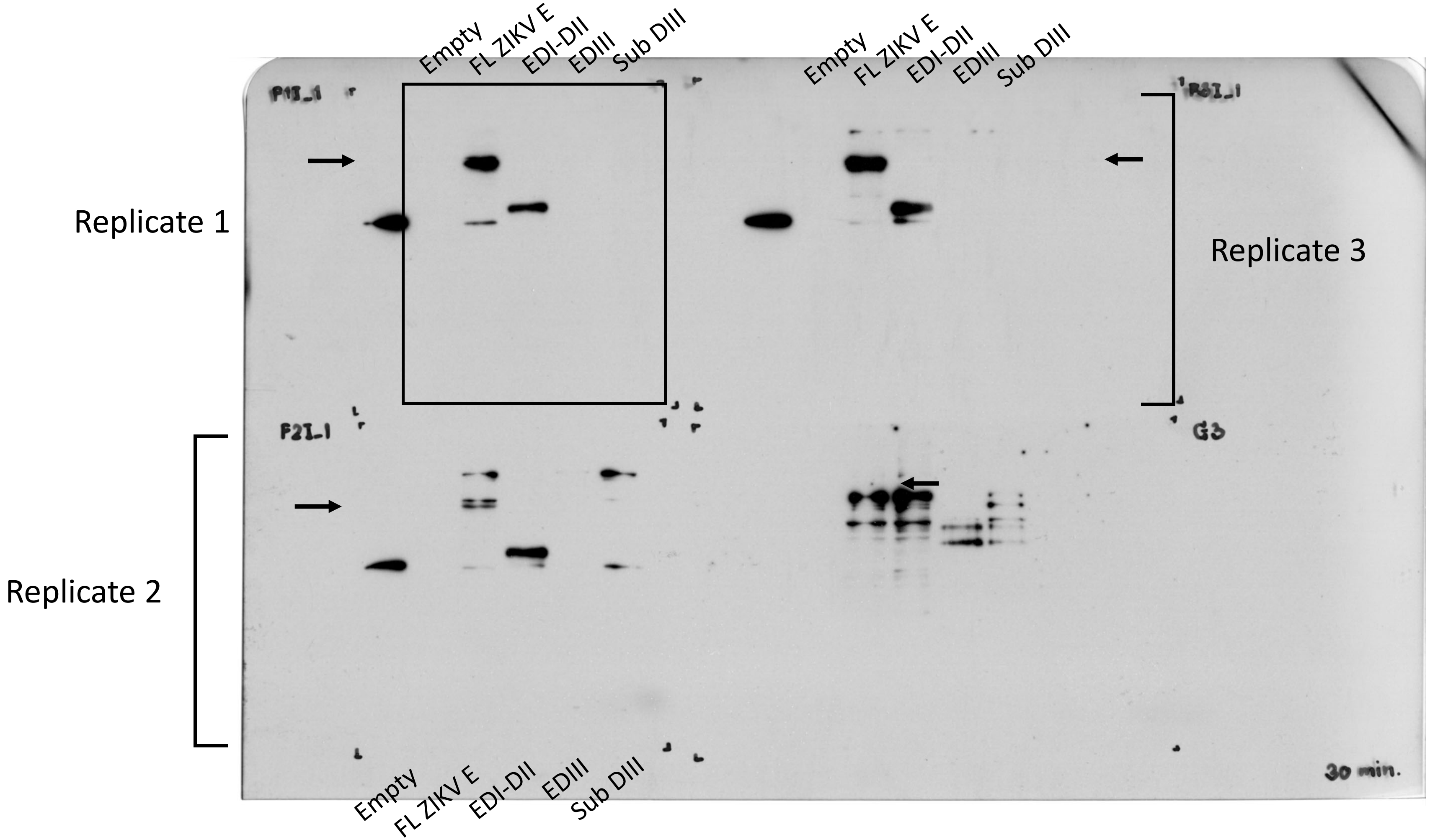

Figure 5B: Input truncated ZIKV E

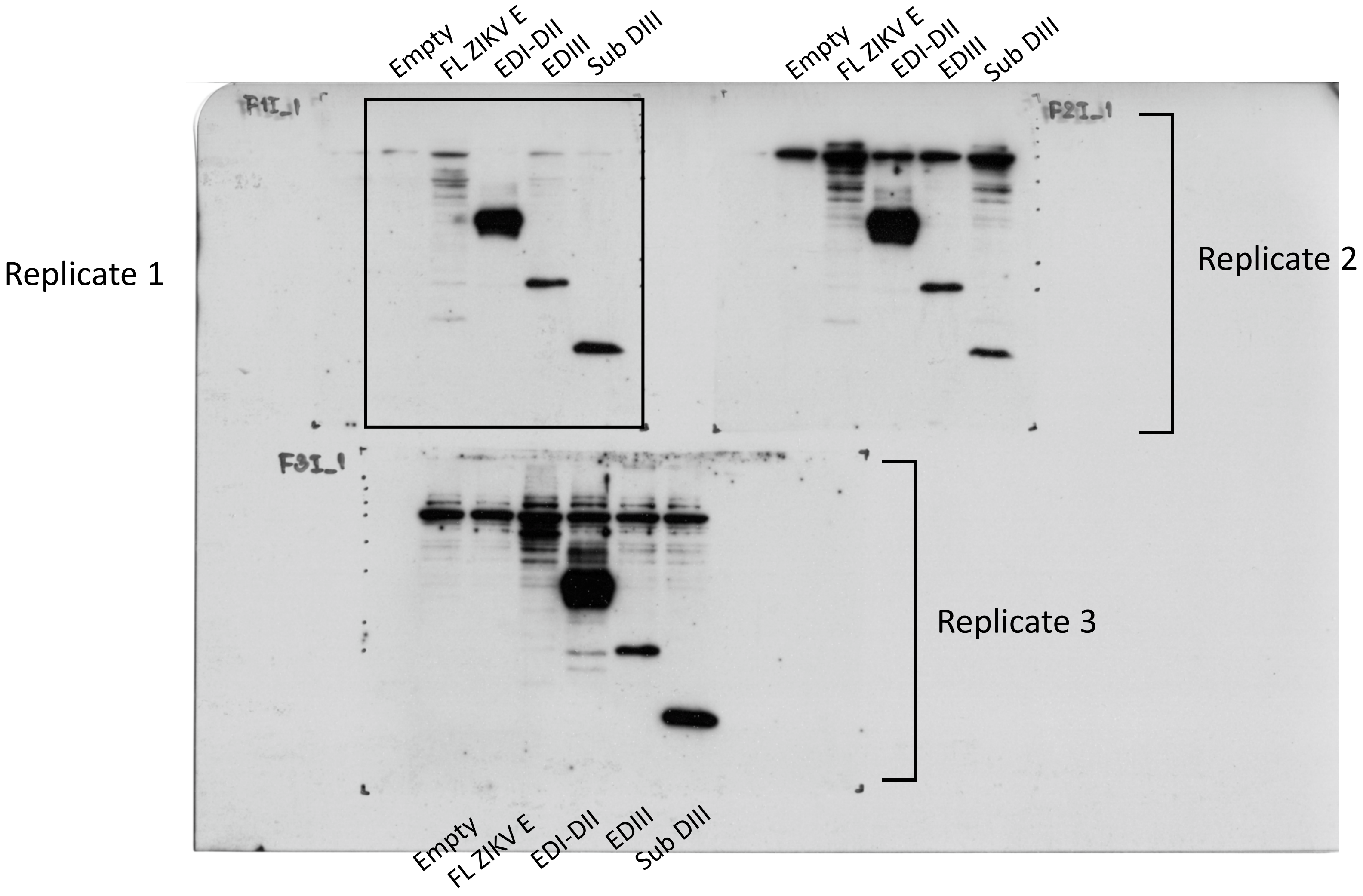

Figure 5B: Input Actin

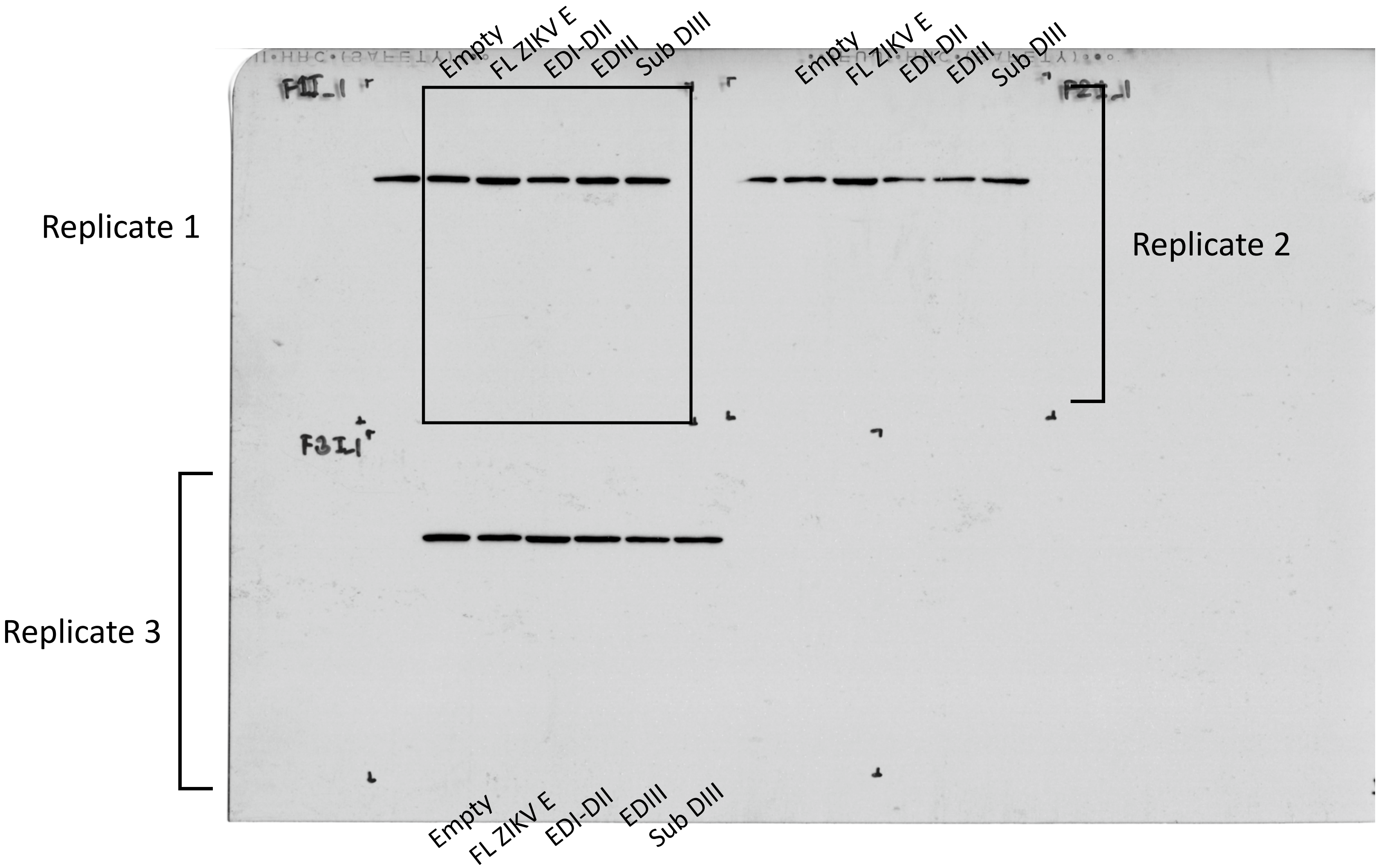

Figure 5B: Output EGFP-GRP78

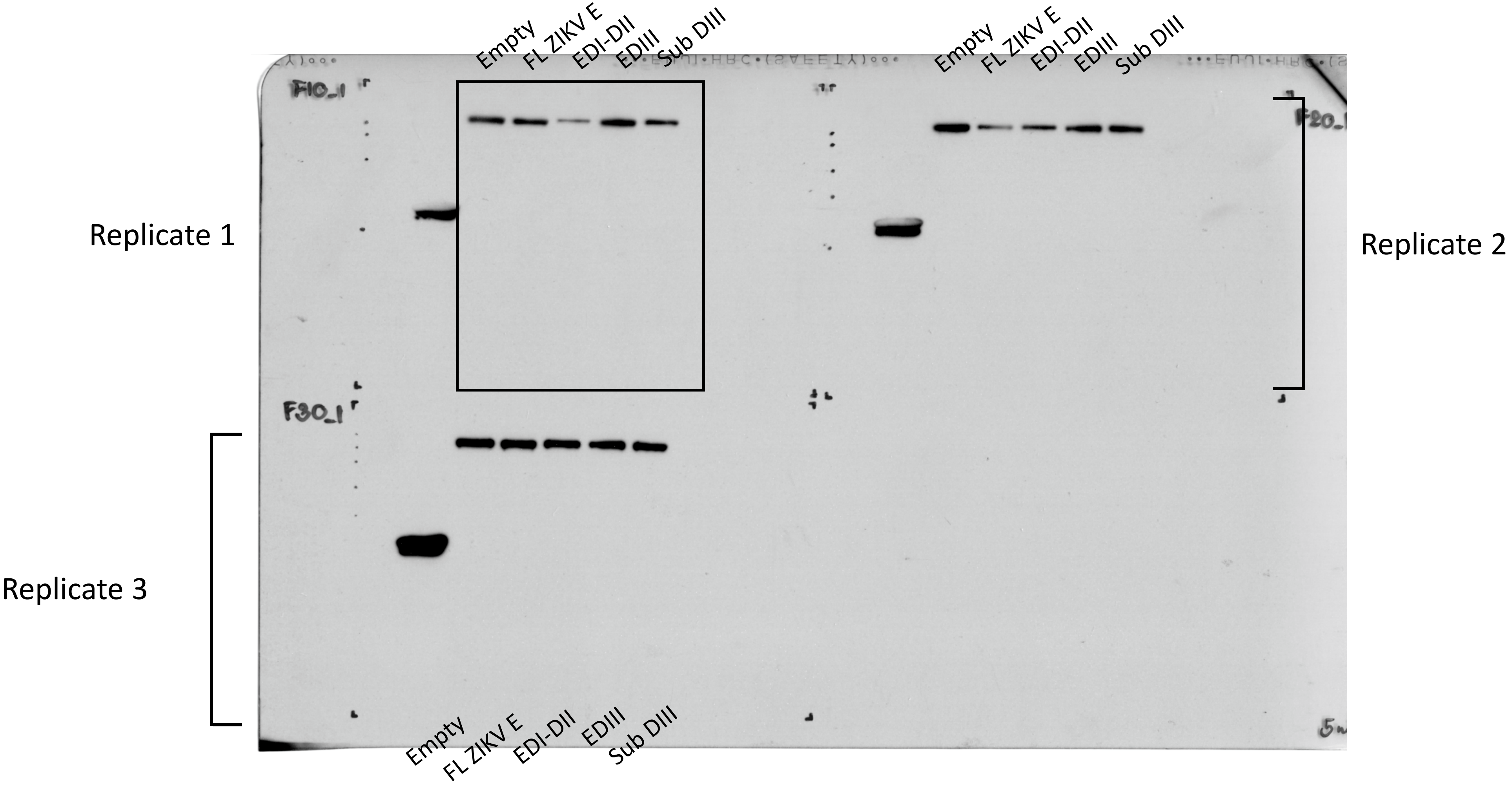

Figure 5B: Output full length ZIKV E

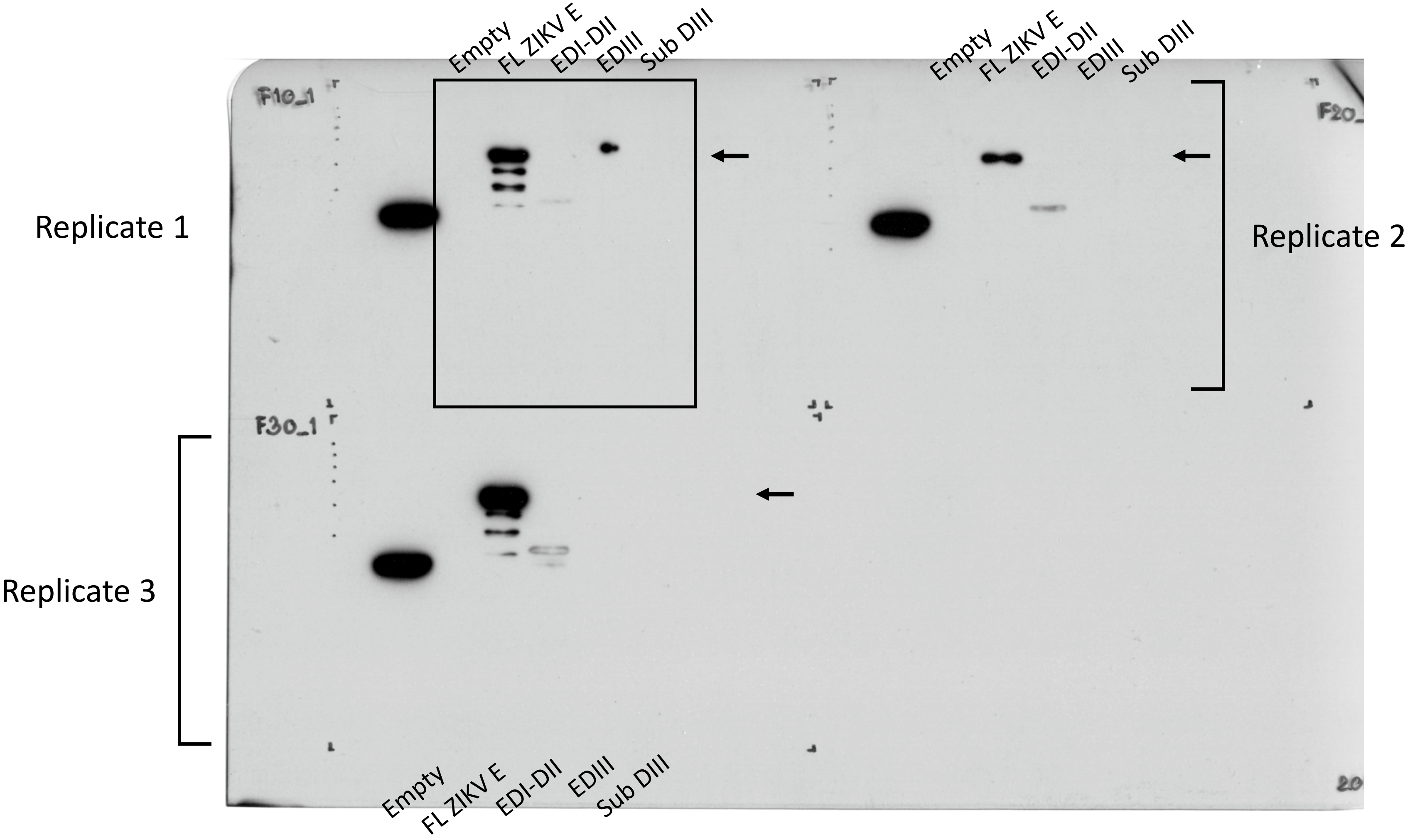

Figure 5B: Output truncated ZIKV E

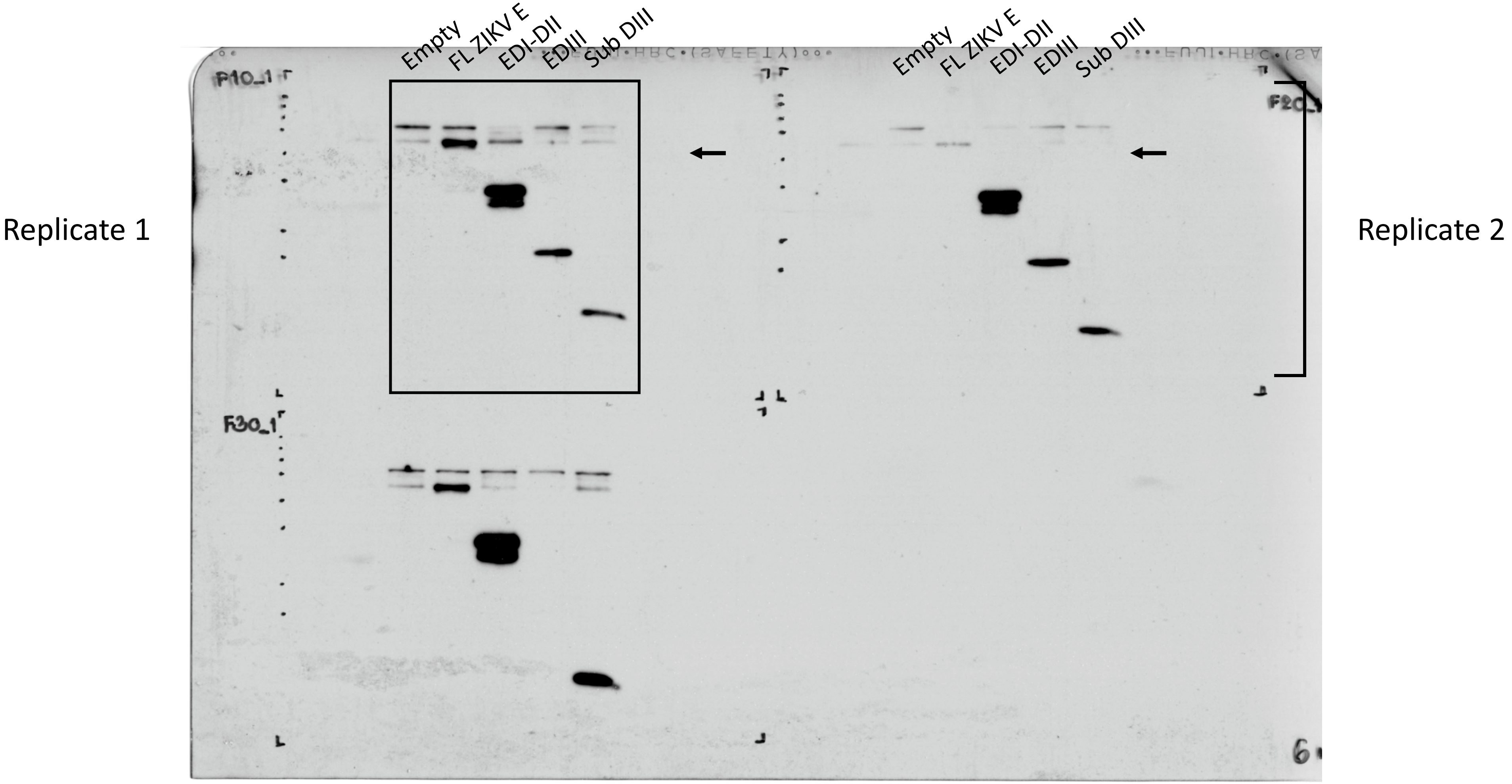

Output truncated ZIKV E replicate 3 of Figure 5B

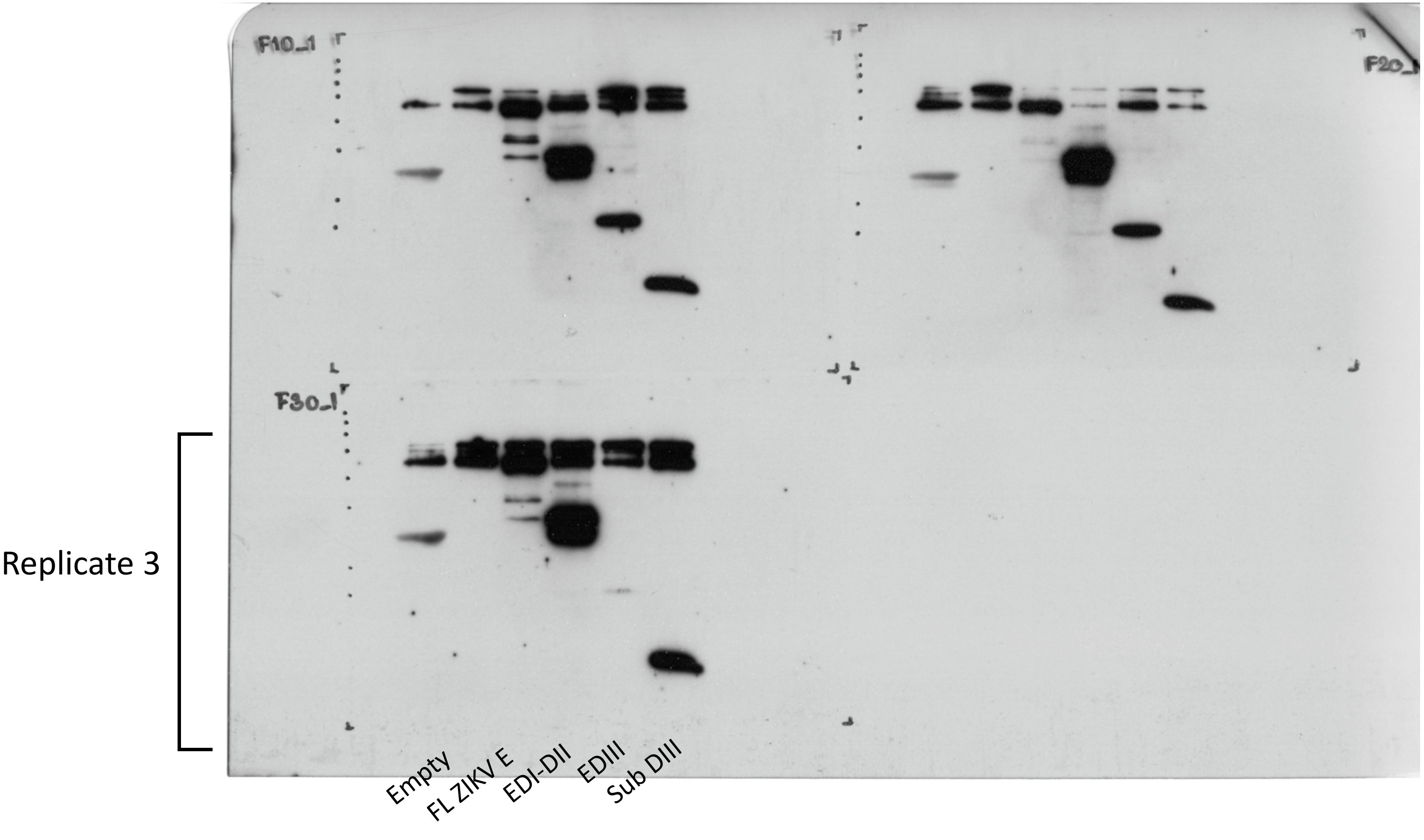

Figure 5D: Input EGFP-GRP78

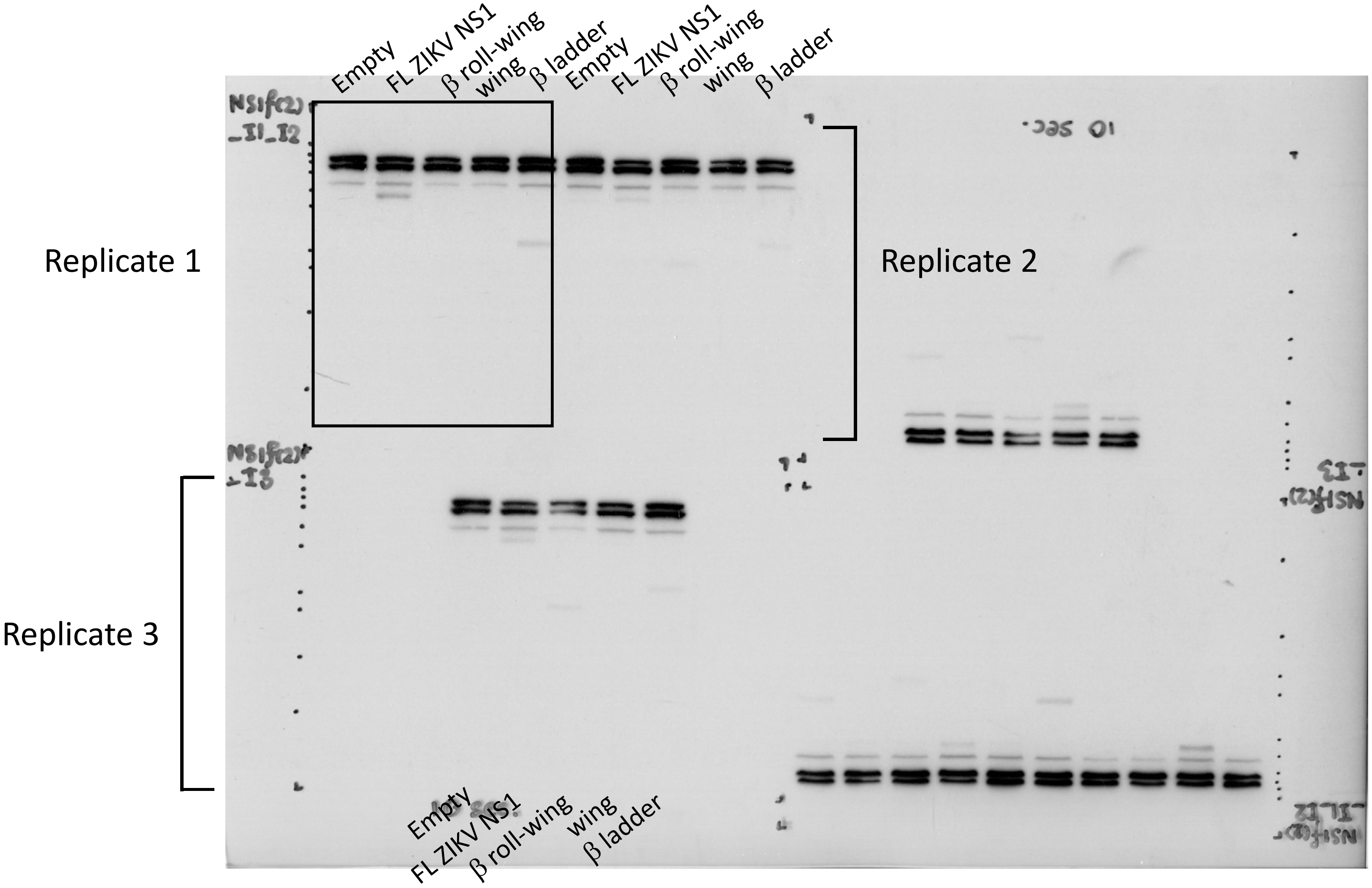

Figure 5D: Input full length ZIKV NS1

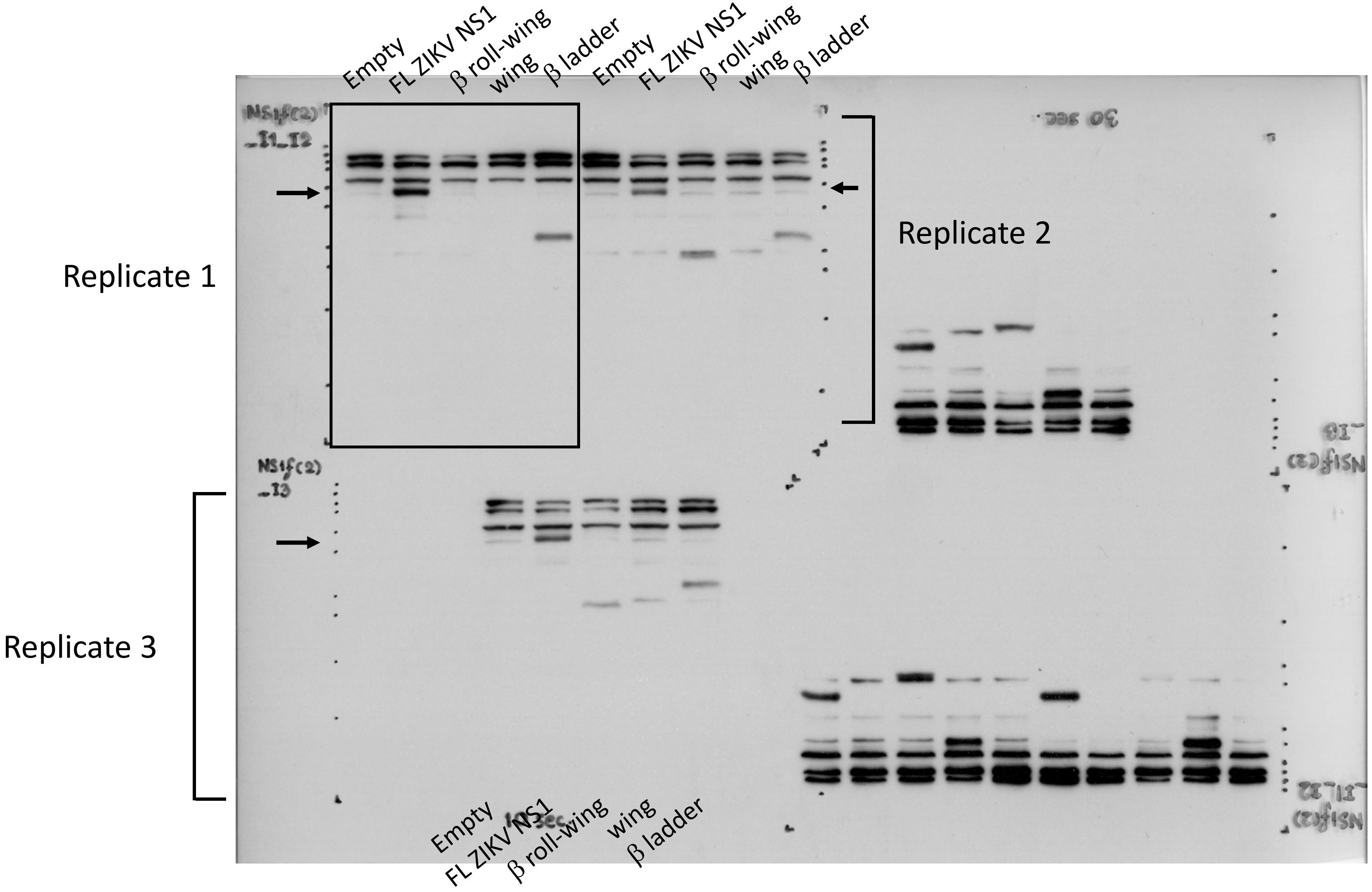

Figure 5D: Input truncated ZIKV NS1

Replicate 1

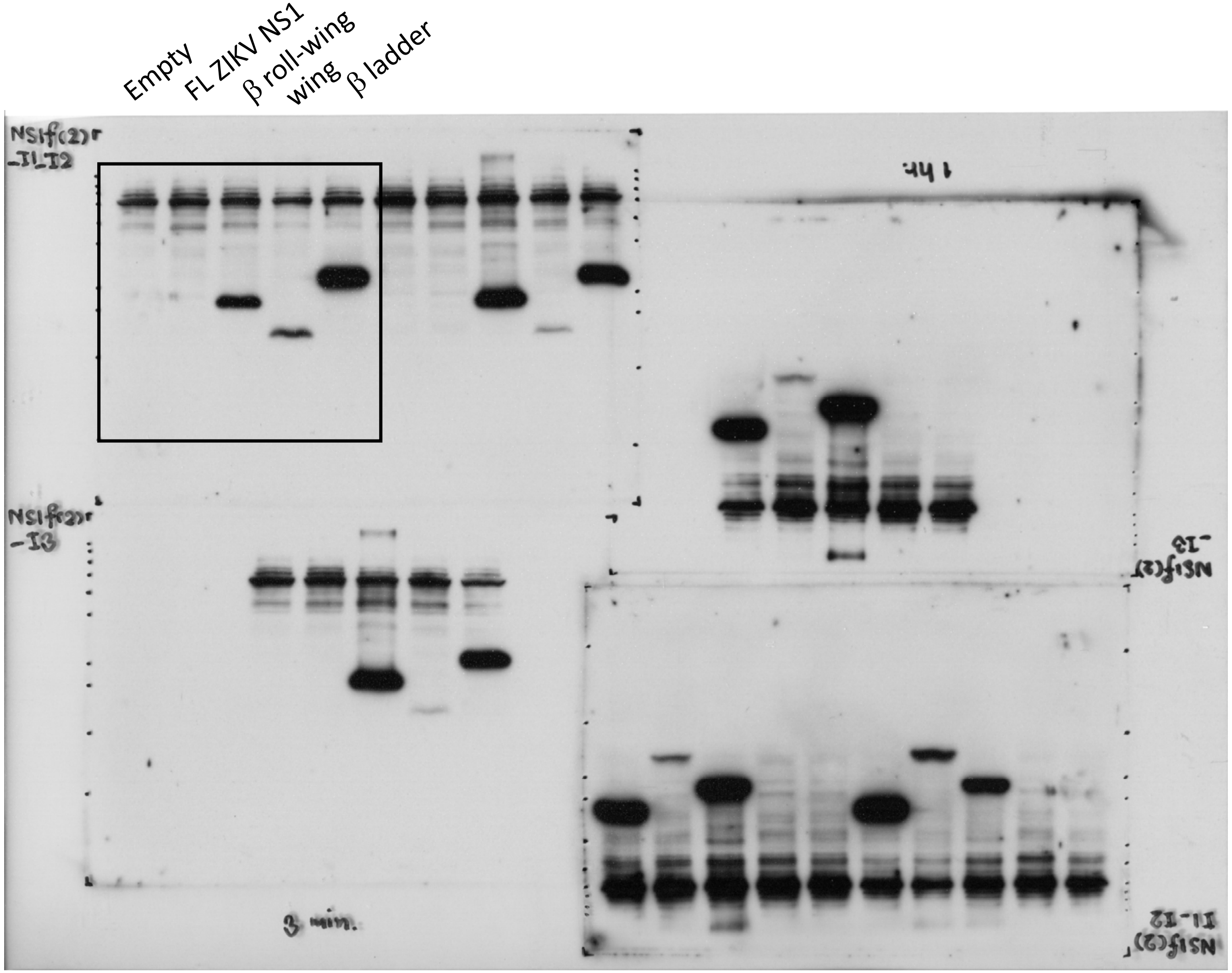

Input truncated ZIKV NS1 replicate 2 and 3 of Figure 5D

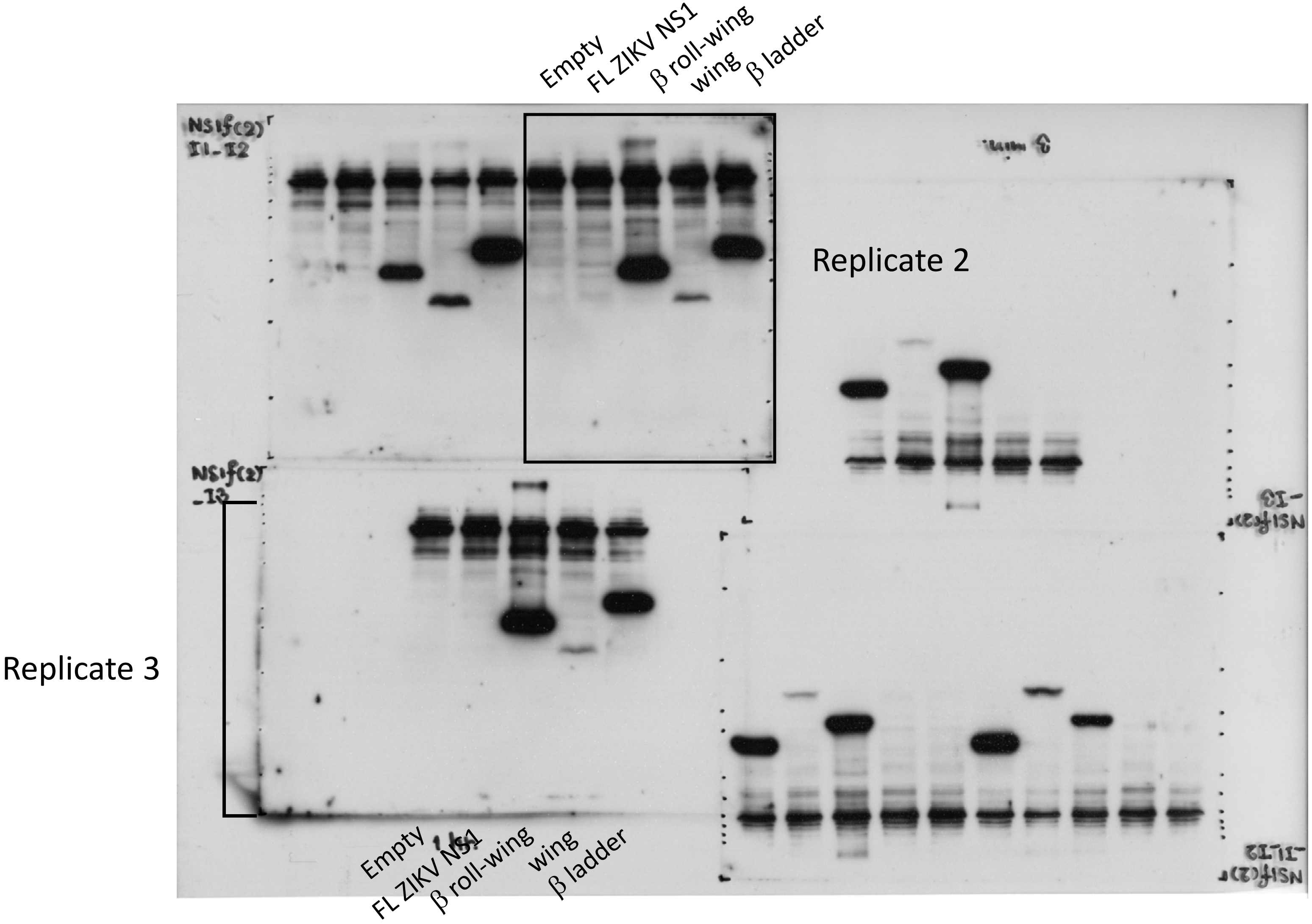

Figure 5D: Input Actin

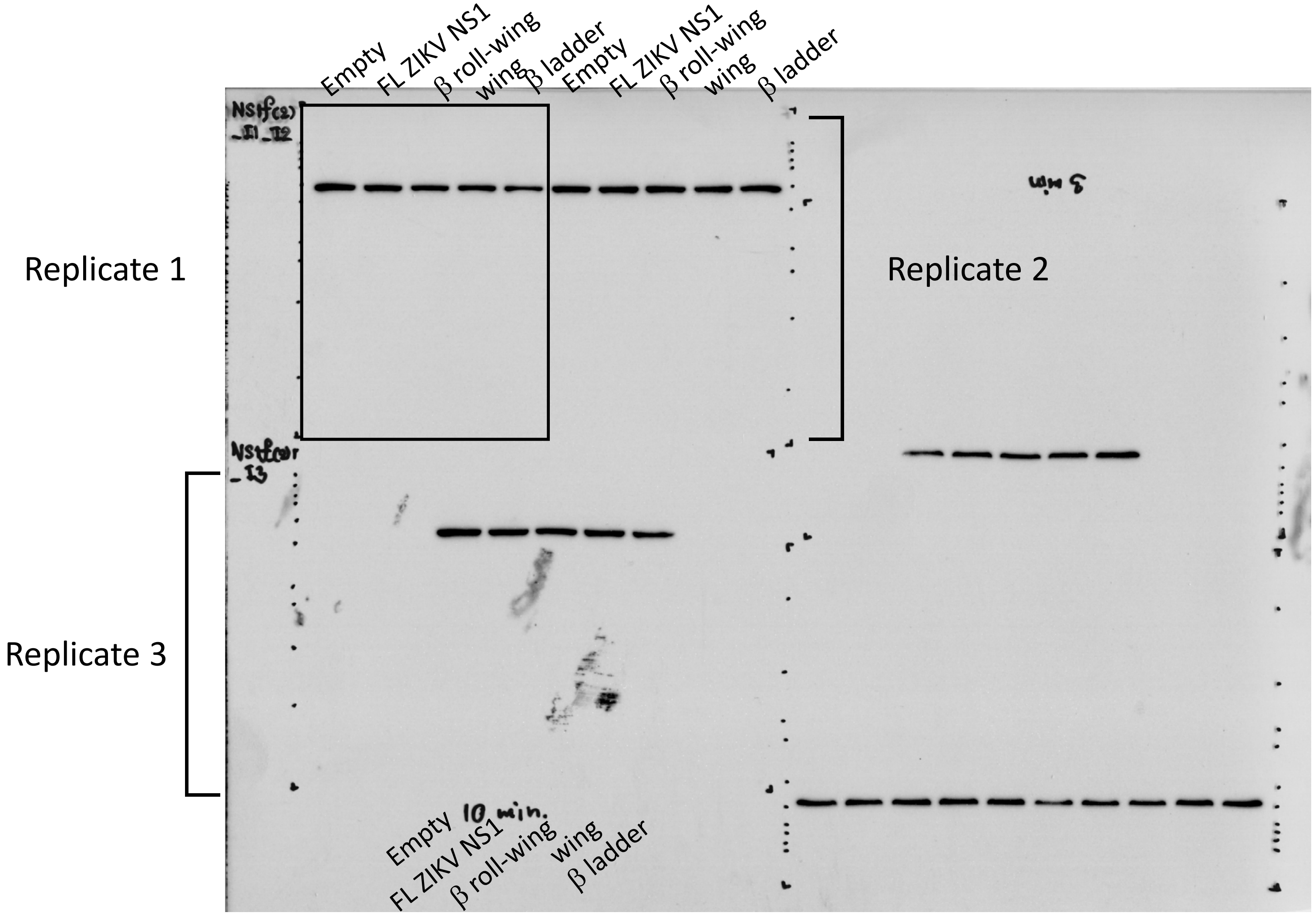

Figure 5D: Output EGFP-GRP78

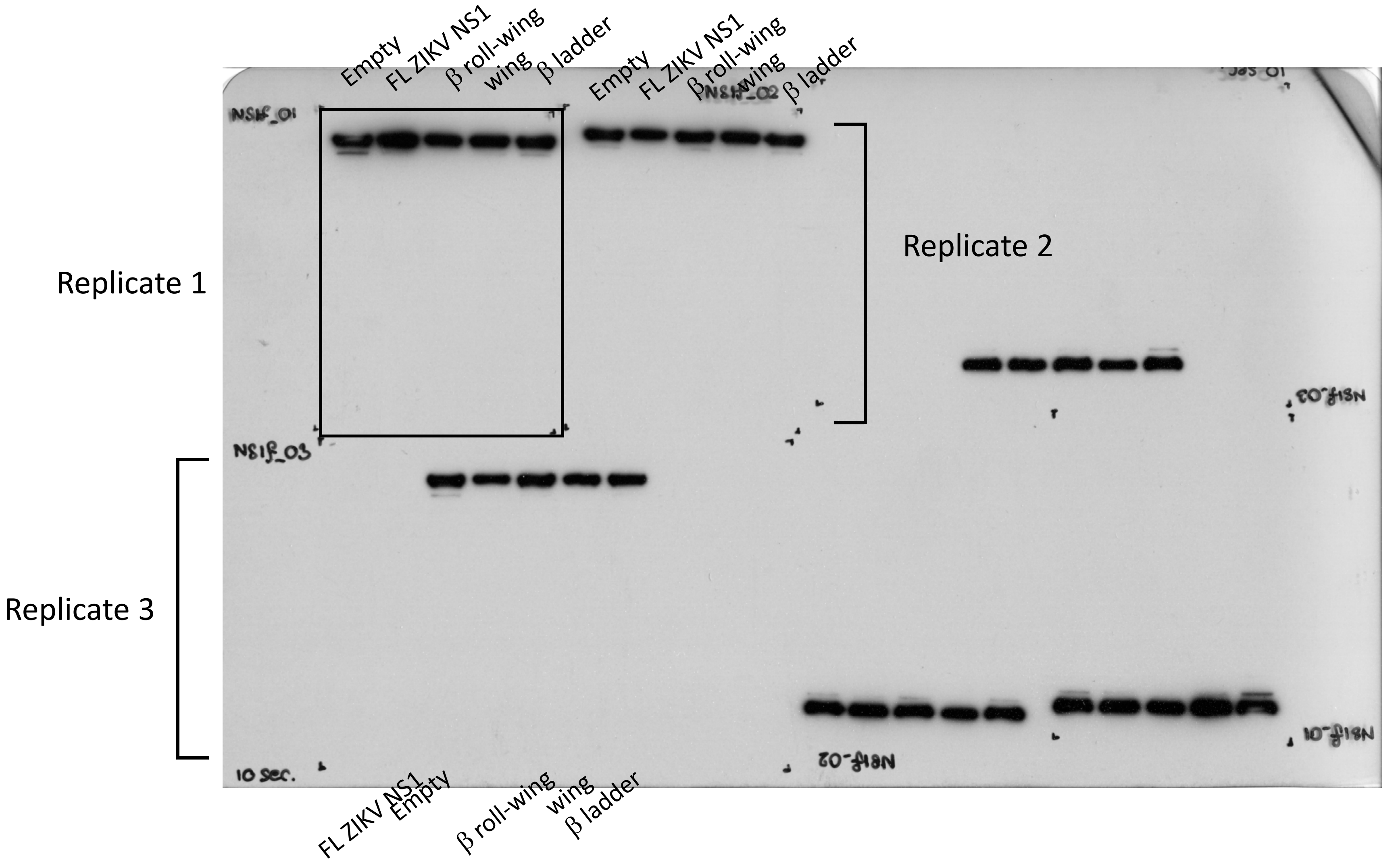

Figure 5D: Output full length ZIKV NS1

Replicate 1

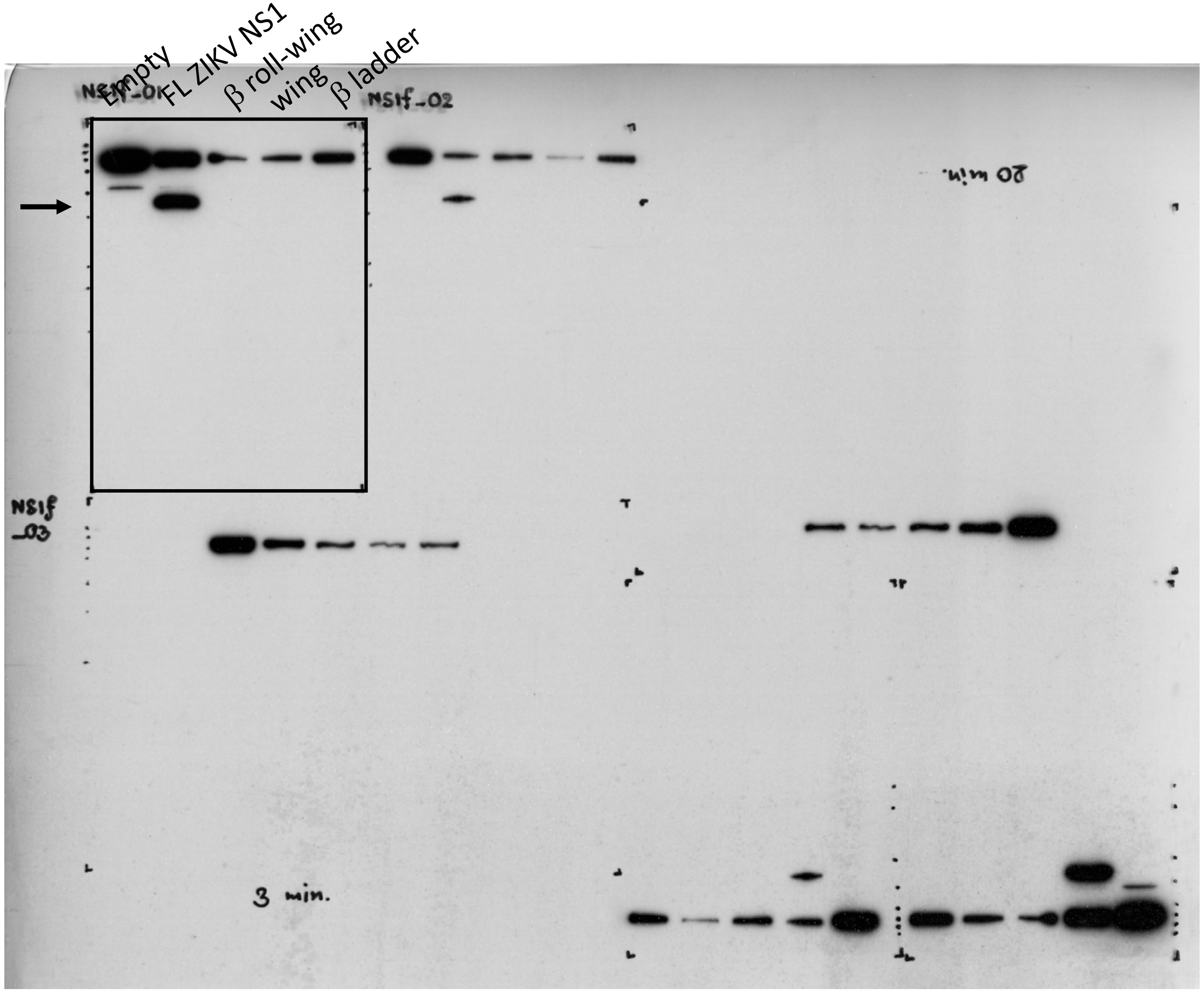

Output full length ZIKV NS1 replicate 2 and 3 of Figure 5D

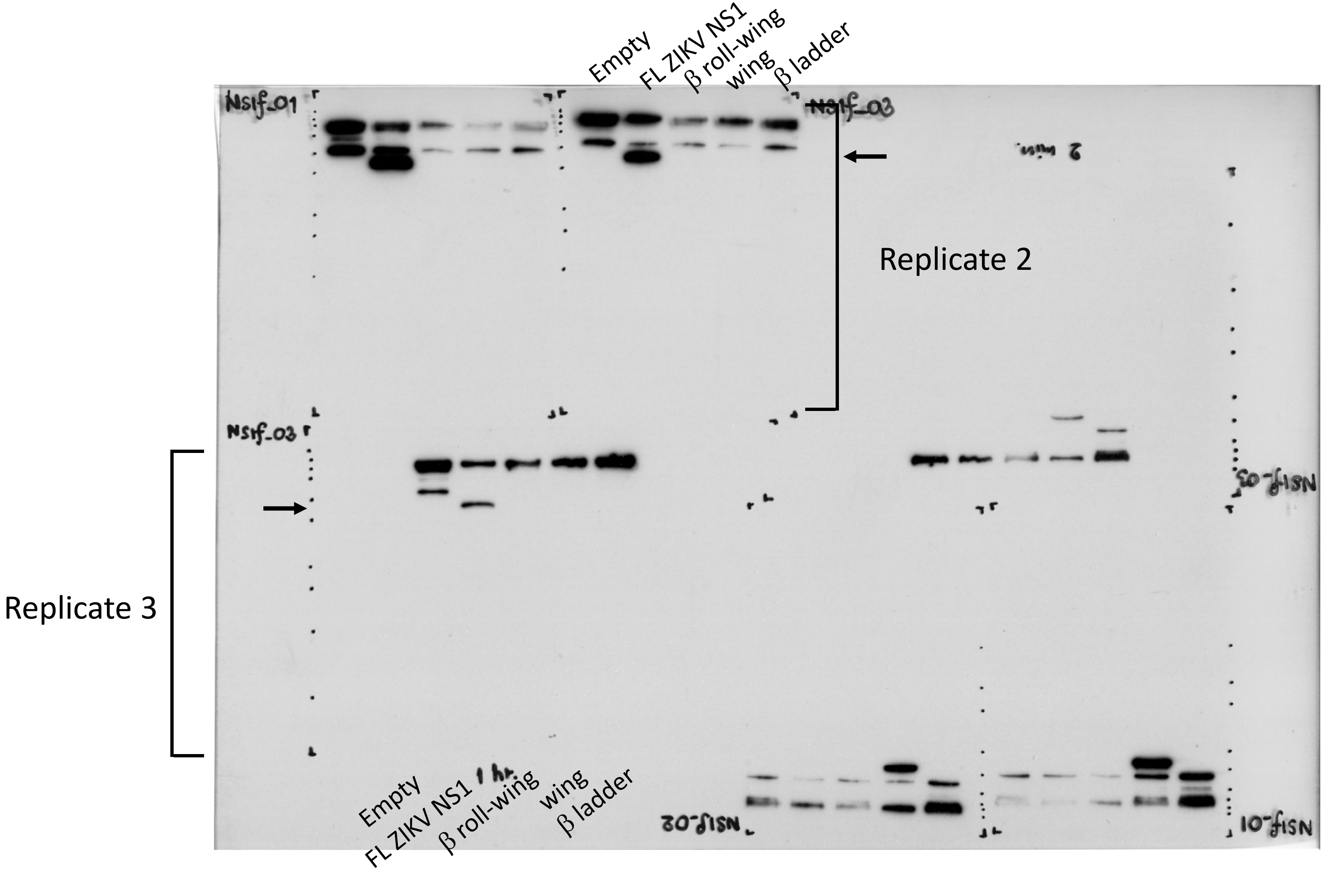

Figure 5D: Output truncated ZIKV NS1

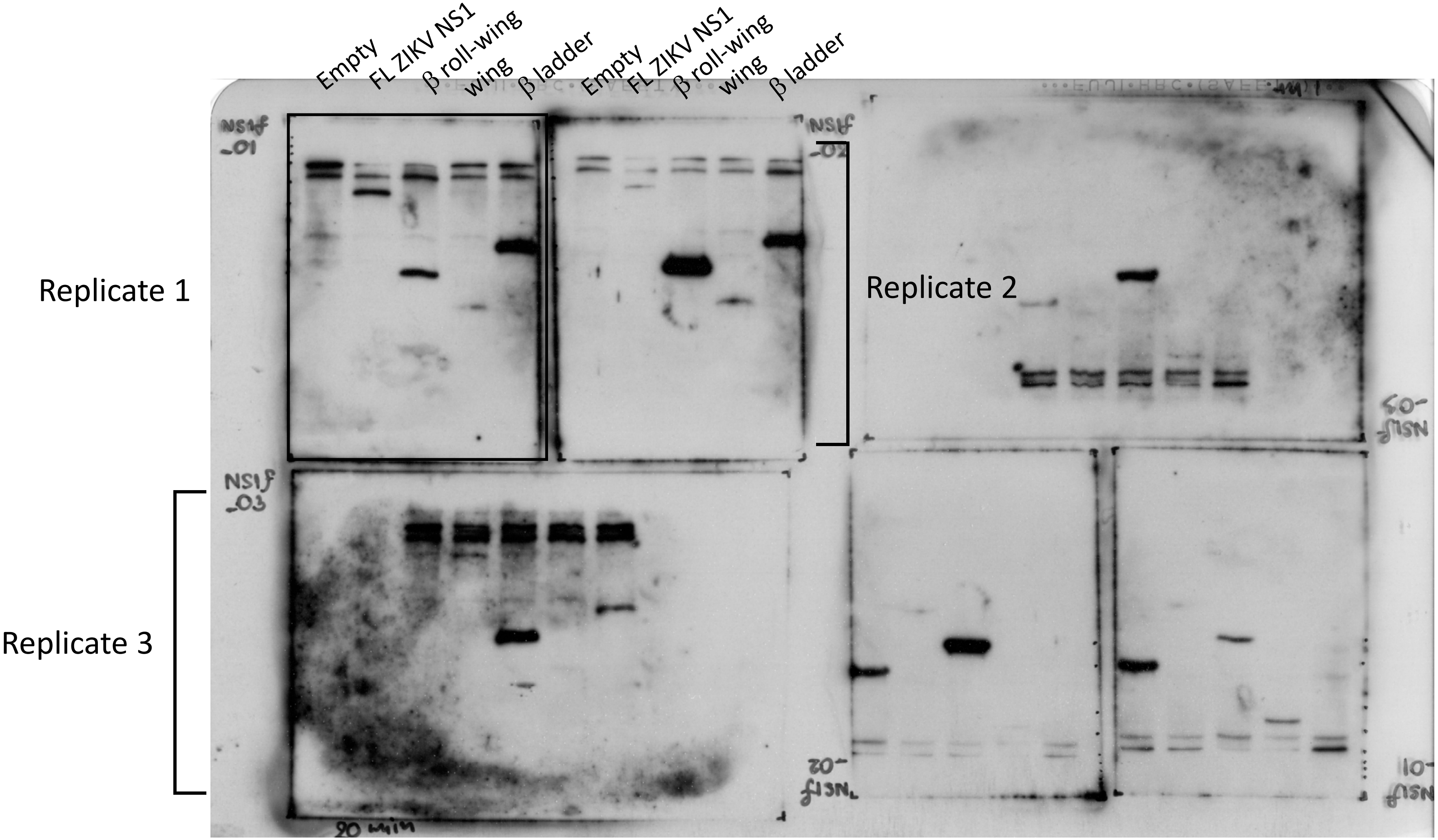

Figure 6C: ZIKV E at 24 h

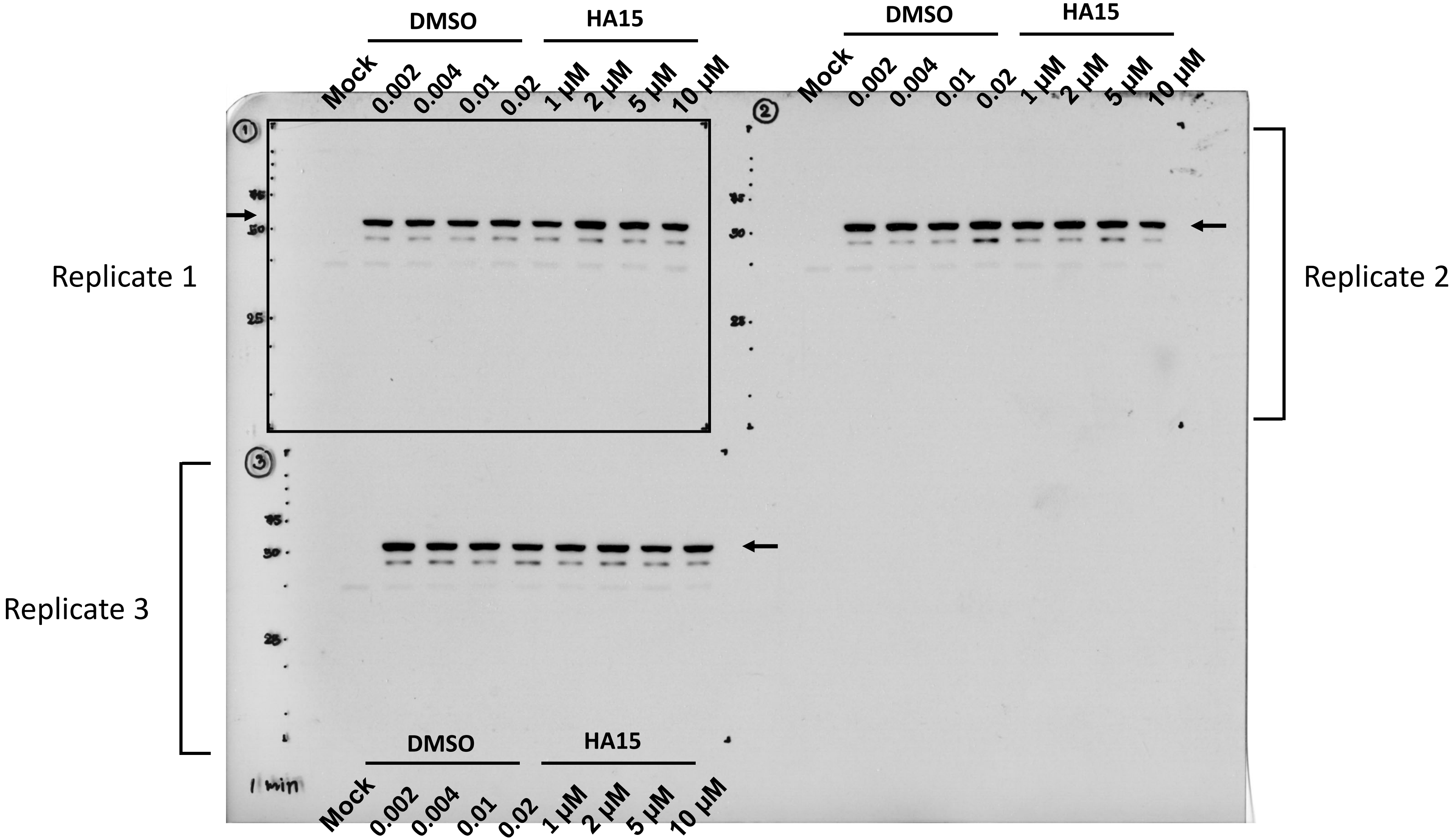

Figure 6C: Actin at 24 h

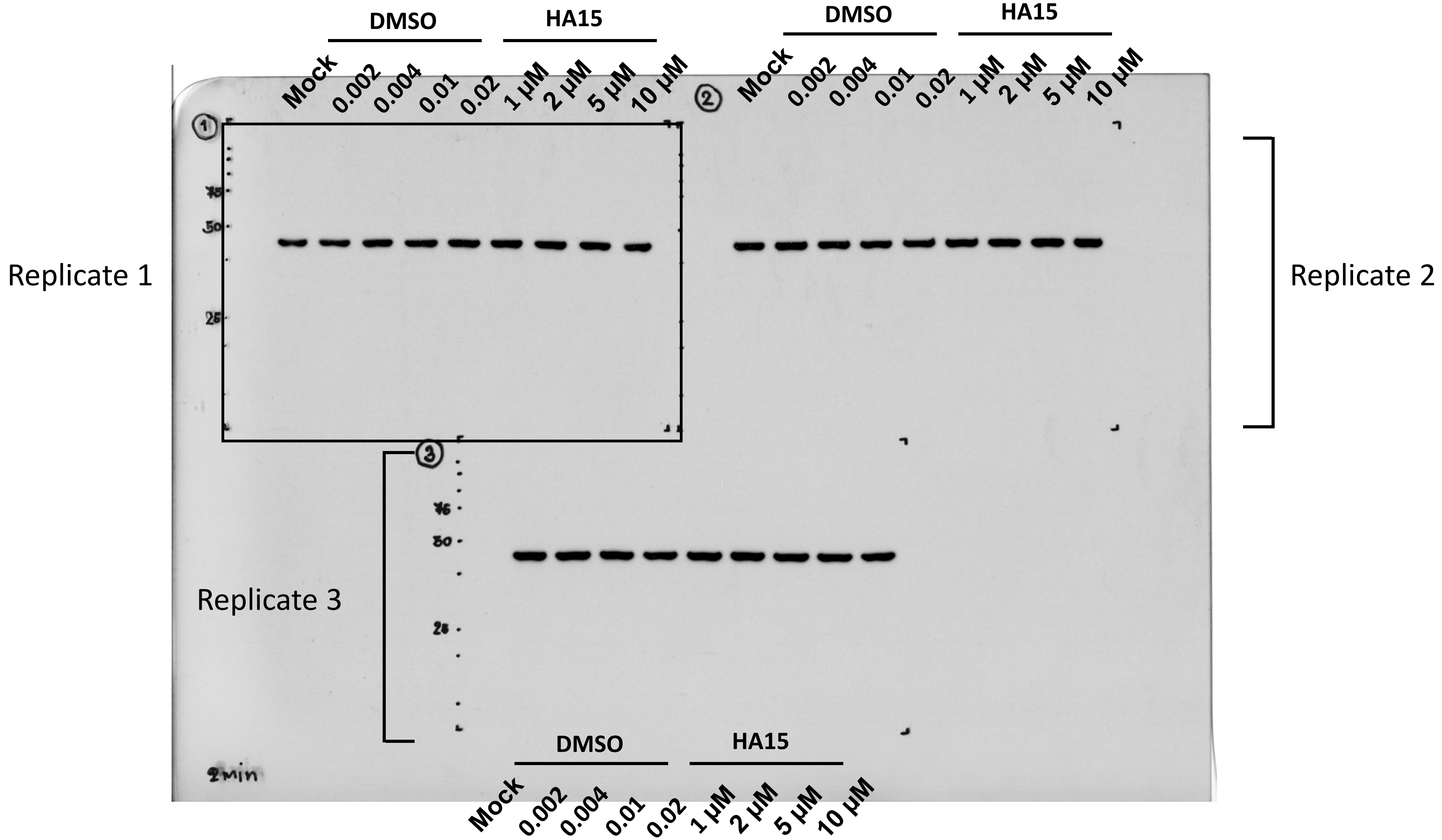

Figure 6C: ZIKV E at 48 h

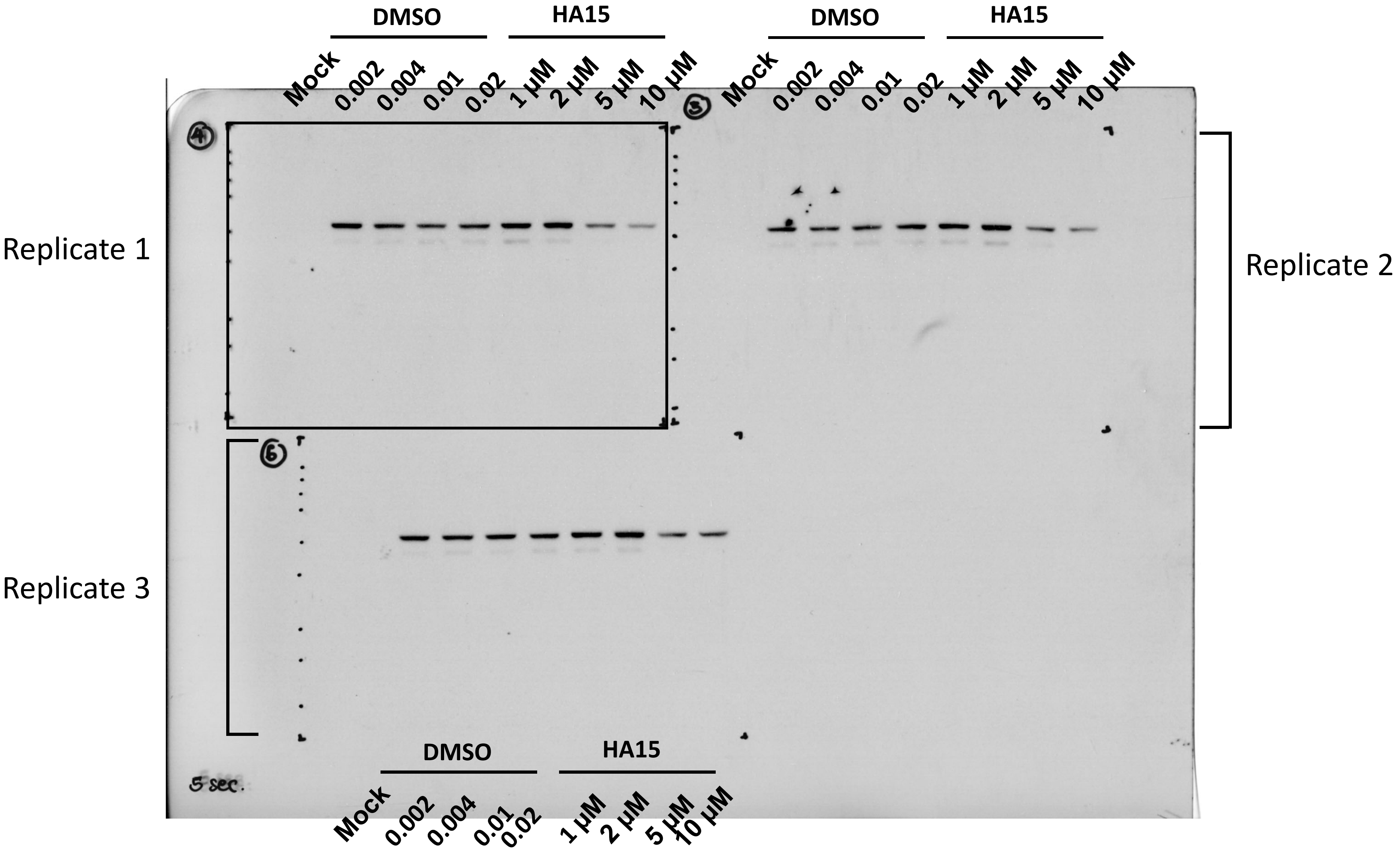

Figure 6C: Actin at 48 h

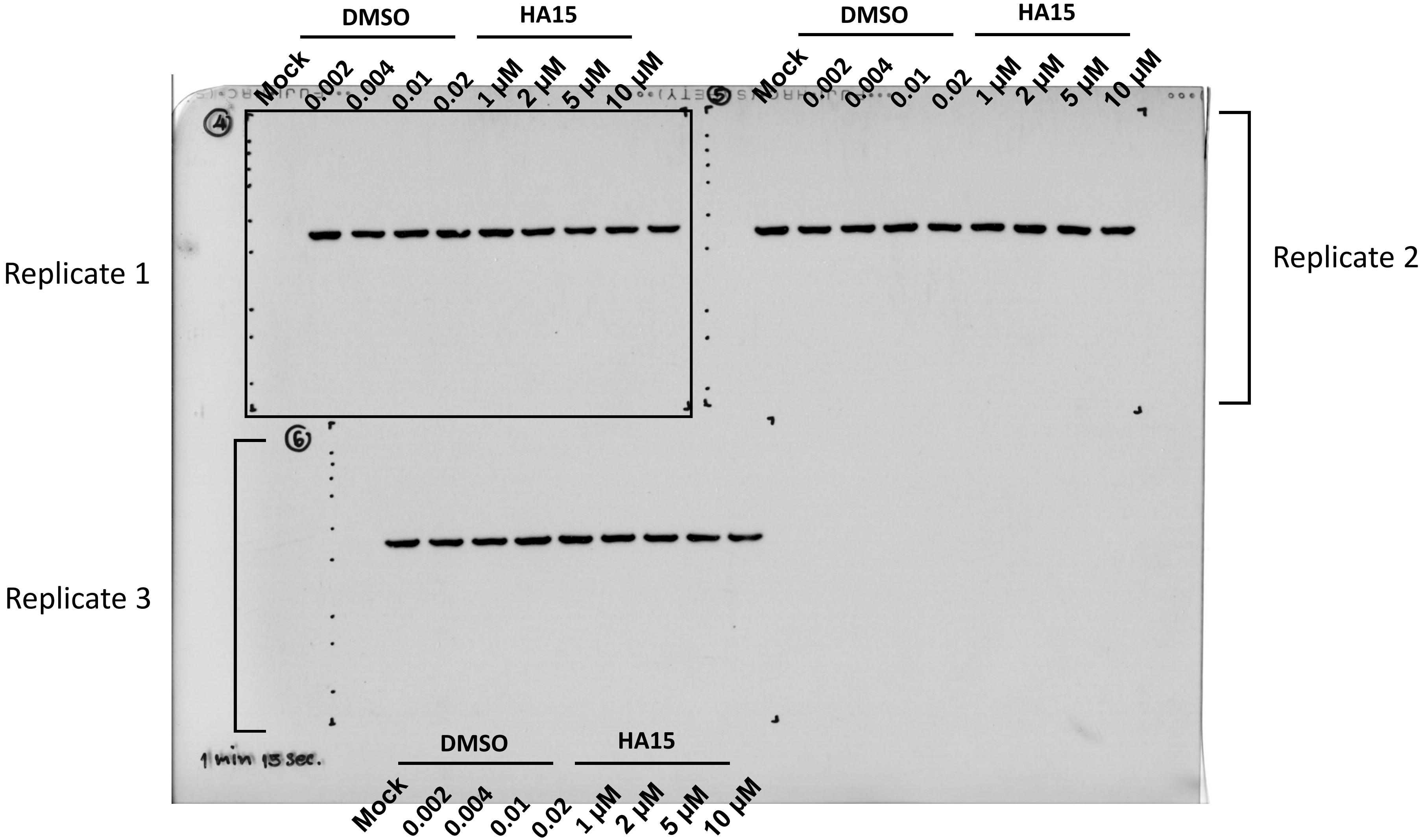

Figure 7D: ZIKV E at 24 h

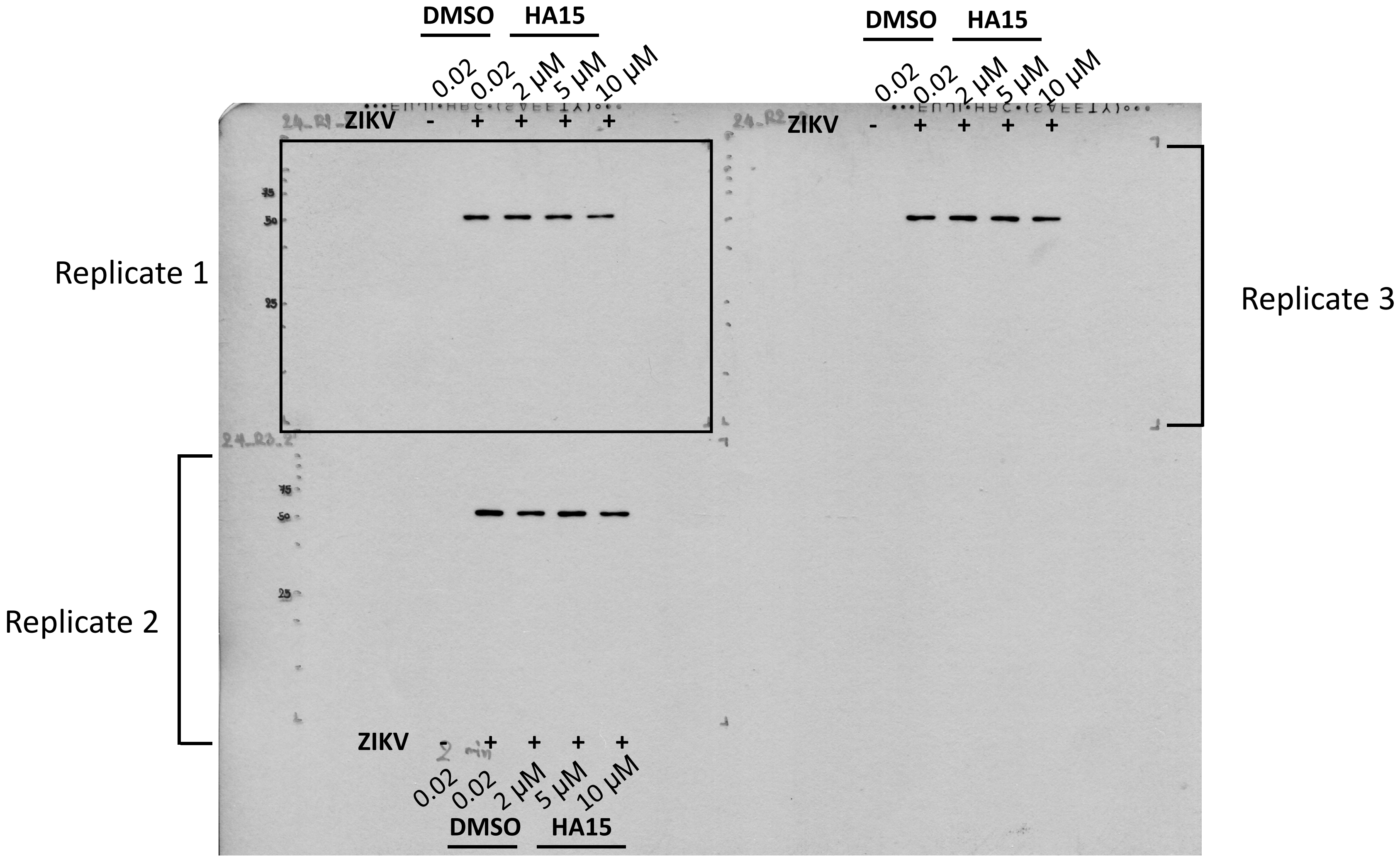

Figure 7D: ZIKV NS1 at 24 h

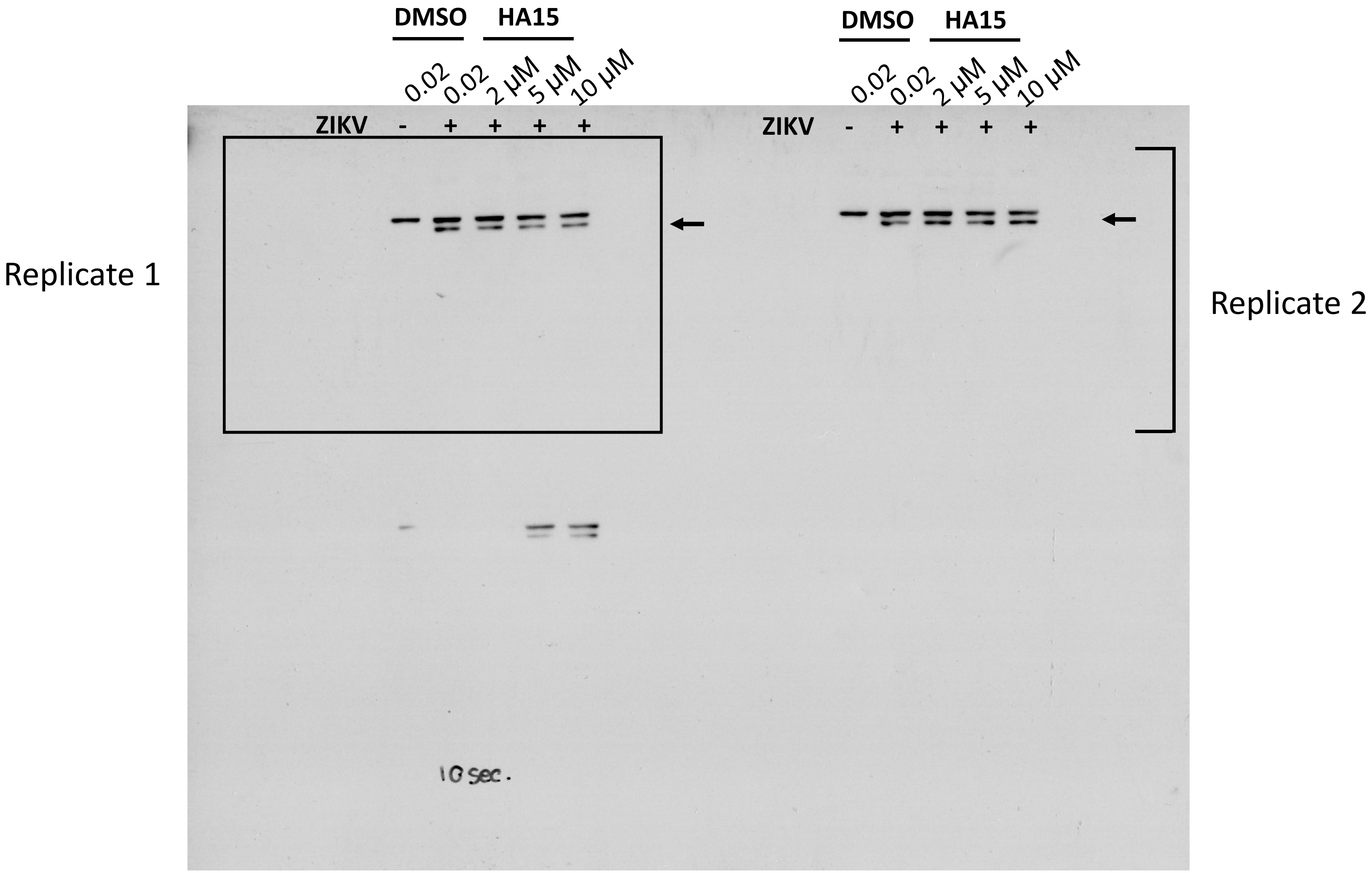

ZIKV NS1 at 24 h replicate 3 of Figure 7D

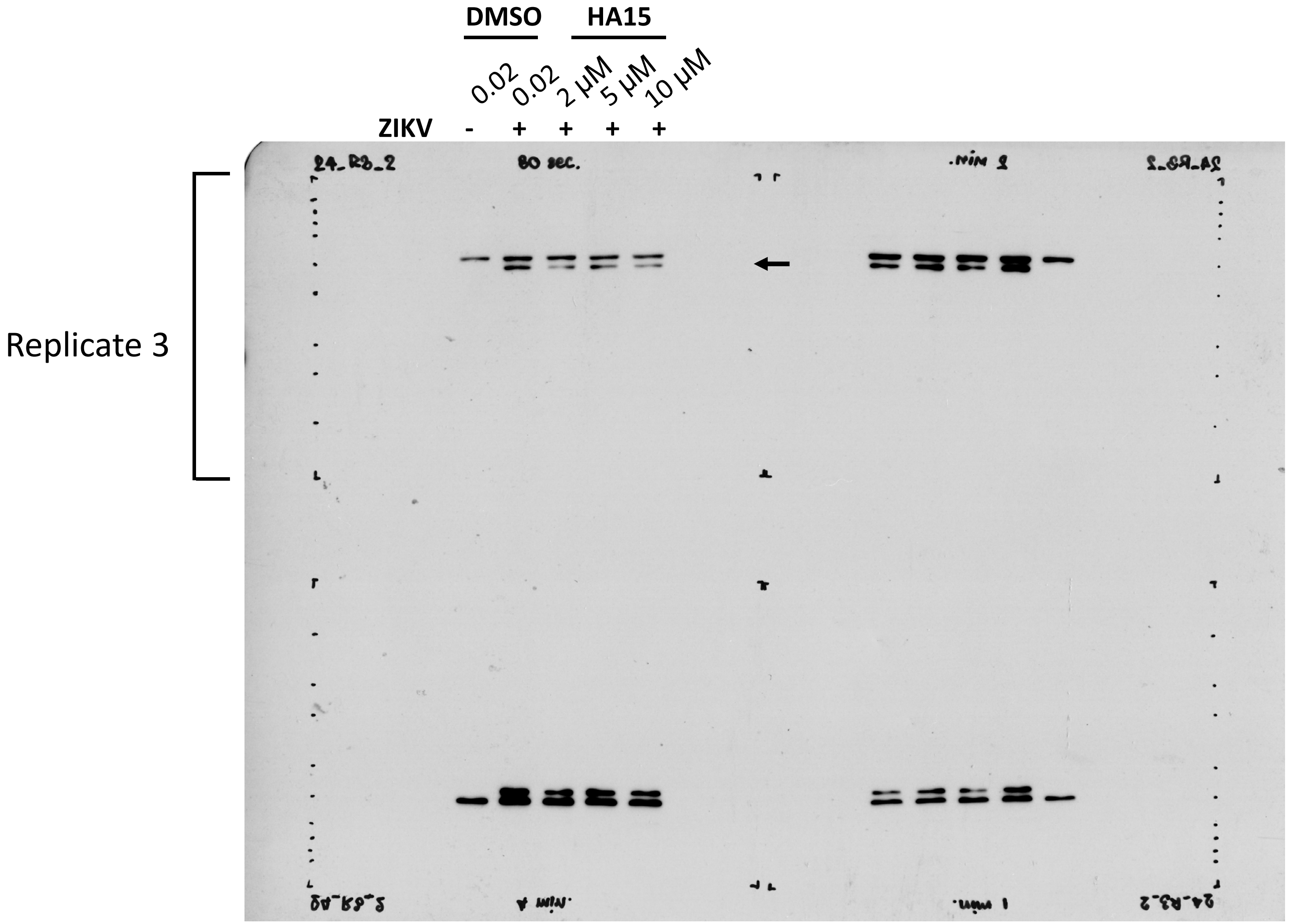

Figure 7D: Actin at 24 h

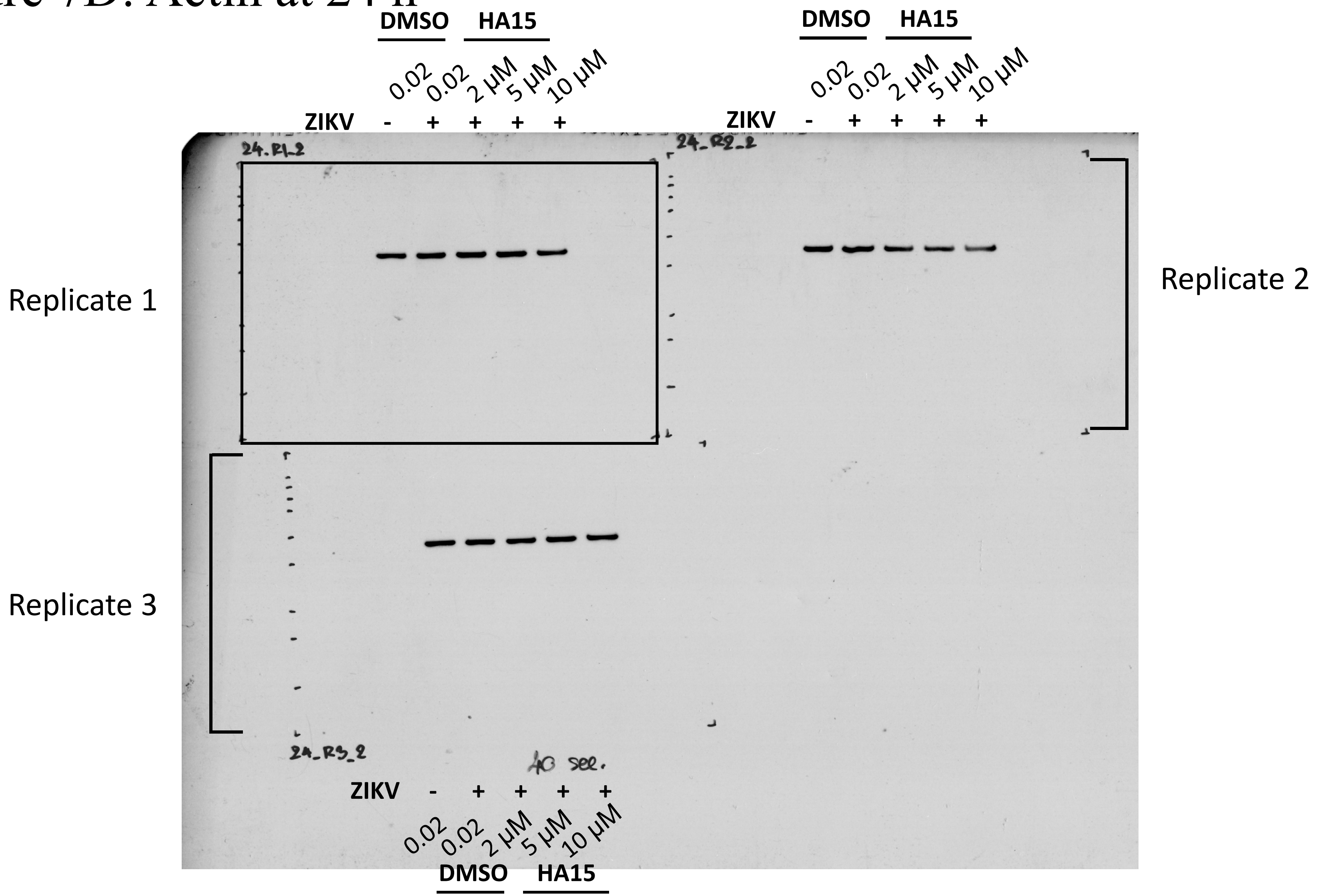

Figure 7D: ZIKV E at 48 h

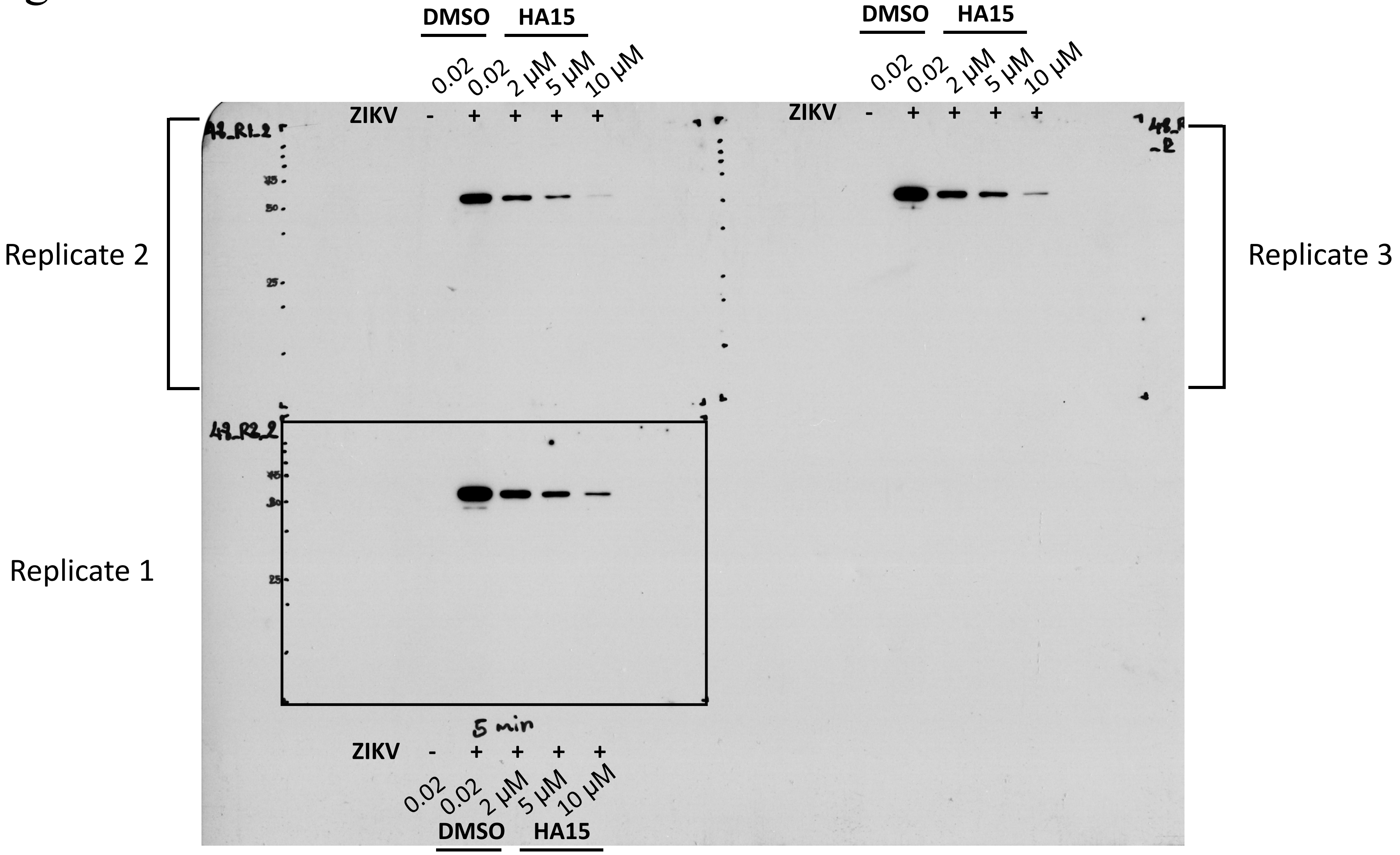

Figure 7D: ZIKV NS1 at 48 h

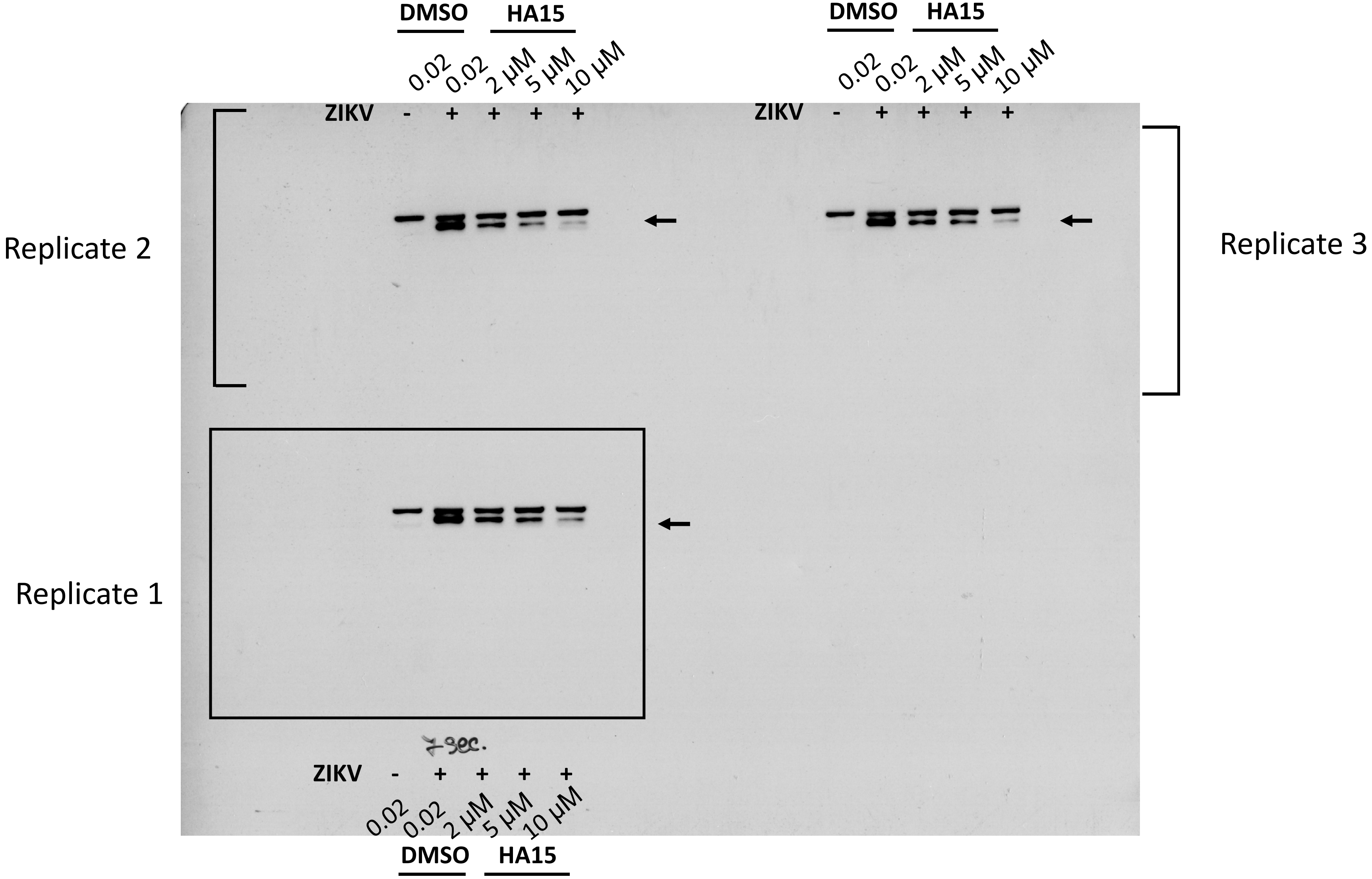

Figure 7D: Actin at 48 h

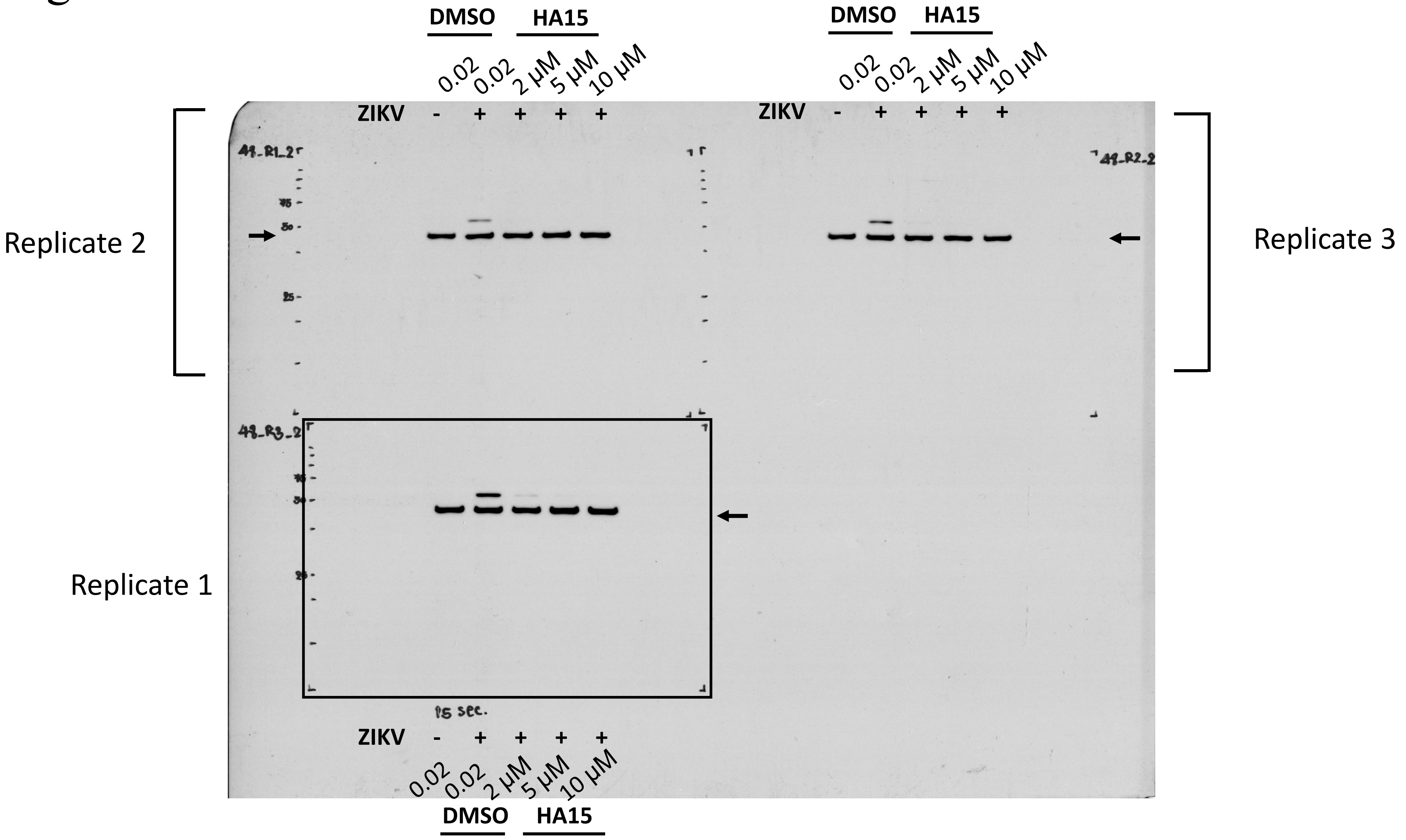

Supplemental Figure: Input EGFP-GRP78 replicate 1

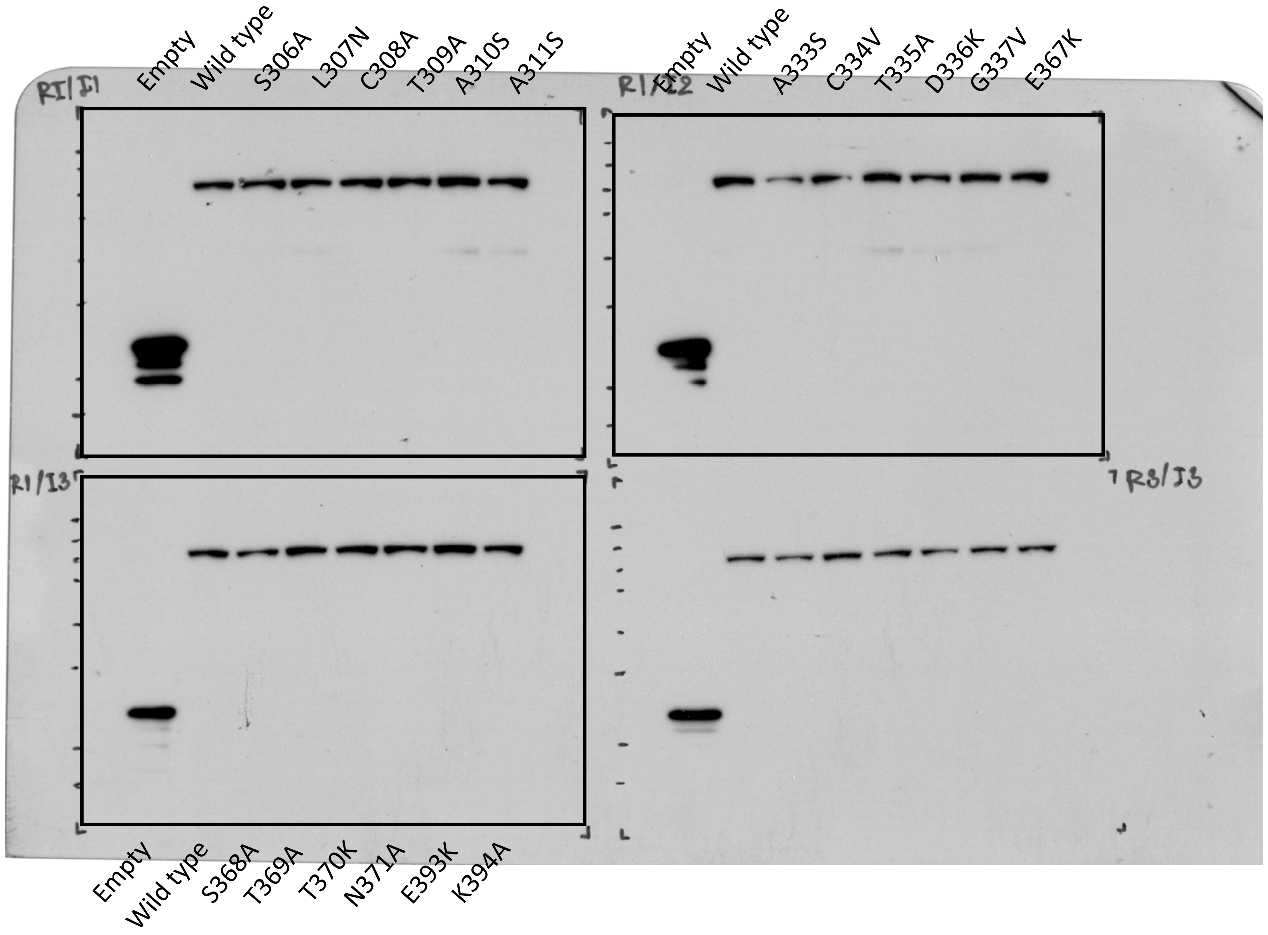

Supplemental Figure: Input ZIKV E-HA replicate 1

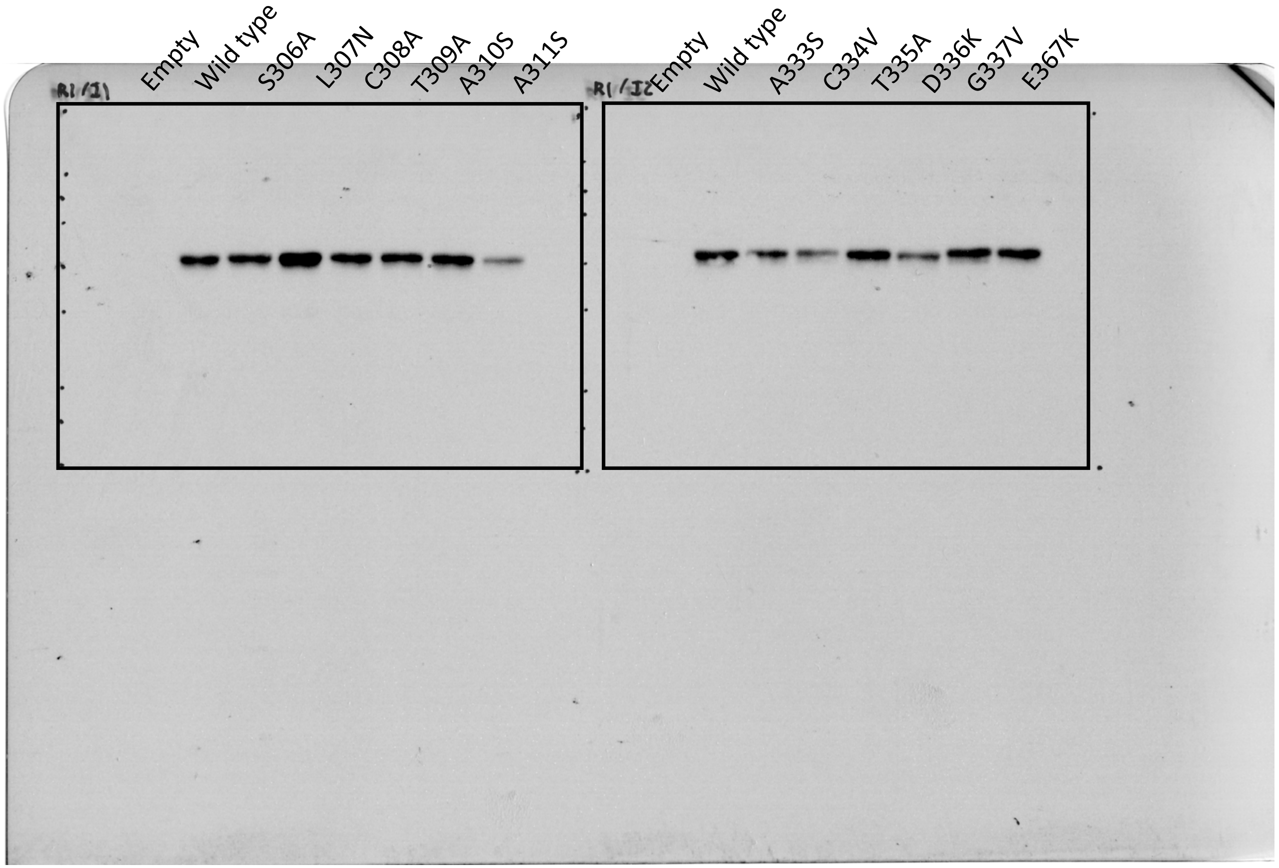

Supplemental Figure: Input ZIKV E-HA replicate 1

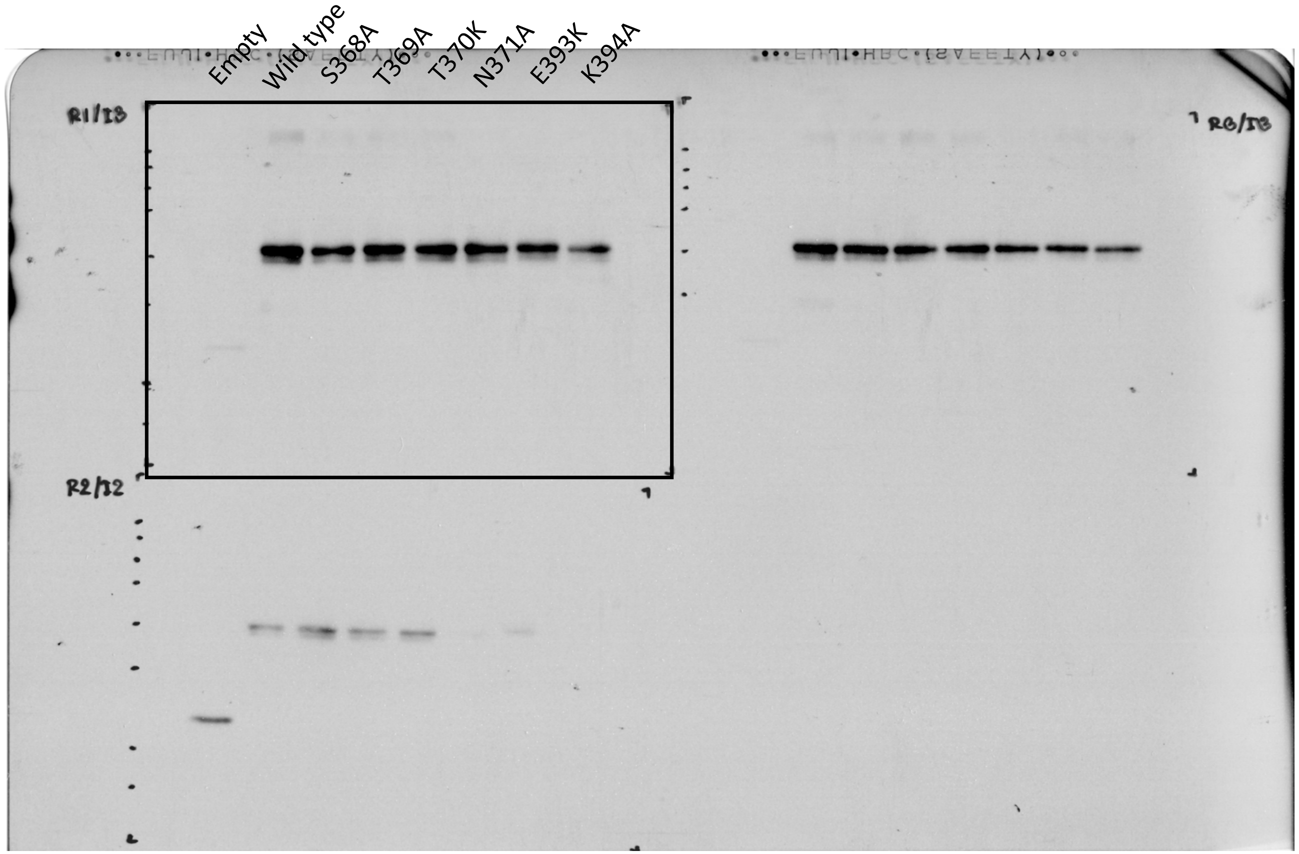

Supplemental Figure: Input Actin replicate 1

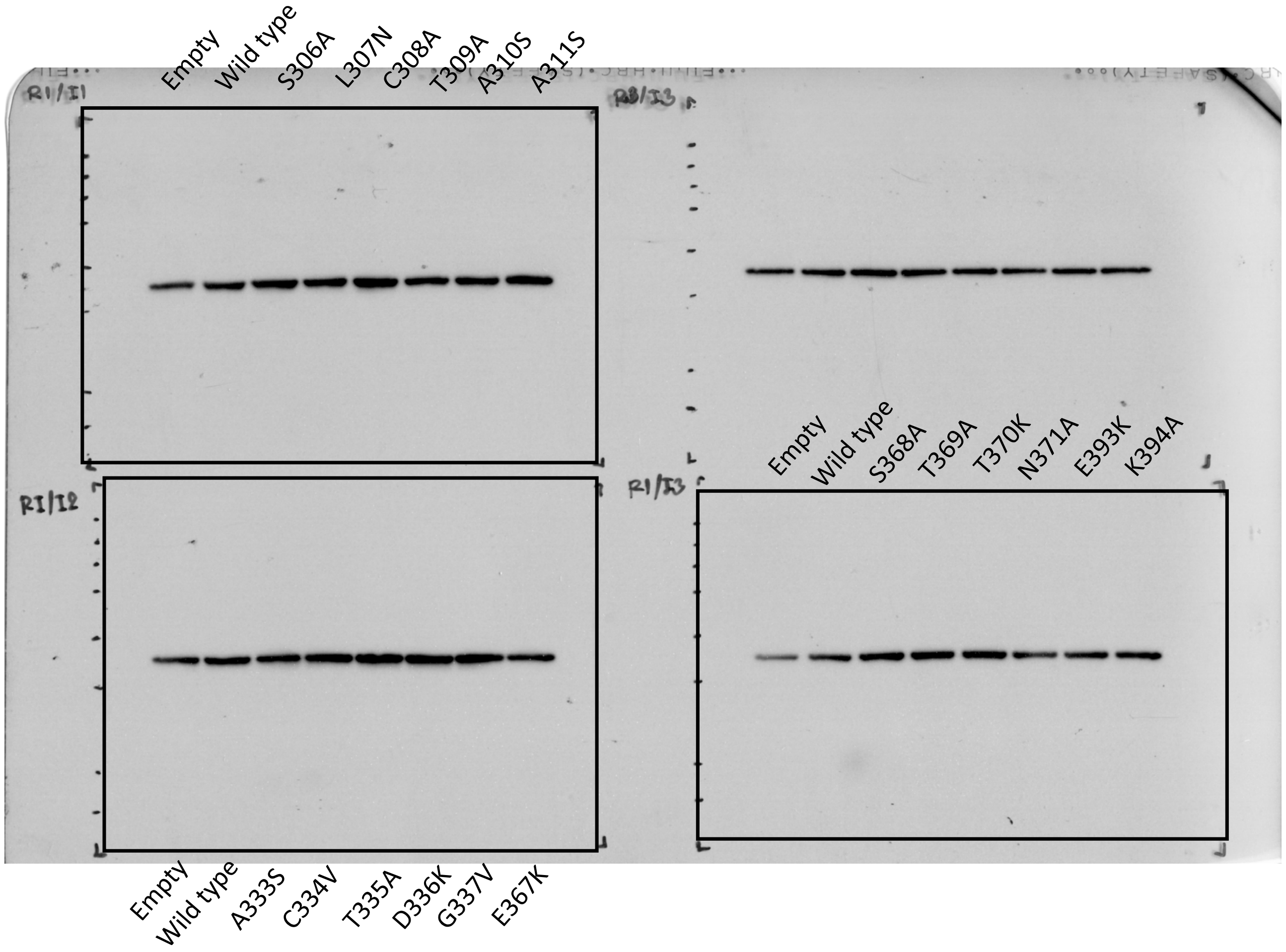

Supplemental Figure: Output ZIKV E-HA replicate 1

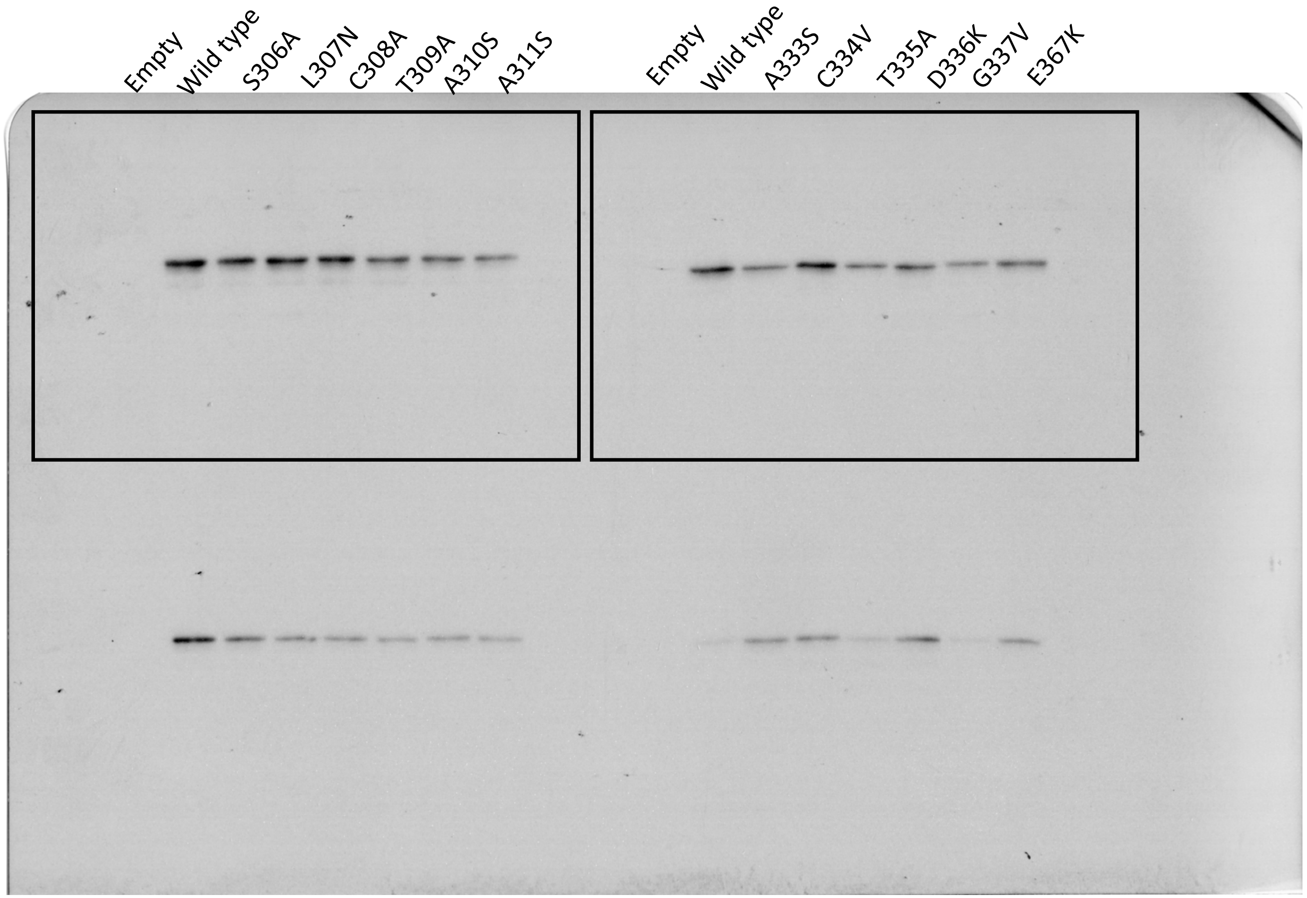

Supplemental Figure: Output ZIKV E-HA replicate 1

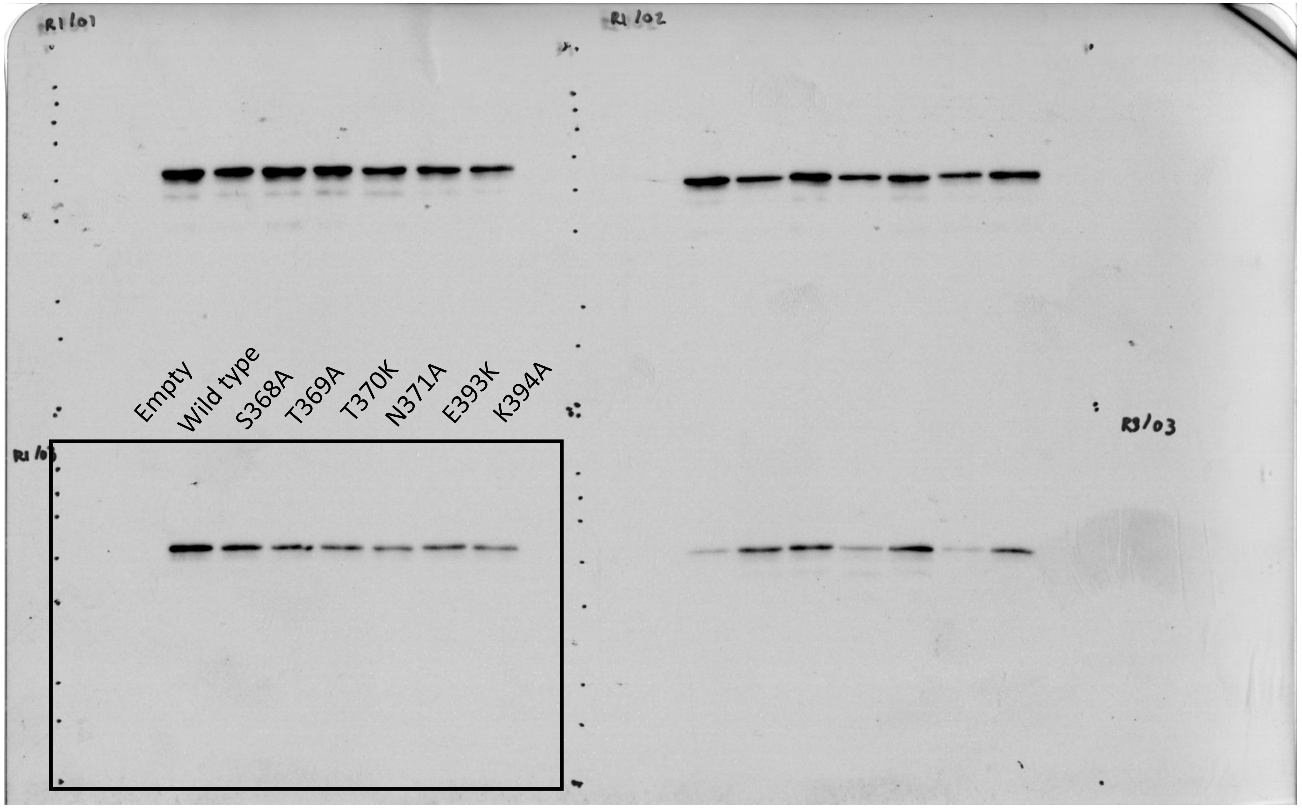

Supplemental Figure: Output EGFP-GRP78 replicate 1

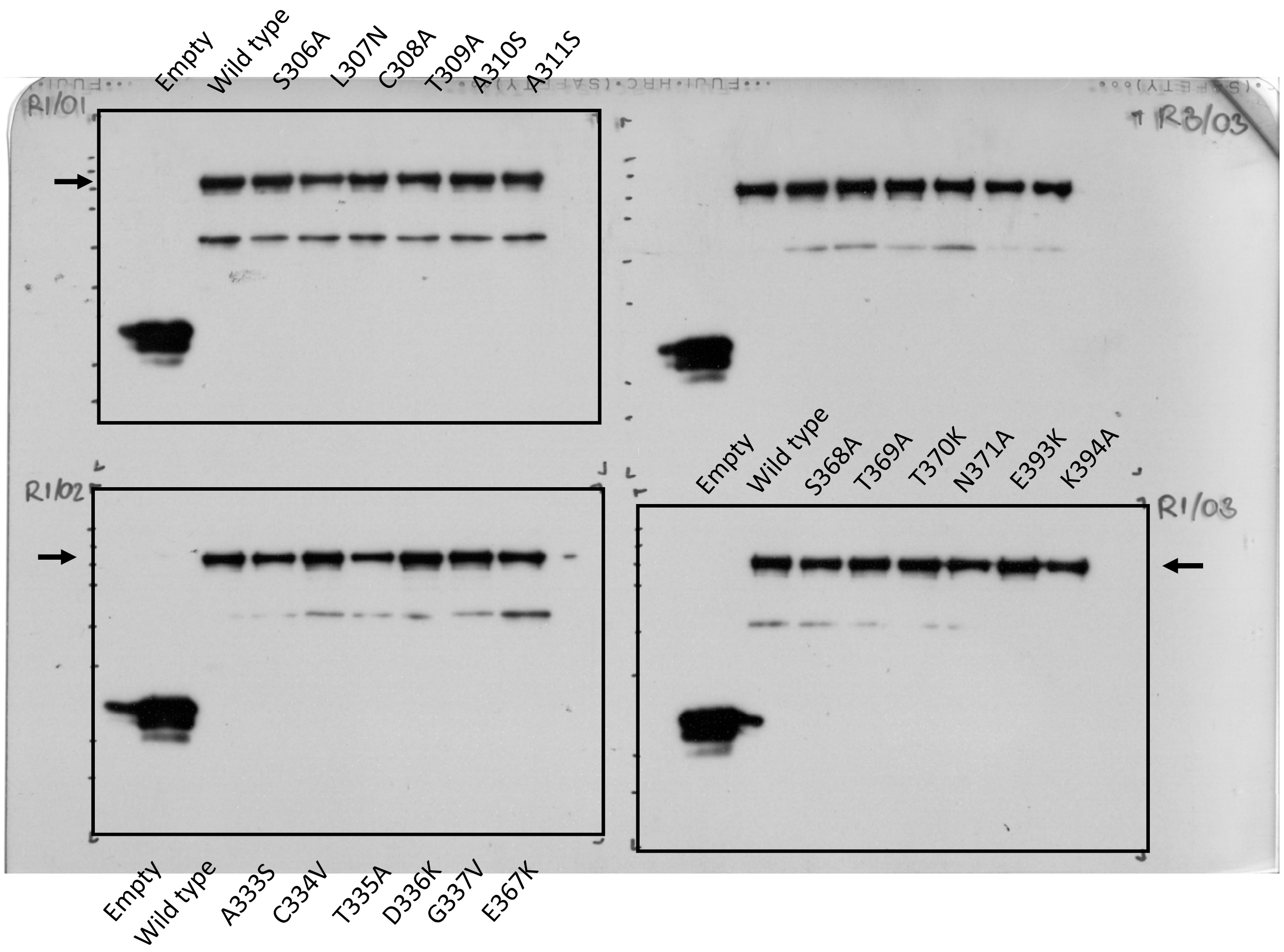

Supplemental Figure: Input ZIKV E-HA replicate 2

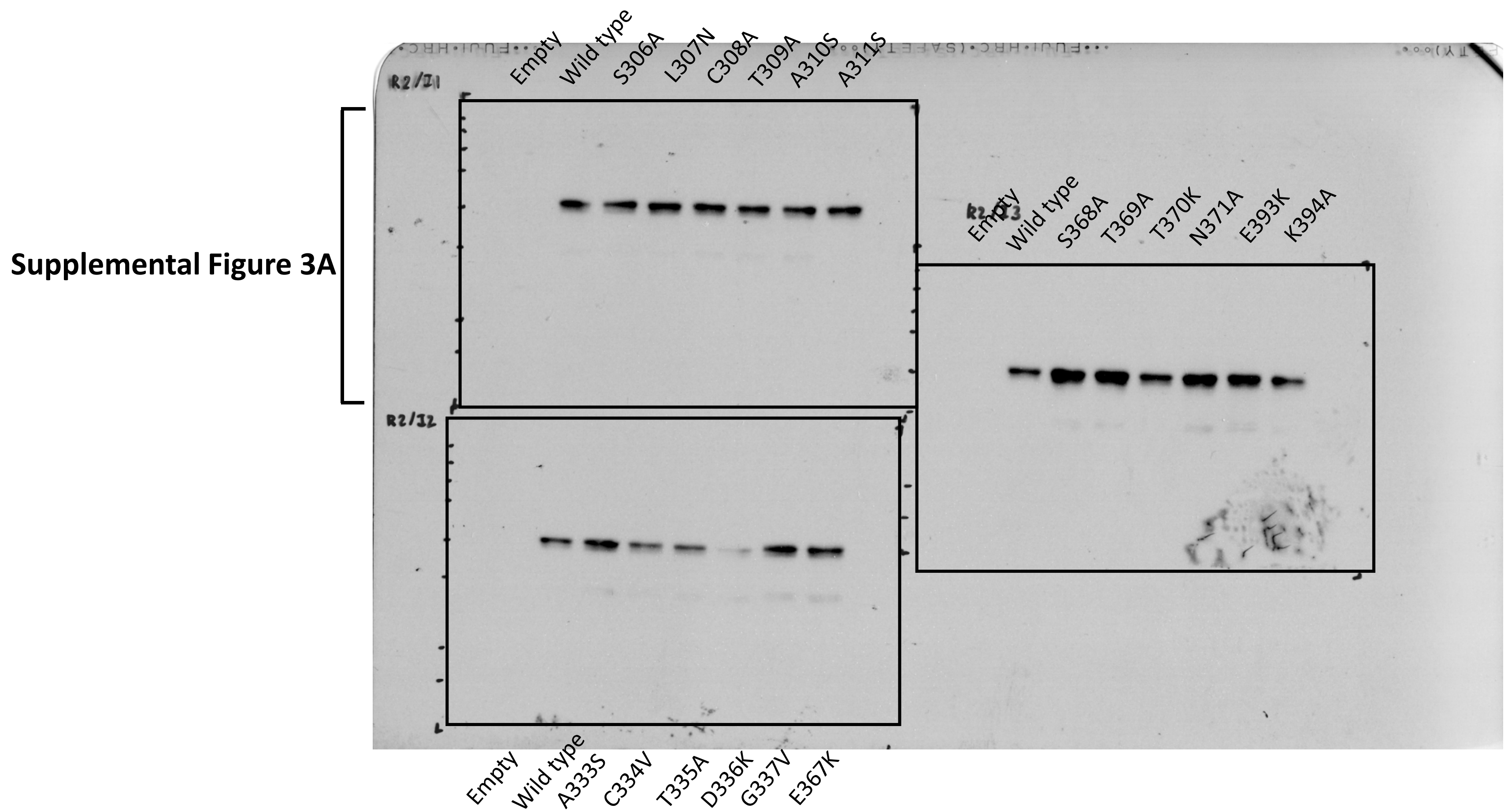

Supplemental Figure: Input EGFP-GRP78 replicate 2

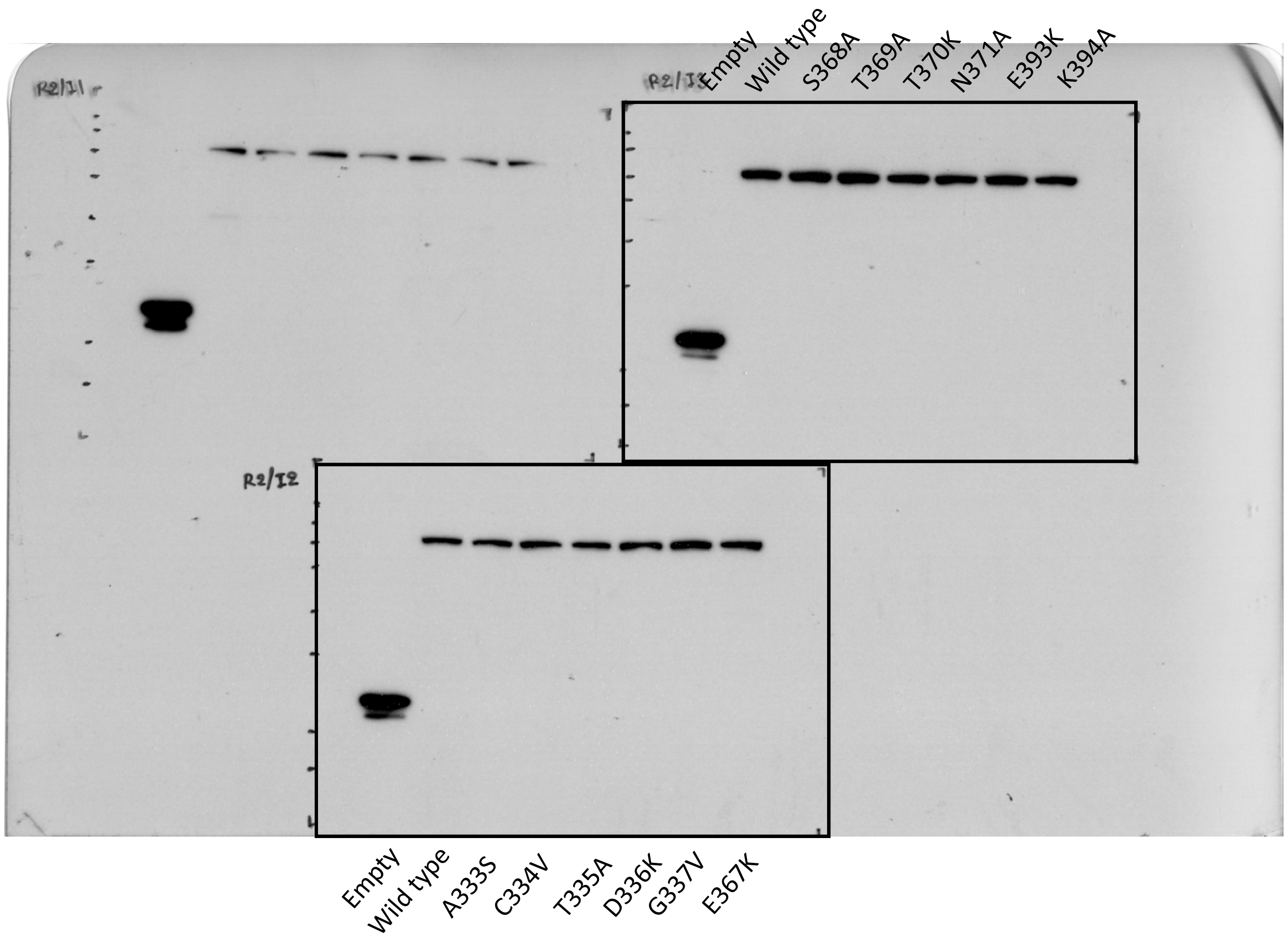

Supplemental Figure: Input EGFP-GRP78 replicate 2

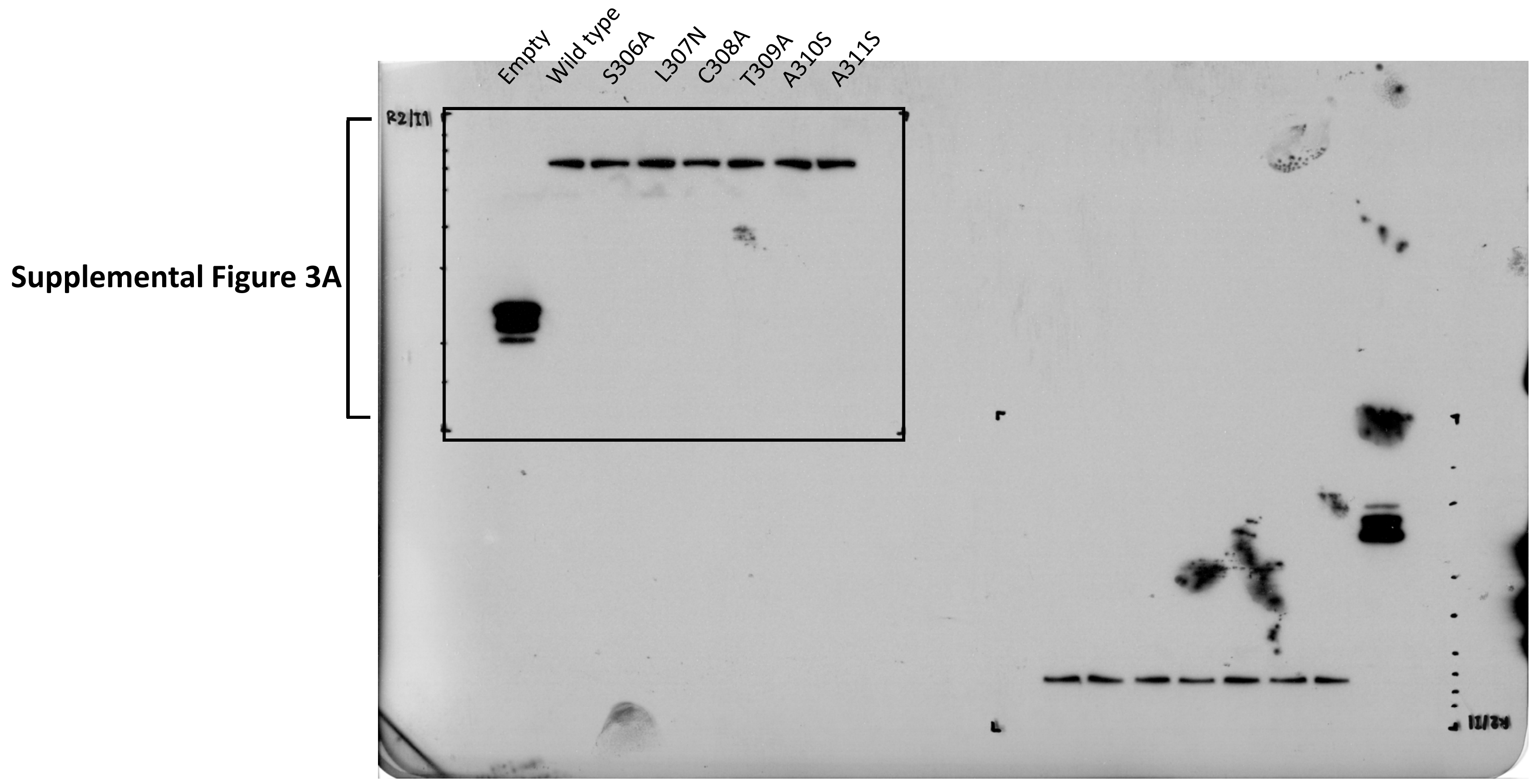

Supplemental Figure: Input Actin replicate 2

Supplemental Figure 3A

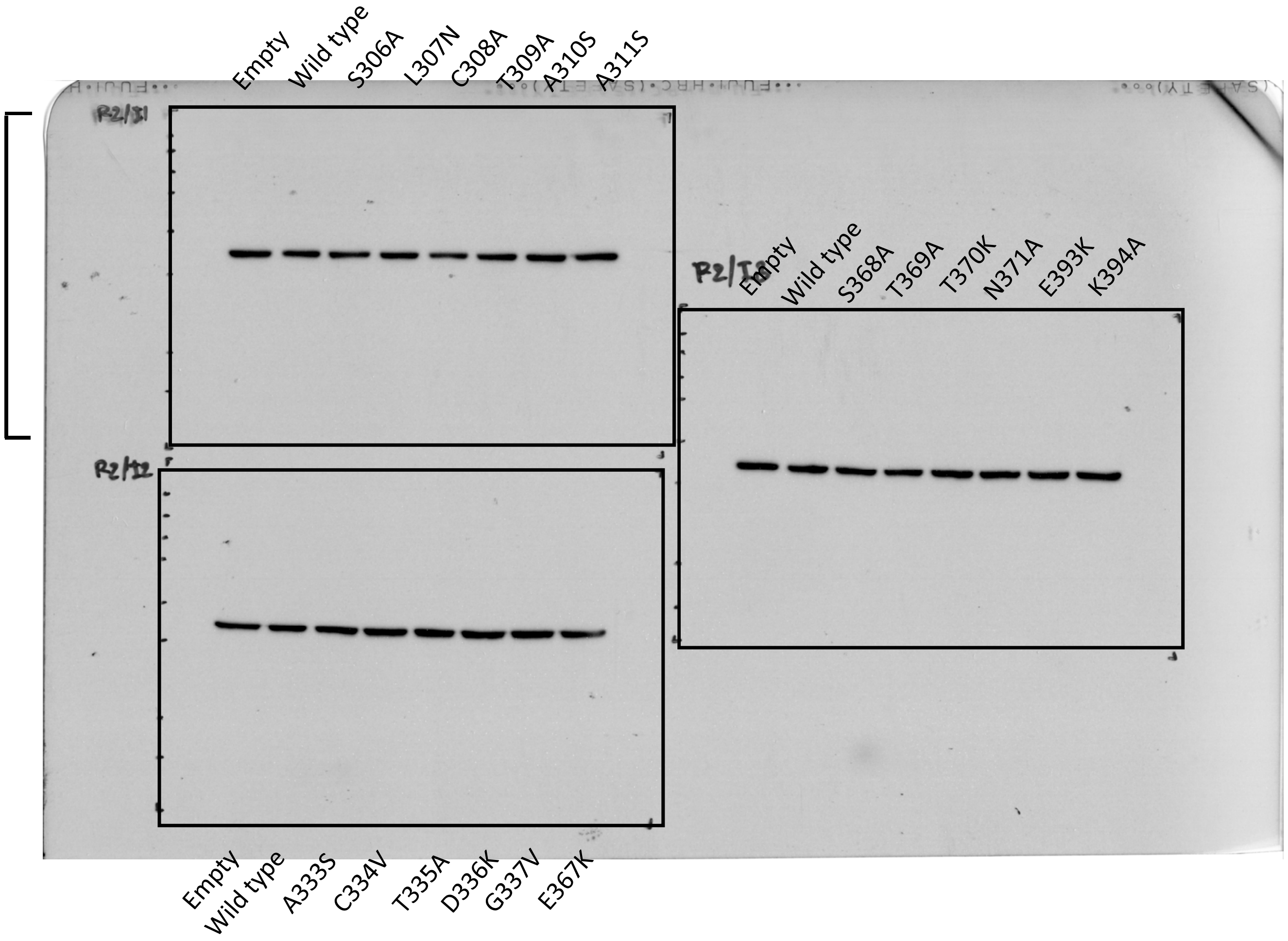

Supplemental Figure: Output ZIKV E-HA replicate 2

Supplemental Figure 3A

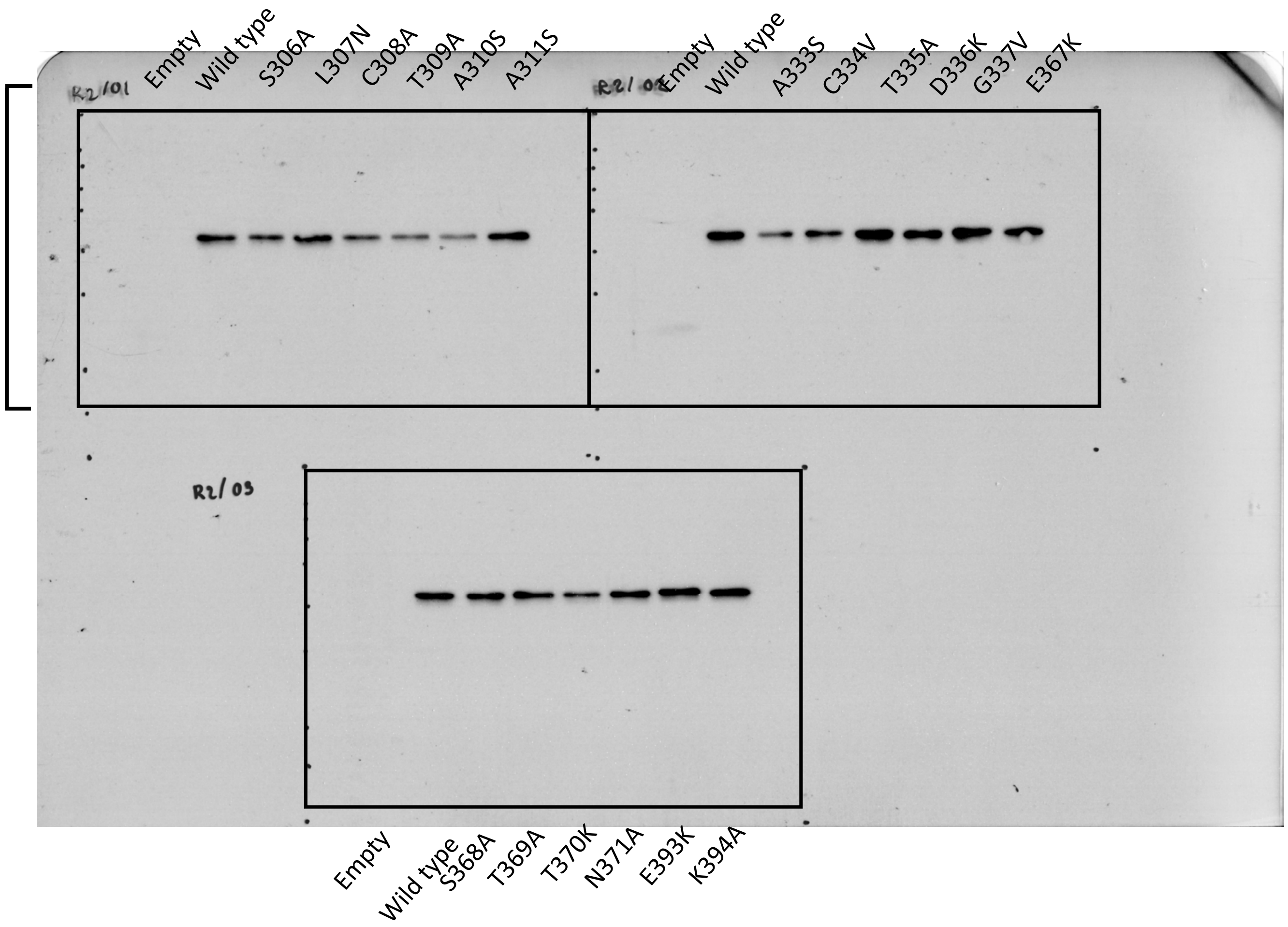

Supplemental Figure: Output EGFP-GRP78 replicate 2

Supplemental Figure 3A

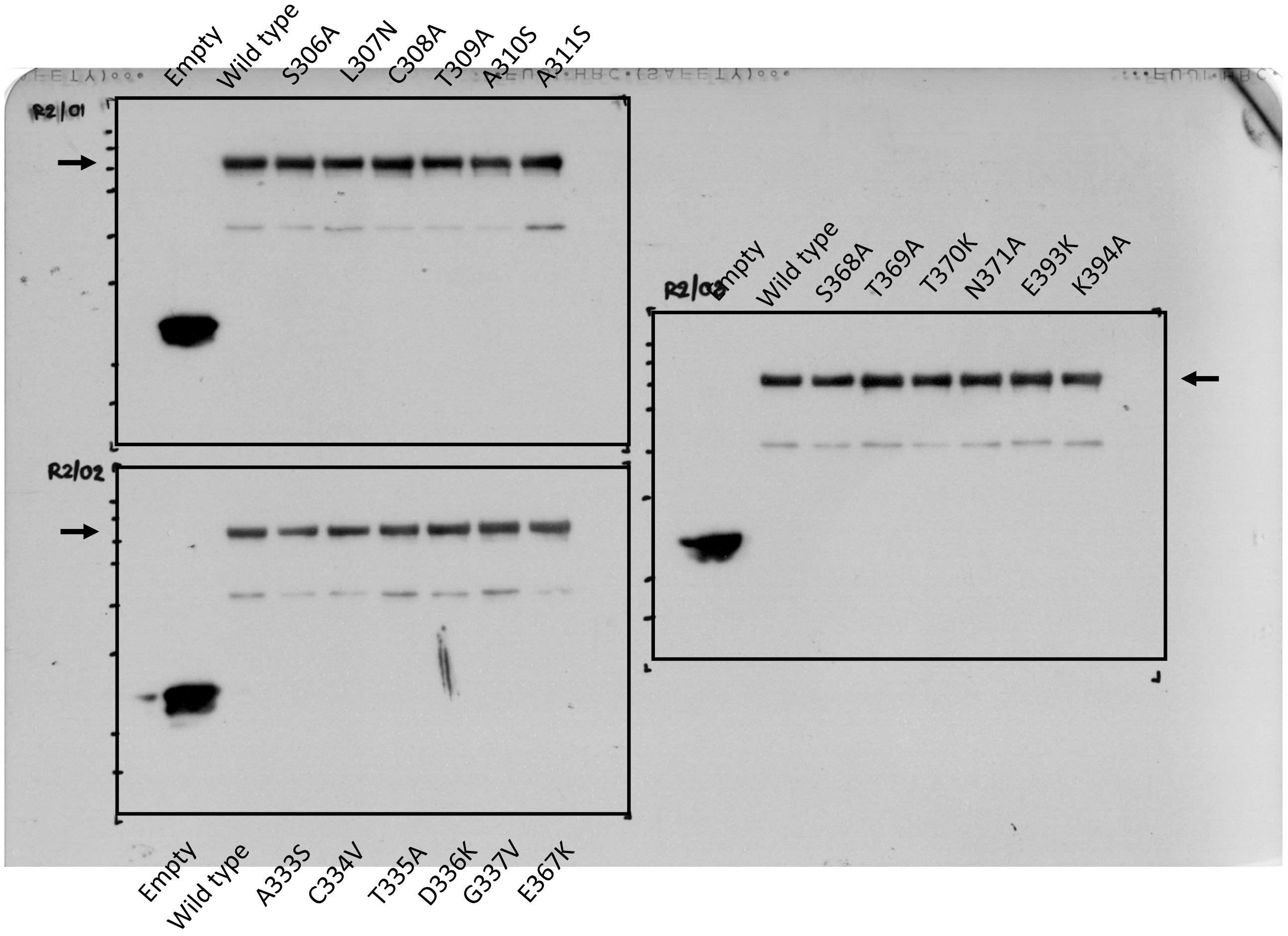

Supplemental Figure: Input ZIKV E-HA replicate 3

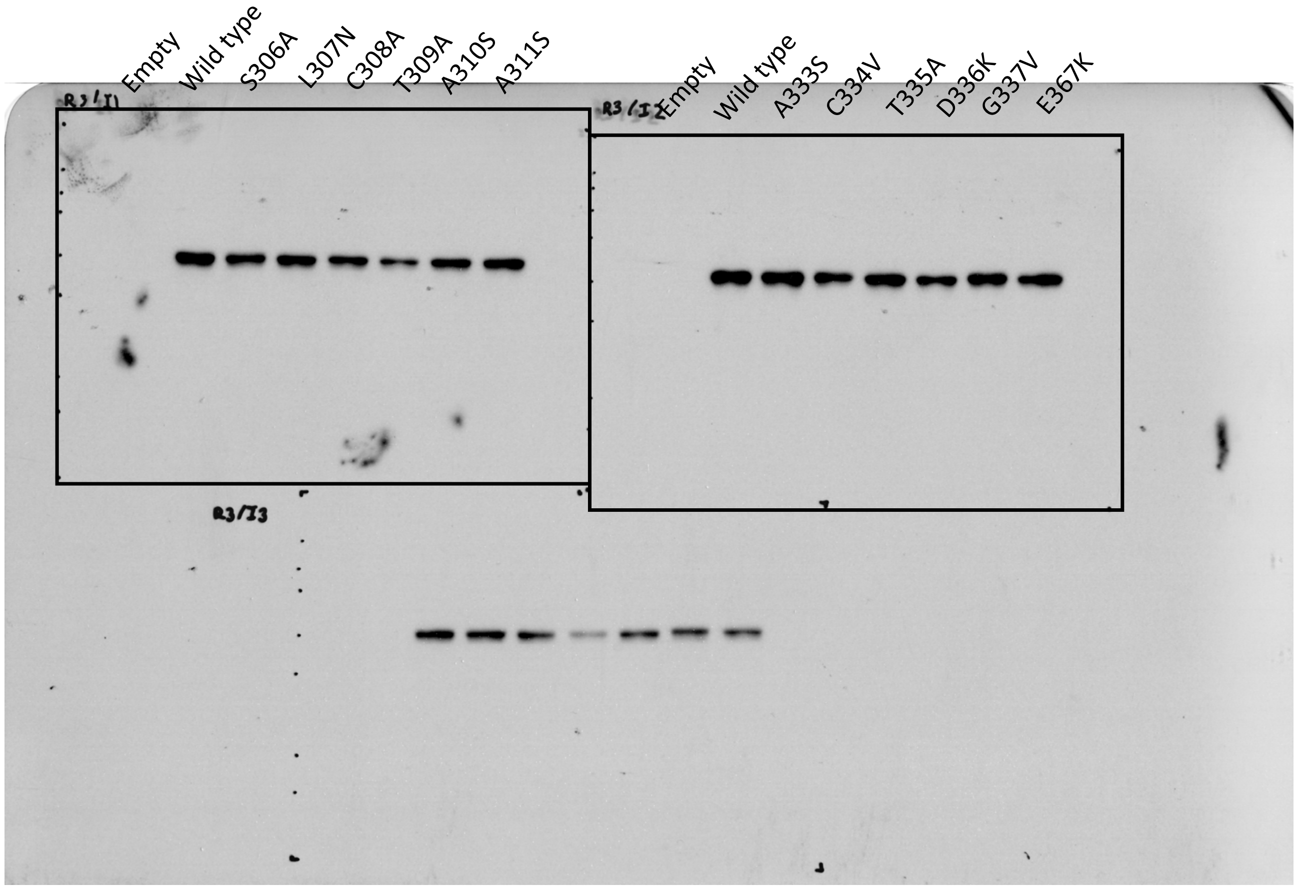

Supplemental Figure: Input ZIKV E-HA replicate 3

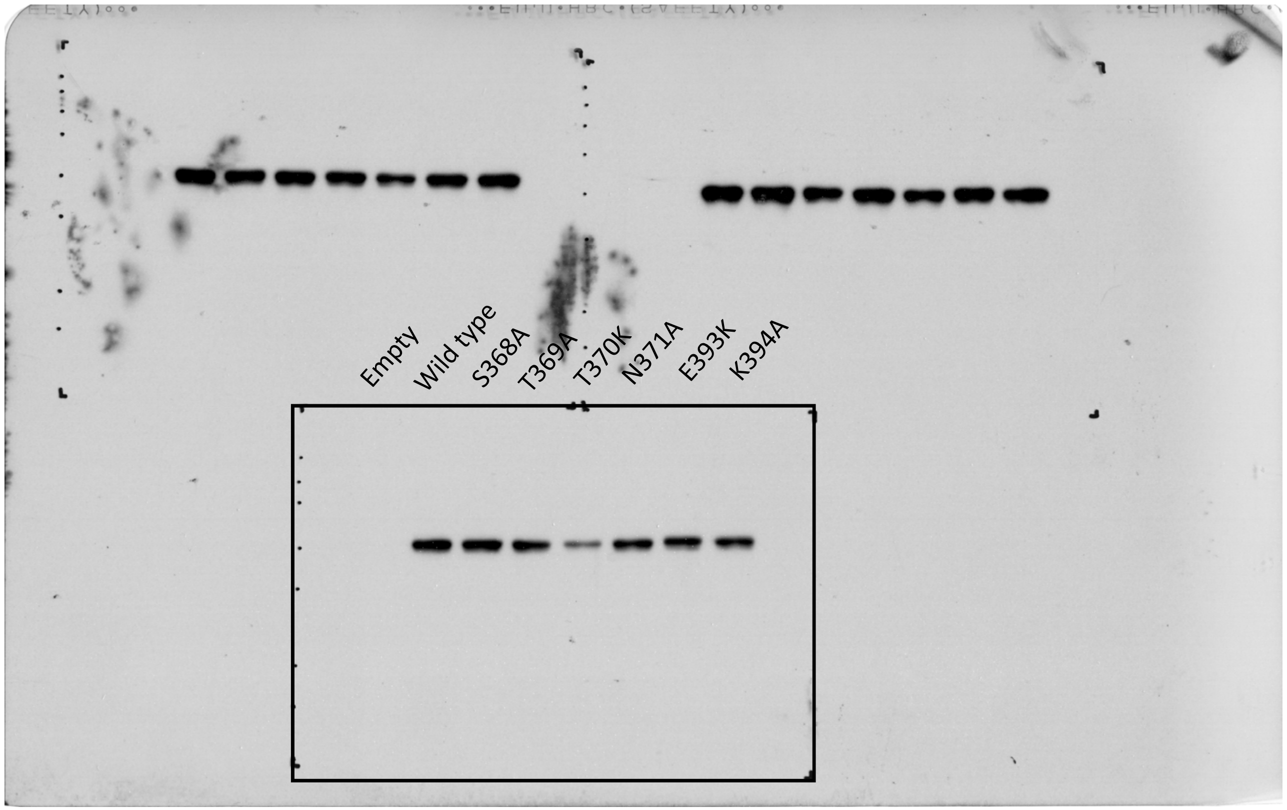

Supplemental Figure 5A

Supplemental Figure: Input EGFP-GRP78 replicate 3

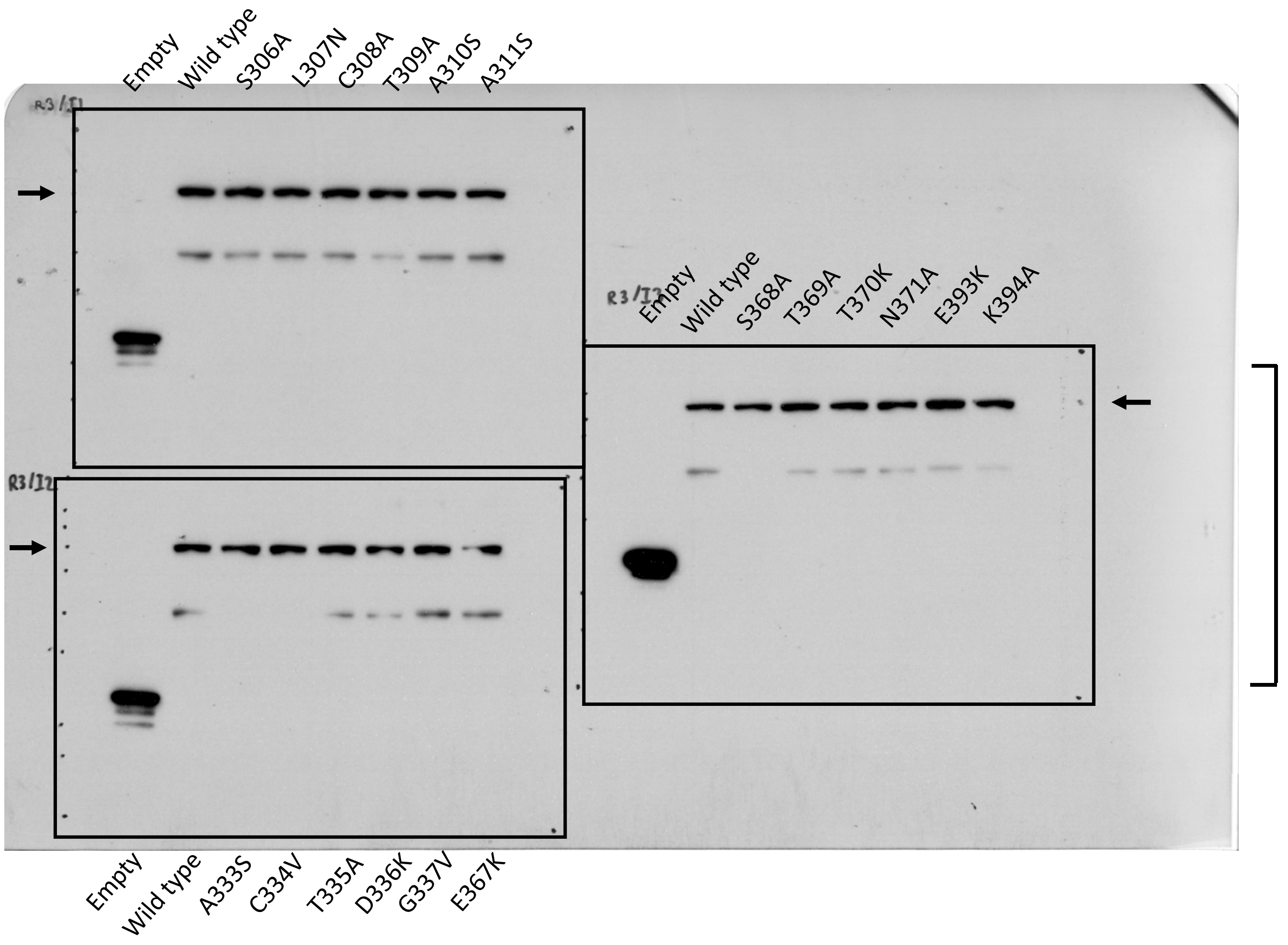

Supplemental Figure 5A

Supplemental Figure: Input Actin replicate 3

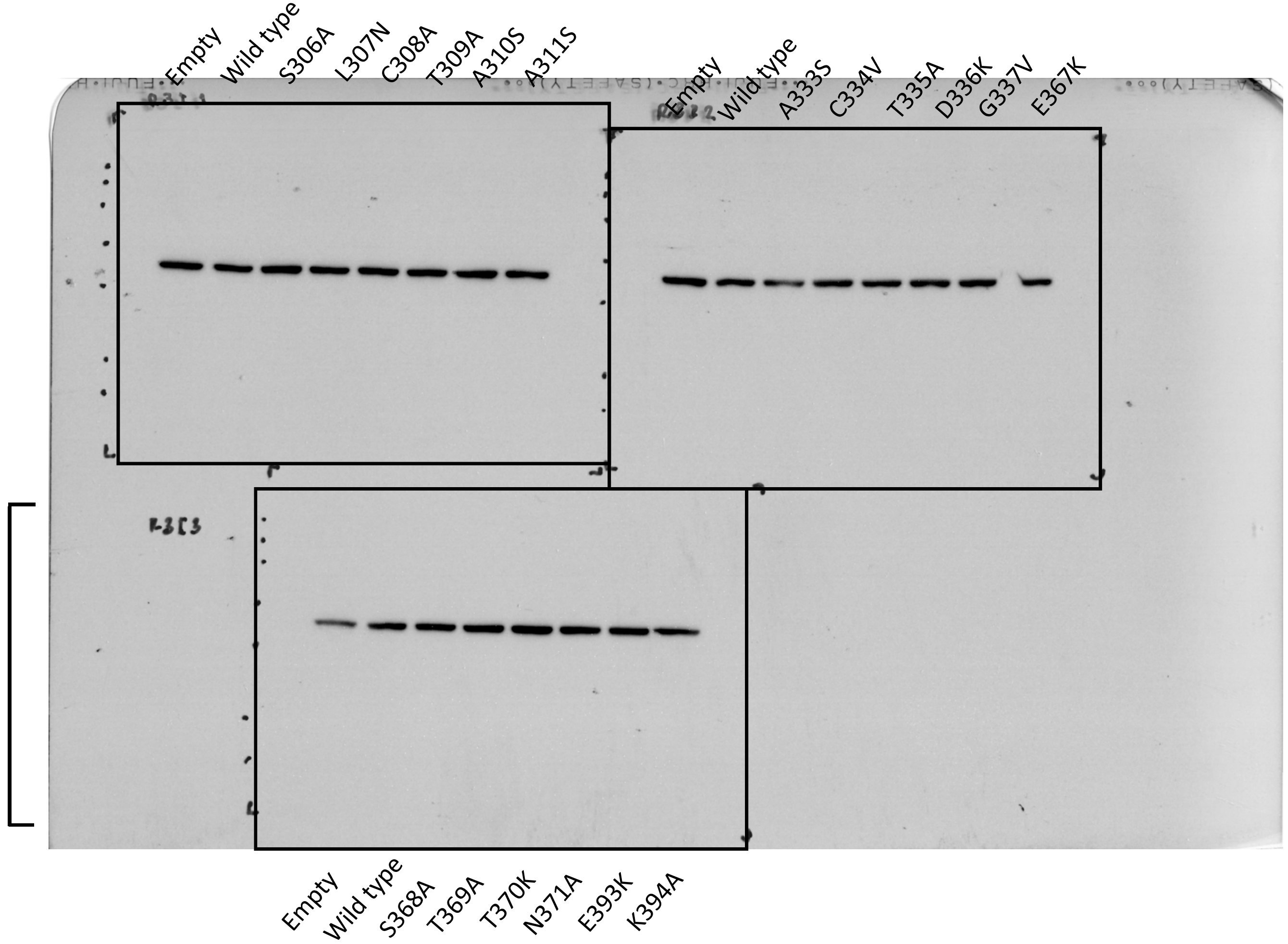

Supplemental Figure 5A

Supplemental Figure: Output ZIKV E-HA replicate 3

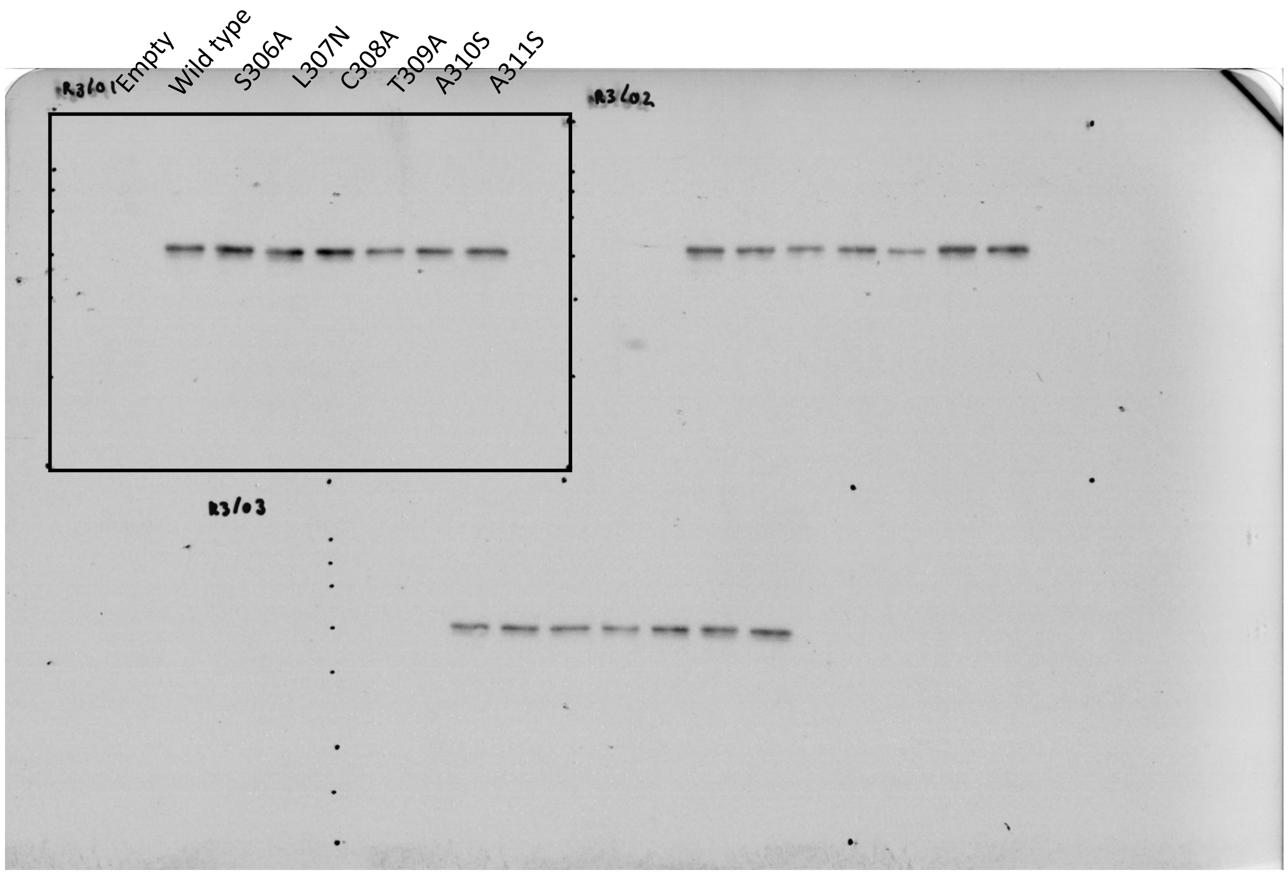

Supplemental Figure: Output ZIKV E-HA replicate 3

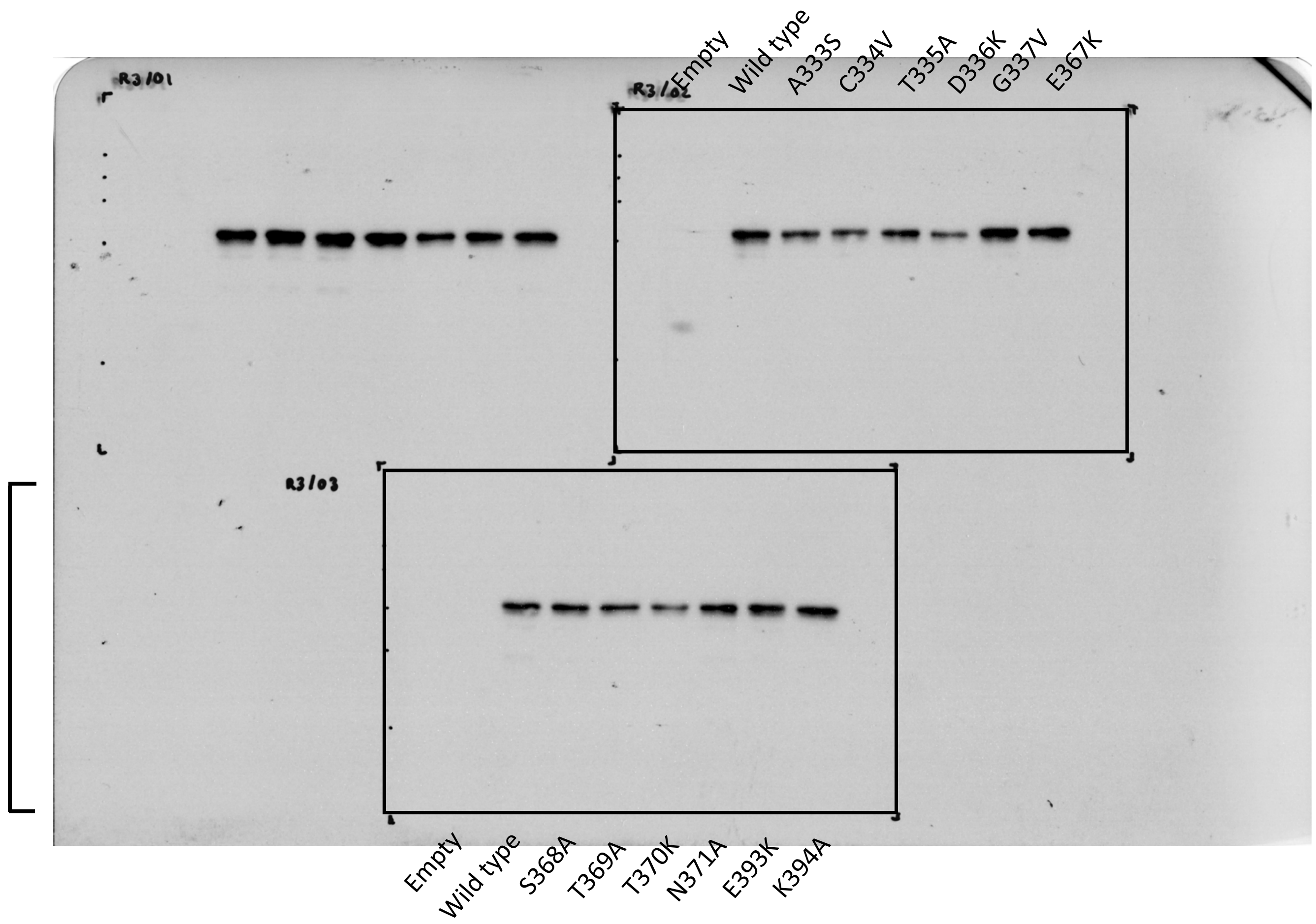

Supplemental Figure 5A

Supplemental Figure: Output EGFP-GRP78 replicate 3

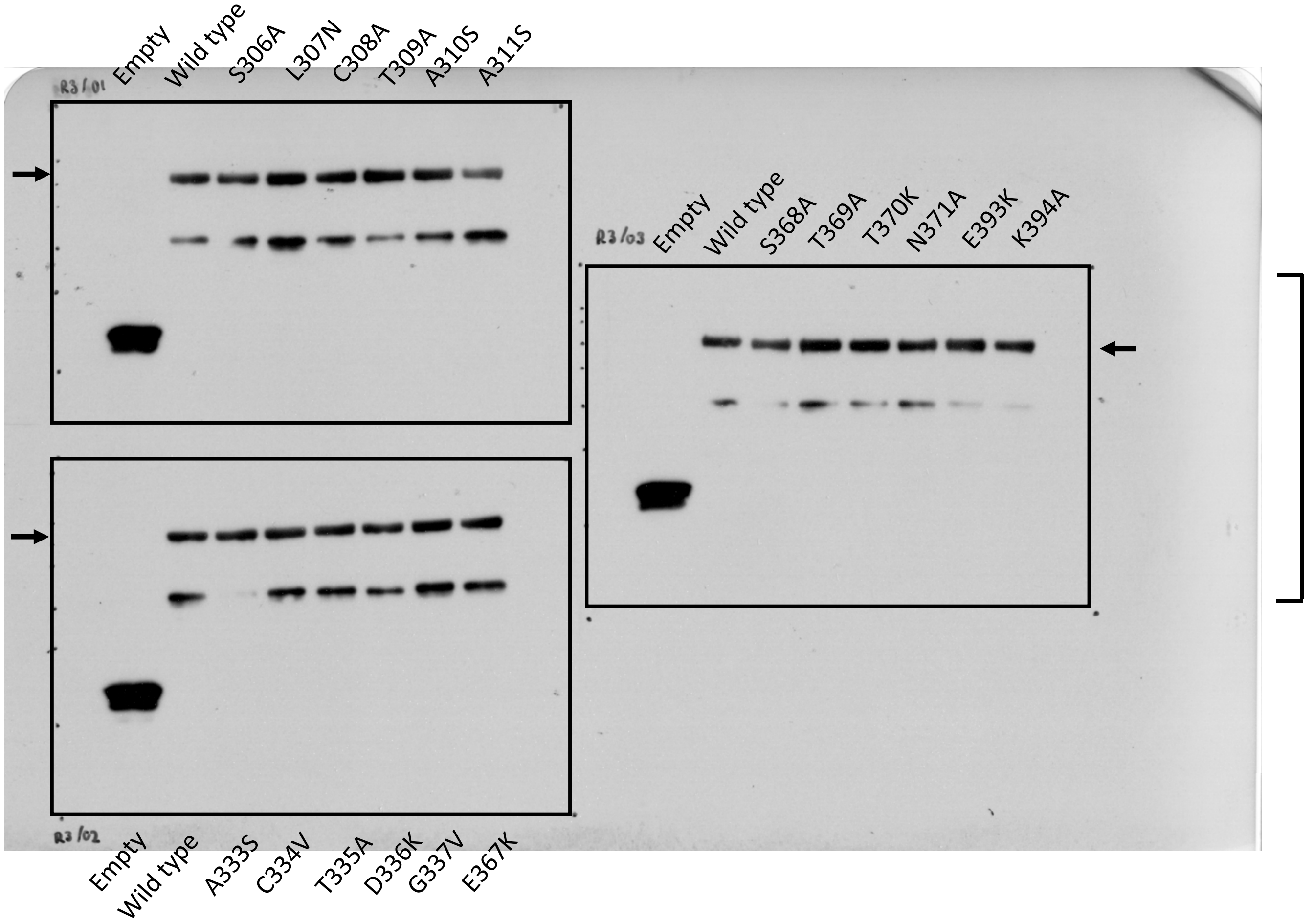

Supplemental Figure 5A

Supplemental Figure: Input ZIKV E-HA replicate 4

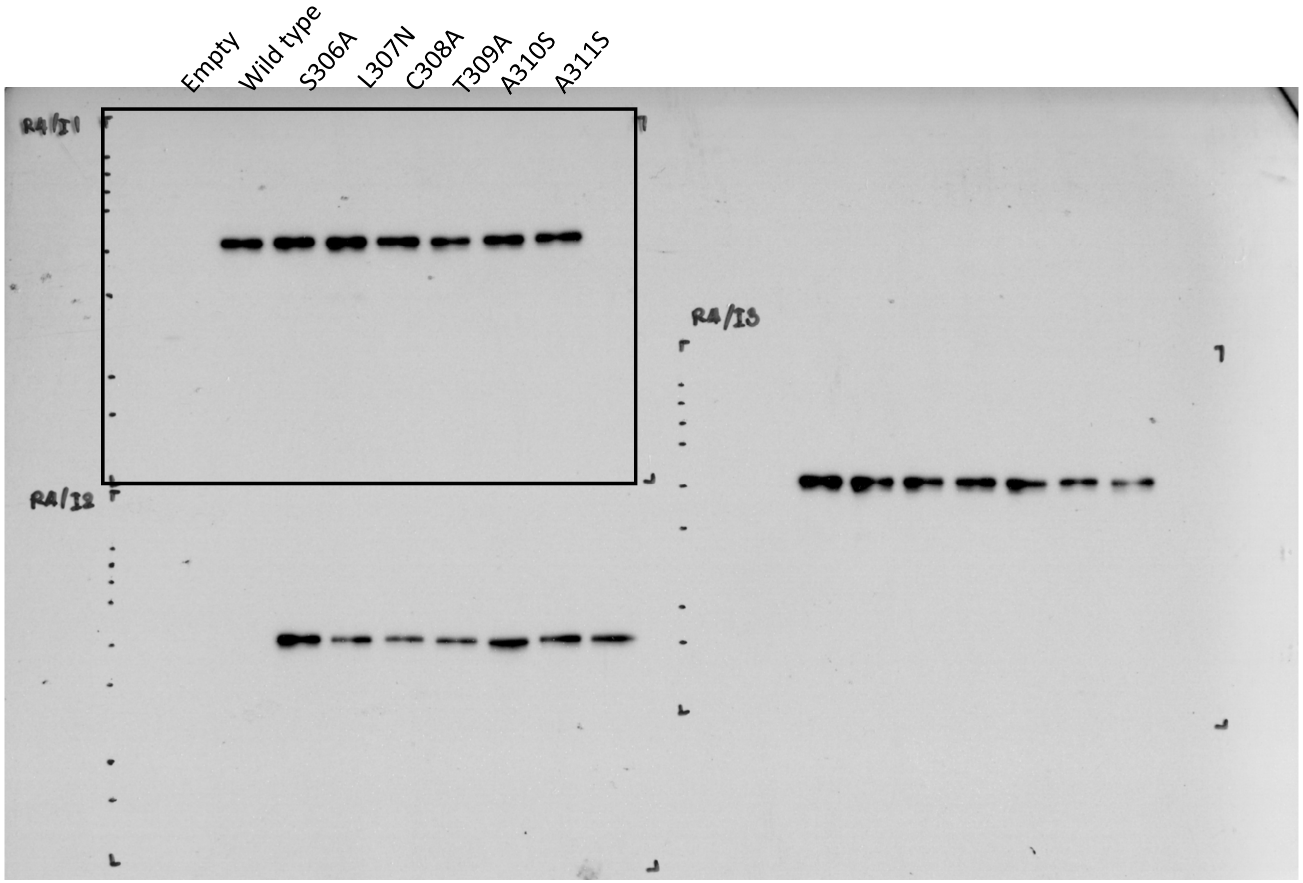

Supplemental Figure: Input ZIKV E-HA replicate 4

Supplemental Figure 4A

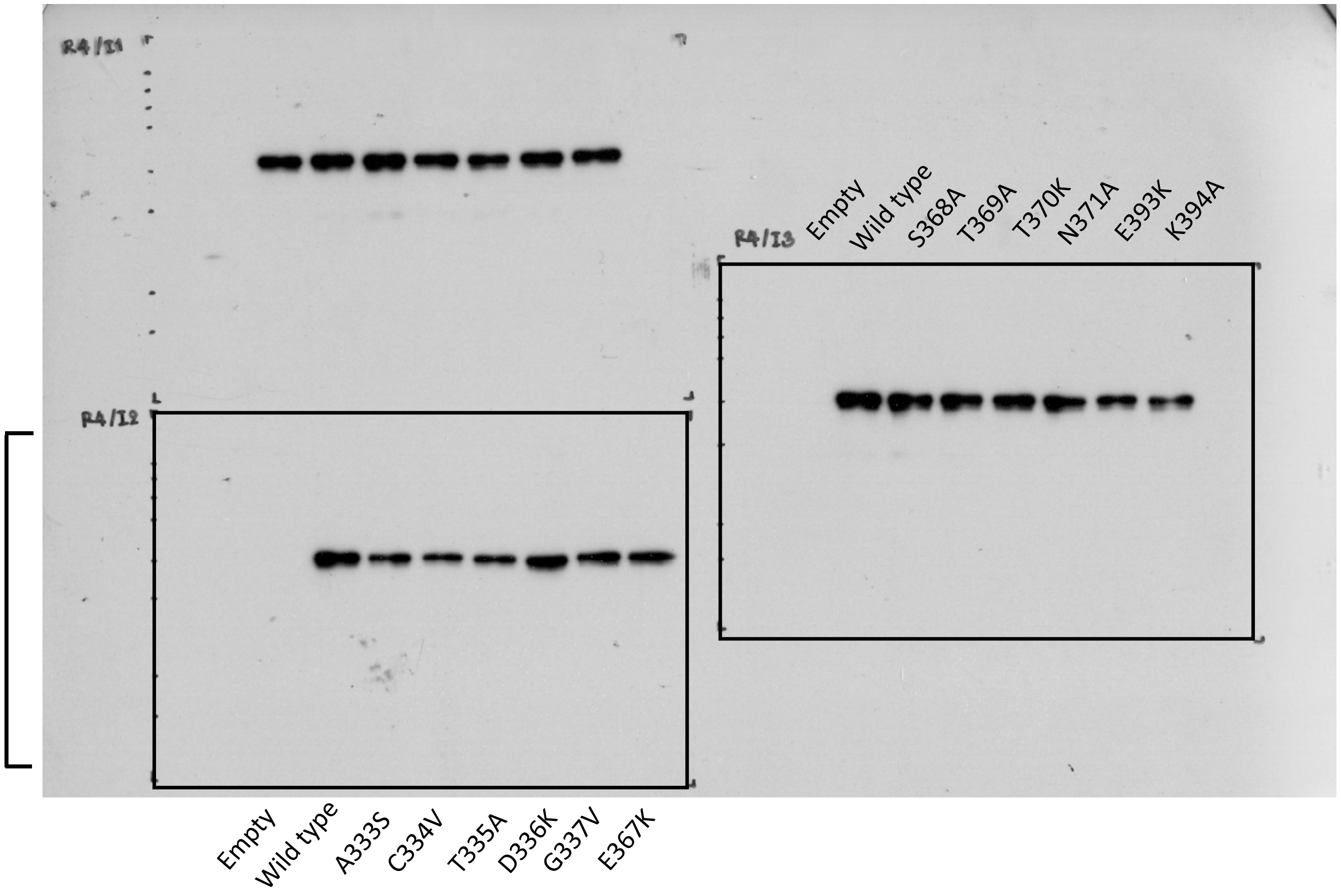

Supplemental Figure: Input EGFP-GRP78 replicate 4

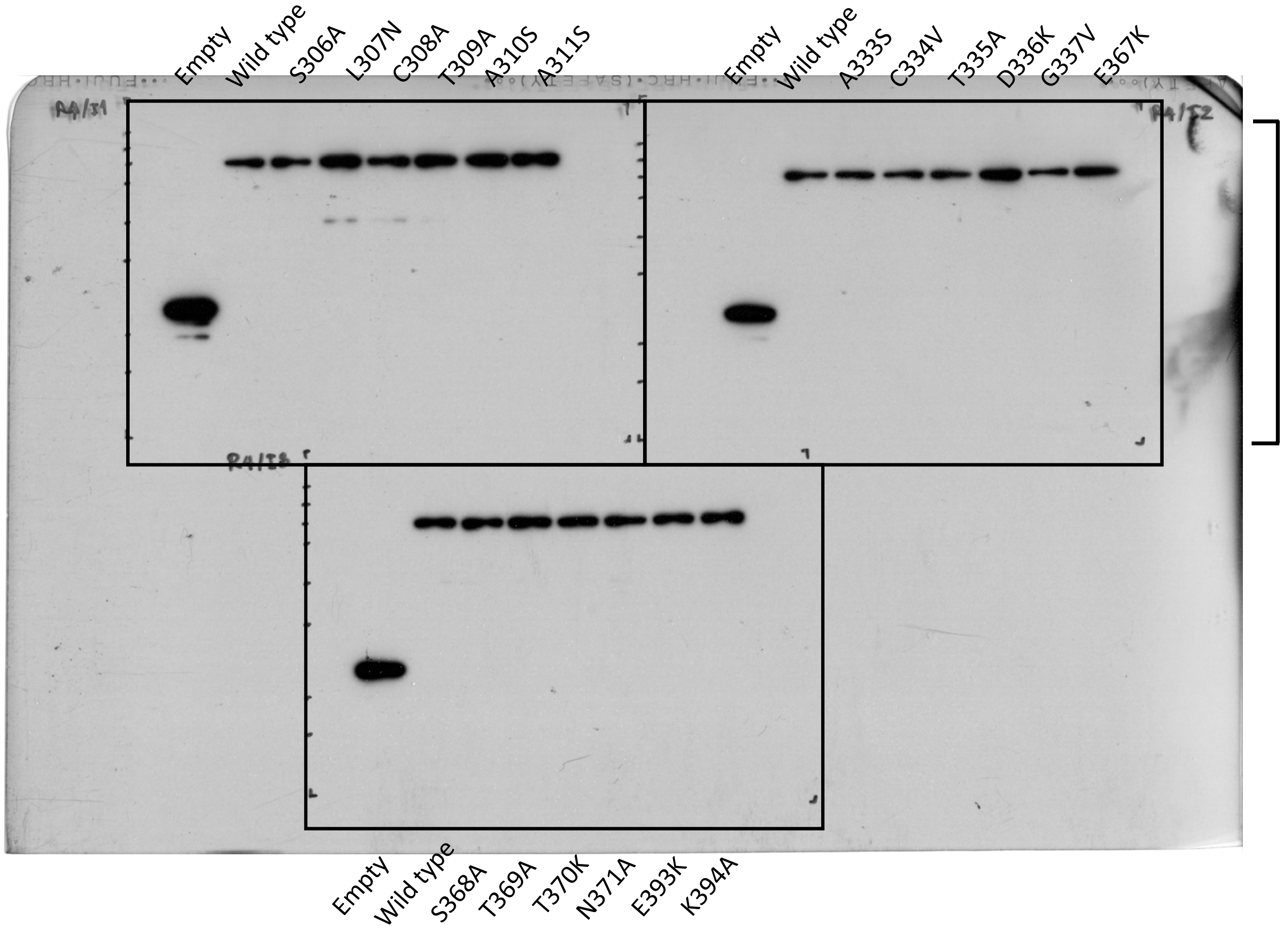

Supplemental Figure 4A

Supplemental Figure: Input Actin replicate 4

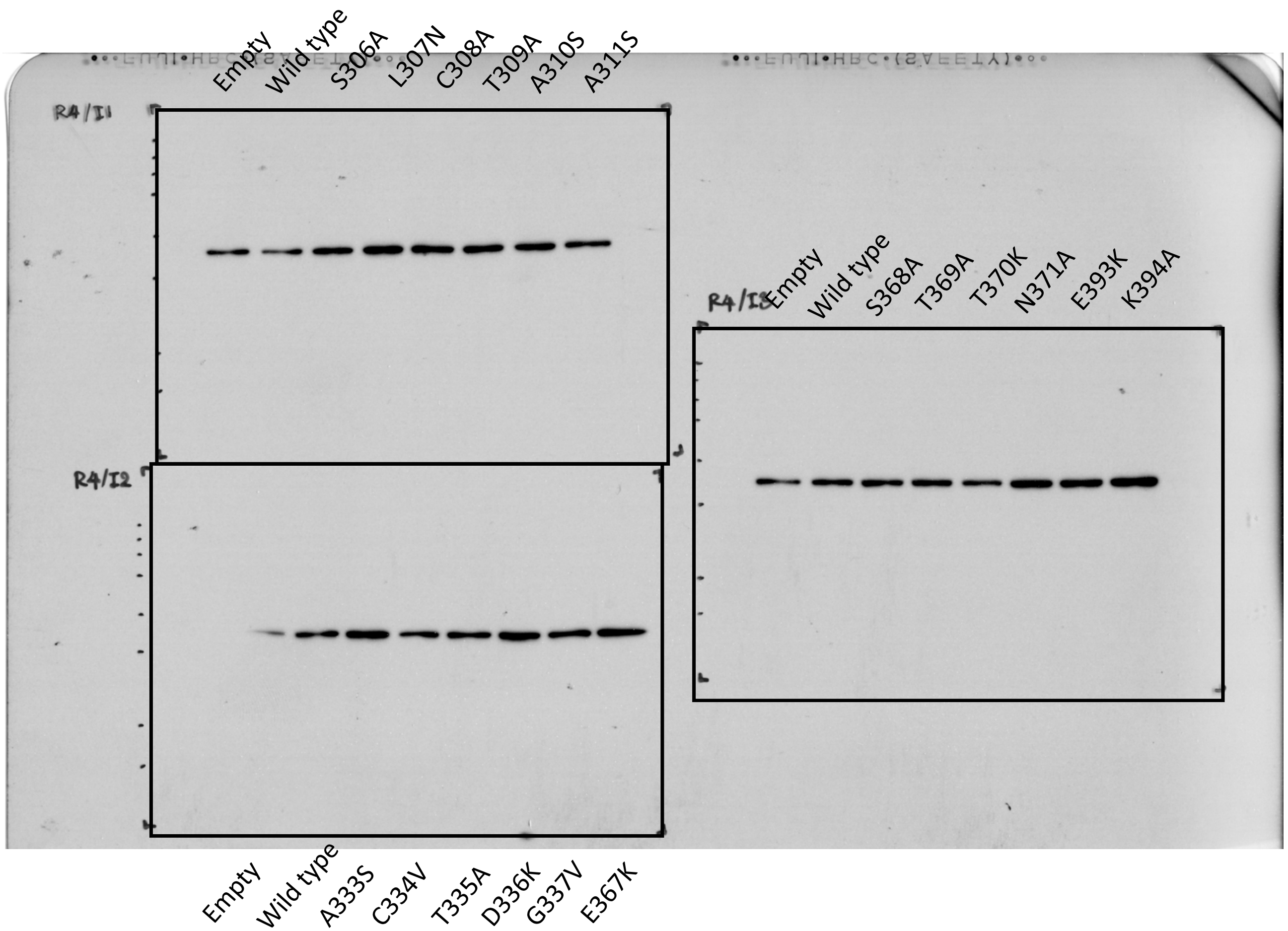

Supplemental Figure 4A

Supplemental Figure: Output ZIKV E-HA replicate 4

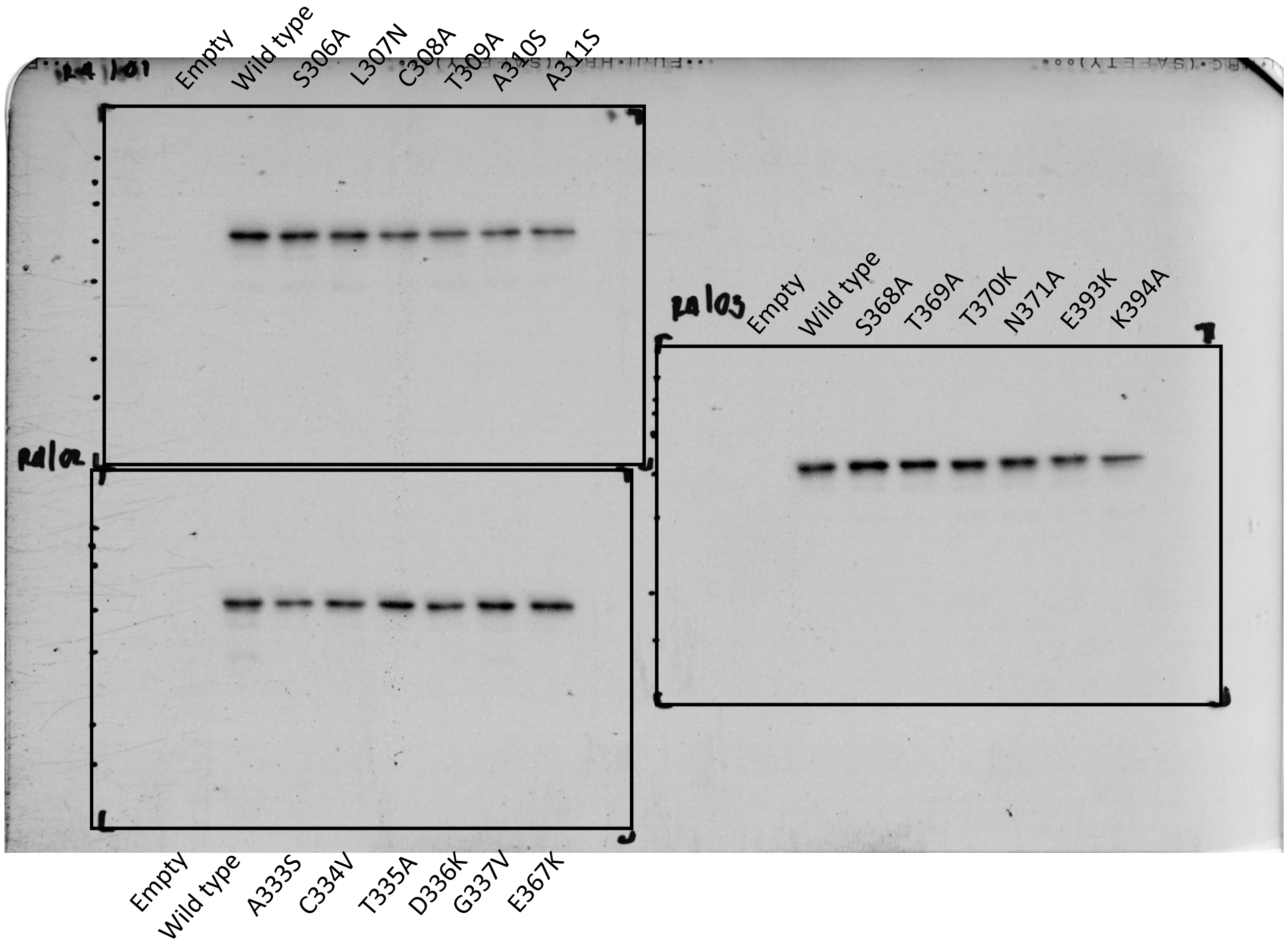

Supplemental Figure 4A

Supplemental Figure: Output EGFP-GRP78 replicate 4

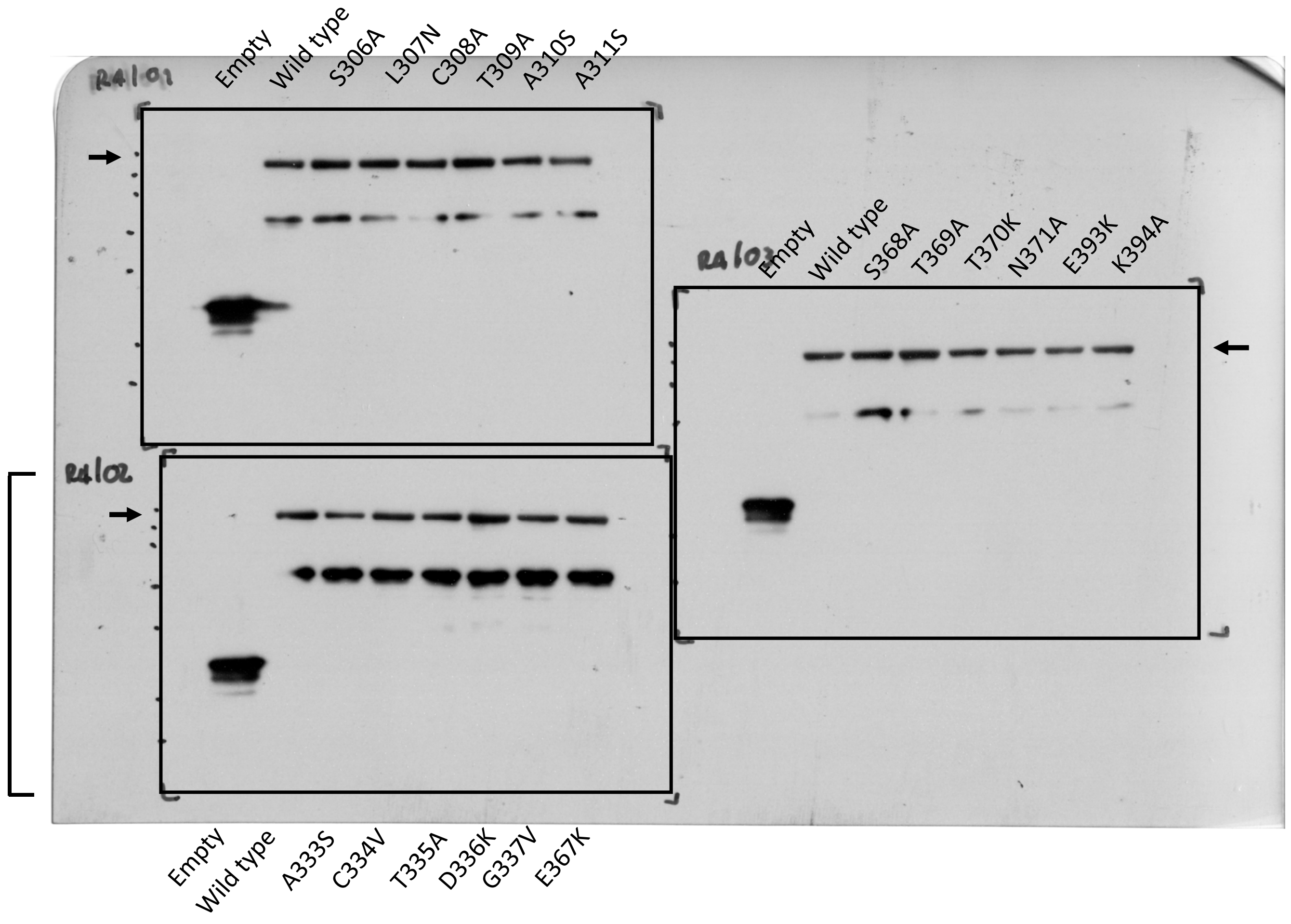

Supplemental Figure 4A
